# The concise bioinspired total synthesis of the 4-hydroxy-2-pyridone family of natural products

**DOI:** 10.1039/d6sc04572c

**Published:** 2026-07-27

**Authors:** Ricardo L. Cruz, Kyu-Hyun Sim, Benjamin N. Chiok, William M. Wuest

**Affiliations:** a Emory University, Department of Chemistry 1515 Dickey Dr. Atlanta GA 30345 USA wwuest@emory.edu

## Abstract

Herein, an asymmetric and modular total synthesis of 4-hydroxy-2-pyridone natural products, a promising class of antifungal alkaloids, is disclosed. Leveraging a key 3,5-dihalogenated pyridone common precursor, five natural products are afforded by an iterative regioselective Suzuki–Miyaura coupling and a bioinspired Knoevenagel condensation. The latter highlights the power of the approach by coupling a highly functionalized lactol fragment with high diastereoselectivity in the final step. This approach furnished the natural products (−)-sambutoxin, (−)-sambutoxin A, (−)-oxysporidinone, (−)-fusapyridone A, and (+)-fusoxypyridone B efficiently (12-14 steps, longest linear sequence) and in high overall yield (4.7–9.1%). This concise, modular approach leveraging a privileged linchpin core unlocks access to the 4-hydroxy-2-pyridone class of natural products and will enable detailed biological studies going forward.

## Abstract 2

Leveraging divergence from an easily accessible linchpin, we report the total synthesis of five pyridone alkaloids, relying on a bioinspired Knoevenagel condensation to unify two complex synthetic intermediates with high stereospecificity.

## Graphical abstract

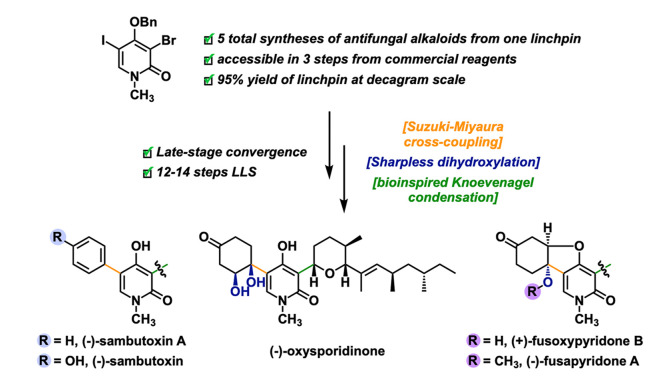

## Introduction

**Fig. 1:**
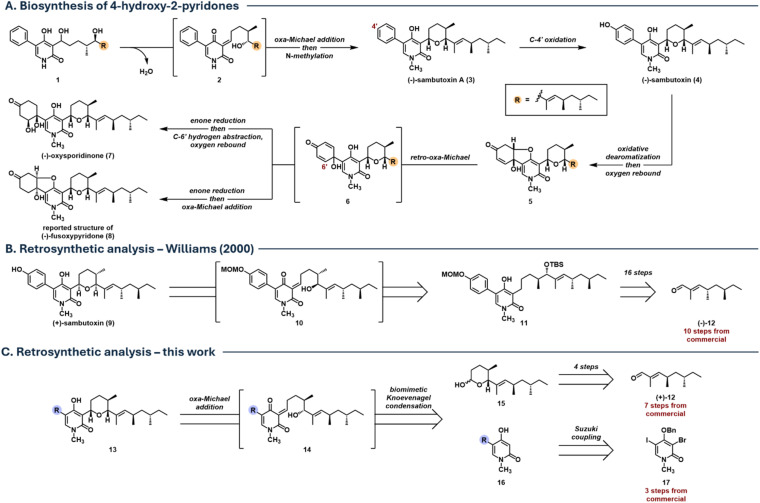
Putative biosynthesis of 4H2Ps (A) and comparison of retrosynthetic approaches by Williams (B) and Wuest (C).

Natural products play a significant role in the development of modern medicine. Over 55% of FDA-approved drugs developed since 1981 either are natural products or are inspired by them.^1^ To this end, accessing these scaffolds in an efficient and high-yielding manner to facilitate drug discovery is essential. One major development in the improvement of discovery efficiency has been the widespread adoption of modular strategies in natural product total synthesis, whereby common intermediates or building blocks facilitate the synthesis of various members of a natural product family or unnatural synthetic derivatives therein.^2–4^ Two seminal examples include the work by the Burke group leveraging their MIDA-linchpin technology for efficient biaryl coupling, as well as that of the Myers lab in developing tetracycline derivatives in a convergent fashion.^5,6^ The 4-hydroxy-2-pyridone (4H2P) family of natural products is reported to possess a wide array of bioactivities, and the structural similarities across the entire natural product family made these compounds attractive targets to pursue through a divergent total synthesis strategy.^7,8^ Given the plethora of work around the biosynthetic origins of these molecules, we sought to leverage key insights from these studies. For example, we quickly recognized that Nature forges the key tetrahydropyran-bond disconnection *via* dehydration of compound 1 followed by an oxa-Michael transformation to directly afford (−)-sambutoxin A (3) after *N*-methylation (Fig. 1A).^9^ From there, Nature alternates the oxidation state of this precursor to arrive at a family of natural products including (−)-sambutoxin A (3), (−)-sambutoxin (4), (−)-oxysporidinone (7), and the unique fusoxypyridone scaffold (8) bearing a [6-5-6] ring system.^10–13^ These insights will be critical in the synthetic design presented herein.

The 4H2P class of compounds has garnered much attention in the realm of total synthesis due to their unusual structures and promising bioactivities.^14–18^ In particular, Williams' landmark total synthesis of the antipodal (+)-sambutoxin (9) leveraged a bio-inspired construction of the tetrahydropyran ring *via* Saegusa–Ito oxidation to intermediate 10 (Fig. 1B).^19^ This key intermediate was accessed in short order from complex precursor 11, which required 12 synthetic steps to access from chiral aldehyde (−)-12, itself demanding the investment of an additional 10 steps. Albeit quite impressive, the laborious and linear nature of this route limits the overall yield and the ability to access other members of the family. Taking these limitations into account, we sought to develop a method to access the 4H2P natural product family (13) in a concise fashion amenable to diversification. Inspired by Nature, we envisioned using an analogous Michael acceptor species (14) to furnish the tetrahydropyran motif at a late stage through a convergent strategy (Fig. 1C). Similar reactivity has previously been reported in the synthesis of similar natural products – previous efforts by Fotiadou and Zografos have accomplished C3 alkylation and leveraged similar intermediates in the construction of the related 4H2P natural product septoriamycin A, making this sort of approach attractive.^20^ Paramount to the success of this proposal was a bioinspired Knoevenagel condensation between a highly functionalized lactol electrophile (15) and a fine-tuned 4H2P nucleophile (16) to generate the desired intermediate 14 following dehydration. The corresponding 4H2P could be assembled in an iterative manner from a privileged dihalogenated common intermediate (17), accessible in three steps from commercial starting materials. In terms of the lactol coupling partner, asymmetric crotylboration of aldehyde (+)-12, accessible in 4 steps from known intermediates, would set the key stereocenters of the incoming tetrahydropyran.^21,22^

## Results and discussion

**Fig. 2:**
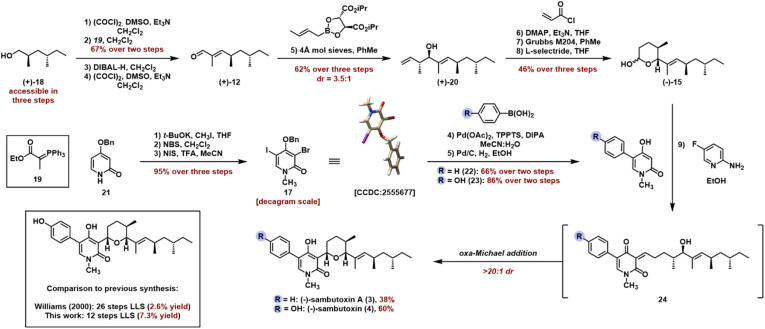
Total synthesis of (−)-sambutoxin A (3) and (−)-sambutoxin (4).

In the forward sense, Swern oxidation of the known alcohol (+)-18 followed by direct subjection of the aldehyde product to Wittig olefination conditions with ylide 19 afforded the α,β-unsaturated ester as a single diastereomer (Fig. 2).^23^ DIBAL-H reduction to the allylic alcohol and Swern oxidation gave aldehyde (+)-12, which was directly subjected to asymmetric crotylation with freshly prepared (*S*,*S*)-diisopropyltartrate-derived crotylboronate to furnish alcohol (+)-20 as a 3.5 : 1 ratio of separable diastereomers.^24,25^ Acylation of (+)-20 with acryloyl chloride gave a diene, which readily underwent intramolecular ring-closing metathesis following treatment with Grubbs Catalyst M204 to afford the α,β-unsaturated lactone. Gratifyingly, we found that treatment of this lactone with excess l-selectride facilitated both 1,4- and 1,2-reduction in one pot to the desired lactol scaffold (−)-15. With the chiral lactol in hand, we first set our sights on preparing the corresponding 4H2P coupling partners that would provide access to sambutoxin-type natural products. To this end, commercial 4H2P 21 readily underwent *N*-methylation followed by regioselective bromination and iodination to give the dihalogenated common intermediate 17, as further confirmed by X-ray crystallography [CCDC: 2555677].^26^ The differential halogenation of this species, key to the success of the proposed modular design, facilitated a highly regioselective Suzuki–Miyaura coupling with either phenylboronic or 4-hydroxyphenylboronic acid to afford the corresponding bromopyridones. Hydrogenolysis of both species mediated one-pot benzyl deprotection and debromination to give the 4H2P fragments 22 and 23, now poised for condensation.

During our initial condensation optimization, we found that secondary amine bases such as piperidine were particularly productive. This also extended to primary amine bases, which led to our identification of 2-amino-5-fluoropyridine (2A5F) as an ideal base for this condensation system in protic solvents such as ethanol (Fig. S1).^27,28^ Our observation that tertiary amines provided significantly lower conversion, as well as the detection of the cyclic hemiaminal present in the reaction mixture, suggested that this condensation proceeded through an underlying organocatalytic mechanism. This revealed that 2A5F not only generated a pyridone enolate but also enhanced reactivity through the formation of an imine intermediate. This imine is then intercepted by the 4H2P nucleophile to give a transient linear intermediate, whereby loss of 2A5F generates the Michael acceptor species (24) necessary for tetrahydropyran construction. Importantly, we also found this condensation to be highly diastereoselective towards the formation of the 2,6-*cis*-tetrahydropyran product, likely owing to templating by the incoming chiral lactol in combination with the thermodynamic preference for improved conformational stability relative to the competing 2,6-*trans*-tetrahydropyran isomer (Fig. S2).^29^ Taken together, the Knoevenagel condensation of lactol (−)-15 and 4H2P 23 mediated the total synthesis of (−)-sambutoxin (3) in a 12 step LLS and 7.3% overall yield from commercial starting materials. Our convergent approach is highly efficient when compared to Williams' elegant total synthesis of (+)-sambutoxin, which was completed in a 26 step LLS and 2.6% overall yield. Furthermore, the divergent nature of our strategy also facilitated the analogous total synthesis of (−)-sambutoxin A in a 12 step LLS and 4.7% overall yield using the same condensation strategy with lactol (−)-15 and simply changing the 4H2P nucleophile (22).

**Fig. 3:**
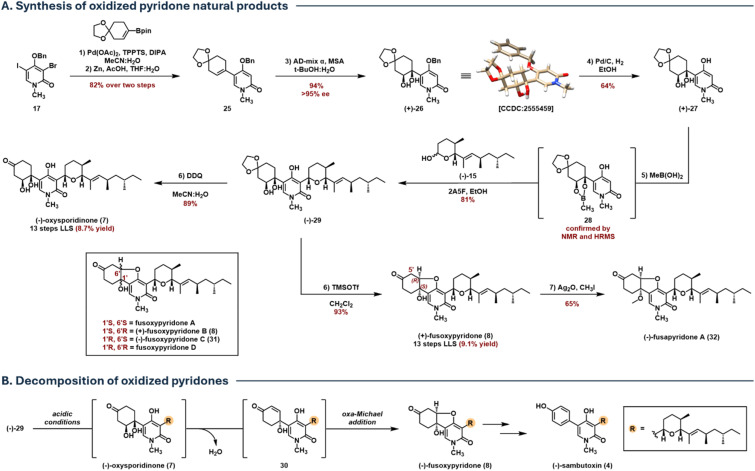
Total synthesis of (−)-oxysporidinone (7) and proposed structural reassignment of fusoxypyridone-type natural products (A) and decomposition pathway observed (B).

Having optimized and validated our synthetic strategy on the sambutoxin-type scaffolds, we next shifted our focus towards accessing the more complex natural products (−)-oxysporidinone and (−)-fusoxypyridone (Fig. 3A). We were able to cleanly translate our regioselective Suzuki–Miyaura coupling strategy to vinylboronates, enabling access to the desired bromopyridone from the linchpin common intermediate 17. Unfortunately, direct Sharpless dihydroxylation of this bromopyridone proceeded with poor enantioselectivity (Fig. S3).^30^ To our surprise, we found that subjecting the debrominated pyridone 25, accessible *via* zinc-mediated reduction, to the same dihydroxylation conditions produced near-enantiopure (>95% ee) pyridone diol (+)-26 in excellent yield, as confirmed by X-ray crystallography (CCDC: 2555459). We hypothesize that this dramatic change in enantioselectivity arises from improvements in substrate-ligand π-stacking interactions in the absence of the C3-bromide (Fig. S4). As in our other substrates, hydrogenolysis towards (+)-27 proceeded smoothly, providing the key 4H2P scaffold needed to access this next class of natural products.

Our initial attempts at translating our general condensation conditions to this new diol system only gave trace conversion to the desired product. We hypothesized that the free diol interfered with the productivity of the condensation; thus, we set out to modulate this reactivity *via* diol protection. Initial attempts at acetonide protection of (+)-26 were similarly unsuccessful, as we found typical conditions mediated by Lewis or Brønsted acid resulted in either protecting group transposition to the ketone acetonide or dehydration to various decomposition products (Table S5). In search of orthogonal protection conditions to avoid this unproductive dehydration, we took inspiration from carbohydrate chemistry and sought to protect the diol in the form of a transient boronate ester.^31^ Although our initial attempt at this strategy with phenylboronic acid failed to produce the expected boronate, likely due to sterics, we found that treatment of (+)-27 with methylboronic acid gave good conversion to the corresponding boronate 28, as confirmed by HRMS and ^11^B-NMR. Moreover, subjection of 28 to our standard condensation conditions with lactol (−)-15 gave the desired product (−)-29 in high yield after mild acidic workup and purification. We further optimized this workflow to a one-pot transformation by simply adding methylboronic acid to our standard condensation conditions, taking full advantage of this traceless protection strategy.

With pyridone diol (−)-29 in hand, only ketal deprotection remained to access (−)-oxysporidinone (7). Much like our earlier attempts at acetonide protection, however, this substrate remained prone to dehydration under acidic conditions. Interestingly, these dehydration intermediates closely mirrored the intermediates reported in the biosynthesis of (−)-oxysporidinone, albeit in the reverse direction.^32^ Further confirmation of this bioinspired approach was evidenced by the formation of the fusoxypyridone-type [6-5-6] ring system, as well as complete dehydration to the sambutoxin-type phenol, under the conditions explored (Fig. 3B). With these findings in mind, we revisited conditions we had previously investigated for acetonide protection and serendipitously found that treatment of pyridone diol (−)-29 with TMSOTf at 0 °C for 90 minutes preferentially gave near-quantitative conversion to the fusoxypyridone scaffold (8), presumably through controlled formation of enone 30 followed by intramolecular oxa-Michael addition.

When gathering full characterization data for the fusoxypyridone material, we were surprised to find that our synthetic sample showed an optical rotation opposite to that previously described in the literature, despite closely matching NMR assignments. A closer look at the two available isolation reports revealed a major inconsistency between them regarding the proton chemical shifts at C-5′, suggesting that the original isolation report may have misassigned the proposed structure.^10,13^ Subsequent correspondence with the Yu group (authors of the most recent isolation report) further supported this, as their optical rotation assignments had been assumed from the original report and not recalculated. Considering these findings, we initially posited that the compound described in the original isolation report may instead be the 1′,6′-diepimer (−)-31, accessible from the proposed prochiral biosynthetic precursor, dienone 6 (Fig. S6). Although we were able to adapt our synthetic strategy towards (−)-31, this compound did not align with any NMR assignments described in the literature. It remains likely that the original isolate is a closely related, hitherto unreported, *trans*-diastereomer of (+)-fusoxypyridone (8) based on the excellent agreement in chemical shift assignment of the rest of the molecule. We posit that an enzymatic desymmetrization of dienone 6 provides privileged access to this *trans*-junction, as synthetic efforts towards accessing these *trans*-diastereomers were unsuccessful. We propose that the natural product formerly named “fusoxypyridone” be expanded to accommodate all four diastereomers. To this end, we suggest that the original compound isolated by Gunatilaka *et al.* be renamed (−)-fusoxypyridone A, with our related diastereomers (+)-8 and (−)-31 similarly joining the family as fusoxypyridones B and C, respectively.^13^ With these revised structural assignments, subsequent methylation of (+)-fusoxypyridone B (8) at the tertiary alcohol provided the related isolate (−)-fusapyridone A (32).^33^ Observation of NOE correlations between the newly installed 1′-OCH_3_ and the 6′-H provided further support for the *syn* stereochemistry at the bridge junction.

Despite our success in controlling dehydration towards the fusoxypyridone scaffold, we were frustrated to find that none of our optimization attempts were fruitful in achieving selective ketal deprotection. It became clear that yet another orthogonal strategy would be necessary to perform the necessary deketalization while preserving the diol motif. We ultimately found that treatment of pyridone diol (−)-29 with DDQ in wet acetonitrile gave excellent conversion to (−)-oxysporidinone (7), completing our total synthesis efforts towards another subset of 4H2P natural products alongside (+)-fusoxypyridone (8), both in a 13 step LLS and in overall yields of 8.7% and 9.1%, respectively.^34–36^

## Conclusions

In summary, we have completed the first total syntheses of (−)-sambutoxin, (−)-sambutoxin A, (−)-oxysporidinone, (−)-fusapyridone A, and (+)-fusoxypyridone B in a convergent manner, allowing for the structural reassignment of the latter natural product through the related compound (−)-fusoxypyridone C. By leveraging a modular bioinspired condensation strategy that engages a functionalized lactol electrophile with a variety of 4H2P nucleophiles, we enabled access to complex chemical space with high efficiency. The 4H2P nucleophiles used in these condensations were prepared in a modular manner from a singular linchpin common intermediate, showcasing the power of the strategy towards accessing members of structurally related natural product families. Further optimization and adaptation of this general strategy towards additional members of the 4H2P natural product class and analogs therein are forthcoming.

## Author contributions

R. L. C. and K.-H. Sim contributed equally. The manuscript was written through contributions from all authors. All authors have given approval to the final version of the manuscript.

## Conflicts of interest

There are no conflicts to declare.

## Supplementary Material

SC-OLF-D6SC04572C-s001

SC-OLF-D6SC04572C-s002

## Data Availability

CCDC 2555459 [Compound (+)-26] and 2555677 [Compound 17] contain the supplementary crystallographic data for this paper.^37*a*,*b*^ All data supporting this work are provided in the supplementary information (SI), which includes experimental procedures, reaction optimization, compound characterization, and NMR spectra. Supplementary information: experimental procedures, reaction optimizations, compound characterization, and NMR spectra. See DOI: https://doi.org/10.1039/d6sc04572c.
