# Supplementary material for: The concise bioinspired total synthesis of the 4-hydroxy-2-pyridone family of natural products
Source: Chem Sci. 2026 Jul 27;17(36):17569–74. doi: 10.1039/d6sc04572c (PMC13431433; doi:10.1039/d6sc04572c)
Supplement: Supplementary file 1 [file SC-017-D6SC04572C-s001.pdf]

Supplementary Information (SI) for Chemical Science.
This journal is © The Royal Society of Chemistry 2026

Supporting Information

The concise bioinspired total synthesis of the 4-hydroxy-2-pyridone family of natural products

Ricardo L. Cruz[1], Kyu-Hyun Sim[1], Benjamin N. Chiok[1], and William M. Wuest*[1]

[1]Department of Chemistry, Emory University, Atlanta GA 30322, United States

Table of Contents

Section 1. Supplemental Figures ....S3 – S7

Section 2. Natural Product NMR Comparisons S8-S13

Section 3. Experimental Procedures and Compound Characterization S14-S40

Section 4. References S41

Section 5. ^{1}H and ^{13}C-NMR Spectra S42-S114

Section 1. Supplemental Figures.

Figure S1: Optimization of Knoevenagel condensation.

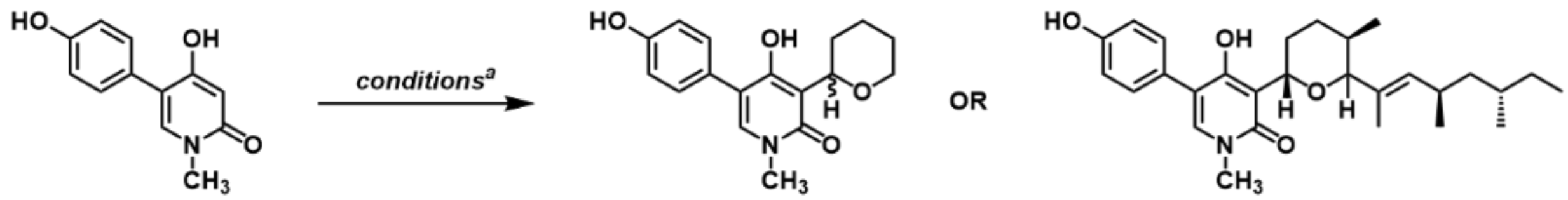

23

(-)-sambutoxin (4)

Entry	Base	Eq Base	Solvent	Temperature (ºC)	Lactol	Yield
1	K_2CO_3	3	acetone	25	S1	-
2	*t*-BuOK	3	1,4-dioxane	95	S1	-
3	DMAP	2.5	1,4-dioxane	105	S1	-
4	DMAP	0.8	EtOH	150	S1	58%
5	piperidine	2.5	1,4-dioxane	105	S1	-
6	piperidine	2.5	EtOH	80	S1	>95%
7	piperidine	2.5	EtOH	80	(-)-15	-
8	2A5F	2.5	EtOH	80	S1	53%
9	2A5F	2.5	EtOH	80	(-)-15	20%
10	2A5F	5	EtOH	80	(-)-15	60%

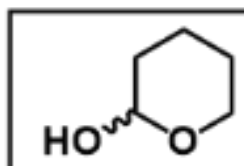

S1[b]

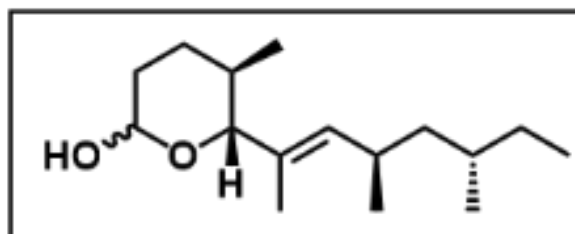

(-)-15

[a] Reactions were performed with 1.0 eq pyridone **23** and 1.3 eq lactol.

[b] Readily prepared from DIBAL-H reduction of commercial γ-valerolactone.

Figure S2: *In silico* thermodynamics of cis- or trans-tetrahydropyran formation.[a]

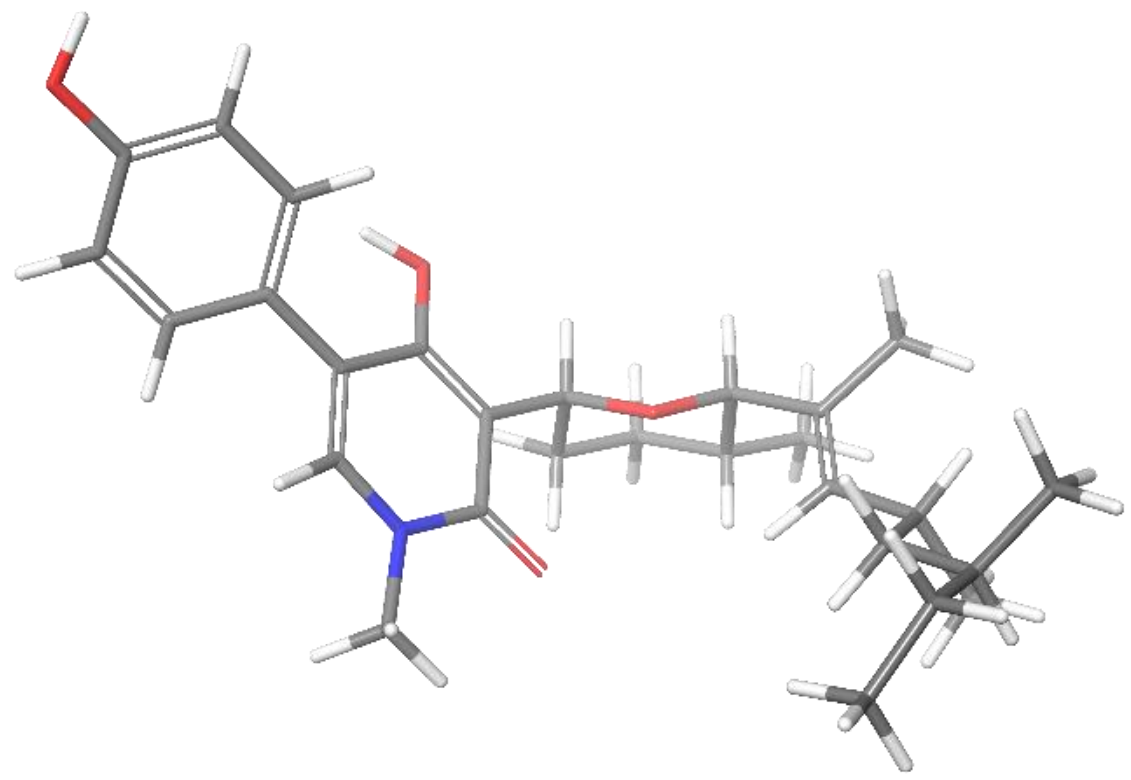

cis-sambutoxin

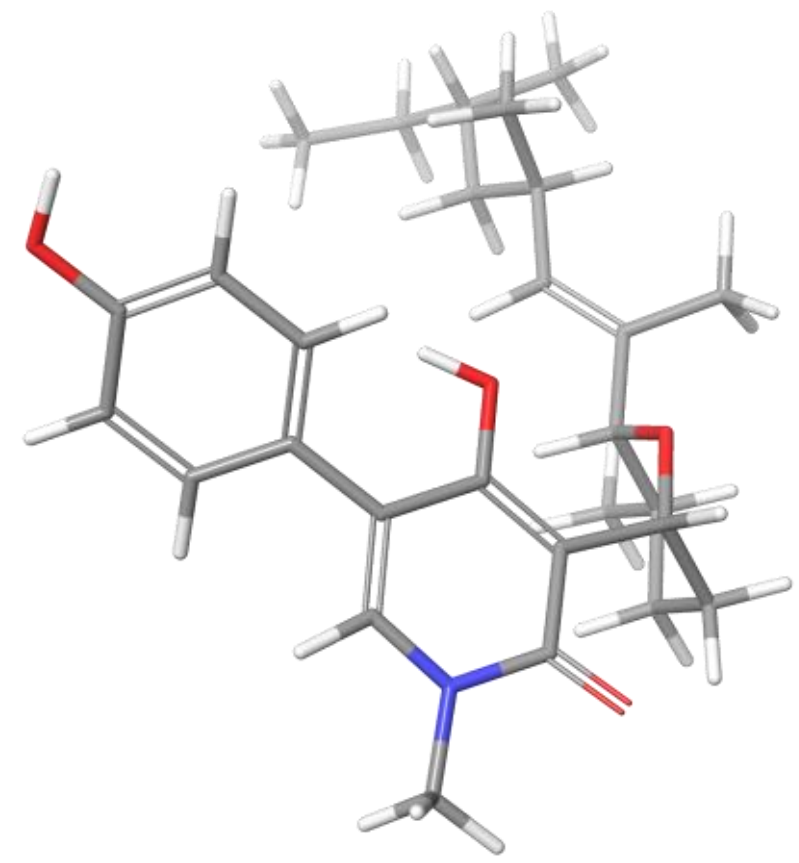

trans-sambutoxin

	cis-sambutoxin	*trans*-sambutoxin
**Relative energy (kcal/mol)**	**0.00**	**+ 1.25**

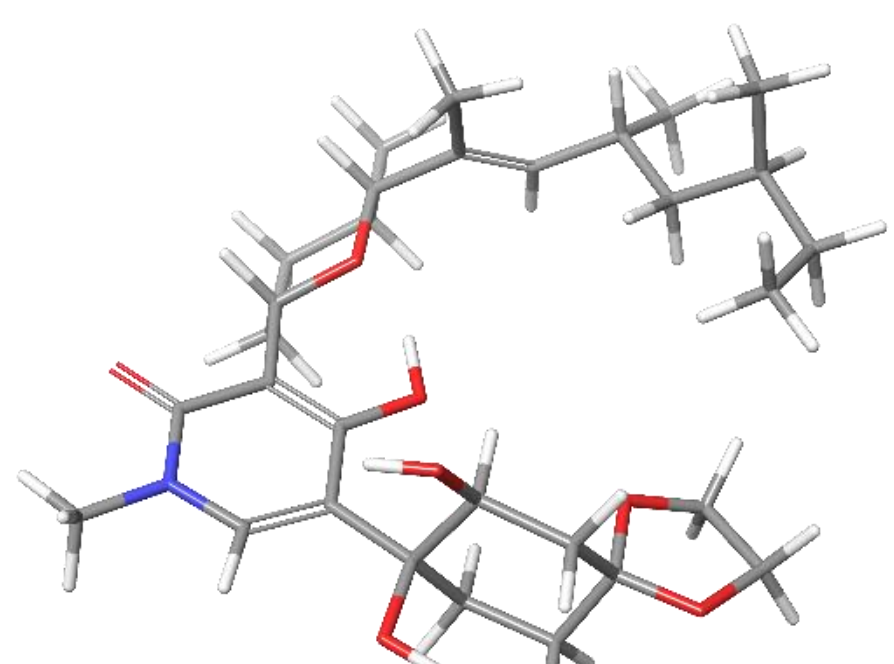

cis-oxysporidinone ketal

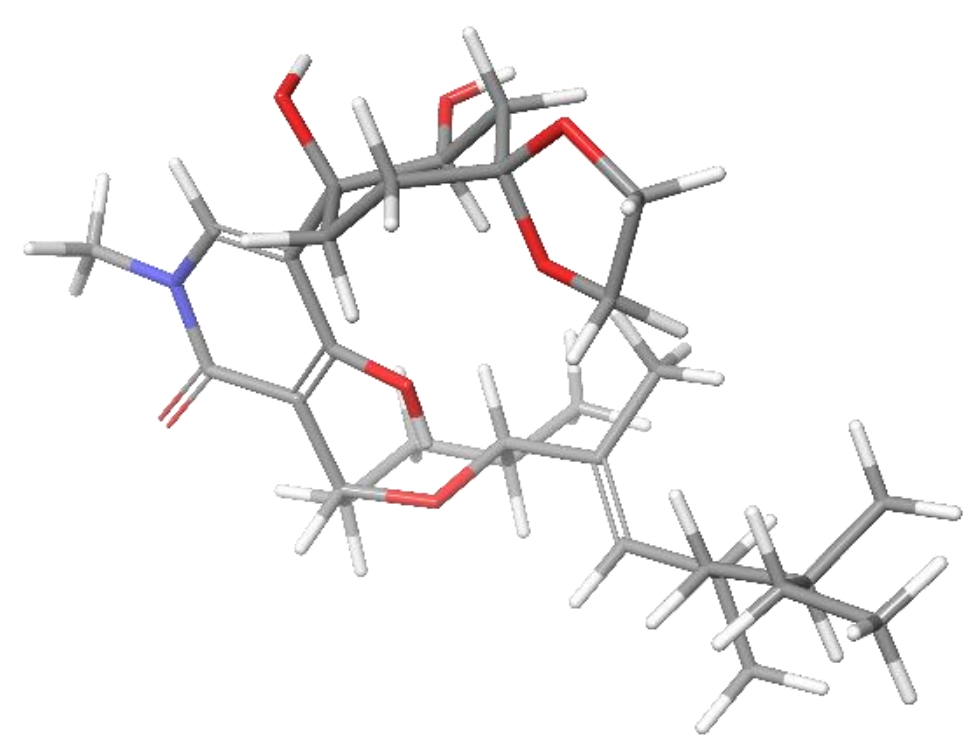

trans-oxysporidinone ketal

	cis-oxysporidinone ketal	*trans*-oxysporidinone ketal
**Relative energy (kcal/mol)**	**0.00**	**+ 4.38**

[a] Relative single-point energies for optimized structures calculated with Jaguar at the B3LYP-D3/6-31G** level.

Figure S3: Optimization of Sharpless dihydroxylation.

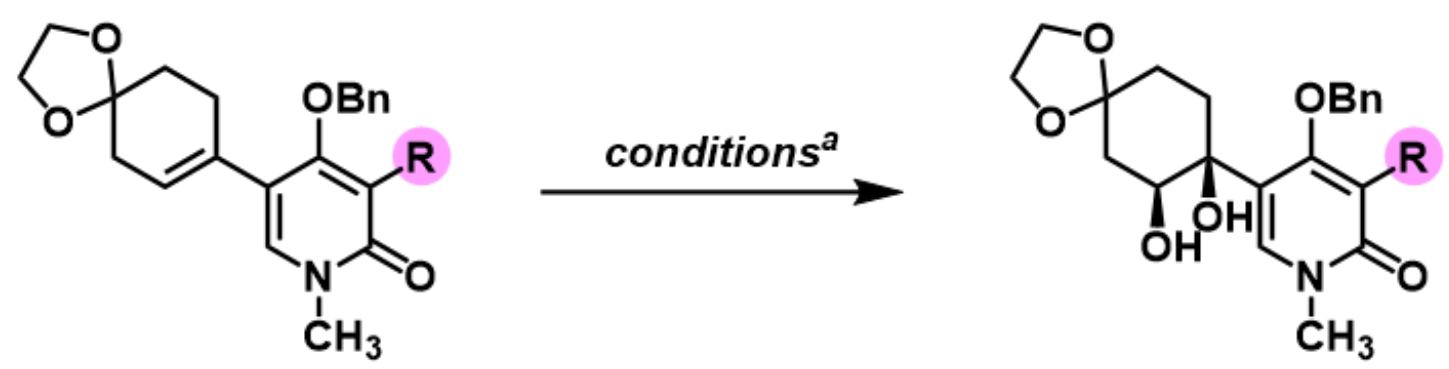

Entry	R	Oxidant	[Os] source	Additives	Yield (%)	%ee	Notes
1	Br	$K_3[Fe(CN)_6]$	AD-mix-alpha (Sigma)[b]	MSA[c], K_2CO_3	N.R.	-	-
2	Br	$K_3[Fe(CN)_6]$	$K_2OsO_4 \cdot 2H_2O$ (2.5 mol%)	MSA, K_2CO_3	80	75	7.5mol% $(DHQ)_2$PHAL
3	Br	NMO	$K_2OsO_4 \cdot 2H_2O$ (2.5 mol%)	-	73	27	7.5mol% $(DHQ)_2$PHAL
4	Br	$K_3[Fe(CN)_6]$	$K_2OsO_4 \cdot 2H_2O$ (2.5 mol%)	MSA, K_2CO_3	83	51	5.0mol% $(DHQ)_2$PHAL
5	H	$K_3[Fe(CN)_6]$	AD-mix-alpha (Sigma)	MSA	65	83	72h reaction time
6	H	$K_3[Fe(CN)_6]$	$K_2OsO_4 \cdot 2H_2O$ (2.5 mol%)	MSA, K_2CO_3	94	>95	5.0mol% (DHQ)2PHAL

[a] Reactions run in a 1:1 mixture of *t*-BuOH:H_2O at 25°C

[b] Commercial AD-mix surprisingly gave poor results compared to reconstitution with individual reagents

[c] MSA = methanesulfonamide

Figure S4: Proposed rationale for substrate-dependent enantioselectivity.

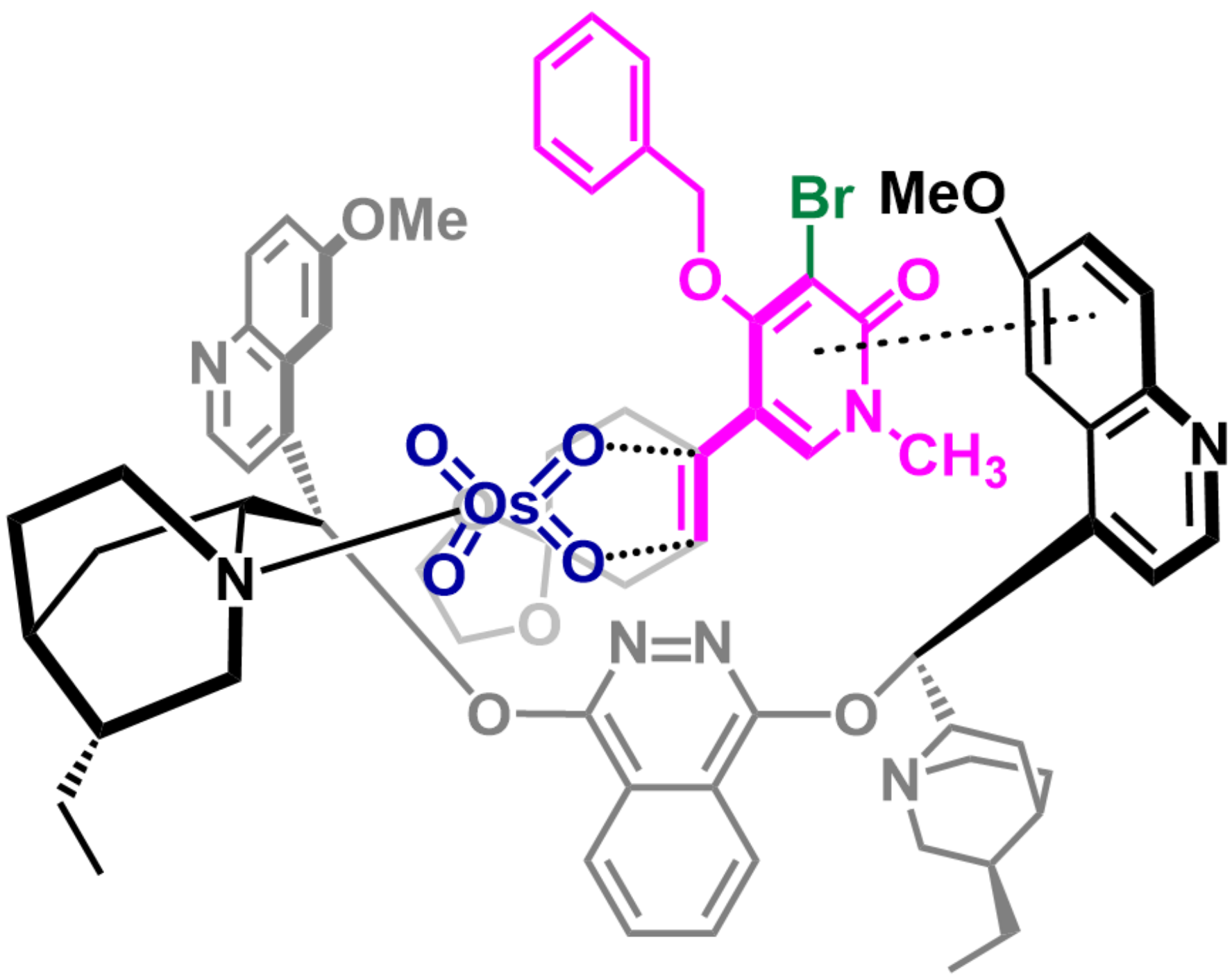

Substrate-ligand π-stacking interactions are well-documented for styrene-type substrates.[1] We posit that these π-stacking interactions are more favorable in the absence of the C3-bromide, translating to exceptionally high enantioselectivity in dihydroxylation. Alternatively, the sterics of the C3-bromide could force conformational changes at the neighboring benzyl ether, introducing steric clashes with the ligand and subsequently deteriorating enantioselectivity.

Figure S5: Attempts at acetonide protection of pyridone diol (+)-26.

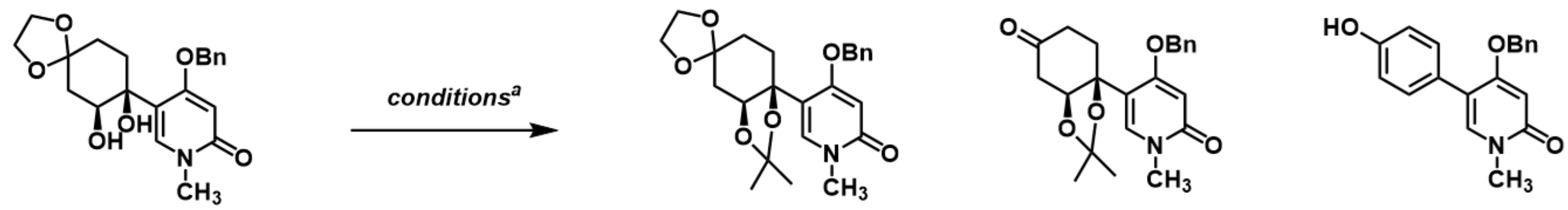

(+)-26 P S2 S3

Entry	Bronsted or Lewis acid	Eq Acid	Electrophile	Temperature (°C)	Result
1	$ZrCl_4$	0.1	DMP	25	SM
2	$Ga(OTf)_3$	0.2	acetone	25	SM
3	$Sc(OTf)_3$	0.2	acetone	25	SM + S2 + S3
4	$Yb(OTf)_3$	0.2	DMP	25	SM
5	*p*-TSA	0.05	acetone	25	SM
6	*p*-TSA	0.05	acetone	60	P + S2 + S3
7	TMSOTf	1.2	DMP	0	SM
8	TMSOTf	1.2	acetone	0	S2
9	TMSOTf	1.2	acetone	-78	SM

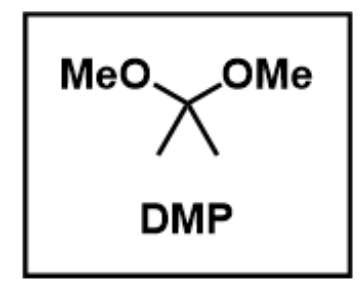

[a] Reactions run in anhydrous dichloromethane under argon atmosphere.

[b] Conversion of starting material was monitored by LCMS and isolates identified by crude ^{1}H-NMR

Figure S6: Studies towards the true structure of (-)-fusoxypyridone.

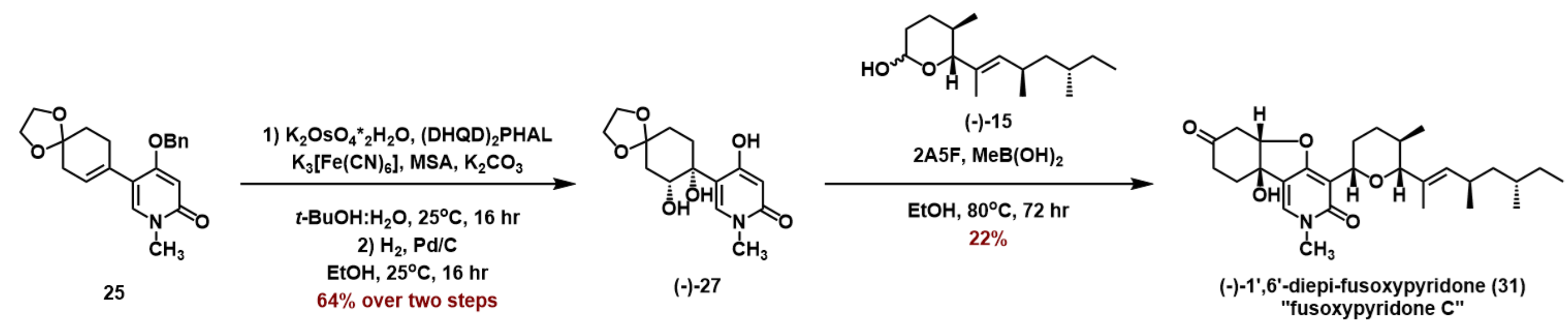

Interestingly, standard Knoevenagel condensations including methylboronic acid gave predominantly fusoxypyridone after purification rather than the expected ketal product.

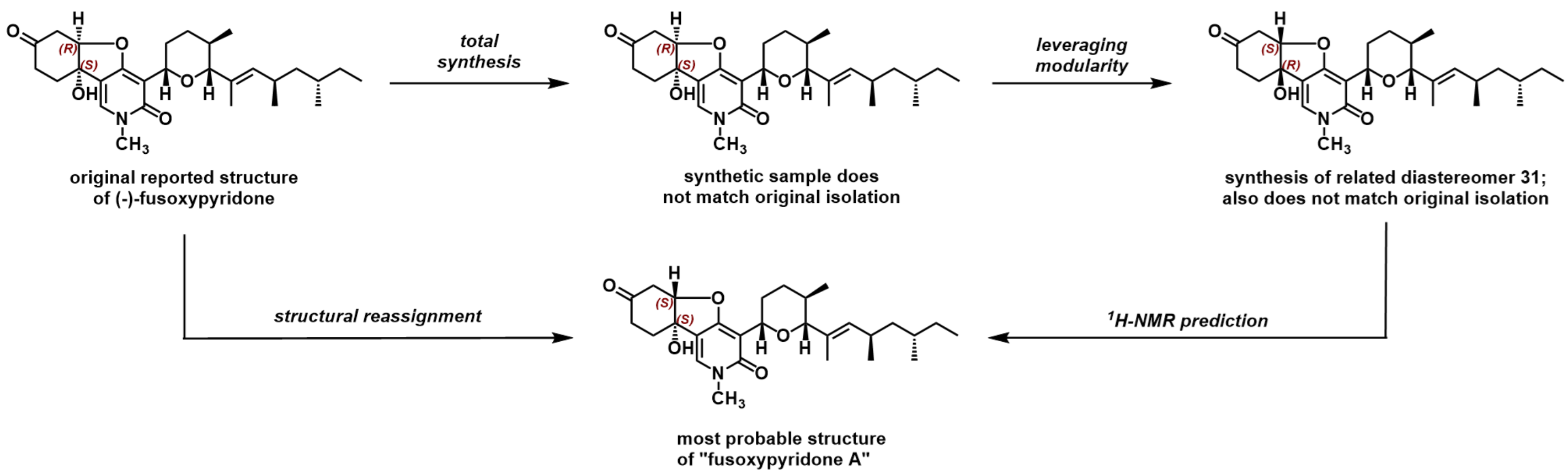

^{1}H- and ^{13}C-NMR characterization of the 1',6'-diepimer still failed to align with the assignments reported in the original isolation (Gunatilaka *et al*, *J. Nat. Prod*. **2007**). Based on the major discrepancies being associated with the two diastereotopic protons at C-5', we hypothesize the original isolate may in fact be the 6'-epimer. Although theoretically accessible from the prochiral biosynthetic precursor **6**, attempts at forging the *anti*-[6,5,6] ring junction synthetically have proven unsuccessful. Nevertheless, our findings support the existence of additional fusoxypyridone diastereomers – expansion of this group to fusoxypyridones A-D would provide a more accurate representation of the natural products isolated thus far in the literature, as well as those undiscovered but theoretically accessible from dienone **6**.

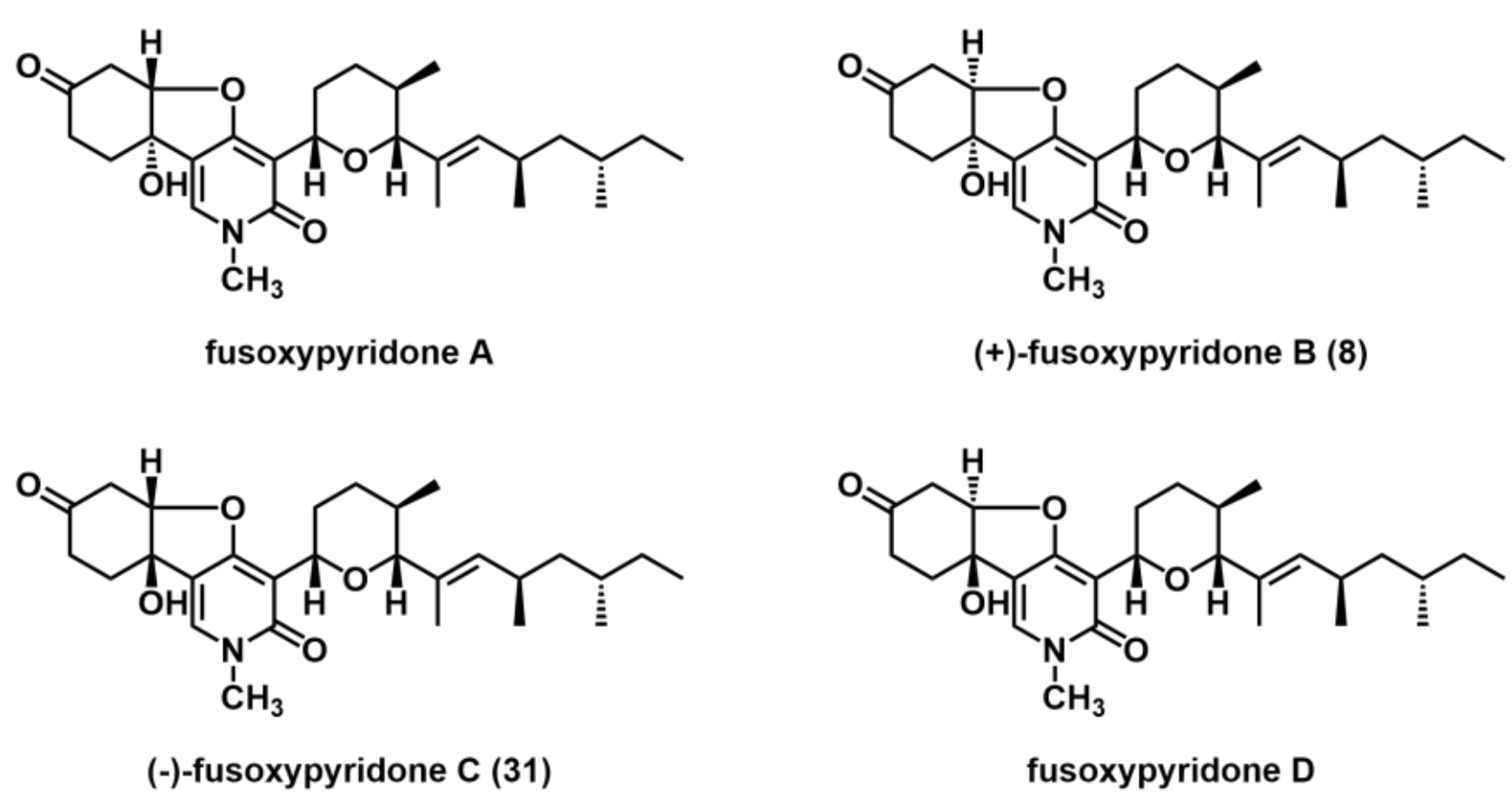

Section 2. Natural Product NMR Comparisons

Figure S7. Comparison of isolated and synthetic (-)-sambutoxin A.[2]

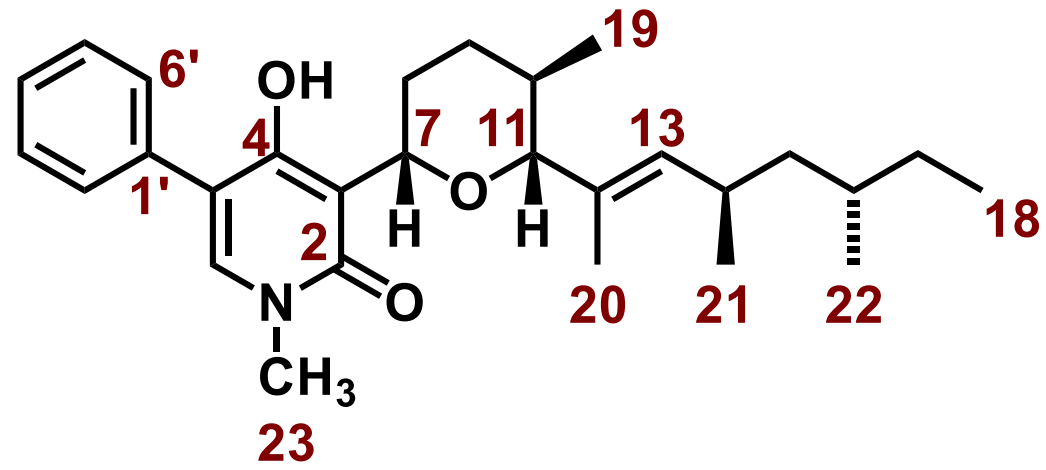

(-)-sambutoxin A (3)

^{1}H / ^{13}C assignments (800MHz, $CDCl_3$)

Position	Literature (1H)	Experimental (1H)	Δδ	Literature (13C)	Experimental (13C)	Δδ
2				161.5	161.4	0.1
3				110.4	110.3	0.1
4				162	161.8	0.2
5				115.2	115.1	0.1
6	7.14	7.15	0.01	136.4	136.3	0.1
7	5.02	5.03	0.01	78	77.9	0.1
8a	1.68	1.69	0.01	30.8	30.7	0.1
8b	2.09	2.1	0.01			
9a	1.44	1.44	0	32.3	32.2	0.1
9b	1.91	1.92	0.01			
10	1.65	1.64	0.01	32.5	32.4	0.1
11	3.53	3.53	0	92.7	92.6	0.1
12				130.4	130.3	0.1
13	5.18	5.2	0.02	138.1	138	0.1
14	2.46	2.47	0.01	29.7	29.6	0.1
15a	1.04	1.04	0	44.8	44.7	0.1
15b	1.19	1.2	0.01			
16	1.3	1.29	0.01	32.1	32	0.1
17a	1.04	1.04	0	29	28.9	0.1
17b	1.34	1.35	0.01			
18	0.83	0.83	0	11.3	11.2	0.1
19	0.74	0.75	0.01	17.8	17.7	0.1
20	1.6	1.62	0.02	11.7	11.6	0.1
21	0.9	0.9	0	20.8	20.7	0.1
22	0.82	0.82	0	19.7	19.6	0.1
23	3.5	3.51	0.01	37.2	37	0.2
1'				134.2	134.1	0.1
2'	7.44	7.43	0.01	129.3	129.2	0.1
3'	7.4	7.39	0.01	128.4	128.3	0.1
4'	7.32	7.32	0	127.5	127.4	0.1
5'	7.4	7.39	0.01	128.4	128.3	0.1
6'	7.44	7.43	0.01	129.3	129.2	0.1
4-OH	9.99	9.98	0.01			

Figure S8. Comparison of isolated and synthetic (-)-sambutoxin.[3]

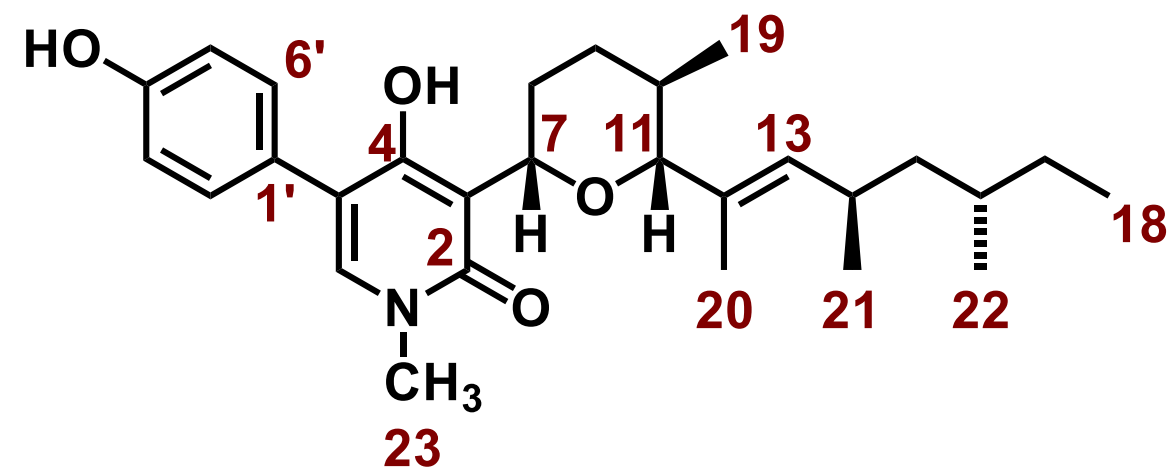

(-)-sambutoxin (4)

^{1}H / ^{13}C assignments (600MHz, $CDCl_3$)

Position	Literature (1H)	Experimental (1H)	Δδ	Literature (13C)	Experimental (13C)	Δδ
2				161.4	161.4	0
3				110.4	110.4	0
4				162.4	162.4	0
5				115.6	115.5	0.1
6	7.12	7.12	0	135.9	135.9	0
7	5.04	5.04	0	77.8	77.8	0
8a	1.68	1.68	0	30.7	30.7	0
8b	2.08	2.09	0.01			
9a	1.43	1.42	0.01	32.1	32.1	0
9b	1.9	1.9	0			
10	1.68	1.68	0	32.3	32.3	0
11	3.51	3.51	0	92.5	92.6	0.1
12				130.1	130.2	0.1
13	5.19	5.19	0	138	138	0
14	2.46	2.46	0	29.6	29.6	0
15a	1.04	1.04	0	44.7	44.7	0
15b	1.2	1.2	0			
16	1.3	1.31	0.01	31.9	32	0.1
17a	1.04	1.04	0	28.9	28.9	0
17b	1.35	1.35	0			
18	0.83	0.83	0	11.2	11.2	0
19	0.74	0.74	0	17.6	17.7	0.1
20	1.62	1.62	0	11.6	11.6	0
21	0.9	0.9	0	20.6	20.7	0.1
22	0.84	0.84	0	19.5	19.6	0.1
23	3.52	3.52	0	37.3	37.3	0
1'				125.2	125.6	0.4
2'	7.28	7.28	0	130.3	130.4	0.1
3'	6.91	6.91	0	115.4	115.4	0
4'				156.3	156.1	0.2
5'	6.91	6.91	0	115.4	115.4	0
6'	7.28	7.28	0	130.3	130.4	0.1
4-OH	10.03	10.03	0			

Figure S9. Comparison of isolated and synthetic (-)-oxysporidinone.[4]

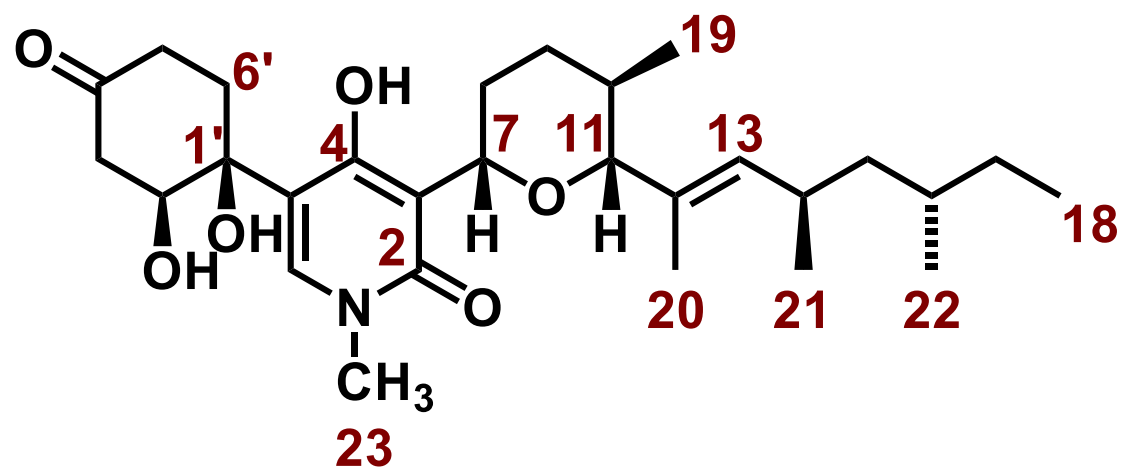

(-)-oxysporidinone (7)

^{1}H / ^{13}C assignments (800MHz, $CDCl_3$)

Position	Literature (1H)	Experimental (1H)	Δδ	Literature (13C)	Experimental (13C)	Δδ
2				161.4	161.4	0
3				113.9	113.8	0.1
4-OH	10.3	10.25	0.05	161.3	161.3	0
5				110.8	110.8	0
6	7.46	7.44	0.02	136.2	136.2	0
7	4.92	4.97	0.05	78	78	0
8a	1.51	1.57	0.06	30.7	30.6	0.1
8b	2	1.99	0.01			
9a	1.39	1.42	0.03	32.1	32	0.1
9b	1.87	1.91	0.04			
10	1.67	1.7	0.03	32.4	32.4	0
11	3.49	3.51	0.02	92.5	92.6	0.1
12				130	130.1	0.1
13	5.2	5.22	0.02	138.2	138.2	0
14	2.49	2.5	0.01	29.6	29.7	0.1
15a	1.04	1.06	0.02	44.7	44.7	0
15b	1.22	1.22	0			
16	1.29	1.23	0.06	32	32	0
17a	1.33	1.3	0.03	28.9	28.9	0
17b	1.04	1.06	0.02			
18	0.83	0.84	0.01	11.2	11.2	0
19	0.74	0.76	0.02	17.6	17.6	0
20	1.63	1.63	0	11.8	11.8	0
21	0.91	0.93	0.02	20.7	20.7	0
22	0.83	0.85	0.02	19.6	19.6	0
23	3.44	3.49	0.05	37.3	37.3	0
1'				73.7	73.7	0
2a'	2.36	2.32	0.04	32.1	32	0.1
2b'	2.05	2.06	0.01			
3a'	2.78	2.79	0.01	36.4	36.4	0
3b'	2.23	2.25	0.02			
4'				208.8	208.8	0
5a'	2.75	2.74	0.01	45.5	45.3	0.2
5b'	2.65	2.65	0			
6'	4.65	4.67	0.02	70.7	70.3	0.4

Figure S10. Comparison of isolated and synthetic (+)-fusoxypyridone B in CD_3OD.[4,5]

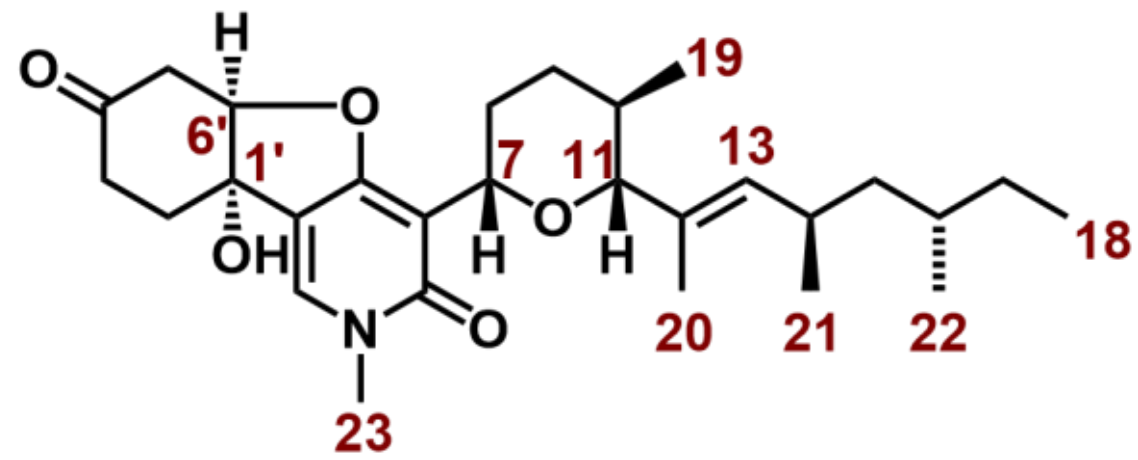

(+)-fusoxypyridone B (8)

^{1}H / ^{13}C assignments (400MHz, CD_3OD)*

Position	Literature (1H) Gunatilaka *et al*	Literature (1H) Yu *et al*	Experimental (1H) This work	Δδ (based on Yu *et al*)	Literature (13C) Gunatilaka *et al*	Literature (13C) Yu *et al*	Experimental (13C) This work	Δδ (based on Yu *et al*)
2					165.3	165.2	165.3	0.1
3					109.5	109.3	109	0.3
4					168.5	168.4	168.3	0.1
5					119.1	119	118.5	0.5
6	7.68	7.76	7.66	0.1	135.8	135.8	136	0.2
7	4.76	4.76	4.72	0.04	73.7	73.5	73.9	0.4
8a	2.18	2.18	2.13	0.05	30.3	30.1	30.2	0.1
8b	1.53	1.54	1.57	0.03				
9a	1.9	1.9	1.9	0	34.1	33.9	33.8	0.1
9b	1.31	1.31	1.31	0				
10	1.65	1.64	1.63	0.01	33.1	32.9	32.9	0
11	3.37	3.37	3.37	0	93.2	93	93.3	0.3
12					133.9	133.8	133.6	0.2
13	5.11	5.11	5.12	0.01	137.4	137.2	137.7	0.5
14	2.51	2.51	2.48	0.03	30.9	30.7	30.7	0
15a	1.22	1.22	1.19	0.03	46.4	46.2	46.2	0
15b	1.08	1.06	1.03	0.03				
16	1.36	1.34	1.34	0	33.4	33.3	33.3	0
17a	1.43	1.42	1.4	0.02	30.1	29.9	29.8	0.1
17b	1.08	1.06	1.03	0.03				
18	0.85	0.86	0.82	0.04	11.7	11.5	11.6	0.1
19	0.73	0.73	0.7	0.03	18.4	18.3	18.2	0.1
20	1.57	1.58	1.59	0.01	11.7	11.6	11.6	0
21	0.9	0.9	0.87	0.03	21.3	21.2	21.2	0
22	0.85	0.86	0.82	0.04	20.2	20.1	20.1	0
23	3.51	3.5	3.47	0.03	38.6	38.5	38.5	0
1'					77.2	77	77	0
2a'	2.33	2.34	2.34	0	34.4	34.2	34	0.2
2b'	2.25	2.24	2.21	0.03				
3a'	2,38	2.37	2.39	0.02	36	35.8	35.9	0.1
3b'	2.03	2.02	2.04	0.02				
4'					210.3	210.4	210.4	0
5a'	3.48	3.03	3.06	0.03	49.4	49.2	49.1	0.1
5b'	3.37	2.79	2.73	0.06				
6'	4.92	4.93	4.93	0	92	91.9	92.4	0.5

*Our synthetic sample closely aligns with the most recent isolation reported (Yu *et al*. *ACIE* **2023**) but has some distinction at C5' when compared to the original isolation report (Gunatilaka *et. al J. Nat. Prod.* **2007**). These major inconsistencies (Δδ > 0.4 ppm) were observed not only when compared against our synthetic sample, but also the 2023 report (highlighted in orange), suggesting the 2007 isolation may have been structurally misassigned.

Figure S11. Comparison of isolated and synthetic (+)-fusoxypyridone B in $CDCl_3$.[4]

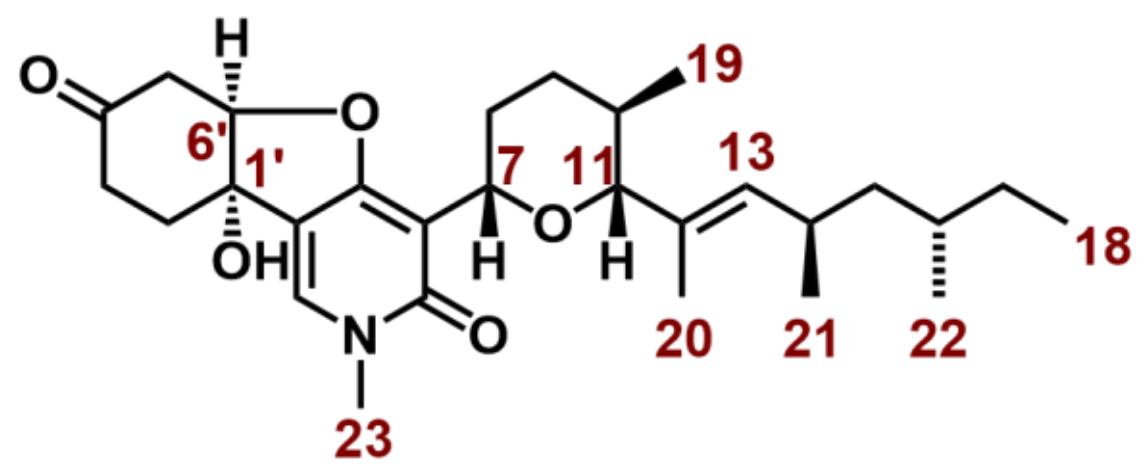

(+)-fusoxypyridone B (8)

^{1}H / ^{13}C assignments (400MHz, $CDCl_3$)

Position	Literature (1H) Yu *et al*	Experimental (1H) This work	Δδ	Literature (13C) Yu *et al*	Experimental (13C) This work	Δδ
2				163.2	163.2	0
3				108.9	109.1	0.2
4				166	165.7	0.3
5				116.5	115.7	0.8
6	7.25	7.25	0	133.6	133.3	0.3
7	4.63	4.75	0.12	72	72.3	0.3
8a	2.1	1.98	0.12	29	29	0
8b	1.44	1.48	0.04			
9a	1.85	1.82	0.03	32.9	32.9	0
9b	1.35	1.34	0.01			
10	1.65	1.62	0.03	31.7	31.6	0.1
11	3.36	3.38	0.02	91.5	91.6	0.1
12				132.7	132.2	0.5
13	5.13	5.13	0	136.3	136.7	0.4
14	2.47	2.44	0.03	29.1	29.3	0.2
15a	1.04	1.01	0.03	45	44.8	0.2
15b	1.18	1.15	0.03			
16	1.31	1.31	0	32	32	0
17a	1.26	1.23	0.03	29.6	29.5	0.1
17b	1.04	1.01	0.03			
18	0.83	0.82	0.01	11.4	11.3	0.1
19	0.7	0.68	0.02	17.9	17.8	0.1
20	1.6	1.62	0.02	11.4	11.3	0.1
21	0.87	0.84	0.03	20.9	20.7	0.2
22	0.83	0.82	0.01	19.7	19.5	0.2
23	3.5	3.41	0.09	38	38.1	0.1
1'				76.4	76.4	0
2a'	2.21	2.2	0.01	33.4	32.8	0.6
2b'	2.33	2.34	0.01			
3a'	2.41	2.42	0.01	35	35	0
3b'	2.1	1.98	0.12			
4'				207.6	207.4	0.2
5a'	2.8	2.83	0.03	42	42	0
5b'	2.92	2.92	0			
6'	4.9	4.92	0.02	90.1	90.4	0.3

Figure S12. Comparison of isolated and synthetic (-)-fusapyridone A in $CDCl_3$.[6]

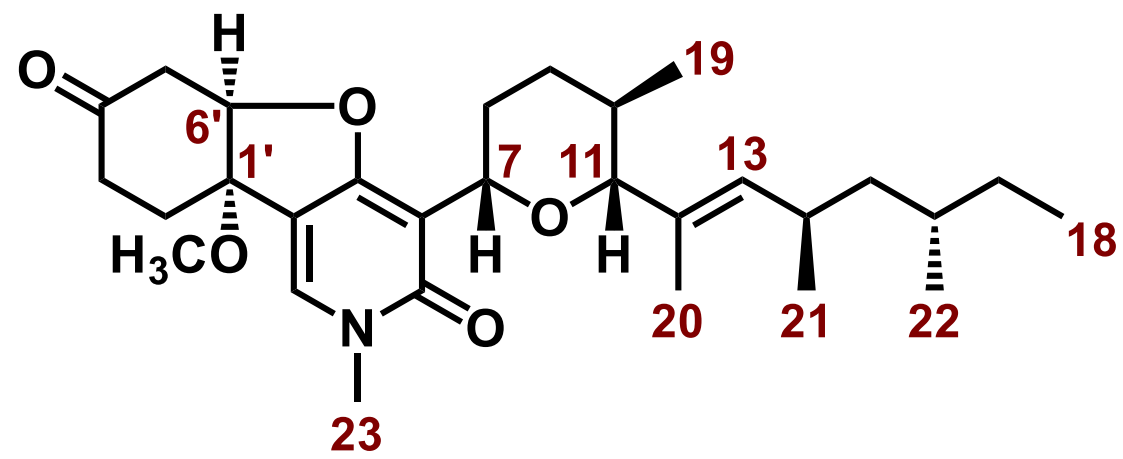

(-)-fusapyridone A (32)

^1H / ^{13}C assignments (400 MHz, $CDCl_3$)

Position	Literature (1H)	Experimental (1H)	Δδ	Literature (13C)	Experimental (13C)	Δδ
2				163.2	163.1	0.1
3				109.9	109.7	0.2
4				166.3	166.3	0.0
5				111.3	110.9	0.4
6	7.20	7.19	0.01	133.1	133.2	0.1
7	4.77	4.78	0.01	71.7	72.1	0.4
8a	1.57	1.55	0.02	29.2	29.2	0.0
8b	2.06	2.06	0.00			
9a	1.31	1.33	0.02	32.8	32.9	0.1
9b	1.85	1.85	0.00			
10	1.58	1.55	0.03	31.7	31.8	0.1
11	3.36	3.39	0.03	91.1	91.3	0.2
12				132.4	132.3	0.1
13	5.07	5.12	0.05	135.8	136.1	0.3
14	2.44	2.47	0.03	29.4	29.2	0.2
15a	1.03	1.03	0.00	44.9	44.9	0.0
15b	1.16	1.17	0.01			
16	1.31	1.33	0.02	31.8	31.8	0.0
17	1.31	1.33	0.02	29.7	29.4	0.3
18	0.84	0.85	0.01	11.2	11.3	0.1
19	0.69	0.70	0.01	17.9	17.9	0.0
20	1.58	1.63	0.05	11.0	11.2	0.2
21	0.87	0.89	0.02	20.6	20.6	0.0
22	0.82	0.83	0.01	19.5	19.5	0.0
23	3.48	3.48	0.00	38.2	38.3	0.1
1'				82.1	82.2	0.1
2'	4.98	5.03	0.05	84.3	84.1	0.2
3a'	2.77	2.86	0.09	42.4	42.2	0.2
3b'	2.86	2.86	0.00			
4'				207.1	207.5	0.4
5a'	2.36	2.4	0.04	35.0	34.9	0.1
5b'	2.14	2.14	0.00			
6'	2.36	2.37	0.01	31.9	32.3	0.4
1'-OMe	3.19	3.21	0.02	50.8	51.2	0.4

Section 3. Experimental Procedures and Compound Characterization.

Non-aqueous reactions were carried out under an atmosphere of argon in flame-dried glassware. Tetrahydrofuran (THF), dichloromethane (DCM), diethyl ether (Et_2O), toluene (PhMe), and N,N-dimethylformamide (DMF) were dried by passage through alumina. All other commercial reagents and anhydrous solvents were used as received (from Sigma Aldrich, Oakwood Chemical, Combi Blocks, Alfa Aesar, Fisher Scientific, TCI America, or AK Scientific) without further purification. Brine refers to a saturated aqueous solution of sodium chloride. Reactions were monitored by analytical thin layer chromatography (TLC) using EMD Millipore silica gel 60 F254 precoated plates and visualized using UV and/or ninhydrin, vanillin, *p*-anisaldehyde, cerium ammonium nitrate (CAN), or $KMnO_4$ stains. Flash chromatography employing Siliaflash silica gel (40-63 μm) was performed on a Biotage Isolera One instrument with a linear normal-phase gradient.

Nuclear magnetic resonance (1H, 13C NMR) spectra were recorded using the following spectrometers: Bruker Avance NEO800 (800 MHz), Varian INOVA600 (600 MHz), Bruker Ascend (600 MHz), Varian INOVA500 (500 MHz), Varian INOVA400 (400 MHz), Bruker Avance NEO400 (400 MHz), and Bruker Avance III (400 MHz). Chemical shifts are reported in parts per million (ppm) relative to tetramethylsilane and referenced to the residual solvent signal. Signal patterns are indicated as follows: s (singlet), d (doublet), t (triplet), q (quartet), m (multiplet), br (broad signal). Structural assignments were made with additional information from gCOSY, gHSQC, and gHMBC experiments. High resolution mass spectra (HRMS) were obtained using a Thermo Electron Corporation Finigan LTQFTMS at the Mass Spectrometry Facility, Emory University. High Pressure Liquid Chromatography (HPLC) was performed on an Agilent 1260 Infinity II series HPLC utilizing CHIRALPAK® IA, IB, IH, IJ, and IK 4.6 x 150 mm analytical columns. Optical rotations were measured on a PerkinElmer 341 polarimeter.

Ketone acetonide S2:

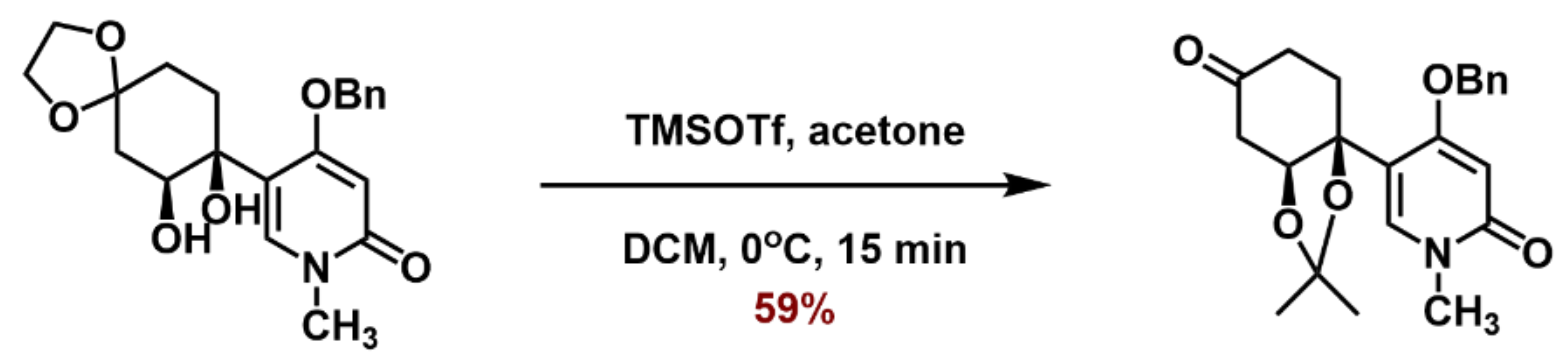

(+)-26 S2

To a flame-dried vial under argon atmosphere was added pyridone diol **(+)-26** (1.00 eq, 15.0 mg, 38.7 µmol) in anhydrous dichloromethane (1.00 mL). To this was added anhydrous acetone (7.00 eq, 19.9 µL, 271 µmol) prior to cooling the mixture to 0ºC, after which TMSOTf (1.24 eq, 8.67 µL, 48.0 µmol) was added. The resulting solution was left to stir at 0ºC for 15 minutes then quenched by addition of deionized water, extracting with additional dichloromethane. The combined organic layers were washed with saturated sodium bicarbonate and brine, then dried over sodium sulfate and concentrated *in vacuo* to give crude material. Purification by column chromatography (0-20% acetone in dichloromethane) afforded ketone acetonide **S2** as an amorphous white solid in 59% yield (8.7 mg, 23.0 µmol).

^{1}H NMR (400 MHz, $CDCl_3$) δ 7.56 (s, 1H), 7.39 (dp, *J* = 3.9, 1.8 Hz, 3H), 7.34 – 7.30 (m, 2H), 6.07 (s, 1H), 4.97 (d, *J* = 1.2 Hz, 2H), 4.57 – 4.51 (m, 1H), 3.52 (s, 3H), 2.45 (ddd, *J* = 19.2, 14.9, 4.5 Hz, 1H), 2.38 – 2.24 (m, 2H), 2.24 – 2.14 (m, 2H), 1.81 – 1.72 (m, 1H), 1.48 (s, 3H), 1.28 (s, 3H).

^{13}C NMR (101 MHz, $CDCl_3$) δ 209.68, 163.97, 163.91, 136.46, 133.86, 129.56, 129.15, 128.83, 117.09, 107.48, 98.14, 80.31, 78.59, 71.43, 41.88, 37.09, 34.25, 33.11, 26.22, 25.12.

α, β - unsaturated ester (-)-S4 :

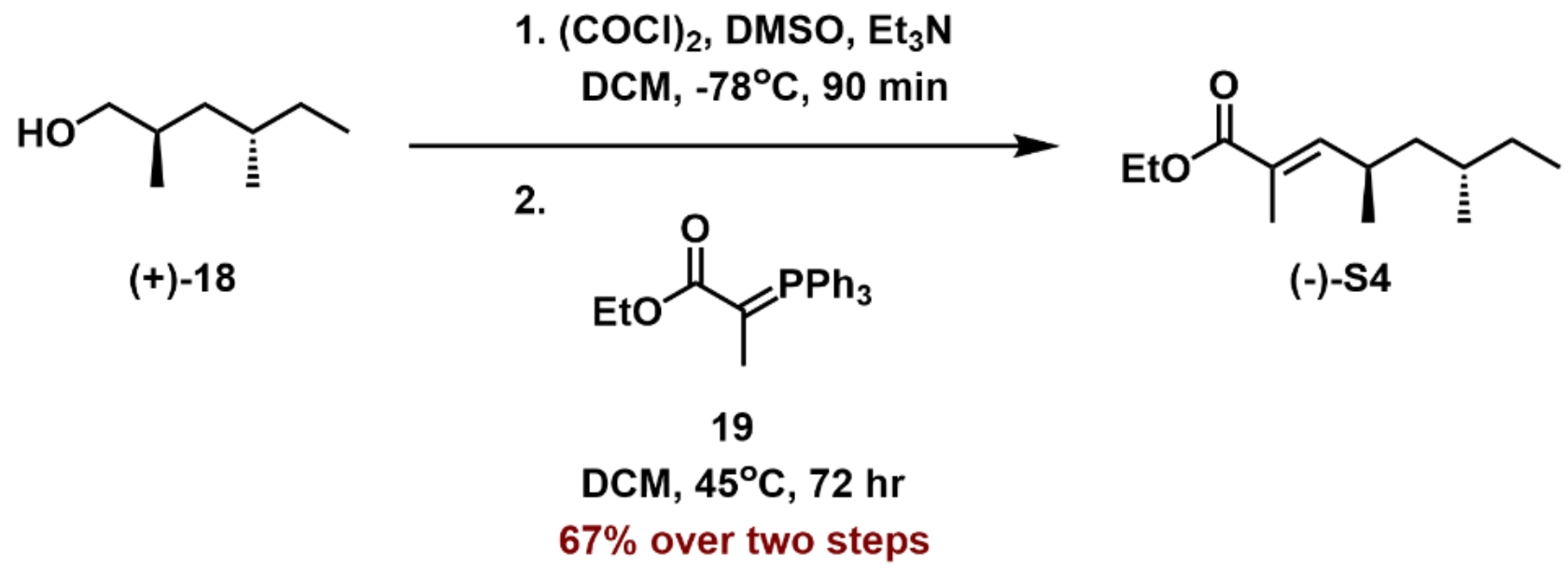

To a flame-dried flask under argon atmosphere was added oxalyl chloride (1.37 g, 930 µL, 10.8 mmol, 1.40 eq) in dichloromethane (15.5 mL) at −78 °C. Dimethylsulfoxide (1.37 mL, 19.31 mmol, 2.50 eq) was added dropwise, and the reaction mixture was stirred for 15 minutes. A solution of known alcohol (2*R*,4*S*)-2,4-dimethylhexan-1-ol **(+)-18**[7] (1.01 g, 7.73 mmol, 1.00 eq) in dichloromethane (15.5 mL) was added dropwise, and the reaction mixture was stirred for an additional 30 minutes at −78 °C. Triethylamine (5.38 mL, 38.6 mmol, 5.00 eq) was then added dropwise, and the reaction mixture was allowed to warm to 0 °C over 45 minutes. The reaction was quenched with saturated aqueous ammonium chloride, and the aqueous layer was extracted with dichloromethane. The combined organic layers were washed with brine twice then dried over anhydrous sodium sulfate, filtered, and concentrated *in vacuo* to afford crude aldehyde, which was used without further purification.

NOTE: Concentration *in vacuo* was performed at temperatures below 10ºC to minimize volatility.

This crude aldehyde was taken up in anhydrous dichloromethane (32.0 mL). Ethyl 2-(triphenylphosphoranylidene)propionate (**19**, 3.08 g, 8.50 mmol, 1.10 eq) was added, then the reaction mixture was left to stir for 72 hours at 45ºC. Additional ylide was added as needed until complete consumption of starting material was observed by TLC. The reaction was quenched with water, and the mixture was diluted with dichloromethane. The organic layer was separated, and the aqueous layer was extracted with dichloromethane. The combined organic layers were washed with brine, dried over anhydrous sodium sulfate and concentrated in vacuo to give crude material. Purification by column chromatography (0-10% ethyl acetate in hexanes) afforded ester **(-)-S4** as a clear oil in 67% yield over two steps (1.10 g, 5.18 mmol).

$\mathbf{R_f}$ = 0.6 (10% ethyl acetate in hexanes, visualized by $KMnO_4$)

^{1}H NMR (400 MHz, $CDCl_3$) δ 6.54 (dq, *J* = 10.0, 1.5 Hz, 1H), 4.18 (q, *J* = 7.1 Hz, 2H), 2.58 (dt, *J* = 10.1, 6.8 Hz, 1H), 1.83 (s, *J* = 1.5 Hz, 3H), 1.42 – 1.23 (m, 6H), 1.15 – 1.03 (m, 2H), 0.97 (d, *J* = 6.6 Hz, 3H), 0.92 – 0.79 (m, 6H).

^{13}C NMR (100 MHz, $CDCl_3$) δ 168.58, 148.53, 125.78, 60.42, 43.83, 31.96, 30.77, 29.25, 19.83, 19.42, 14.32, 12.41, 11.20.

HRMS (ESI): calculated $C_{13}H_{23}O_2$ for $[M+H]^+$ 213.1849, found 213.1846.

$[\alpha]_D^{25}$ = -20.0 (c = 0.2, $CHCl_3$).

Allylic alcohol (+)-S5 :

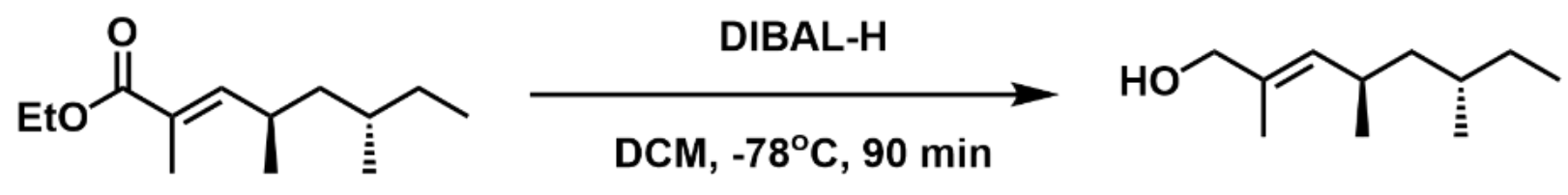

(-)-S4 (+)-S5

To a flame-dried flask under argon atmosphere was added ester (-)-**S4** (1.10 g, 5.16 mmol, 1.00 eq) in anhydrous dichloromethane (26.0 mL).This solution was cooled to -78°C, then diisobutylaluminum hydride (1.0M in hexanes, 11.4 mL, 11.4 mmol, 2.20 eq) was added dropwise. The resulting mixture was left to stir at -78°C for 90 minutes. The reaction was quenched by addition of saturated potassium sodium tartrate, extracting with ethyl acetate. The combined organic layers were washed with brine, dried over sodium sulfate, and concentrated *in vacuo* to give crude material. Purification by column chromatography (0-20% ethyl acetate in hexanes) gave allylic alcohol **(+)-S5** as a clear, colorless oil in 95% yield (832 mg, 4.89 mmol).

R_f = 0.2 (10% ethyl acetate in hexanes, visualized by $KMnO_4$)

^{1}H NMR (400 MHz, $CDCl_3$) δ 5.18 (dd, J = 9.4, 1.4 Hz, 1H), 3.99 (s, 2H), 2.48 (m, J = 9.4, 6.9 Hz, 1H), 1.67 (d, J = 1.4 Hz, 3H), 1.42 – 1.29 (m, 1H), 1.28 – 1.16 (m, 2H), 1.15 – 0.98 (m, 2H), 0.90 (d, J = 6.6 Hz, 3H), 0.87 – 0.78 (m, 6H).

^{13}C NMR (100 MHz, $CDCl_3$) δ 133.50, 132.62, 69.16, 44.65, 31.89, 29.50, 29.35, 20.74, 19.48, 13.76, 11.26.

HRMS (ESI): calculated $C_{11}H_{22}ONa$ for $[M+Na]^+$ 193.1563, found 193.1562.

$[\alpha]_D^{25}$ = +0.4 (c = 0.2, $CHCl_3$).

Homoallylic alcohol (+)-20 :

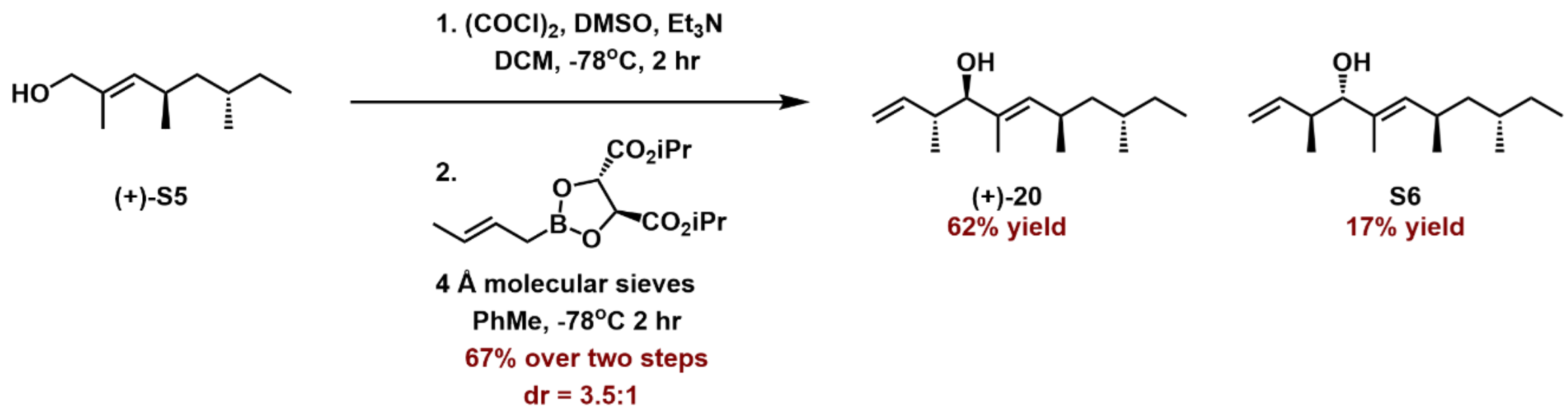

To a flame-dried flask under argon atmosphere was added oxalyl chloride (631 µL, 7.33 mmol, 1.50 eq) in anhydrous dichloromethane (24 mL) at −78 °C. Dimethyl sulfoxide (940 µL, 13.19 mmol, 2.70 eq) was added dropwise, and the reaction mixture was stirred at -78ºC for 15 minutes. A solution of allylic alcohol (+)-**S5** (832.2 mg, 4.887 mmol, 1.00 eq) in anhydrous dichloromethane (6.0 mL) was added dropwise, and the reaction mixture was stirred for an additional 30 minutes at −78 °C. Triethylamine (2.04 mL, 14.66 mmol, 3.00 eq) was then added dropwise, and the reaction mixture was allowed to warm to 0 °C over 2 hours. The reaction was quenched with the addition of saturated aqueous ammonium chloride, extracting with dichloromethane. The combined organic layers were washed with brine twice, dried over anhydrous sodium sulfate, and concentrated *in vacuo* to give crude aldehyde **(+)-12**, which was used immediately without further purification.

R$_f$ = 0.5 (9% ethyl acetate in hexanes,, visualized by *p*-anisaldehyde)

To a flame-dried flask under argon atmosphere containing 4 Å molecular sieves (1.20 g) in anhydrous toluene (24 mL) was added freshly prepared diisopropyl (4*S*,5*S*)-2-((*E*)-but-2-en-1-yl)-1,3,2-dioxaborolane-4,5-dicarboxylate[8] (1.0 M in toluene, 10.0 mL, 10.0 mmol, 2.05 eq). The resulting suspension was cooled to -78ºC prior to the dropwise addition of aldehyde **(+)-12**, after which the reaction mixture was stirred at -78ºC for 2 hours then allowed to warm to room temperature. The reaction was quenched by addition of deionized water, extracting with diethyl ether. The combined organic layers were washed with brine, dried over sodium sulfate, and concentrated in vacuo to give crude material as a mixture of diastereomers (dr = 3.5:1). Purification by column chromatography (0-10% ethyl acetate in hexanes) gave homoallylic alcohol **(+)-20** as the major diastereomer in 62% yield (619 mg, 2.76 mmol) as well as the minor diastereomer **S6** in 17% yield (247 mg, 1.10 mmol).

R$_f$ = 0.4 (10% ethyl acetate in hexanes, visualized by $KMnO_4$)

^{1}H NMR (400 MHz, $CDCl_3$) δ 5.74 (ddd, *J* = 17.1, 10.2, 8.4 Hz, 1H), 5.24 – 5.10 (m, 3H), 3.60 (dd, *J* = 9.0, 2.1 Hz, 1H), 2.57 – 2.42 (m, 1H), 2.30 (ddt, *J* = 15.5, 8.8, 6.9 Hz, 1H), 1.74 (d, *J* = 2.1 Hz, 1H), 1.61 (d, *J* = 1.4 Hz, 3H), 1.43 – 1.12 (m, 3H), 1.10 – 1.00 (m, 2H), 0.92 (d, *J* = 6.7 Hz, 3H), 0.89 – 0.76 (m, 9H).

^{13}C NMR (100 MHz, $CDCl_3$) δ 141.55, 136.47, 132.60, 116.46, 81.72, 44.80, 42.26, 32.00, 29.61, 28.96, 20.86, 19.61, 16.86, 11.25, 10.79.

HRMS (ESI): calculated $C_{15}H_{28}ONa$ for $[M+Na]^+$ 247.2032, found 247.2029.

$[\alpha]_D^{25}$ = +0.5 (c = 0.2, $CHCl_3$).

(**S6**, minor) **^{1}H NMR** (400 MHz, $CDCl_3$) δ 5.74 (ddd, J = 17.2, 10.2, 8.3 Hz, 1H), 5.19 – 5.16 (m, 1H), 5.13 (tdd, J = 8.9, 5.8, 2.0 Hz, 2H), 3.62 (dd, J = 8.6, 5.8 Hz, 1H), 2.57 – 2.41 (m, 1H), 2.31 (h, J = 7.4 Hz, 1H), 1.62 (dd, J = 2.9, 1.4 Hz, 3H), 1.41 – 1.17 (m, 3H), 1.16 – 0.98 (m, 2H), 0.93 – 0.80 (m, 12H).

Diene (+)-S7 :

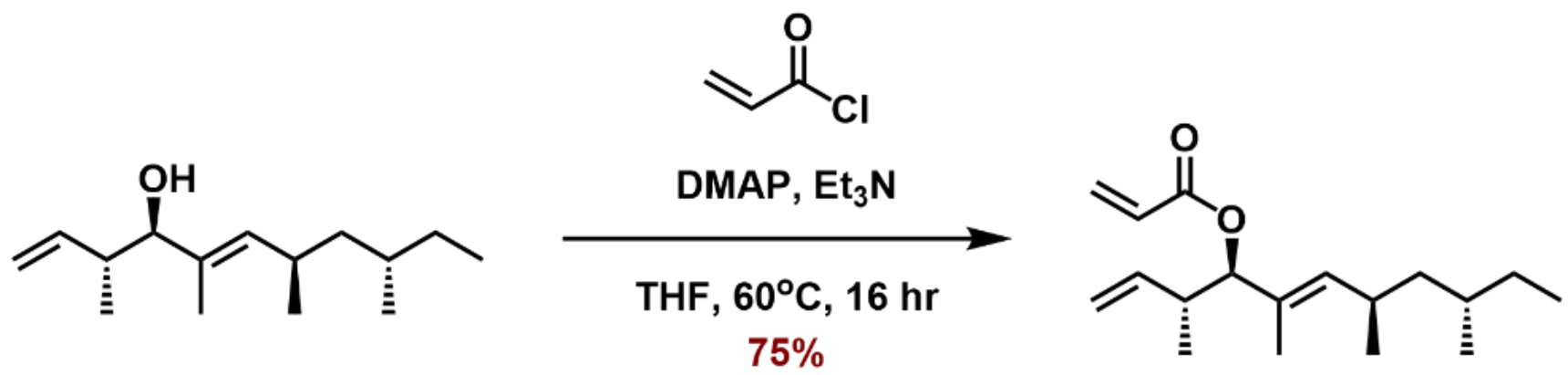

(+)-20 (+)-S7

To a flame-dried flask under argon atmosphere was added homoallylic alcohol **(+)-20** (100 mg, 446 µmol, 1.00 eq) and 4-dimethylaminopyridine (10.9 mg, 89.1 µmol, 0.20 eq) in a 4:1 mixture of anhydrous tetrahydrofuran and triethylamine (10.0 mL) at room temperature. Acryloyl chloride (72.4 µL, 891 µmol, 2.00 eq) was added, and the reaction mixture was stirred at 60 °C for 16 hours. Additional acryloyl chloride was added as needed until complete consumption of starting material was observed by TLC. The reaction was quenched with saturated aqueous ammonium chloride at 0 °C, extracting with diethyl ether. The combined organic layers were washed with brine, dried over anhydrous sodium sulfate, and concentrated in vacuo to give crude material. Purification by column chromatography (0-5% ethyl acetate in hexanes) afforded diene **(+)-S7** as a clear, colorless oil in 75% yield (93 mg, 330 µmol).

R_f = 0.8 (10% ethyl acetate in hexanes, visualized by $KMnO_4$)

^{1}H NMR (400 MHz, $CDCl_3$) δ 6.36 (dd, J = 17.4, 1.6 Hz, 1H), 6.09 (dd, J = 17.3, 10.4 Hz, 1H), 5.78 (dd, J = 10.4, 1.6 Hz, 1H), 5.70 (ddd, J = 17.2, 10.3, 8.0 Hz, 1H), 5.25 (dd, J = 9.5, 1.6 Hz, 1H), 5.06 – 4.93 (m, 3H), 2.47 (m, 2H), 1.62 (d, J = 1.4 Hz, 3H), 1.40 – 1.27 (m, 2H), 1.22 (dq, J = 20.2, 6.7 Hz, 1H), 1.04 (dtd, J = 13.9, 7.1, 1.6 Hz, 2H), 0.90 (dd, J = 10.5, 6.8 Hz, 6H), 0.86 – 0.76 (m, 6H).

^{13}C NMR (101 MHz, $CDCl_3$) δ 165.32, 140.46, 137.64, 130.10, 129.35, 129.07, 115.05, 82.91, 44.64, 40.23, 32.02, 29.66, 28.98, 20.70, 19.59, 16.67, 11.82, 11.26.

HRMS (ESI): calculated $C_{18}H_{30}O_2Na$ for $[M+Na]^+$ 301.2138, found 301.2134.

$[\alpha]_D^{25}$ = +0.5 (c = 0.2, $CHCl_3$).

α, β – unsaturated lactone (-)-S8 :

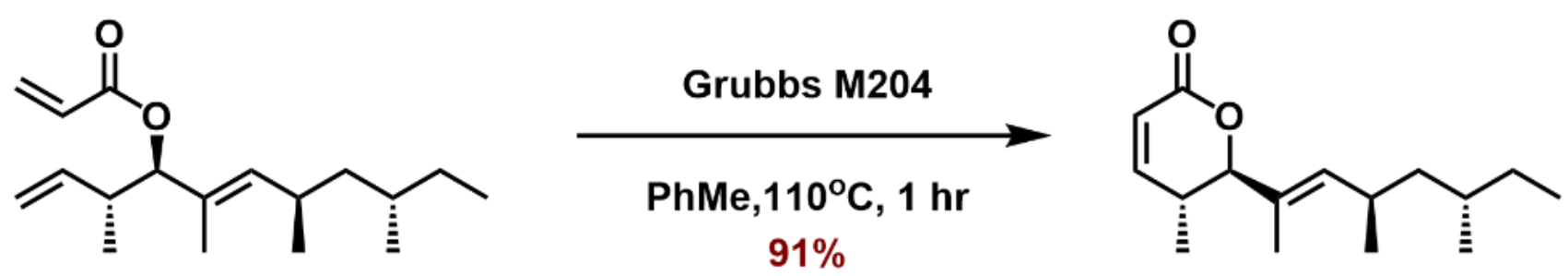

(+)-S7 (-)-S8

To a flame-dried flask under argon atmosphere was added diene **(+)-S7** (87.8 mg, 315 µmol, 1.00 eq) in anhydrous toluene (5.0 mL) at room temperature. This solution was sparged with argon for 30 minutes prior to the addition of Grubbs second-generation catalyst (6.70 mg, 7.88 µmol, 2.5 mol%), then left to stir at reflux for 1 hour under argon atmosphere. Concentration of the reaction mixture *in vacuo* gave crude material, which was then purified by column chromatography (0-25% ethyl acetate in hexanes) to give lactone **(-)-S8** as a clear, colorless oil in 91% yield (71.5 mg, 286 µmol).

R_f = 0.5 (20% ethyl acetate in hexanes, visualized by $KMnO_4$)

^{1}H NMR (400 MHz, $CDCl_3$) δ 6.68 (dd, *J* = 9.8, 1.9 Hz, 1H), 5.97 (dd, *J* = 9.8, 2.7 Hz, 1H), 5.26 (dd, *J* = 9.6, 1.5 Hz, 1H), 4.32 (d, *J* = 11.4 Hz, 1H), 2.69 (dqt, *J* = 11.9, 7.2, 2.4 Hz, 1H), 2.52 (dddt, *J* = 9.5, 7.8, 6.3, 4.5 Hz, 1H), 1.68 (d, *J* = 1.4 Hz, 3H), 1.43 – 1.17 (m, 3H), 1.13 – 1.01 (m, 2H), 0.99 (d, *J* = 7.3 Hz, 3H), 0.93 (d, *J* = 6.6 Hz, 3H), 0.84 (dt, *J* = 7.5, 4.1 Hz, 6H).

^{13}C NMR (100 MHz, $CDCl_3$) δ 164.65, 152.02, 139.93, 128.31, 120.21, 90.77, 44.50, 32.09, 30.88, 29.82, 28.91, 20.48, 19.58, 15.63, 11.31, 11.25.

HRMS (ESI): calculated $C_{16}H_{26}O_2Na$ for $[M+Na]^+$ 273.1825, found 273.1820.

$[\alpha]_D^{25}$ = -65.0 (c = 0.2, $CHCl_3$).

Lactol (-)-15 :

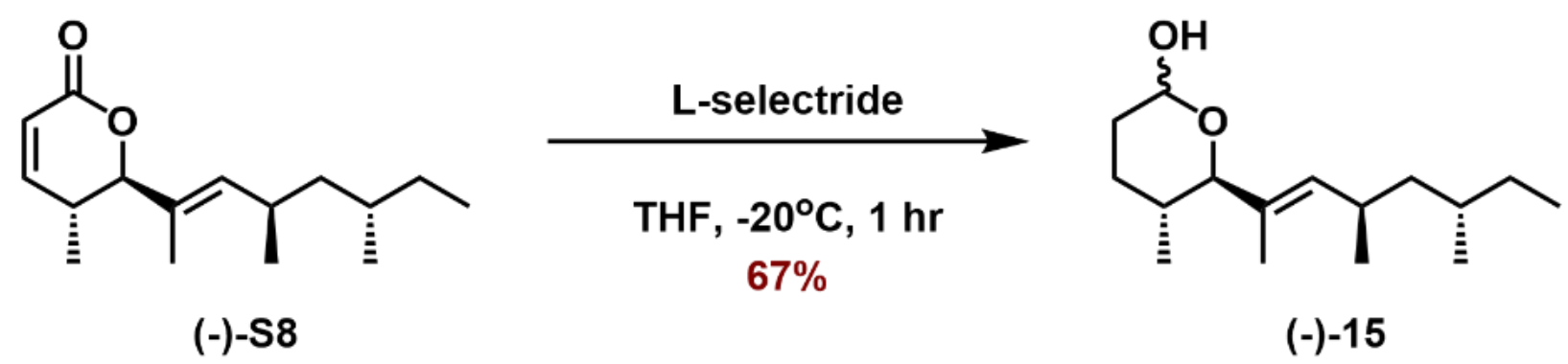

To a flame-dried flask under argon atmosphere was added lactone **(-)-S8** (71.5 mg, 286 µmol, 1.00 eq) in anhydrous tetrahydrofuran (5.0 mL). Lithium tri-*sec*-butylborohydride (1.0 M in THF, 685 µL, 685 µmol, 2.40 eq) was added at -20 °C, and the resulting solution reaction mixture was stirred for 1 hour at -20ºC. The reaction was quenched by addition of saturated aqueous ammonium chloride at 0 °C, extracting with diethyl ether. The combined organic layers were washed with brine, dried over sodium sulfate, and concentrated *in vacuo* to give crude material. Purification by column chromatography (0-25% ethyl acetate in hexanes) gave lactol **(-)-15** as a clear, colorless oil in 67% yield (49 mg, 190 µmol).

NOTE: The product was obtained as an inseparable mixture of diastereomers, resulting in overlapping signals in the NMR spectra, preventing complete and accurate assignment.

$\mathbf{R_f}$ = 0.35 (14% ethyl acetate in hexanes, visualized by *p*-anisaldehyde, $KMnO_4$, and CAM)

^{1}**H NMR** (400 MHz, $CDCl_3$) δ 5.34 (q, *J* = 2.5 Hz, 1H), 5.17 (ddd, *J* = 16.9, 9.5, 1.6 Hz, 2H), 4.75 (ddd, *J* = 9.5, 5.5, 2.2 Hz, 1H), 3.91 (d, *J* = 9.4 Hz, 1H), 3.37 (d, *J* = 9.9 Hz, 1H), 3.19 (d, *J* = 5.5 Hz, 1H), 2.63 (t, *J* = 2.2 Hz, 1H), 2.48 (m, 2H), 1.90 – 1.79 (m, 2H), 1.73 (ddq, *J* = 9.0, 6.0, 2.8 Hz, 2H), 1.67 – 1.50 (m, 10H), 1.49 – 1.14 (m, 9H), 1.09 – 0.99 (m, 3H), 0.93 (d, *J* = 6.6 Hz, 6H), 0.83 (ddd, *J* = 7.5, 4.6, 3.4 Hz, 12H), 0.70 (dd, *J* = 11.6, 6.2 Hz, 6H).

^{13}**C NMR** (100 MHz, $CDCl_3$) δ 137.16, 137.00, 131.57, 131.39, 96.40, 91.83, 89.37, 81.98, 44.82, 33.07, 32.03, 31.97, 31.34, 31.11, 30.24, 29.54, 29.51, 28.95, 26.05, 20.84, 20.79, 19.60, 17.95, 17.11, 11.29, 11.26, 11.01.

HRMS (ESI): calculated $C_{16}H_{30}O_2Na$ for $[M+Na]^+$ 277.2135, found 277.2138.

$[\alpha]_D^{25}$ = -32.0 (c = 0.2, $CHCl_3$).

N-methylpyridone **S10** :

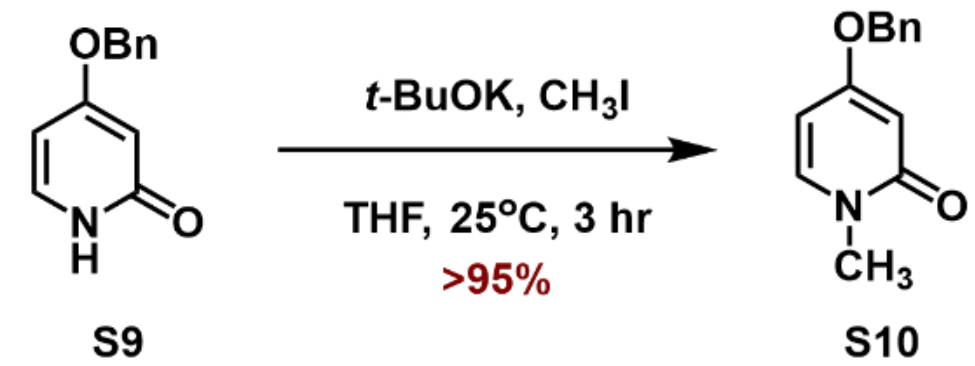

To a flame-dried flask under argon atmosphere was added potassium *tert*-butoxide (6.13 g, 54.7 mmol, 1.1 eq) and pyridone **S9** (10.0 g, 49.7 mmol, 1.0 eq) in anhydrous tetrahydrofuran (150 mL). To this was then added iodomethane (4.66 mL, 74.5 mmol, 1.5 eq) dropwise and the resulting suspension was left to stir at room temperature for 3 hours. To the reaction mixture was then added 1M aqueous sodium hydroxide, extracting with ethyl acetate. The combined organic layers were then washed with saturated sodium chloride and dried over sodium sulfate, then concentrated *in vacuo* to give *N*-methylpyridone **S10** as an off-white solid (10.41 g, 48.36 mmol) in 97% yield without need for further purification.

$\mathbf{R_f}$ = 0.30 (10% acetone in dichloromethane, visualized by UV)

^{1}H NMR (400 MHz, $CDCl_3$) δ 7.44 – 7.30 (m, 5H), 7.14 (d, *J* = 7.5 Hz, 1H), 5.99 (d, *J* = 2.7 Hz, 1H), 5.95 (dd, *J* = 7.5, 2.7 Hz, 1H), 4.98 (s, 2H), 3.47 (s, 3H).

^{13}C NMR (101 MHz, $CDCl_3$) δ 167.19, 164.50, 138.12, 135.32, 128.74, 128.49, 127.74, 100.93, 98.29, 70.15, 36.87.

HRMS (ESI): calculated $C_{13}H_{14}O_2N$ for $[M+H]^+$ 216.1019, found 216.1016.

3-bromopyridone S11 :

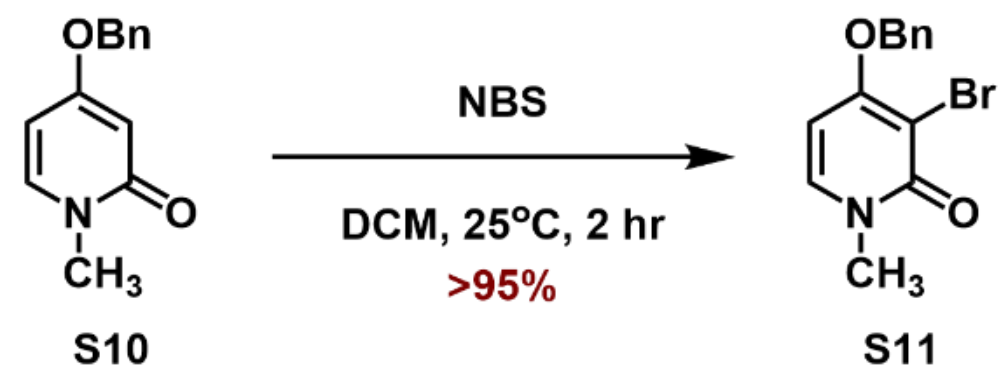

To a flame-dried flask under argon atmosphere was added *N*-methylpyridone **S10** (6.12 g, 28.4 mmol, 1.0 eq) in anhydrous dichloromethane (95 mL). To this was then added freshly recrystallized *N*-bromosuccinimide (5.06 g, 29.4 mmol, 1.00 eq) and the resulting yellow solution was left to stir at room temperature for 2 hours. To the reaction mixture was then added saturated sodium thiosulfate, extracting with additional dichloromethane. The combined organic layers were then washed with saturated sodium chloride and dried over sodium sulfate, then concentrated *in vacuo* to give bromopyridone **S11** as a pale yellow solid (8.22 g, 27.9 mmol) in 98% yield without need for further purification.

R_f = 0.50 (10% acetone in dichloromethane, visualized by UV)

^{1}H NMR (400 MHz, $CDCl_3$) δ 7.44 – 7.30 (m, 5H), 7.22 (d, *J* = 7.6 Hz, 1H), 6.05 (d, *J* = 7.7 Hz, 1H), 5.23 (s, 2H), 3.56 (s, 3H).

^{13}C NMR (101 MHz, $CDCl_3$) δ 163.34, 160.34, 137.28, 135.42, 128.80, 128.37, 126.78, 99.76, 95.62, 71.02, 38.13.

HRMS (ESI): calculated $C_{13}H_{13}O_2NBr$ for $[M+H]^+$ 294.0124, found 294.0126.

3-bromo-5-iodopyridone 17 :

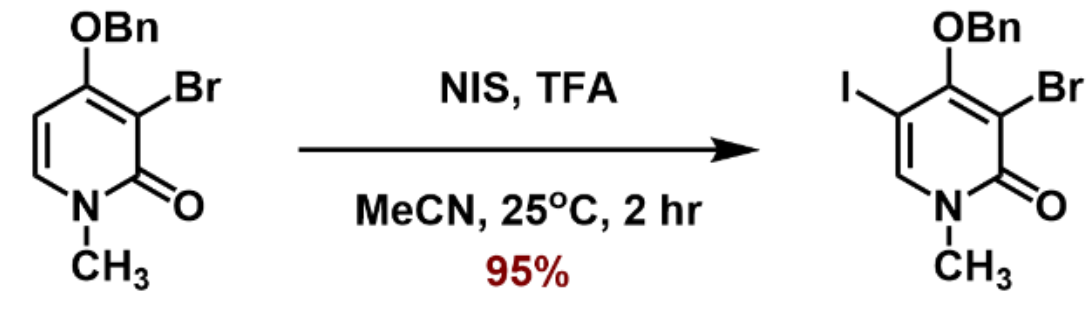

To a flame-dried flask under argon atmosphere was added bromopyridone **S11** (14.345 g, 48.768 mmol, 1.0 eq) in anhydrous acetonitrile (140 mL). To this was then added *N*-iodosuccinimide (14.264 g, 63.398 mmol, 1.30 eq) followed by trifluoroacetic acid (1.13 mL, 14.630 mmol, 0.3 eq). The resulting yellow solution was left to stir at room temperature for 2 hours. The reaction mixture was concentrated *in vacuo*, then the crude material was taken up in dichloromethane. To the reaction mixture was then added saturated sodium thiosulfate, extracting with additional dichloromethane. The combined organic layers were then washed with water three times followed by saturated sodium chloride, then dried over sodium sulfate and concentrated *in vacuo* to give 3-bromo-5-iodopyridone **17** as a light brown solid (19.39 g, 48.16 mmol) in 95% yield without need for further purification.

R_f = 0.60 (10% acetone in dichloromethane, visualized by UV)

^{1}H NMR (400 MHz, $CDCl_3$) δ 7.66 (s, 1H), 7.62 – 7.51 (m, 2H), 7.45 – 7.36 (m, 3H), 5.12 (s, 2H), 3.60 (s, 3H).

^{13}C NMR (101 MHz, $CDCl_3$) δ 163.34, 160.25, 142.47, 135.27, 128.79, 128.59, 108.06, 74.66, 65.92, 38.35, 29.60.

HRMS (ESI): calculated $C_{13}H_{12}O_2NBrI$ for $[M+H]^+$ 419.9091, found 419.9088.

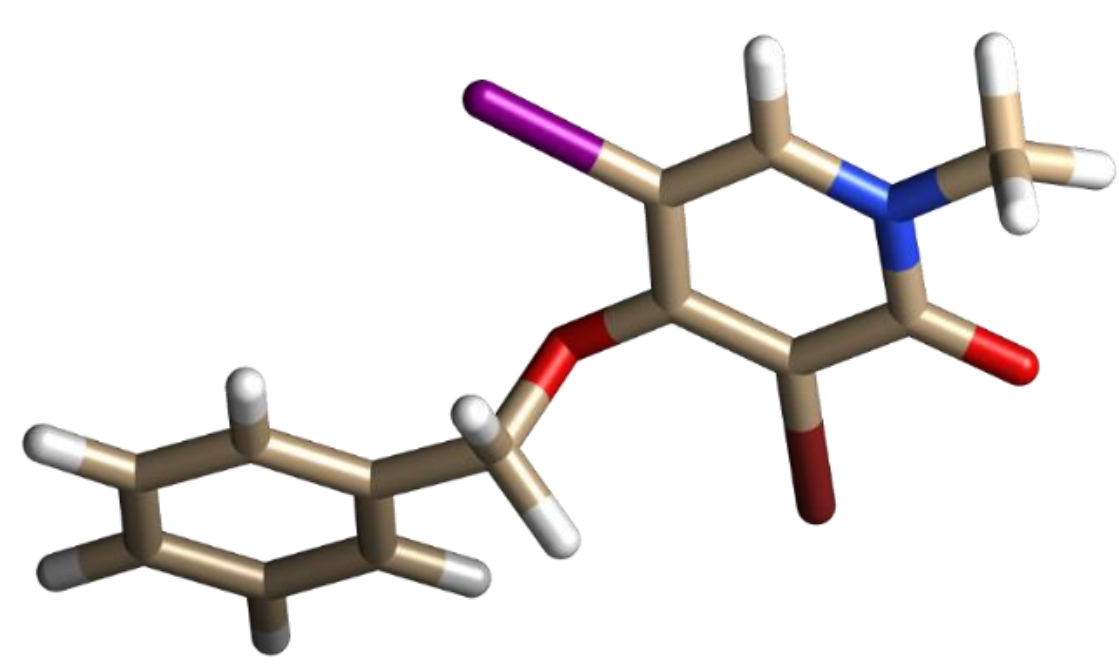

Figure S12. X-ray crystal structure of compound **17** - see attached CIF file "Compound 17"

General Procedure A: Suzuki-Miyaura coupling

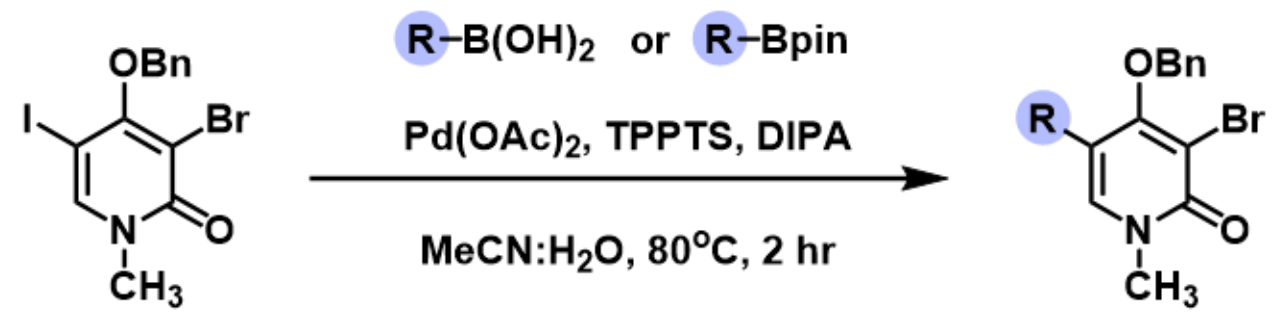

To a flask under argon atmosphere was added pyridone **17** (1.0 eq), boronic acid or boronate (1.1 eq), and triphenylphosphine-3,3',3''-trisulfonic acid (TPPTS, 0.37 eq) in a 3:1 mixture of acetonitrile and water (72 mM). This suspension was generously degassed with argon prior to the successive addition of palladium(II) acetate (15 mol%) followed by diisopropylamine (3.2 eq). This suspension was then left to stir at 80°C for 2 hours, after which the crude reaction mixture was filtered through a pad of Celite, washing with dichloromethane. The collected filtrate was concentrated *in vacuo*, and the crude material obtained was purified by column chromatography (0-30% acetone in dichloromethane) to give the desired product.

Pyridone phenol S12 :

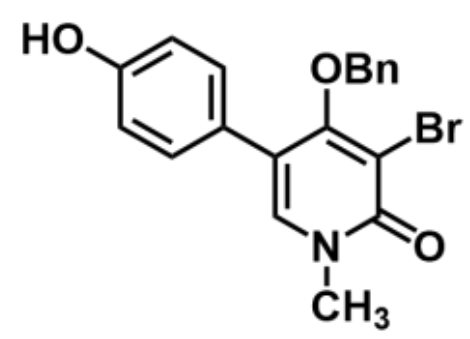

S12

Following **General Procedure A**, coupling of pyridone **17** (2.00 g, 4.76 mmol) with 4-hydroxyphenylboronic acid (722 mg, 5.24 mmol) gave pyridone phenol **S12** as a brown solid in 91% yield (1.6786 g, 4.3460 mmol).

R_f = 0.55 (20% acetone in dichloromethane, visualized by UV and $KMnO_4$)

^{1}H NMR (400 MHz, CD_3OD) δ 7.67 (s, 1H), 7.34 – 7.22 (m, 5H), 7.16 (td, *J* = 4.9, 3.1 Hz, 2H), 6.90 – 6.82 (m, 2H), 4.67 (s, 2H), 3.65 (s, 3H).

^{13}C NMR (101 MHz, CD_3OD) δ 167.97, 164.60, 161.36, 141.65, 139.52, 134.07, 132.38, 132.03, 131.82, 128.45, 122.69, 119.09, 110.91, 77.99, 41.27.

HRMS (ESI): calculated $C_{19}H_{17}O_3NBr$ for $[M+H]^+$ 386.0386, found 386.0383.

Phenylpyridone S13 :

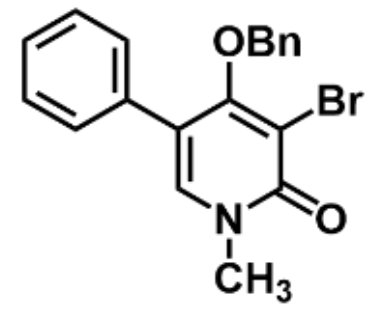

S13

Following **General Procedure A**, coupling of pyridone **17** (2.00 g, 4.76 mmol) with phenylboronic acid (639 mg, 5.24 mmol) gave phenylpyridone **S13** as a brown solid in 81% yield (1.4345 g, 3.8744 mmol).

R_f = 0.65 (20% acetone in dichloromethane, visualized by UV and $KMnO_4$)

^{1}H NMR (400 MHz, $CDCl_3$) δ 7.38 (d, *J* = 1.8 Hz, 5H), 7.25 – 7.22 (m, 3H), 7.09 (dd, *J* = 7.4, 2.1 Hz, 2H), 4.61 (s, 2H), 3.63 (s, 3H).

^{13}C NMR (101 MHz, $CDCl_3$) δ 163.16, 160.21, 136.66, 135.32, 133.73, 129.01, 128.76, 128.73, 128.50, 128.34, 128.02, 118.11, 108.77, 74.54, 38.38.

HRMS (ESI): calculated $C_{19}H_{17}O_2NBr$ for $[M+H]^+$ 370.0437, found 370.0441.

General Procedure B: Global hydrogenolysis of 5-aryl-3-bromopyridones

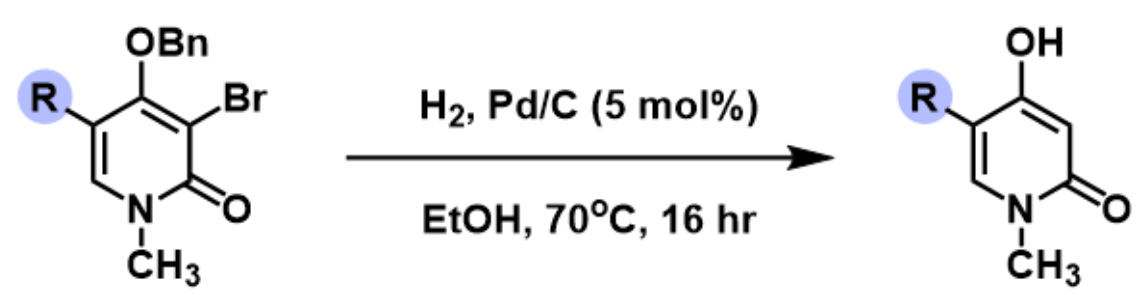

To a flask containing 3-bromopyridone (1.0 eq) in ethanol was added 5% palladium on activated charcoal (5 mol%). This suspension was backfilled five times with hydrogen, then the reaction mixture was left to stir at 70°C for 16 hours under hydrogen atmosphere. The crude reaction mixture was then filtered through a pad of Celite, washing with methanol. The filtrate obtained was concentrated *in vacuo* to give the desired product, without need for further purification.

4-hydroxy-2-pyridone 22 :

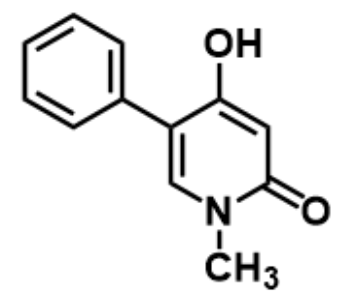

22

Following **General Procedure B**, hydrogenolysis of bromopyridone **S13** (1.00 g, 2.70 mmol) gave 4-hydroxy-2-pyridone **22** as a light brown solid in 82% yield (448 mg, 2.23 mmol).

R_f = 0.35 (50% acetone in dichloromethane, visualized by UV and $KMnO_4$)

^{1}H NMR (400 MHz, CD_3OD) δ 7.57 (d, J = 3.0 Hz, 1H), 7.44 (dq, J = 6.4, 1.5 Hz, 2H), 7.38 – 7.32 (m, 2H), 7.31 – 7.27 (m, 1H), 5.94 (d, J = 3.0 Hz, 1H), 3.51 (d, J = 3.0 Hz, 3H).

^{13}C NMR (101 MHz, CD_3OD) δ 165.95, 164.74, 138.95, 133.85, 128.77, 127.80, 126.95, 116.10, 98.11, 35.82.

HRMS (ESI): calculated $C_{12}H_{12}O_2N$ for $[M+H]^+$ 202.0863, found 202.0861.

4-hydroxy-2-pyridone 23 :

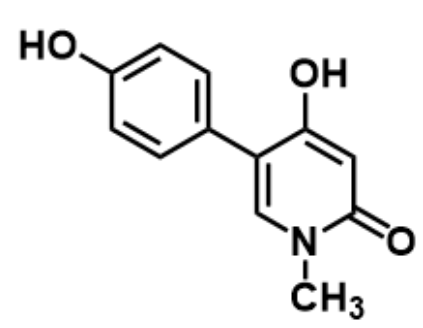

23

Following **General Procedure B**, hydrogenolysis of bromopyridone **S12** (738 mg, 1.91 mmol) gave 4-hydroxy-2-pyridone **23** as a light brown solid in 95% yield (390 mg, 1.80 mmol).

R_f = 0.25 (50% acetone in dichloromethane, visualized by UV and $KMnO_4$)

^{1}H NMR (400 MHz, CD_3OD) δ 7.56 (s, 1H), 7.31 – 7.23 (m, 2H), 6.83 – 6.74 (m, 2H), 6.00 (s, 1H), 3.54 (s, 3H).

^{13}C NMR (101 MHz, CD_3OD) δ 166.55, 164.04, 156.83, 138.63, 129.95, 124.49, 117.00, 114.64, 97.77, 36.06.

HRMS (ESI): calculated $C_{12}H_{12}O_3N$ for $[M+H]^+$ 218.0812, found 218.0808.

(-)-sambutoxin A (3) :

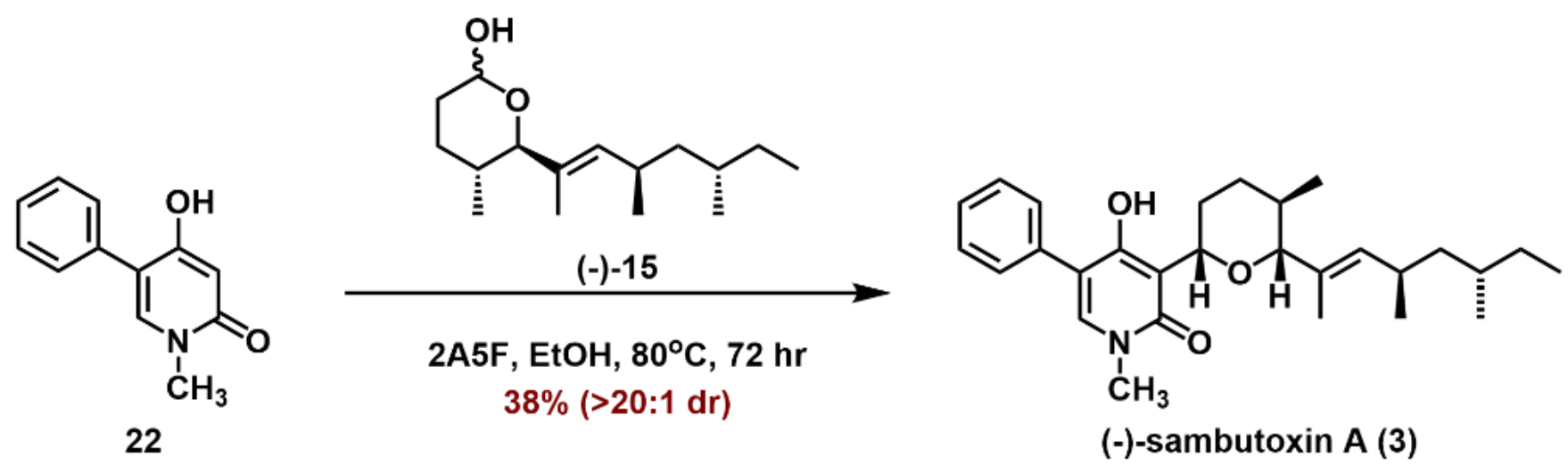

To an oven-dried vial under argon atmosphere was added 4-hydroxy-2-pyridone **22** (5.1 mg, 26 µmol, 1.30 eq) and 2-amino-5-fluoropyridine (11.0 mg, 98 µmol, 5.00 eq). To this solution was then added lactol **(-)-15** (5.0 mg, 20 µmol, 1.00 eq) in absolute ethanol (500 µL), then the reaction mixture was left to stir at 80°C for 72 hours. The crude reaction mixture was then taken up in dichloromethane, washing with 1M aqueous hydrochloric acid. The combined organic layers were washed with saturated sodium chloride, dried over sodium sulfate, then concentrated in vacuo to give crude material. Purification by column chromatography (0-30% acetone in dichloromethane) afforded (-)-sambutoxin A (**3**) as a yellow amorphous solid in 38% yield (3.3 mg, 7.5 µmol, >20:1 dr).

R_f = 0.4 (20% acetone in dichloromethane, visualized by UV and $KMnO_4$)

^{1}H NMR (800 MHz, $CDCl_3$) δ 9.98 (s, 1H), 7.43 (dd, *J* = 8.2, 1.4 Hz, 2H), 7.39 (dd, *J* = 8.6, 6.8 Hz, 2H), 7.34 – 7.31 (m, 1H), 7.15 (s, 1H), 5.20 (dd, *J* = 9.5, 1.6 Hz, 1H), 5.03 (dd, *J* = 11.3, 2.3 Hz, 1H), 3.53 (d, *J* = 10.1 Hz, 1H), 3.51 (s, 3H), 2.50 – 2.44 (m, 1H), 2.10 (dq, *J* = 13.6, 3.0 Hz, 1H), 1.92 (dq, *J* = 13.4, 3.5 Hz, 1H), 1.72 – 1.66 (m, 1H), 1.66 – 1.63 (m, 1H), 1.62 (d, *J* = 1.3 Hz, 3H), 1.44 (qd, *J* = 13.0, 3.8 Hz, 1H), 1.38 – 1.32 (m, 1H), 1.32 – 1.27 (m, 1H), 1.20 (dt, *J* = 13.7, 6.9 Hz, 1H), 1.08 – 1.02 (m, 2H), 0.90 (d, *J* = 6.6 Hz, 3H), 0.83 (dt, *J* = 7.4, 4.1 Hz, 6H), 0.75 (d, *J* = 6.6 Hz, 3H).

^{13}C NMR (201 MHz, $CDCl_3$) δ 161.77, 161.38, 137.96, 136.26, 134.09, 130.26, 129.18, 128.30, 127.36, 115.07, 110.30, 92.60, 77.90, 44.73, 36.99, 32.37, 32.18, 31.96, 30.67, 29.60, 28.91, 20.68, 19.59, 17.68, 11.64, 11.21.

HRMS (ESI): calculated $C_{28}H_{40}NO_3$ for $[M+H]^+$ 438.3003, found 438.3002.

$[\alpha]_D^{25}$ = -29.4 (*c* = 0.13, MeOH)

(-)-sambutoxin (4) :

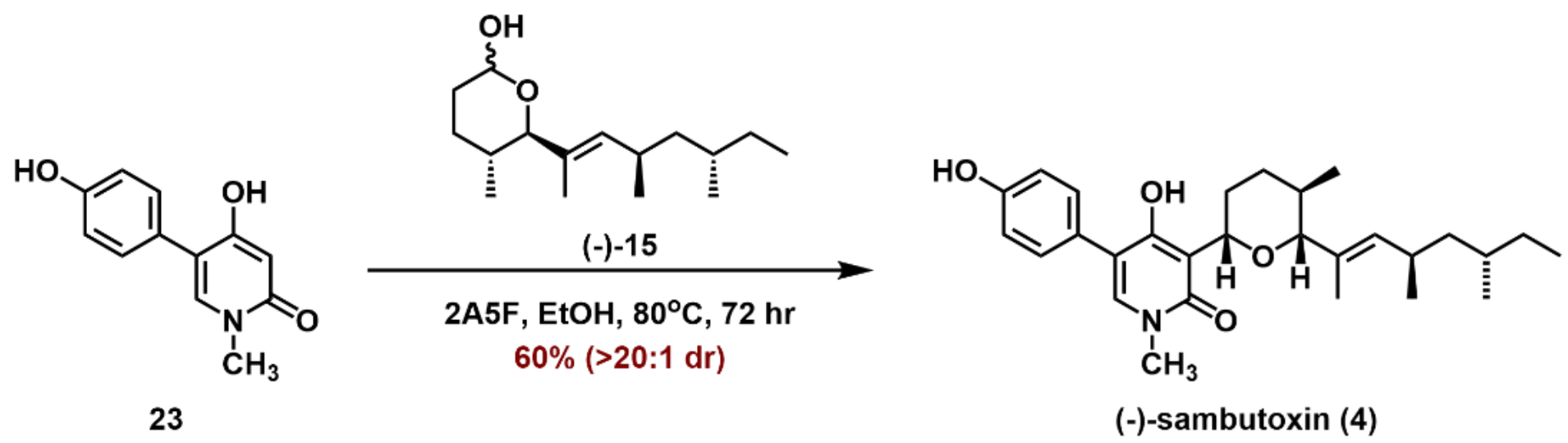

To an oven-dried vial under argon atmosphere was added 4-hydroxy-2-pyridone **23** (24.1 mg, 111 µmol, 1.50 eq) and 2-amino-5-fluoropyridine (41.4 mg, 369 µmol, 5.00 eq). To this solution was then added lactol **(-)-15** (18.8 mg, 73.9 µmol, 1.00 eq) in absolute ethanol (2.0 mL), then the reaction mixture was left to stir at 80°C for 72 hours. The crude reaction mixture was then taken up in dichloromethane, washing with 1M aqueous HCl. The combined organic layers were washed with saturated sodium chloride, dried over sodium sulfate, then concentrated *in vacuo* to give crude material. Purification by column chromatography (0-30% acetone in dichloromethane) afforded (-)-sambutoxin (**4**) as an amorphous white solid in 60% yield (20 mg, 44 µmol, >20:1 dr).

R_f = 0.4 (11% hexanes in ethyl acetate, visualized by *p*-anisaldehyde, $KMnO_4$ and CAM)

^{1}H NMR (600 MHz, $CDCl_3$) δ 10.03 (s, 1H), 7.29 – 7.27 (m, 2H), 7.12 (s, 1H), 6.92 – 6.89 (m, 2H), 5.19 (dd, *J* = 9.5, 1.6 Hz, 1H), 5.04 (dd, *J* = 11.3, 2.3 Hz, 1H), 3.52 (s, 3H), 3.51 (s, 1H), 2.46 (tdt, *J* = 13.1, 8.9, 6.5 Hz, 1H), 2.12 – 2.06 (m, 1H), 1.90 (dq, *J* = 13.4, 3.4 Hz, 1H), 1.73 – 1.63 (m, 1H), 1.62 (d, *J* = 1.3 Hz, 3H), 1.42 (qd, *J* = 13.1, 3.8 Hz, 1H), 1.38 – 1.27 (m, 1H), 1.24 – 1.15 (m, 1H), 1.09 – 0.99 (m, 2H), 0.90 (d, *J* = 6.6 Hz, 3H), 0.84 – 0.81 (m, 6H), 0.74 (d, *J* = 6.6 Hz, 3H).

^{13}C NMR (151 MHz, $CDCl_3$) δ 162.37, 161.44, 156.05, 138.02, 135.89, 130.43, 130.20, 125.56, 115.47, 115.43, 110.40, 92.58, 77.83, 44.72, 37.25, 32.35, 32.12, 31.97, 30.72, 29.61, 28.91, 20.68, 19.59, 17.65, 11.62, 11.22.

HRMS (ESI): calculated $C_{28}H_{40}NO_4$ for $[M+H]^+$ 454.2949, found 454.2952; calculated $C_{28}H_{39}NO_4Na$ $[M+Na]^+$ 476.2769, found 476.2771.

$[\alpha]_D^{25}$ = -125.0 (c = 0.6, MeOH)

Bromopyridone S15 :

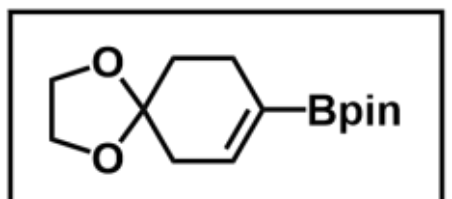

S14

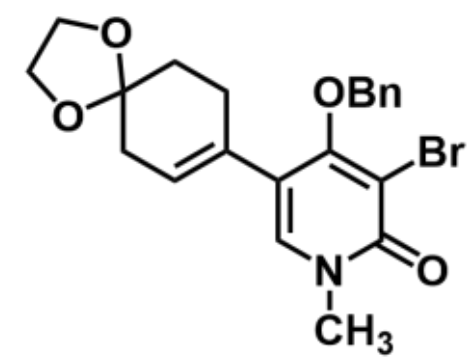

S15

Following **General Procedure A**, coupling of pyridone **17** (2.39 g, 5.70 mmol) with vinylboronate **S14** (1.6678 g, 6.2665 mmol) gave bromopyridone **S15** as a brown solid in 87% yield (2.15 g, 4.97 mmol).

$\mathbf{R_f}$ = 0.6 (20% acetone in dichloromethane, visualized by UV and $KMnO_4$)

^{1}**H NMR** (400 MHz, $CDCl_3$) δ 7.52 – 7.43 (m, 2H), 7.42 – 7.32 (m, 3H), 7.15 (d, J = 0.9 Hz, 1H), 5.64 (tt, J = 3.9, 1.6 Hz, 1H), 4.98 (s, 2H), 4.01 (p, J = 1.6 Hz, 4H), 3.57 (d, J = 1.0 Hz, 3H), 2.53 – 2.44 (m, 2H), 2.38 (dd, J = 3.9, 2.2 Hz, 2H), 1.77 (t, J = 6.5 Hz, 2H).

^{13}**C NMR** (101 MHz, $CDCl_3$) δ 207.07, 163.49, 160.27, 135.88, 135.41, 132.48, 128.89, 128.63, 128.51, 125.30, 119.42, 108.23, 107.32, 77.25, 74.78, 64.48, 53.46, 38.18, 36.22, 31.13, 30.98, 28.22, 24.88.

HRMS (ESI): calculated $C_{21}H_{23}NO_4Br$ for $[M+H]^+$ 432.0805, found 432.0802.

Alkenylpyridone 25 :

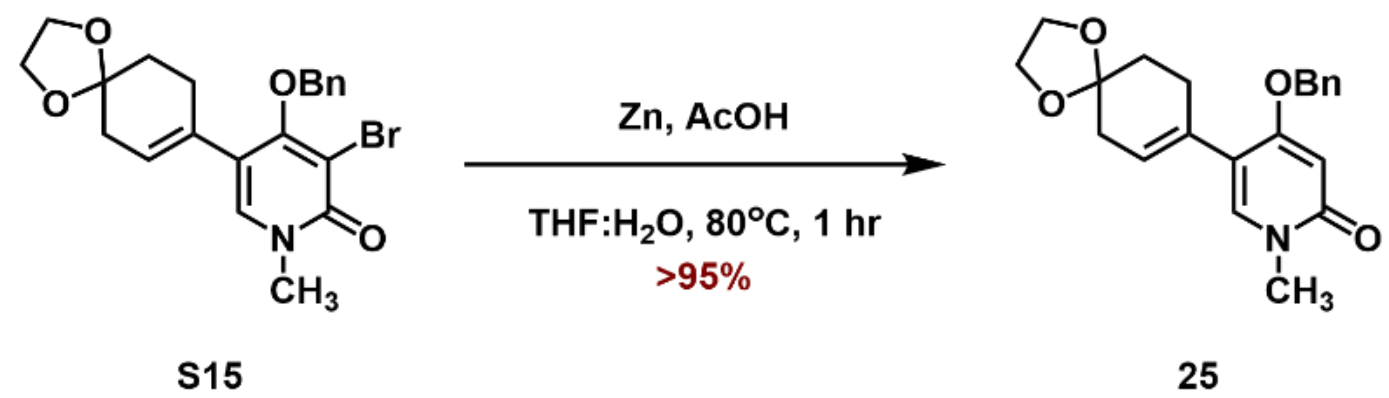

To a flask under argon atmosphere was added freshly activated zinc dust (6.44 g, 98.5 mmol, 30.0 eq) followed by bromopyridone **S15** (1.42 g, 3.28 mmol, 1.0 eq) in a 4:1 mixture of tetrahydrofuran and water (19 mL). To this was then added glacial acetic acid (940 µL, 16.4 mmol, 5.00 eq) and the resulting suspension was left to stir at 80ºC for 1 hour. The reaction mixture was filtered through celite, washing with dichloromethane. The collected filtrate was then quenched by addition of saturated sodium bicarbonate solution, extracting with additional dichloromethane. The combined organic layers were washed with brine, dried over sodium sulfate, and concentrated *in vacuo* to give crude material, which was then purified by column chromatography (0-10% methanol in ethyl acetate) to give alkenylpyridone **25** as an off-white solid in 99% yield (1.16 g, 3.28 mmol).

R_f = 0.3 (20% acetone in dichloromethane, visualized by UV and $KMnO_4$)

^{1}H NMR (400 MHz, CD_3OD) δ 7.74 (s, 1H), 7.60 – 7.51 (m, 2H), 7.49 – 7.32 (m, 3H), 6.10 (s, 1H), 5.14 (s, 2H), 4.52 (dd, J = 11.6, 4.9 Hz, 1H), 4.02 – 3.88 (m, 2H), 3.92 – 3.77 (m, 2H), 3.55 (s, 3H), 2.61 (td, J = 14.4, 4.8 Hz, 1H), 2.02 – 1.88 (m, 2H), 1.82 (ddd, J = 12.3, 5.0, 2.5 Hz, 1H), 1.61 – 1.47 (m, 2H).

^{13}C NMR (101 MHz, CD_3OD) δ 165.94, 165.03, 138.21, 135.29, 128.16, 127.76, 127.46, 118.57, 108.88, 96.98, 73.75, 70.52, 68.85, 64.01, 63.80, 38.35, 36.06, 31.33, 29.23.

HRMS (ESI): calculated $C_{21}H_{24}NO_4$ for $[M+H]^+$ 354.1700, found 354.1698.

Pyridone diol (+)-26 :

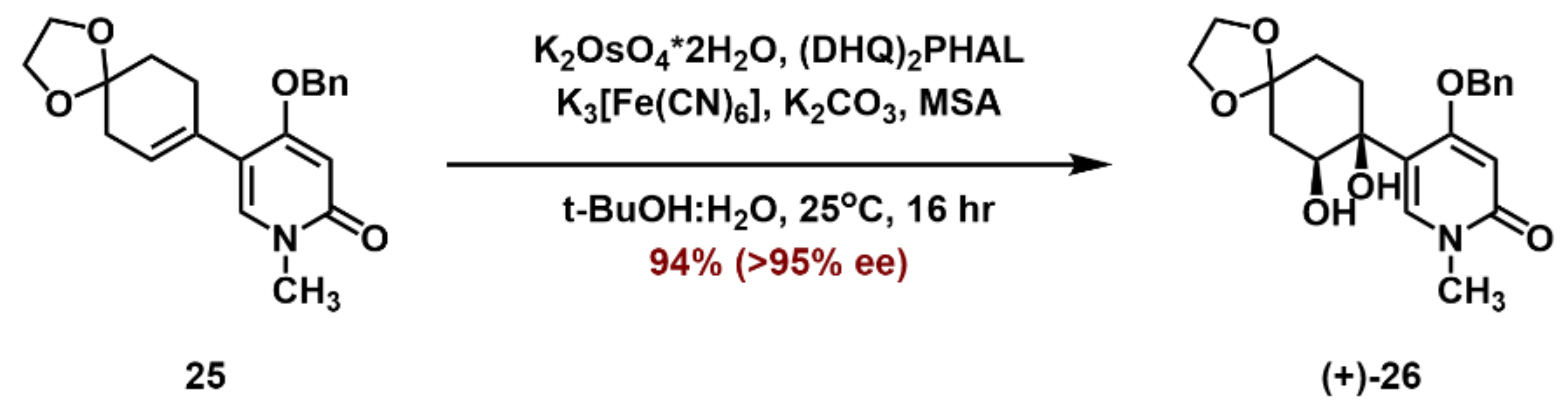

To a flask was added potassium carbonate (1.36 g, 9.85 mmol, 3.00 eq), potassium ferricyanide (2.09 g, 9.85 mmol, 3.00 eq), methanesulfonamide (375 mg, 3.94 mmol, 1.20 eq), $(DHQ)_2PHAL$ (128 mg, 164 µmol, 5 mol%) and potassium osmate(VI) dihydrate (30.2 mg, 82.1 µmol, 2.5 mol%) in a 1:1 mixture of tert-butanol and water (40 mL). This slurry was left to stir vigorously at room temperature for 15 minutes prior to the addition of alkenylpyridone **25** (1.16 g, 3.28 mmol, 1.00 eq), after which the reaction mixture was left to continue stirring at that temperature for 16 hours. Sodium sulfite (414 mg, 3.28 mmol, 1.00 eq) was added to quench the reaction mixture, which was then taken up in deionized water, extracting with a 4:1 mixture of chloroform : isopropanol three times. The combined organic layers were washed with brine, dried over sodium sulfate, and concentrated in vacuo to give crude material. Purification by column chromatography (0-10% methanol in dichloromethane) gave pyridone diol **(+)-26** as a wispy white solid in 94% yield (1.20 g, 3.10 mmol, >95% ee).

R_f = 0.25 (10% methanol in dichloromethane, visualized by UV and $KMnO_4$)

^{1}H NMR (400 MHz, CD_3OD) δ 7.74 (s, 1H), 7.60 – 7.51 (m, 2H), 7.49 – 7.32 (m, 3H), 6.10 (s, 1H), 5.14 (s, 2H), 4.52 (dd, J = 11.6, 4.9 Hz, 1H), 4.02 – 3.88 (m, 2H), 3.92 – 3.77 (m, 2H), 3.55 (s, 3H), 2.61 (td, J = 14.4, 4.8 Hz, 1H), 2.02 – 1.88 (m, 2H), 1.82 (ddd, J = 12.3, 5.0, 2.5 Hz, 1H), 1.61 – 1.47 (m, 2H).

^{13}C NMR (101 MHz, CD_3OD) δ 165.94, 165.03, 138.21, 135.29, 128.16, 127.76, 127.46, 118.57, 108.88, 96.98, 73.75, 70.52, 68.85, 64.01, 63.80, 38.35, 36.06, 31.33, 29.23.

HRMS (ESI): calculated $C_{21}H_{26}NO_6$ for $[M+H]^+$ 388.1755, found 388.1750.

$[\alpha]_D^{25}$ = +1.0 (*c* = 0.53, MeOH)

HPLC (Chiralpak IA, 25% isopropanol in hexanes, 1.0 mL/min) λ = 254 nm, t_R = 11.757 min, >95% ee

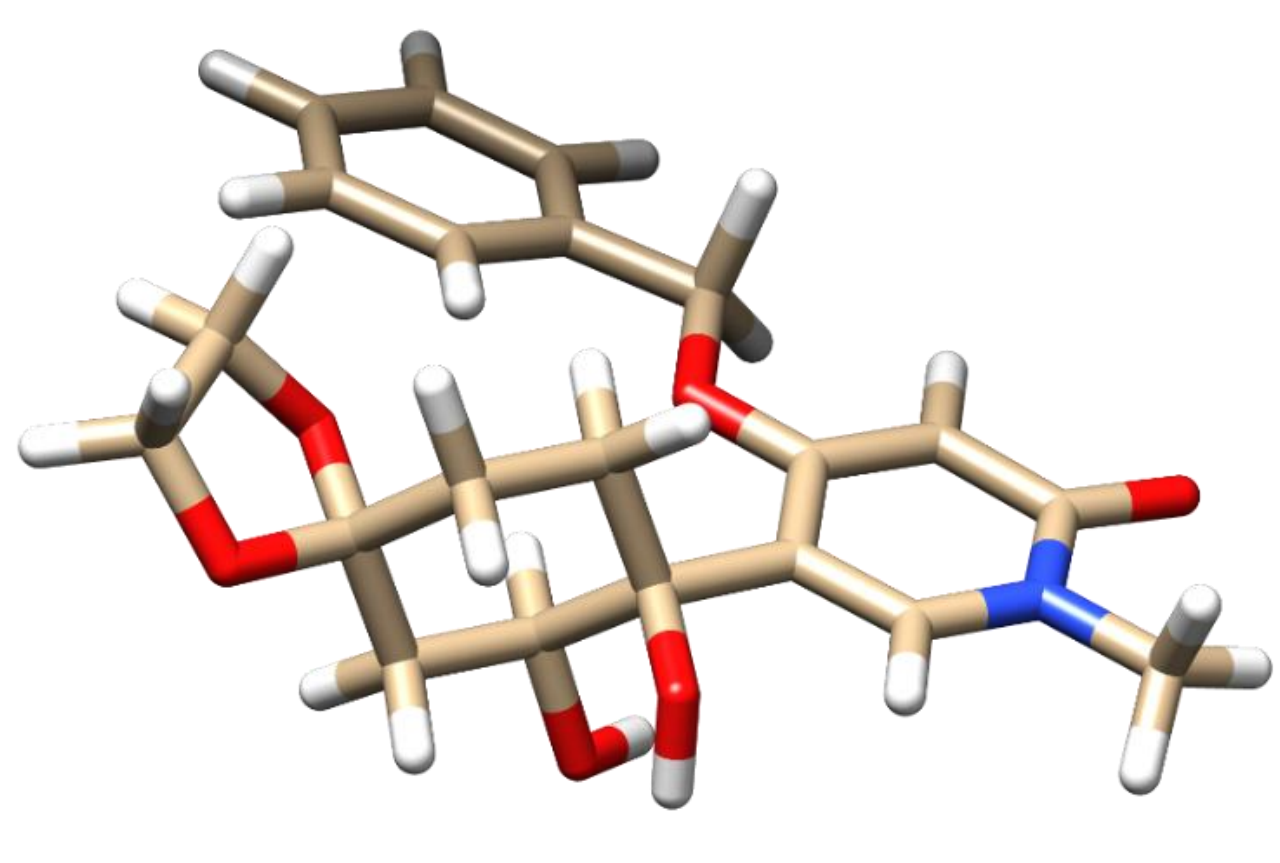

Figure S13. X-ray crystal structure of compound **(+)-26** - see attached CIF file "Compound (+)-26"

4-hydroxy-2-pyridone (+)-27 :

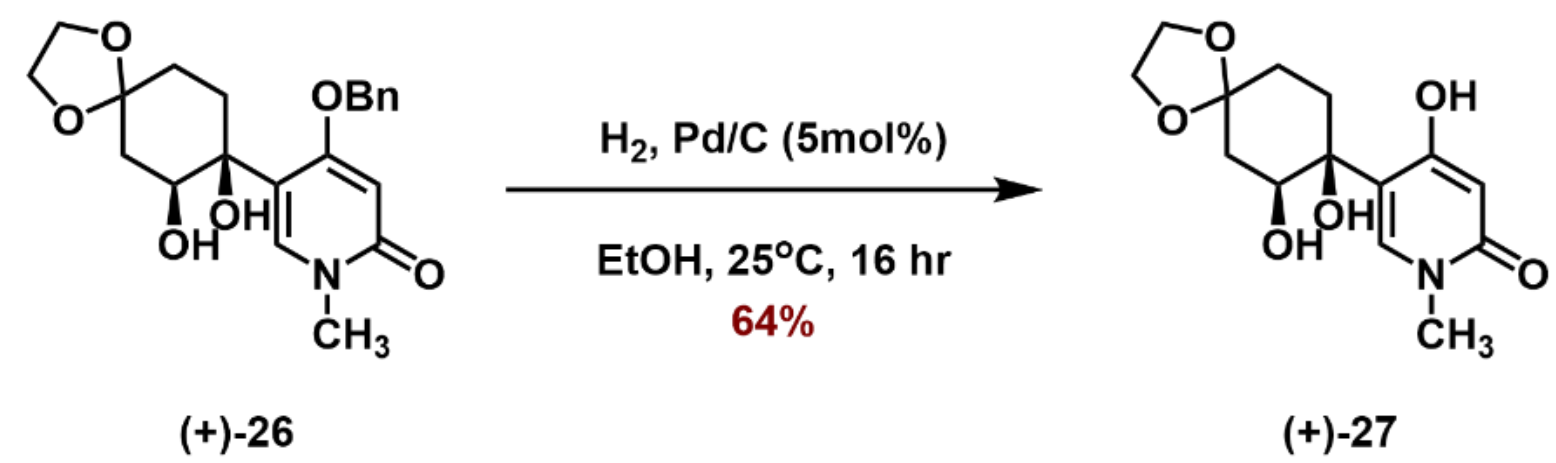

To a flask was added pyridone diol **(+)-26** (1.0 eq, 325.0 mg, 839 µmol) and 5% palladium on activated charcoal (10 mol%, 178.5 mg, 83.9µmol) in ethanol (7 mL). The resulting suspension was sparged five times with hydrogen, then the mixture was left to stir at room temperature for 16 hours under hydrogen atmosphere. The suspension was then filtered through a pad of Celite, washing with methanol. The filtrate obtained was concentrated *in vacuo* to give crude material. Purification by column chromatography (0-30% methanol in dichloromethane) afforded 4-hydroxy-2-pyridone **(+)-27** as a light brown solid in 64% yield (160.0 mg, 538 µmol).

R_f = 0.1 (10% methanol in dichloromethane, visualized by UV and $KMnO_4$)

^{1}H NMR (400 MHz, CD_3OD) δ 7.60 (s, 1H), 4.36 (dd, J = 11.7, 4.9 Hz, 1H), 4.08 – 3.91 (m, 4H), 3.51 (s, 3H), 2.45 (td, J = 14.1, 4.4 Hz, 1H), 2.08 – 1.90 (m, 2H), 1.87 (ddd, J = 12.3, 4.9, 2.6 Hz, 1H), 1.66 (ddd, J = 14.0, 4.5, 2.5 Hz, 1H), 1.57 (ddt, J = 13.0, 4.6, 2.5 Hz, 1H).

^{13}C NMR (101 MHz, CD_3OD) δ 166.63, 164.83, 138.21, 118.07, 108.96, 74.02, 69.36, 64.04, 63.83, 62.99, 38.46, 36.10, 31.30, 29.20.

HRMS (ESI): calculated $C_{14}H_{20}NO_6$ for $[M+H]^+$ 298.1285, found 298.1283.

$[\alpha]_D^{25}$ = +16.7 (*c* = 0.25, MeOH)

Tetrahydropyranylpyridone diol (-)-29 :

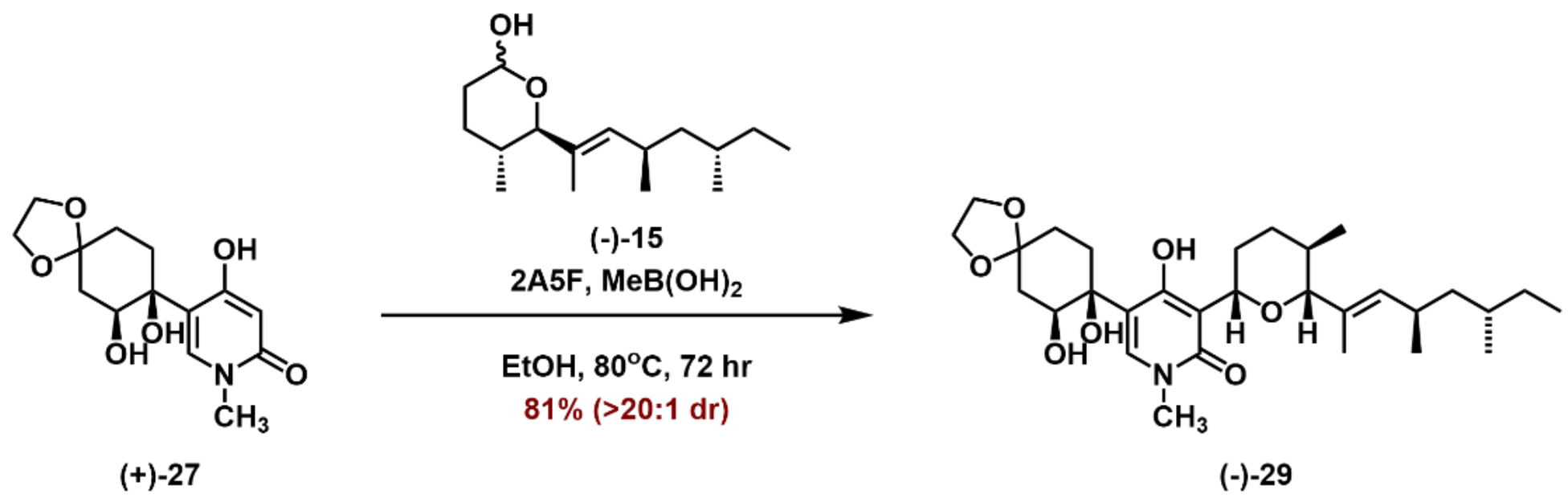

To an oven-dried vial under argon atmosphere was added 4-hydroxy-2-pyridone **(+)-27** (37.98 mg, 127.7 µmol, 1.30 eq), 2-amino-5-fluoropyridine (55.08 mg, 491.3 µmol, 5.00 eq) and methylboronic acid (29.41 mg, 491.3 µmol, 5.00 eq). To this was then added lactol **(-)-15** (25.00 mg, 98.27 µmol, 1.00 eq) in absolute ethanol (2.0 mL), and the resulting solution was left to stir at 80ºC for 48 hours. The crude reaction mixture was then taken up in dichloromethane, washing with 1M aqueous hydrochloric acid. The combined organic layers were washed with saturated sodium chloride, dried over sodium sulfate, then concentrated in vacuo to give crude material. Purification by column chromatography (0-10% methanol in dichloromethane) afforded pyridone diol **(-)-29** as an amorphous white solid in 81% yield (42.5 mg, 79.6 µmol, >20:1 dr).

R_f = 0.45 (10% methanol in dichloromethane, visualized by UV and $KMnO_4$)

^{1}H NMR (400 MHz, $CDCl_3$) δ 10.16 (s, 1H), 7.37 (s, 1H), 5.30 (s, 1H), 5.24 – 5.16 (m, 1H), 4.98 (dd, *J* = 11.3, 2.3 Hz, 1H), 4.46 (q, *J* = 7.5 Hz, 1H), 3.98 (hd, *J* = 5.1, 2.9 Hz, 5H), 3.51 (d, *J* = 10.1 Hz, 1H), 3.46 (s, 3H), 3.28 (s, 1H), 2.50 (p, *J* = 7.8 Hz, 1H), 2.06 – 1.95 (m, 8H), 1.90 (dd, *J* = 13.3, 3.5 Hz, 1H), 1.67 (d, *J* = 1.3 Hz, 3H), 1.47 – 1.38 (m, 1H), 1.36 – 1.17 (m, 2H), 1.10 – 1.00 (m, 2H), 0.93 (d, *J* = 6.6 Hz, 3H), 0.85 (d, *J* = 3.0 Hz, 3H), 0.84 (d, *J* = 2.3 Hz, 3H), 0.82 (s, 1H), 0.75 (d, *J* = 6.6 Hz, 3H).

^{13}C NMR (101 MHz, $CDCl_3$) δ 162.02, 161.30, 137.98, 135.96, 130.21, 114.71, 110.78, 108.92, 92.66, 77.99, 74.00, 69.46, 64.52, 64.37, 44.73, 38.42, 37.31, 32.50, 32.47, 32.11, 31.97, 30.55, 29.81, 29.63, 28.91, 20.73, 19.61, 17.67, 12.02, 11.25.

HRMS (ESI): calculated $C_{30}H_{48}O_7N$ for $[M+H]^+$ 534.3425, found 534.3427.

$[\alpha]_D^{25}$ = -65.1 (*c* = 0.18, $CHCl_3$)

(-)-oxysporidinone (7) :

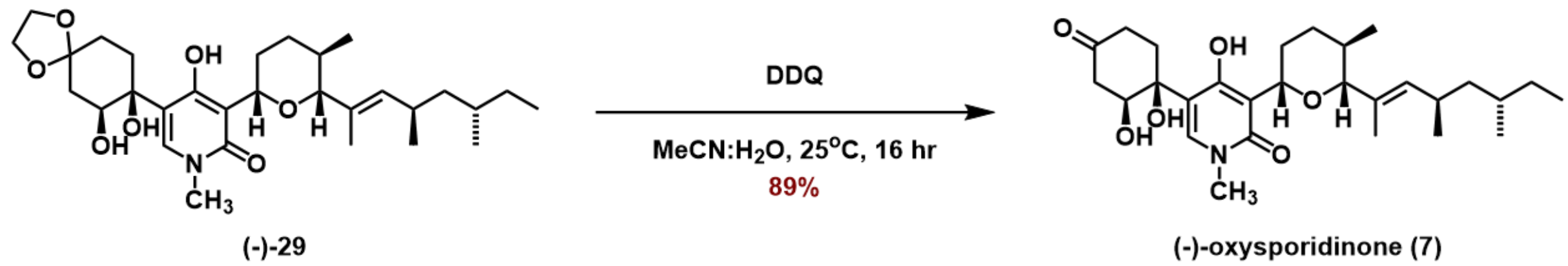

To a vial was added pyridone diol **(-)-29** (2.20 mg, 4.122 µmol, 1.00 eq) in a 9:1 mixture of acetonitrile and water (222 µL). To this was then added 2,3-dichloro-5,6-dicyano-1,4-benzoquinone (4.679 mg, 20.61 µmol, 5.00 eq) and the resulting dark red solution was left to stir at room temperature for 16 hours. The reaction mixture was then concentrated *in vacuo* then diluted in dichloromethane, washing with water three times. The combined organic layers were washed with saturated sodium chloride then dried over sodium sulfate and concentrated *in vacuo* to give crude material. Purification by column chromatography (0-10% methanol in dichloromethane) afforded (-)-oxysporidinone (**7**) as an amorphous white solid in 89% yield (1.8 mg, 3.7 µmol).

$\mathbf{R_f}$ = 0.45 (10% methanol in dichloromethane, visualized by UV and $KMnO_4$)

^{1}**H NMR** (800 MHz, $CDCl_3$) δ 10.25 (s, 1H), 7.44 (s, 1H), 5.22 (dd, *J* = 9.6, 1.6 Hz, 1H), 4.97 (dd, *J* = 11.3, 2.4 Hz, 1H), 4.67 (dt, *J* = 11.2, 5.6 Hz, 1H), 3.51 (d, *J* = 10.1 Hz, 1H), 3.49 (s, 3H), 3.27 (d, *J* = 1.7 Hz, 1H), 2.79 (td, *J* = 14.1, 6.7 Hz, 1H), 2.74 (dd, *J* = 14.0, 11.3 Hz, 1H), 2.65 (ddd, *J* = 14.0, 5.7, 2.2 Hz, 1H), 2.54 – 2.47 (m, 1H), 2.32 (tdd, *J* = 14.1, 5.0, 2.1 Hz, 1H), 2.25 (ddt, *J* = 14.4, 4.7, 2.2 Hz, 1H), 2.14 (ddd, *J* = 14.5, 6.7, 2.2 Hz, 1H), 2.07 – 2.05 (m, 1H), 2.04 – 1.93 (m, 0H), 1.91 (dq, *J* = 13.3, 3.4 Hz, 1H), 1.74 – 1.67 (m, 1H), 1.65 – 1.62 (m, 1H), 1.59 – 1.56 (m, 1H), 1.45 – 1.39 (m, 1H), 1.38 – 1.33 (m, 1H), 1.33 – 1.28 (m, 1H), 1.23 (ddd, *J* = 13.8, 7.6, 6.4 Hz, 1H), 1.06 (dtd, *J* = 13.4, 8.0, 2.0 Hz, 2H), 0.93 (d, *J* = 6.6 Hz, 3H), 0.85 (d, *J* = 3.0 Hz, 3H), 0.84 (d, *J* = 2.3 Hz, 3H), 0.76 (d, *J* = 6.6 Hz, 3H).

^{13}**C NMR** (201 MHz, $CDCl_3$) δ 208.84, 161.37, 161.29, 138.21, 136.21, 130.06, 113.77, 110.82, 92.64, 78.04, 73.74, 70.34, 45.31, 44.73, 37.33, 36.40, 32.43, 32.00, 30.56, 29.65, 28.90, 20.71, 19.61, 17.63, 11.80, 11.23.

HRMS (ESI): calculated $C_{28}H_{44}O_6N$ for $[M+H]^+$ 490.3163, found 490.3169.

$[\alpha]_D^{25}$ = -15.1 (c = 0.16, $CHCl_3$) ; $[\alpha]_D^{25}$ = -16.1 (c = 0.17, EtOH)

(+)-fusoxypyridone B (8) :

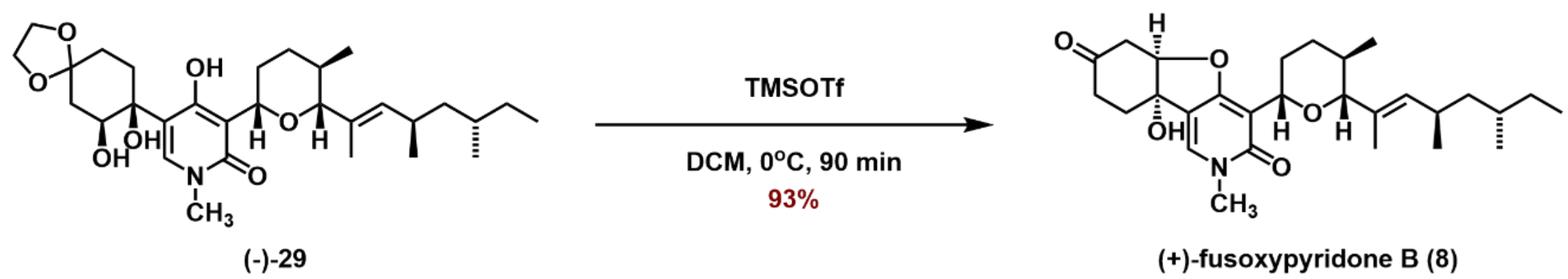

To a flame-dried flask under argon atmosphere was added pyridone diol **(-)-29** (11 mg, 21 µmol, 1.00 eq) in anhydrous dichloromethane (2.0 mL) at 0ºC. To this was added trimethylsilyl trifluoromethanesulfonate (9.2 mg, 7.5 µL, 41 µmol, 2.00 eq) dropwise, and the resulting mixture was stirred for 90 minutes at 0°C. The reaction was quenched by addition of saturated aqueous sodium bicarbonate, extracting with additional dichloromethane. The combined organic layers were washed with brine, dried over sodium sulfate, and concentrated in vacuo to give crude material. Purification by column chromatography (0-5% methanol in dichloromethane) afforded (+)-fusoxypyridone B (**8**) as a white solid in 93% yield (9 mg, 19 µmol).

R_f = 0.5 (7% methanol in ethyl acetate, visualized by *p*-anisaldehyde and CAM)

^{1}H NMR (400 MHz, CD_3OD) δ 7.66 (s, 1H), 5.12 (dd, J = 9.6, 1.6 Hz, 1H), 4.93 (t, J = 4.2 Hz, 1H), 4.72 (dd, J = 11.7, 2.3 Hz, 1H), 3.47 (s, 3H), 3.37 (d, J = 9.9 Hz, 1H), 3.06 (dd, J = 16.4, 4.4 Hz, 1H), 2.73 (dd, J = 16.4, 3.9 Hz, 1H), 2.54 – 2.43 (m, 1H), 2.42 – 2.36 (m, 1H), 2.34 (t, J = 4.2 Hz, 1H), 2.28 – 2.17 (m, 1H), 2.17 – 2.09 (m, 1H), 2.09 – 1.96 (m, 1H), 1.92 – 1.86 (m, 1H), 1.59 (d, J = 1.4 Hz, 5H), 1.50 – 1.35 (m, 1H), 1.35 – 1.28 (m, 1H), 1.19 (ddd, J = 13.3, 7.5, 5.8 Hz, 1H), 1.10 – 0.97 (m, 2H), 0.87 (d, J = 6.6 Hz, 3H), 0.85 – 0.80 (m, 6H), 0.70 (d, J = 6.6 Hz, 3H).

^{13}C NMR (101 MHz, CD_3OD) δ 210.36, 168.28, 165.25, 137.67, 135.98, 133.59, 118.46, 108.96, 93.28, 92.36, 77.04, 73.89, 49.07, 46.16, 38.49, 35.86, 34.02, 33.84, 33.29, 32.93, 30.73, 30.20, 29.81, 21.18, 20.10, 18.22, 11.57.

^{1}H NMR (400 MHz, $CDCl_3$) δ 7.27 (s, 1H), 5.15 (d, J = 9.4 Hz, 1H), 4.94 (t, J = 4.5 Hz, 1H), 4.77 (dd, J = 11.7, 2.3 Hz, 1H), 3.43 (s, 3H), 3.41 – 3.38 (m, 1H), 3.07 (s, 1H), 2.94 (dd, J = 16.5, 4.6 Hz, 1H), 2.87 – 2.79 (m, 1H), 2.51 – 2.31 (m, 3H), 2.28 – 2.16 (m, 1H), 2.08 – 1.90 (m, 2H), 1.84 (m, 1H), 1.67 (m, 1H), 1.64 (s, 3H), 1.49 (m, 1H), 1.40-1.22 (m, 3H), 1.17 (m, 1H), 1.12 – 0.96 (m, 2H), 0.87 (d, J = 6.6 Hz, 3H), 0.82 (d, J = 6.7 Hz, 6H), 0.70 (d, J = 6.6 Hz, 3H).

^{13}C NMR (100 MHz, $CDCl_3$) δ 207.43, 165.72, 163.24, 136.74, 133.31, 132.23, 115.69, 109.12, 91.66, 90.37, 76.46, 72.33, 44.84, 42.05, 38.15, 34.99, 32.88, 32.82, 31.86, 31.66, 29.47, 29.28, 29.05, 20.70, 19.56, 17.79, 11.36, 11.29.

HRMS (ESI): calculated for $C_{28}H_{42}NO_5$ $[M+H]^+$ 472.3058, found 472.3057; calculated for $C_{28}H_{41}NO_5Na$ $[M+Na]^+$ 494.2877, found 494.2875

$[\alpha]_D^{25}$ = +8.0 (c = 0.2, MeOH) ; **$[\alpha]_D^{25}$** +18.6 (c = 0.2, $CHCl_3$)

Pyridone diol (-)-26 :

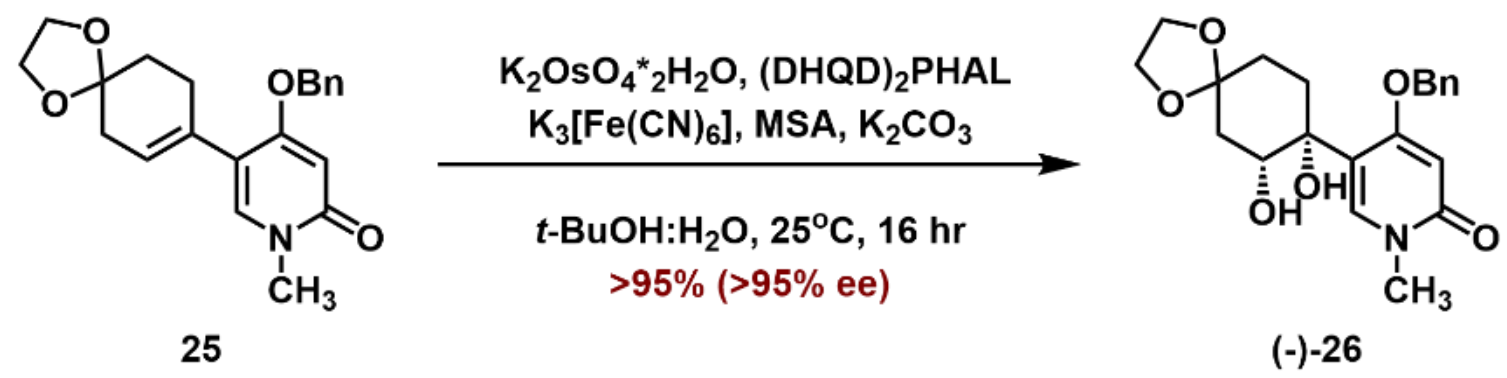

To a flask was added potassium carbonate (384 mg, 2.78 mmol, 3.00 eq), potassium ferricyanide (588 mg, 2.78 mmol, 3.00 eq), methanesulfonamide (106 mg, 1.11 mmol, 1.20 eq), $(DHQD)_2PHAL$ (72 mg, 92.5 µmol, 10 mol%) and potassium osmate(VI) dihydrate (17 mg, 46.3 µmol, 5 mol%) in a 1:1 mixture of tert-butanol and water (26 mL). This slurry was left to stir vigorously at room temperature for 15 minutes prior to the addition of pyridone (327 mg, 925 µmol, 1.00 eq), after which the reaction mixture was left to continue stirring at that temperature for 16 hours. Sodium sulfite (117 mg, 925 µmol, 1.00 eq) was added to quench the reaction mixture, which was then taken up in deionized water, extracting with a 4:1 mixture of chloroform : isopropanol three times. The combined organic layers were washed with brine, dried over sodium sulfate, and concentrated in vacuo to give crude material. Purification by column chromatography (0-10% methanol in dichloromethane) gave pyridone diol **(-)-26** as a wispy white solid in 99% yield (358 mg, 924 µmol, >95% ee).

R_f = 0.25 (10% methanol in dichloromethane, visualized by UV and $KMnO_4$)

^{1}H NMR (400 MHz, CD_3OD) δ 7.74 (s, 1H), 7.60 – 7.51 (m, 2H), 7.49 – 7.32 (m, 3H), 6.10 (s, 1H), 5.14 (s, 2H), 4.52 (dd, J = 11.6, 4.9 Hz, 1H), 4.02 – 3.88 (m, 2H), 3.92 – 3.77 (m, 2H), 3.55 (s, 3H), 2.61 (td, J = 14.4, 4.8 Hz, 1H), 2.02 – 1.88 (m, 2H), 1.82 (ddd, J = 12.3, 5.0, 2.5 Hz, 1H), 1.61 – 1.47 (m, 2H).

^{13}C NMR (101 MHz, CD_3OD) δ 165.94, 165.03, 138.21, 135.29, 128.16, 127.76, 127.46, 118.57, 108.88, 96.98, 73.75, 70.52, 68.85, 64.01, 63.80, 38.35, 36.06, 31.33, 29.23.

HRMS (ESI): calculated $C_{21}H_{26}NO_6$ for $[M+H]^+$ 388.1755, found 388.1756.

HPLC (Chiralpak IA, 25% isopropanol in hexanes, 1.0 mL/min) λ = 254 nm, t_R = 16.155 min, >95% ee

4-hydroxy-2-pyridone (-)-27 :

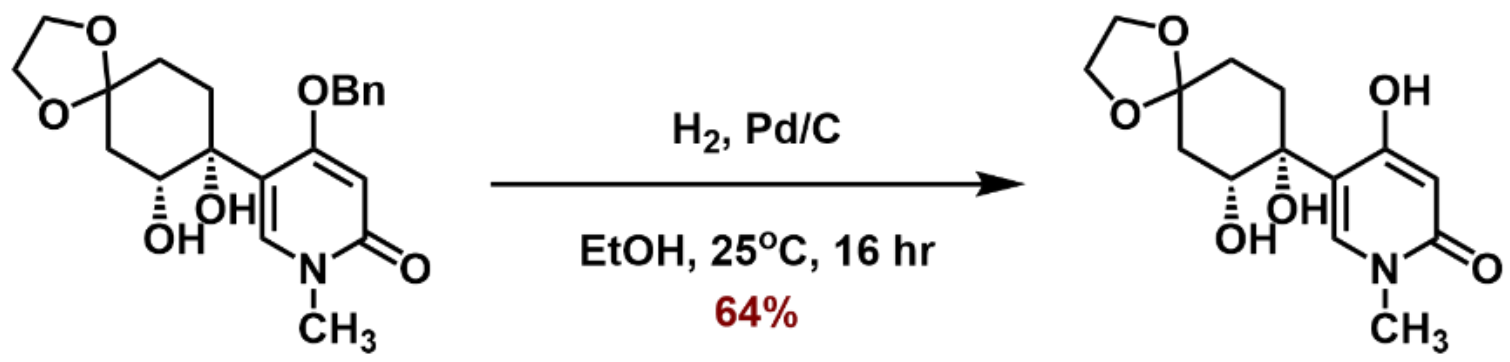

(-)-26 (-)-27

To a flask was added pyridone diol **(-)-26** (1.0 eq, 325.0 mg, 839 µmol) and 5% palladium on activated charcoal (10 mol%, 178.5 mg, 83.9µmol) in ethanol (7 mL). The resulting suspension was sparged five times with hydrogen, then the mixture was left to stir at room temperature for 16 hours under hydrogen atmosphere. The suspension was then filtered through a pad of Celite, washing with methanol. The filtrate obtained was concentrated *in vacuo* to give crude material. Purification by column chromatography (0-30% methanol in dichloromethane) afforded 4-hydroxy-2-pyridone **(-)-27** as a light brown solid in 64% yield (160.0 mg, 538 µmol).

R_f = 0.1 (10% methanol in dichloromethane, visualized by UV and $KMnO_4$)

^{1}H NMR (400 MHz, CD_3OD) δ 7.60 (s, 1H), 4.36 (dd, J = 11.7, 4.9 Hz, 1H), 4.08 – 3.91 (m, 4H), 3.51 (s, 3H), 2.45 (td, J = 14.1, 4.4 Hz, 1H), 2.08 – 1.90 (m, 2H), 1.87 (ddd, J = 12.3, 4.9, 2.6 Hz, 1H), 1.66 (ddd, J = 14.0, 4.5, 2.5 Hz, 1H), 1.57 (ddt, J = 13.0, 4.6, 2.5 Hz, 1H).

^{13}C NMR (101 MHz, CD_3OD) δ 108.98, 74.03, 63.81, 56.92, 38.47, 36.01, 31.44, 29.20, 16.97.

HRMS (ESI): calculated $C_{14}H_{20}NO_6$ for $[M+H]^+$ 298.1285, found 298.1280.

(-)-fusoxypyridone C (31) :

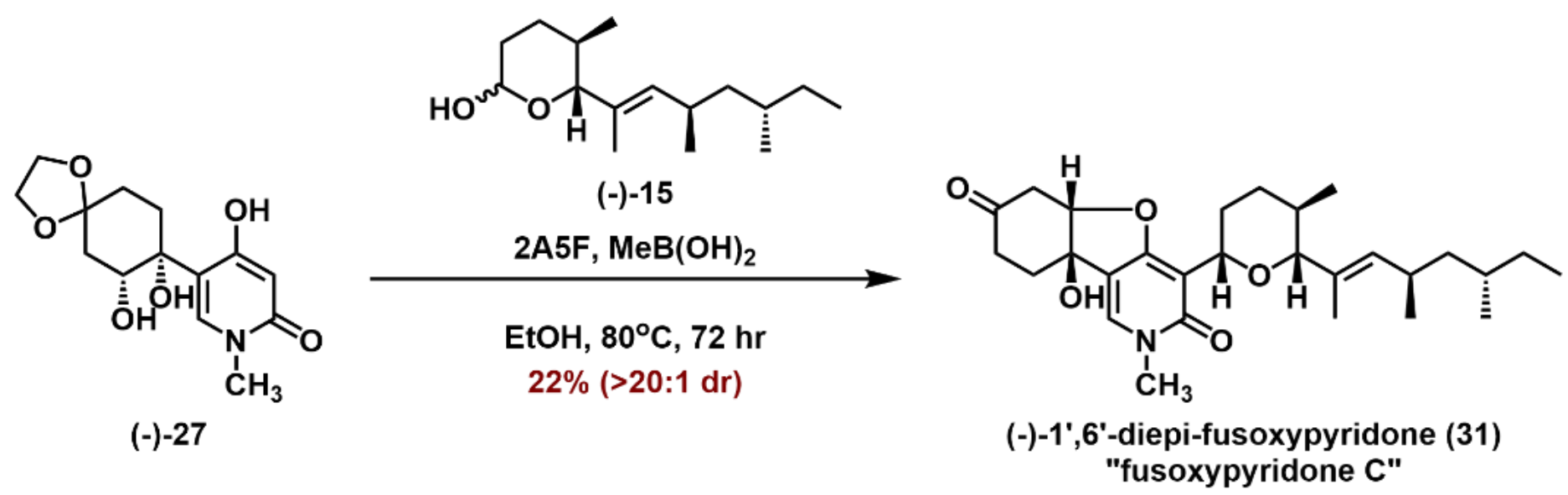

To an oven-dried vial under argon atmosphere was added 4-hydroxy-2-pyridone **(-)-27** (22.8 mg, 76.7 µmol, 1.30 eq), 2-amino-5-fluoropyridine (33.0 mg, 295.0 µmol, 5.00 eq) and methylboronic acid (17.6 mg, 295.0 µmol, 5.00 eq). To this was then added lactol **(-)-15** (15.0 mg, 59.0 µmol, 1.00 eq) in absolute ethanol (2.0 mL), and the resulting solution was left to stir at 80ºC for 48 hours. The crude reaction mixture was then taken up in dichloromethane, washing with 1M aqueous hydrochloric acid. The combined organic layers were washed with saturated sodium chloride, dried over sodium sulfate, then concentrated in vacuo to give crude material. Purification by column chromatography (0-10% methanol in dichloromethane) afforded (-)-fusoxypyridone C (**32**) as an amorphous white solid in 22% yield (6.0 mg, 13 µmol).

R$_f$ = 0.5 (7% methanol in ethyl acetate, visualized by *p*-anisaldehyde and CAM)

^{1}H NMR (400 MHz, CD_3OD) δ 7.66 (s, 1H), 5.07 (d, *J* = 9.9 Hz, 1H), 4.73 (dd, *J* = 11.7, 2.4 Hz, 1H), 3.47 (s, 3H), 3.35 – 3.30 (m, 1H), 3.00 (dd, *J* = 16.8, 4.4 Hz, 1H), 2.75 (dd, *J* = 16.8, 4.2 Hz, 1H), 2.47 (p, *J* = 8.3 Hz, 1H), 2.38 – 2.27 (m, 2H), 2.27 – 2.08 (m, 2H), 2.05 – 1.93 (m, 1H), 1.93 – 1.80 (m, 1H), 1.61 (d, *J* = 12.2 Hz, 2H), 1.53 (d, *J* = 1.3 Hz, 3H), 1.44 – 1.27 (m, 1H), 1.18 (dt, *J* = 13.7, 6.8 Hz, 1H), 1.02 (dq, *J* = 14.1, 7.2 Hz, 2H), 0.88 – 0.81 (m, 9H), 0.70 (d, *J* = 6.6 Hz, 3H).

^{13}C NMR (101 MHz, CD_3OD) δ 208.81, 167.02, 135.87, 134.39, 132.41, 117.59, 107.87, 91.64, 90.54, 75.63, 72.10, 44.81, 41.23, 37.10, 34.39, 32.87, 32.55, 31.86, 31.48, 29.37, 29.27, 28.74, 28.46, 19.77, 18.69, 16.87, 10.19, 10.11.

HRMS (ESI): calculated $C_{28}H_{42}NO_5$ $[M+H]^+$ 472.3058, found 472.30614

$[\alpha]_D^{23}$ = -24.7 (*c* = 0.2, MeOH)

(-)-fusapyridone A (32):

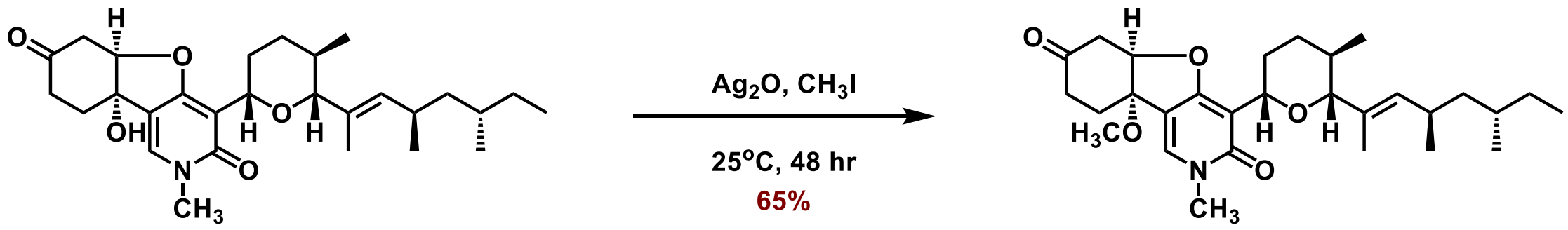

(+)-fusoxypyridone B (8) (-)-fusapyridone A (31)

To a flame-dried flask under argon atmosphere was added (+)-fusoxypyridone B (**8**) (9 mg, 19 µmol, 1.0 eq) and silver(I) oxide (22 mg, 95 µmol, 5.0 eq) in iodomethane (1.0 mL) at room temperature. The reaction mixture was left to stir in the dark for 48 hours, then filtered through a pad of Celite and washed with methanol. The filtrate was concentrated *in vacuo*, and the resulting crude residue was purified by column chromatography (0-5% methanol in ethyl acetate) to give (-)-fusapyridone A (**31**) as a white solid in 65% yield (6 mg, 12 µmol).

R_f = 0.6 (7% methanol in ethyl acetate, visualized by *p*-anisaldehyde and $KMnO_4$)

^{1}H NMR (400 MHz, $CDCl_3$) δ 7.19 (s, 1H), 5.12 (d, J = 8.0 Hz, 1H), 5.03 (t, J = 4.6 Hz, 1H), 4.78 (dd, J = 11.7, 2.3 Hz, 1H), 3.48 (s, 3H), 3.39 (d, J = 9.7 Hz, 1H), 3.21 (s, 3H), 2.86 (t, J = 4.3 Hz, 2H), 2.49-2.44 (m, 1H), 2.40 (m, 1H), 2.39-2.35 (m, 1H), 2.16 – 2.12 (m, 1H), 2.09 – 2.03 (m, 1H), 1.97-2.03 (m, 1H), 1.85 (dd, J = 12.9, 3.4 Hz, 1H), 1.63 (s, 3H), 1.60-1.50 (m, 2H), 1.38-1.28 (m, 4H), 1.17 (m, 1H), 1.03 (m, 1H), 0.89 (d, J = 6.5 Hz, 3H), 0.85 (m, 3H), 0.83 (m, 3H), 0.70 (d, J = 6.7 Hz, 3H). 190

^{13}C NMR (101 MHz, $CDCl_3$) δ 207.5, 166.3, 163.3, 136.1, 133.2, 132.3, 110.9, 109.7, 91.3, 84.1, 82.2, 72.1, 51.2, 44.9, 42.2, 38.3, 34.9, 32.9, 32.33, 31.8, 31.8, 29.7, 29.4, 29.2, 20.6, 19.5, 17.9, 11.3, 11.2.

HRMS (ESI): calculated for $C_{29}H_{44}NO_5$ $[M+H]^+$ 486.3214, found 486.3218

$[\alpha]_D^{25}$ = -21.6 (c = 0.25, MeOH)

Section 4. References

(1) Kolb, H. C.; Vannieuwenhze, M. S.; Sharpless, K. B. Catalytic Asymmetric Di Hydroxylation. *Chem. Rev* **1994**, *94*, 2483–2547.

(2) Yang, W.; Tan, Q.; Yin, Y.; Chen, Y.; Zhang, Y.; Wu, J.; Gao, L.; Wang, B.; She, Z. Secondary Metabolites with α-Glucosidase Inhibitory Activity from Mangrove Endophytic Fungus Talaromyces Sp. Cy-3. *Mar. Drugs* **2021**, *19* (9), 492.

(3) Kim, J. C.; Lee, Y. W.; Tamura, H.; Yoshizawa, T. Sambutoxin: A New Mycotoxin Isolated from Fusarium Sambucinum. *Tetrahedron Lett.* **1995**, *36* (7), 1047–1050.

(4) Li, D.; Wang, W.; Xu, K.; Li, J.; Long, B.; Li, Z.; Tan, G.; Yu, X. Elucidation of a Dearomatization Route in the Biosynthesis of Oxysporidinone Involving a TenA-like Cytochrome P450 Enzyme. *Angew. Chem. Int. Ed.* **2023**, *62* (25), e202301976.

(5) Zhan, J.; Burns, A. M.; Liu, M. X.; Faeth, S. H.; Gunatilaka, A. A. L. Search for Cell Motility and Angiogenesis Inhibitors with Potential Anticancer Activity: Beauvericin and Other Constituents of Two Endophytic Strains of Fusarium Oxysporum1. *J. Nat. Prod.* **2007**, *70* (2), 227–232.

(6) Tsuchinari, M.; Shimanuki, K.; Hiramatsu, F.; Murayama, T.; Koseki, T.; Shiono, Y. Fusapyridons A and B, Novel Pyridone Alkaloids from an Endophytic Fungus, Fusarium Sp. YG-45. *Zeitschrift fur Naturforschung - Section B Journal of Chemical Sciences* **2007**, *62* (9), 1203–1207.

(7) Preindl, J.; Schulthoff, S.; Wirtz, C.; Lingnau, J.; Fürstner, A. Polyunsaturated C-Glycosidic 4-Hydroxy-2-Pyrone Derivatives: Total Synthesis Shows That Putative Orevactaene Is Likely Identical with Epipyrone A. *Angew. Chem. Int. Ed.* **2017**, *56* (26), 7525–7530.

(8) Roush, W. R.; Walts, A. E.; Hoong, L. K. Diastereo- and Enantioselective Aldehyde Addition Reactions of 2-Allyl-1,3,2-Dioxaborolane-4,5-Dicarboxylic Esters, a Useful Class of Tartrate Ester Modified Allylboronates. *J. Am. Chem. Soc.* **2002**, *107* (26), 8186–8190.

Section 5. ^{1}H and ^{13}C NMR Spectra

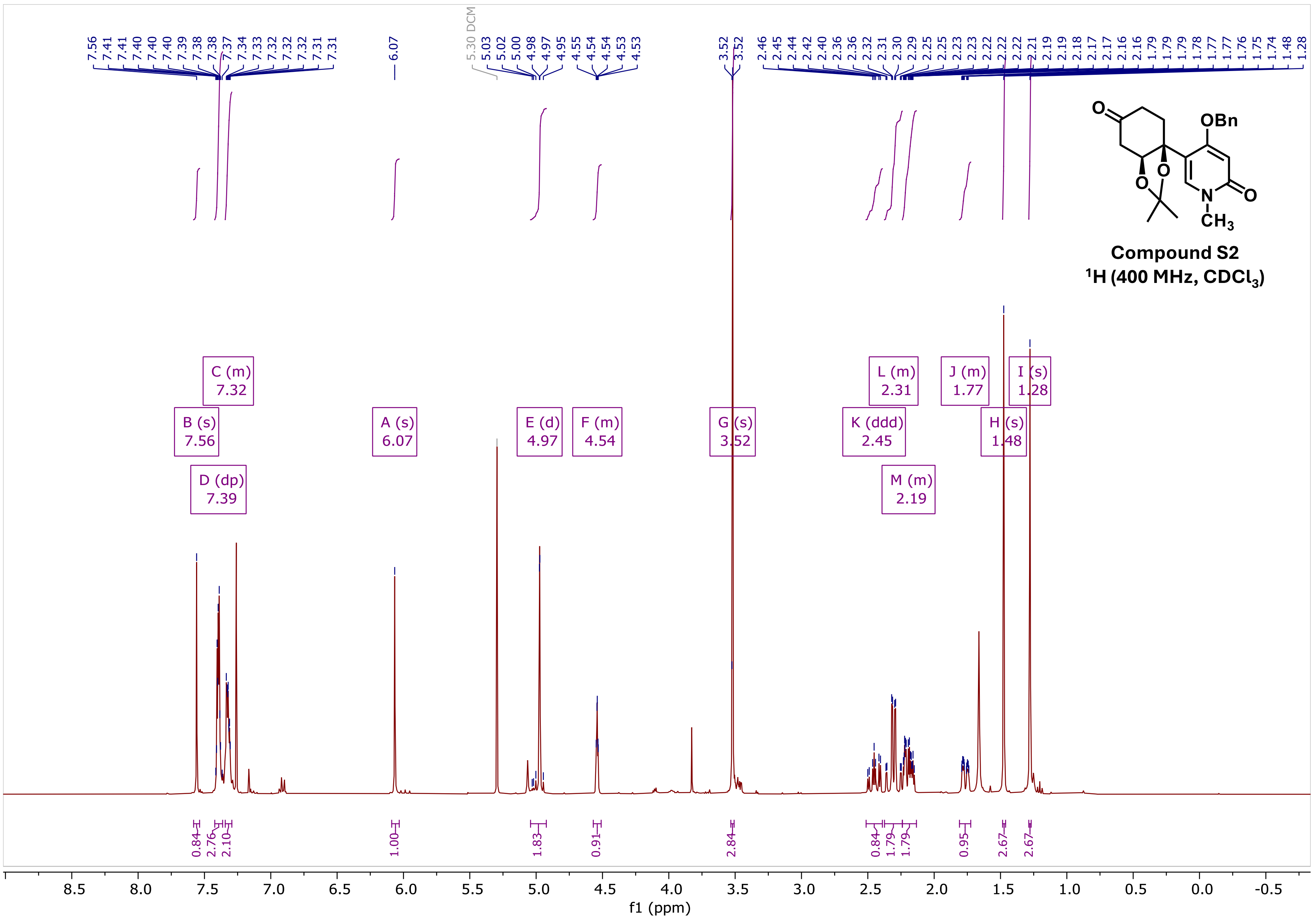

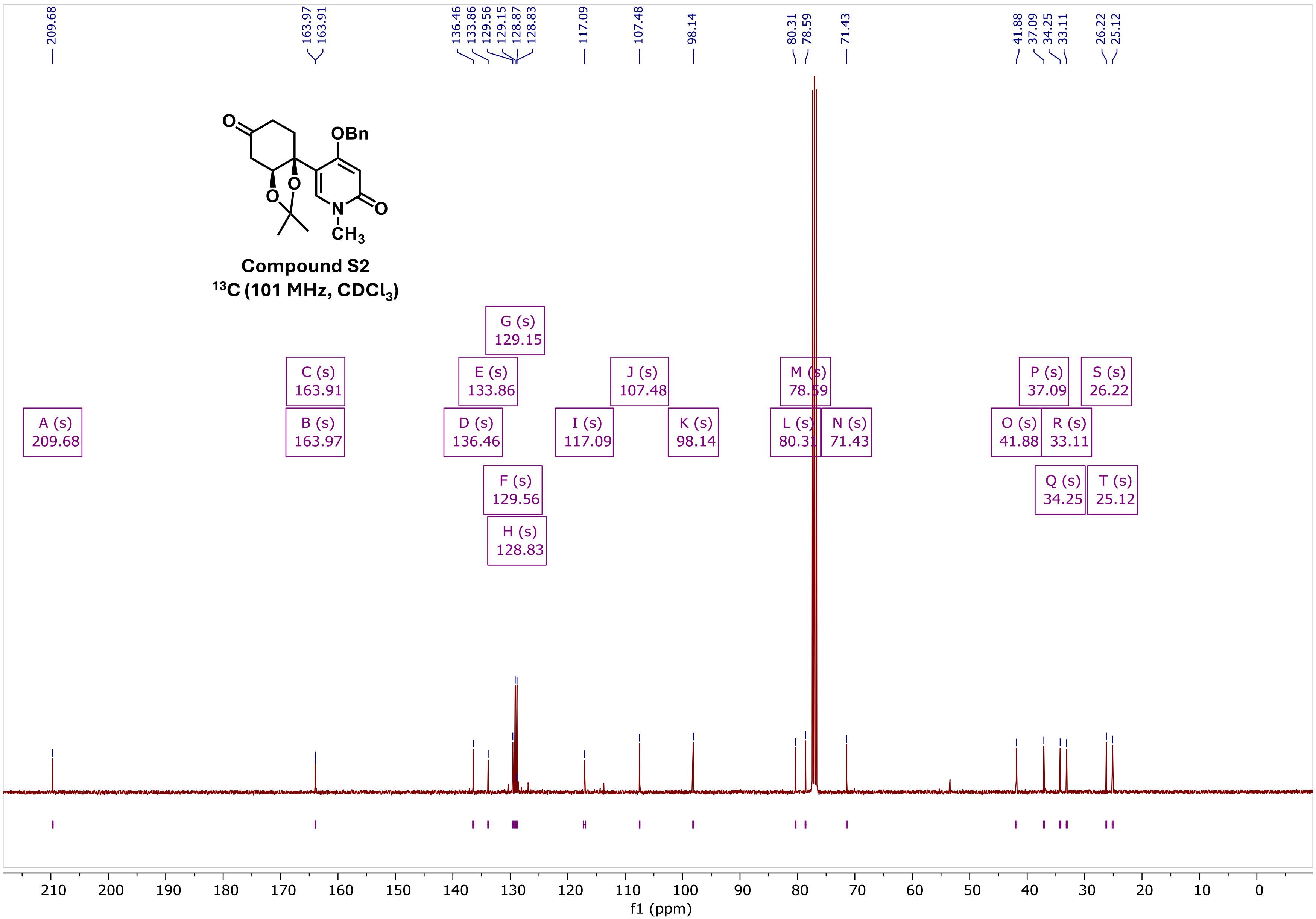
209.68
163.97
163.91
136.46
133.86
129.56
129.15
128.87
128.83
117.09
107.48
98.14
80.31
78.59
71.43
41.88
37.09
34.25
33.11
26.22
25.12
OBn
Compound S2
13C (101 MHz, CDCl3)
A (s) 209.68
B (s) 163.97
C (s) 163.91
D (s) 136.46
E (s) 133.86
F (s) 129.56
G (s) 129.15
H (s) 128.83
I (s) 117.09
J (s) 107.48
K (s) 98.14
N (s) 71.43
O (s) 41.88
P (s) 37.09
Q (s) 34.25
R (s) 33.11
S (s) 26.22
T (s) 25.12
f1 (ppm)

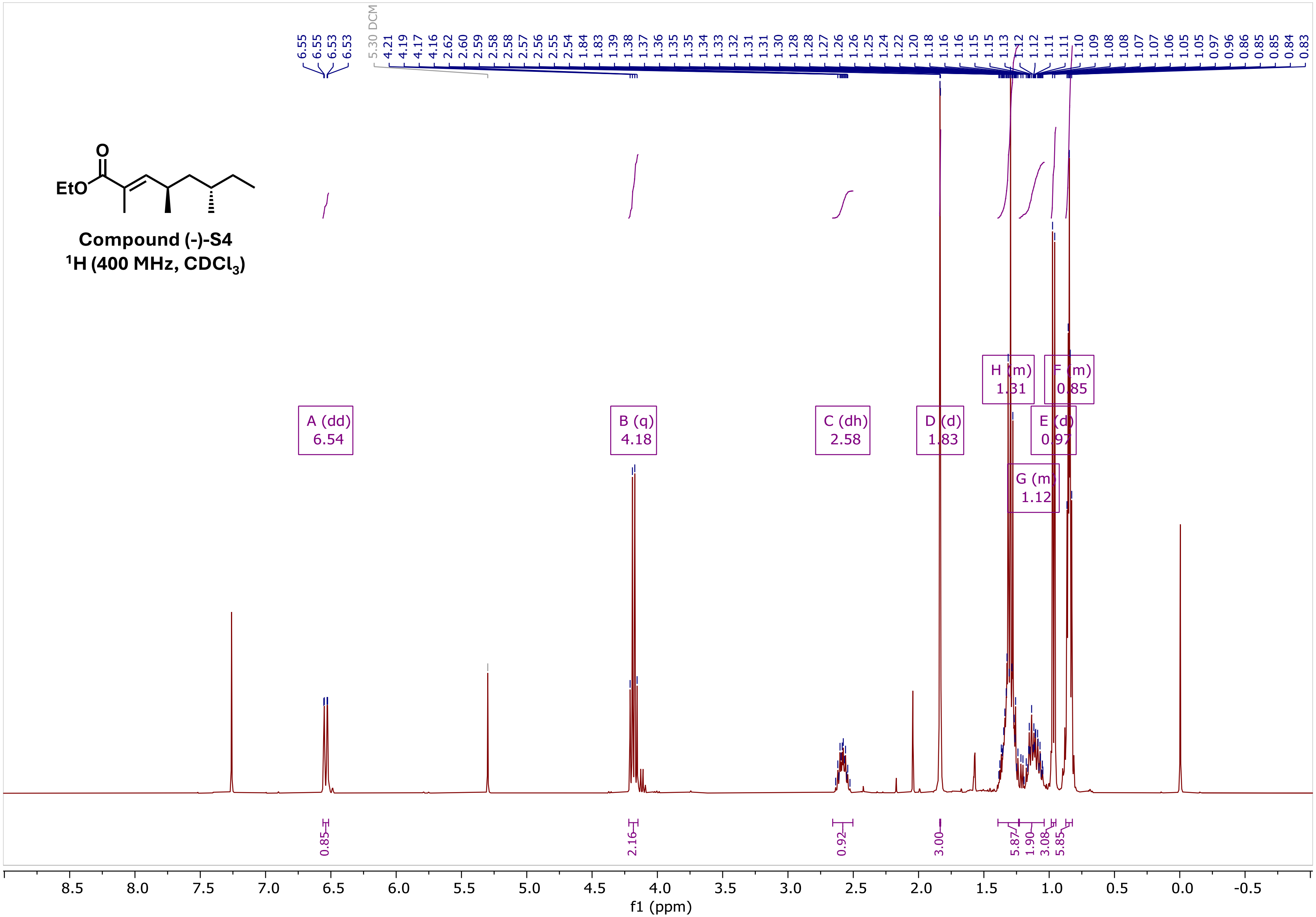
EtO
O
Compound (-)-S4
1H (400 MHz, CDCl3)
A (dd) 6.54
B (q) 4.18
C (dh) 2.58
D (d) 1.83
H (m) 1.31
G (m) 1.12
E (d) 0.97
F (m) 0.85
0.85
2.16
0.92
3.00
5.87
1.90
3.08
5.85
5.30 DCM
f1 (ppm)

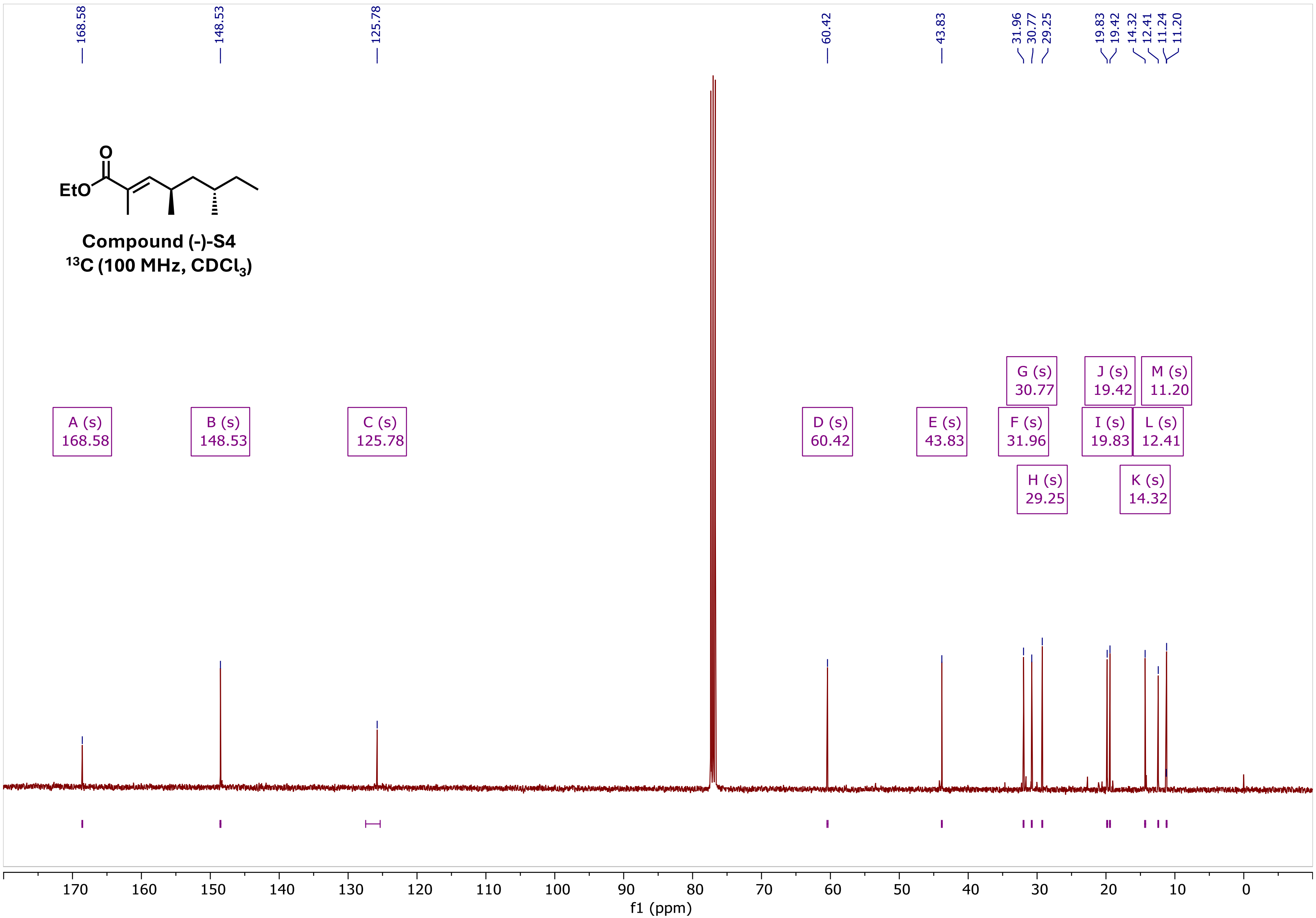

168.58
148.53
125.78
60.42
43.83
31.96
30.77
29.25
19.83
19.42
14.32
12.41
11.24
11.20
EtO
O
Compound (-)-S4
^{13}C (100 MHz, $CDCl_3$)
A (s) 168.58
B (s) 148.53
C (s) 125.78
D (s) 60.42
E (s) 43.83
F (s) 31.96
G (s) 30.77
H (s) 29.25
I (s) 19.83
J (s) 19.42
K (s) 14.32
L (s) 12.41
M (s) 11.20
170 160 150 140 130 120 110 100 90 80 70 60 50 40 30 20 10 0
f1 (ppm)

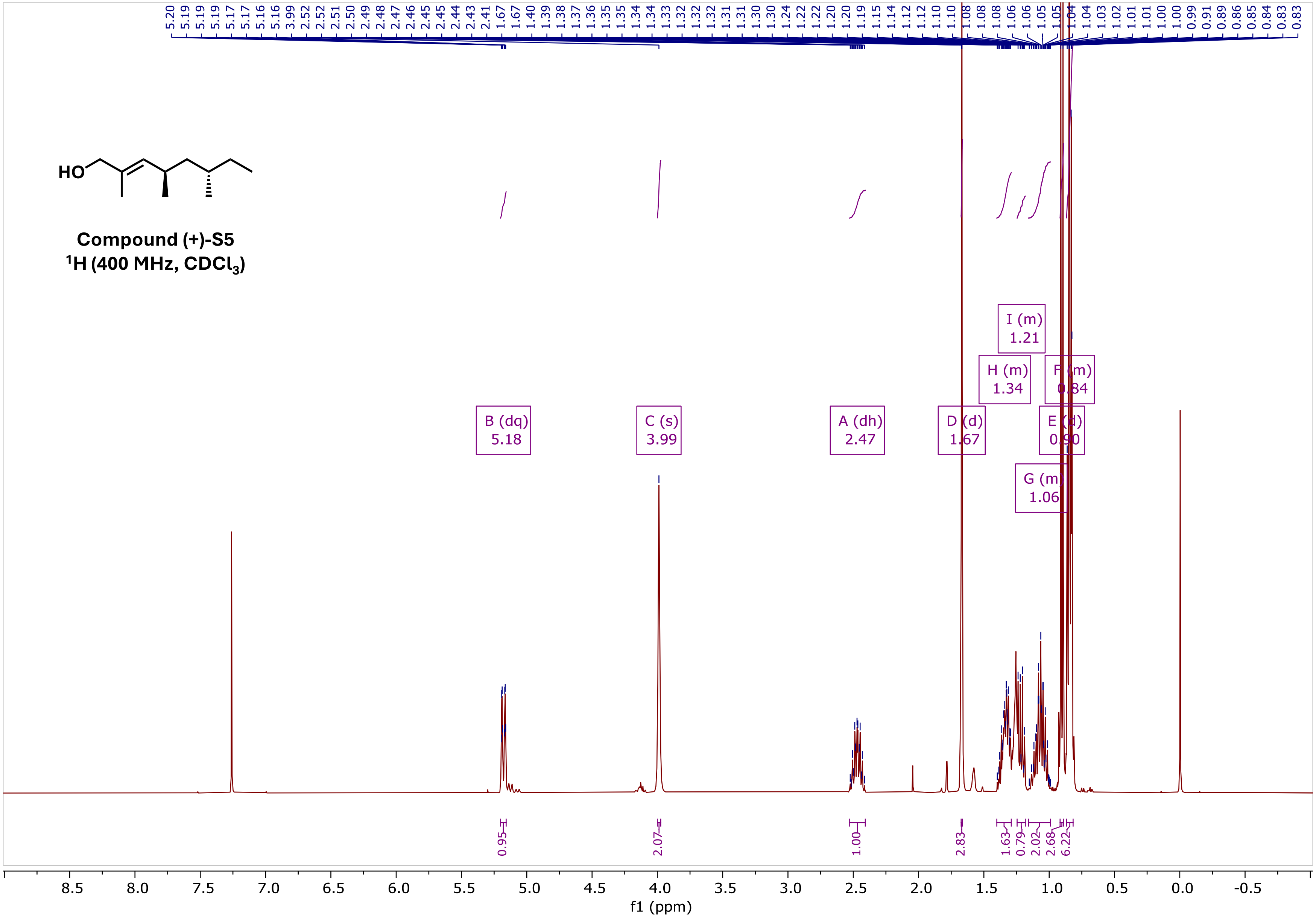

HO
Compound (+)-S5
1H (400 MHz, CDCl3)
B (dq) 5.18
C (s) 3.99
A (dh) 2.47
D (d) 1.67
H (m) 1.34
I (m) 1.21
G (m) 1.06
E (d) 0.90
F (m) 0.84
0.95
2.07
1.00
2.83
1.63
0.79
2.02
2.68
6.22
f1 (ppm)

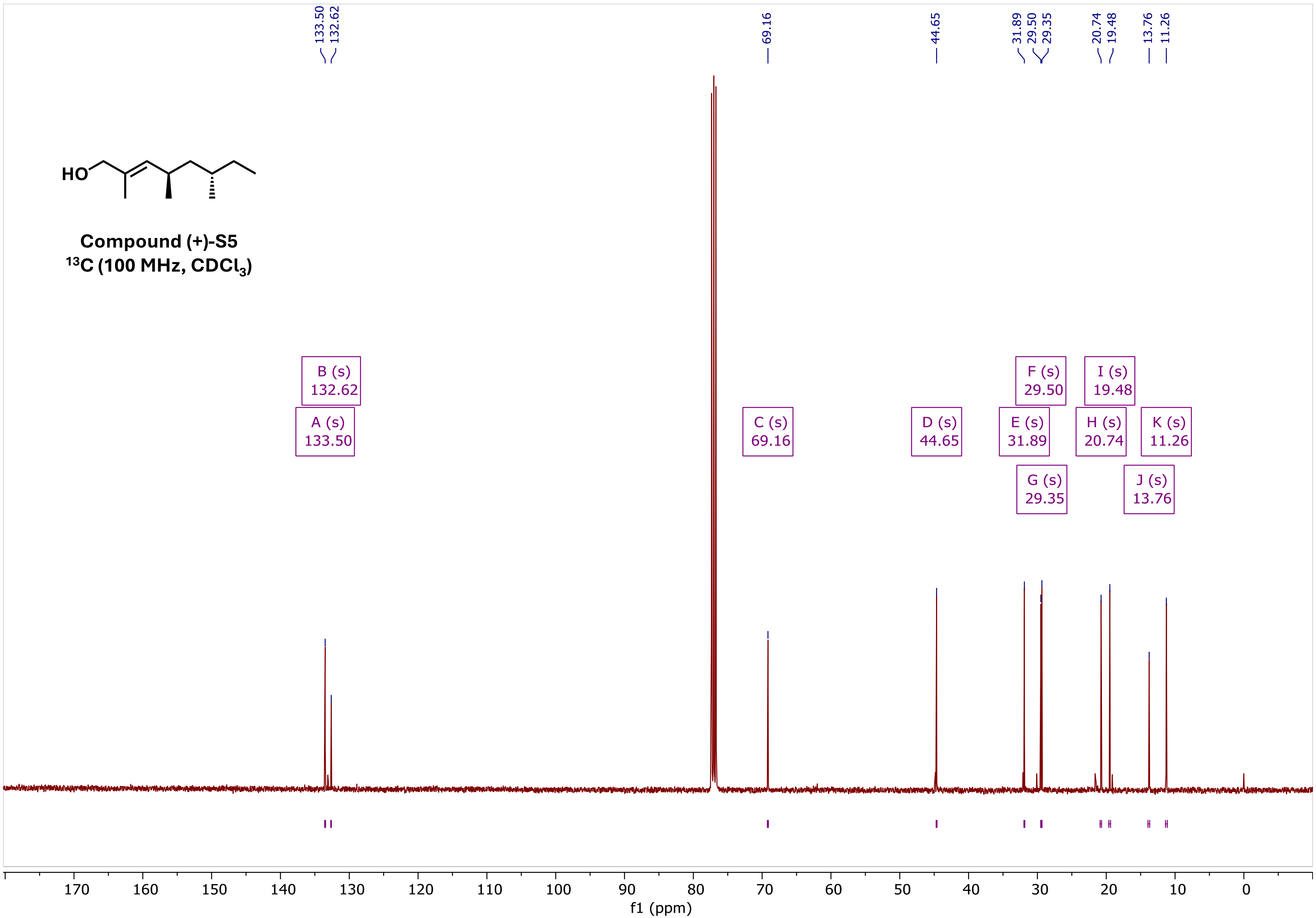

HO
Compound (+)-S5
13C (100 MHz, CDCl3)
133.50
132.62
69.16
44.65
31.89
29.50
29.35
20.74
19.48
13.76
11.26
B (s)
132.62
A (s)
133.50
C (s)
69.16
D (s)
44.65
F (s)
29.50
E (s)
31.89
G (s)
29.35
I (s)
19.48
H (s)
20.74
J (s)
13.76
K (s)
11.26
170
160
150
140
130
120
110
100
90
80
70
60
50
40
30
20
10
0
f1 (ppm)

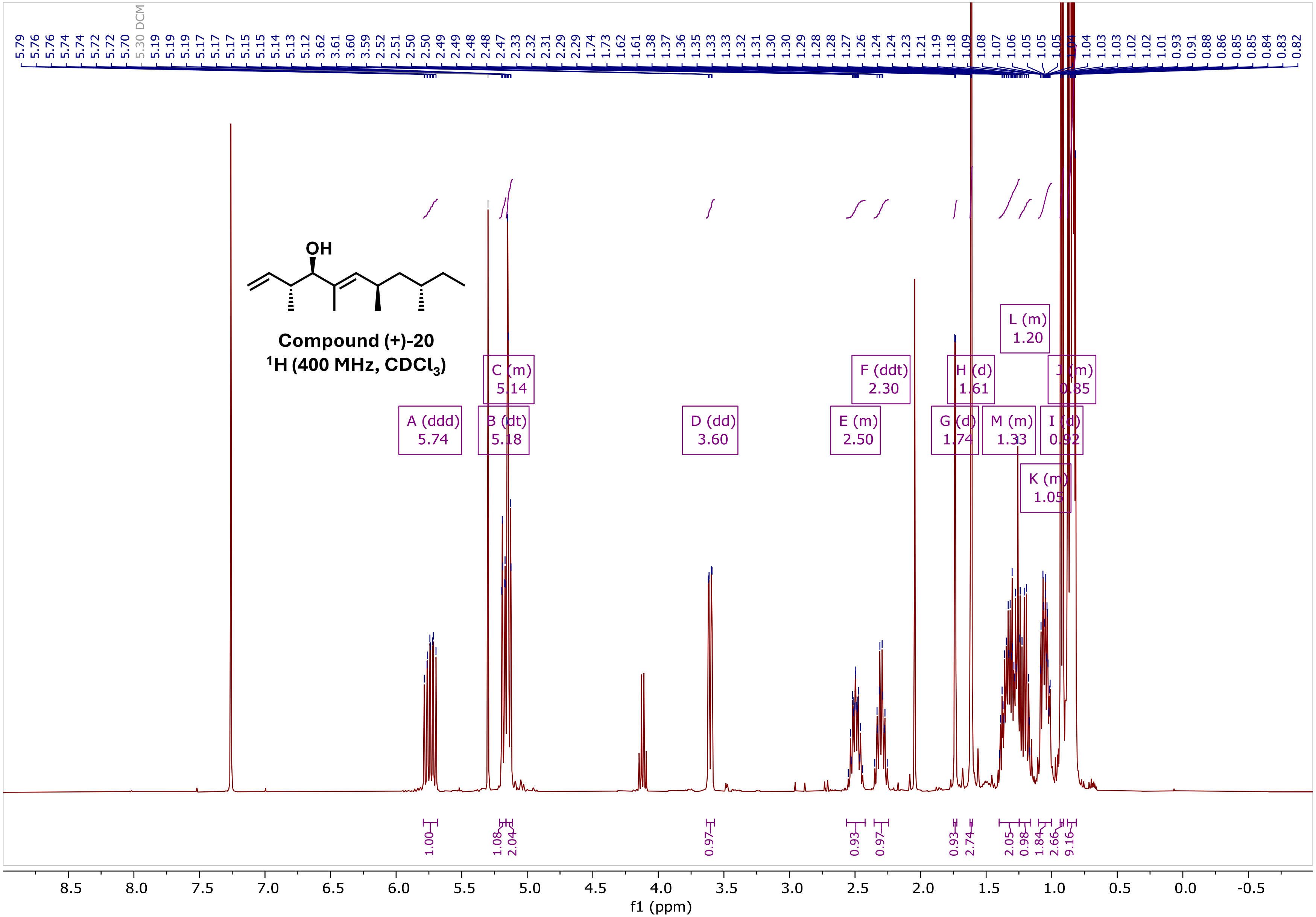
Compound (+)-20
^{1}H (400 MHz, CDCl$_3$)
OH
A (ddd) 5.74
B (dt) 5.18
C (m) 5.14
D (dd) 3.60
E (m) 2.50
F (ddt) 2.30
G (d) 1.74
H (d) 1.61
I (d) 0.92
J (m) 0.85
K (m) 1.05
L (m) 1.20
M (m) 1.33
5.79 5.76 5.76 5.74 5.74 5.72 5.72 5.70 5.30 DCM 5.19 5.19 5.19 5.17 5.17 5.17 5.15 5.15 5.14 5.13 5.12 3.62 3.61 3.60 3.59 2.52 2.51 2.50 2.49 2.49 2.48 2.48 2.47 2.33 2.32 2.31 2.29 2.29 1.74 1.73 1.62 1.61 1.38 1.37 1.36 1.35 1.33 1.33 1.32 1.31 1.30 1.30 1.29 1.28 1.28 1.27 1.26 1.24 1.24 1.23 1.21 1.19 1.18 1.09 1.08 1.07 1.06 1.05 1.05 1.05 1.04 1.04 1.03 1.03 1.02 1.02 1.01 0.93 0.91 0.88 0.86 0.85 0.85 0.84 0.83 0.82
1.00 1.08 2.04 0.97 0.93 0.97 0.93 2.74 2.05 0.98 1.84 2.66 9.16
8.5 8.0 7.5 7.0 6.5 6.0 5.5 5.0 4.5 4.0 3.5 3.0 2.5 2.0 1.5 1.0 0.5 0.0 -0.5
f1 (ppm)

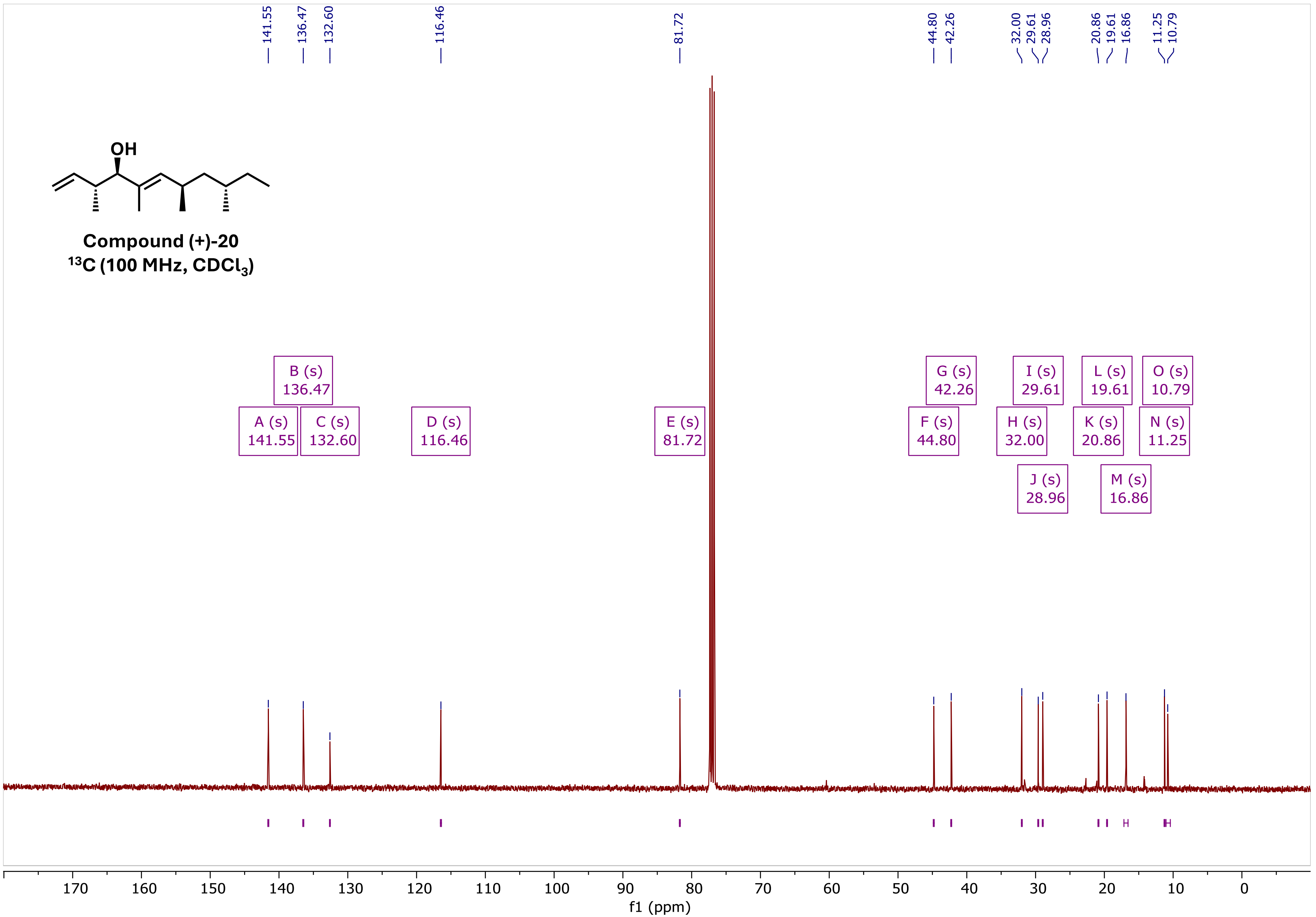
OH
Compound (+)-20
13C (100 MHz, CDCl3)
141.55
136.47
132.60
116.46
81.72
44.80
42.26
32.00
29.61
28.96
20.86
19.61
16.86
11.25
10.79
A (s) 141.55
B (s) 136.47
C (s) 132.60
D (s) 116.46
E (s) 81.72
F (s) 44.80
G (s) 42.26
H (s) 32.00
I (s) 29.61
J (s) 28.96
K (s) 20.86
L (s) 19.61
M (s) 16.86
N (s) 11.25
O (s) 10.79
170 160 150 140 130 120 110 100 90 80 70 60 50 40 30 20 10 0
f1 (ppm)

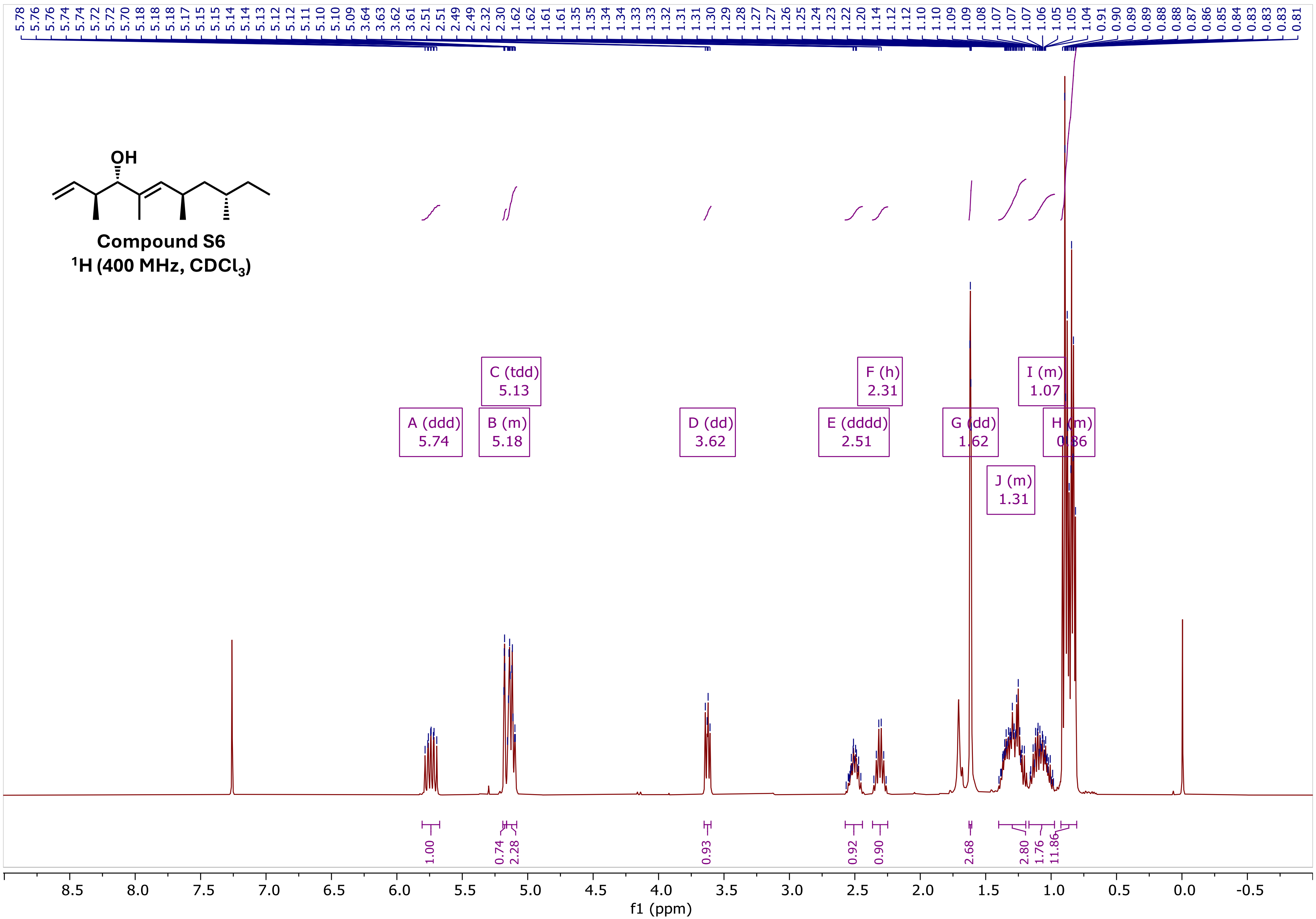
5.78 5.76 5.76 5.74 5.74 5.72 5.72 5.70 5.18 5.18 5.18 5.17 5.15 5.15 5.14 5.14 5.13 5.12 5.12 5.11 5.10 5.10 5.09 3.64 3.63 3.62 3.61 2.51 2.51 2.49 2.49 2.32 2.30 1.62 1.62 1.61 1.61 1.35 1.35 1.34 1.34 1.33 1.33 1.32 1.31 1.31 1.30 1.29 1.28 1.27 1.27 1.26 1.25 1.24 1.23 1.22 1.20 1.14 1.12 1.12 1.10 1.10 1.09 1.09 1.08 1.07 1.07 1.07 1.06 1.05 1.05 1.04 0.91 0.90 0.89 0.89 0.88 0.88 0.87 0.86 0.85 0.84 0.83 0.83 0.83 0.81
OH
Compound S6
¹H (400 MHz, CDCl₃)
A (ddd) 5.74
B (m) 5.18
C (tdd) 5.13
D (dd) 3.62
E (dddd) 2.51
F (h) 2.31
G (dd) 1.62
J (m) 1.31
I (m) 1.07
H (m) 0.86
1.00
0.74
2.28
0.93
0.92
0.90
2.68
2.80
1.76
11.86
8.5 8.0 7.5 7.0 6.5 6.0 5.5 5.0 4.5 4.0 3.5 3.0 2.5 2.0 1.5 1.0 0.5 0.0 -0.5
f1 (ppm)

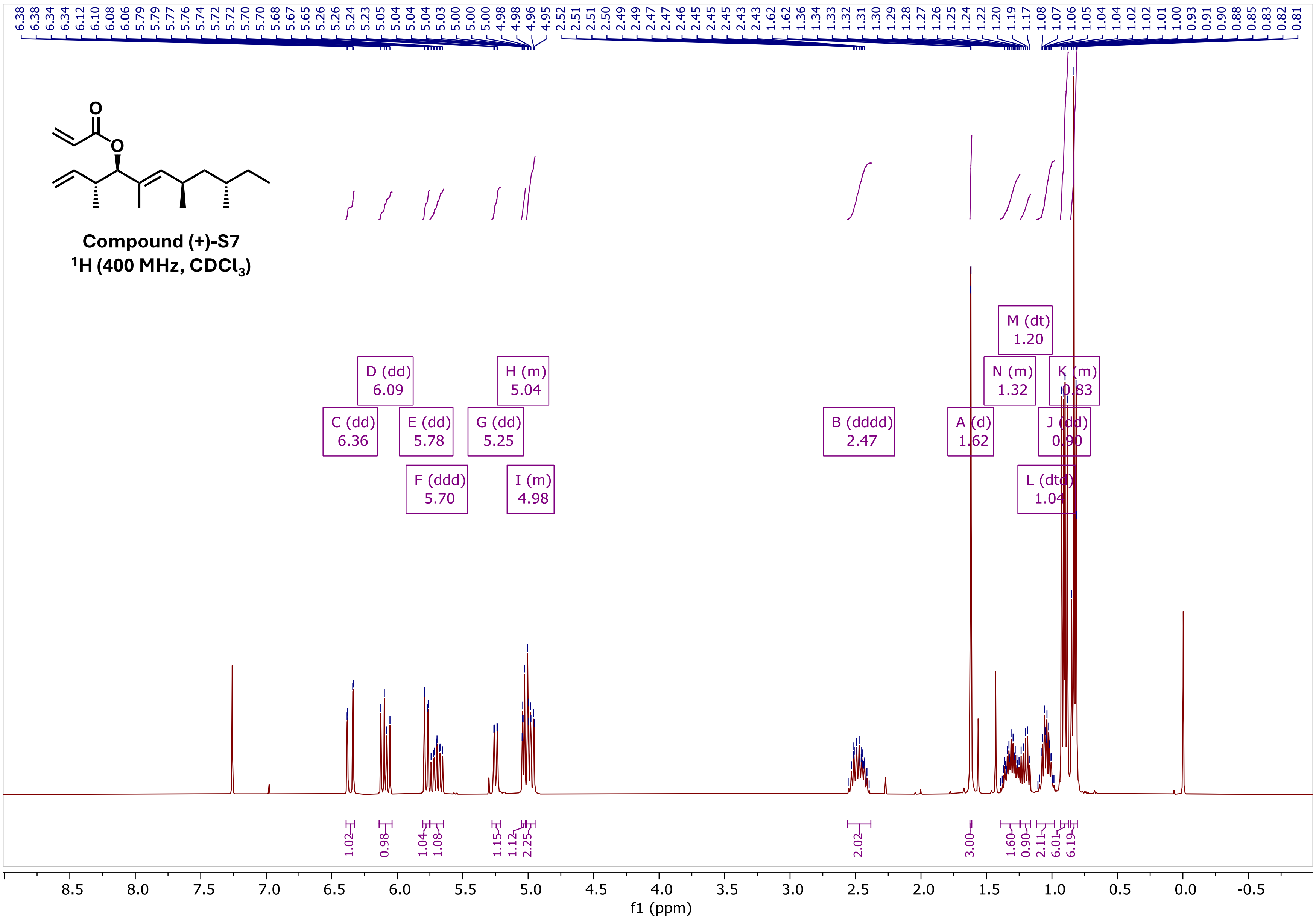
Compound (+)-S7
^{1}H (400 MHz, $CDCl_3$)
C (dd) 6.36
D (dd) 6.09
E (dd) 5.78
F (ddd) 5.70
G (dd) 5.25
H (m) 5.04
I (m) 4.98
B (dddd) 2.47
A (d) 1.62
N (m) 1.32
M (dt) 1.20
L (dtd) 1.04
J (dd) 0.90
K (m) 0.83
1.02
0.98
1.04
1.08
1.15
1.12
2.25
2.02
3.00
1.60
0.90
2.11
6.01
6.19
f1 (ppm)

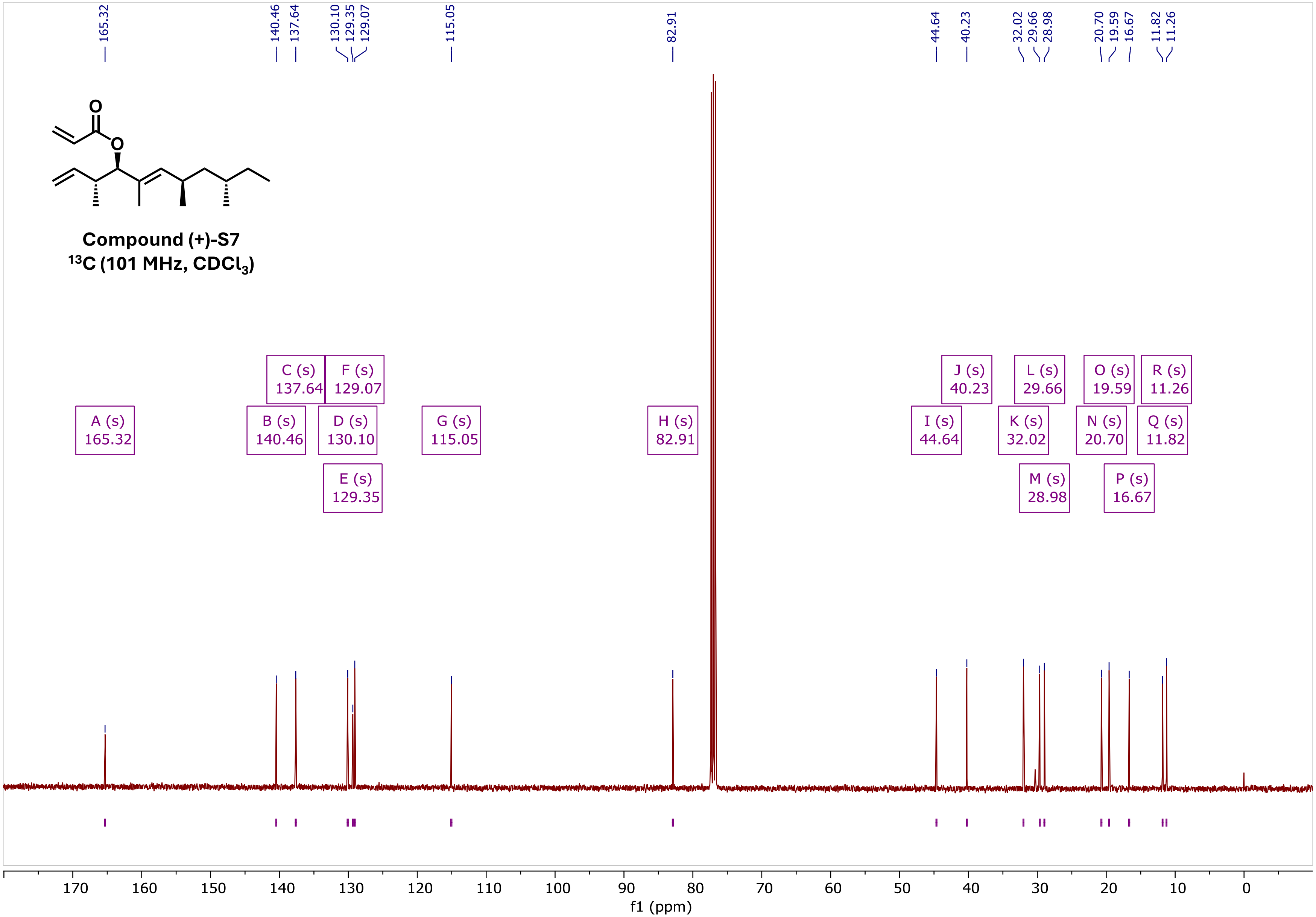
165.32
140.46
137.64
130.10
129.35
129.07
115.05
82.91
44.64
40.23
32.02
29.66
28.98
20.70
19.59
16.67
11.82
11.26
Compound (+)-S7
13C (101 MHz, CDCl3)
A (s) 165.32
B (s) 140.46
C (s) 137.64
D (s) 130.10
E (s) 129.35
F (s) 129.07
G (s) 115.05
H (s) 82.91
I (s) 44.64
J (s) 40.23
K (s) 32.02
L (s) 29.66
M (s) 28.98
N (s) 20.70
O (s) 19.59
P (s) 16.67
Q (s) 11.82
R (s) 11.26
f1 (ppm)

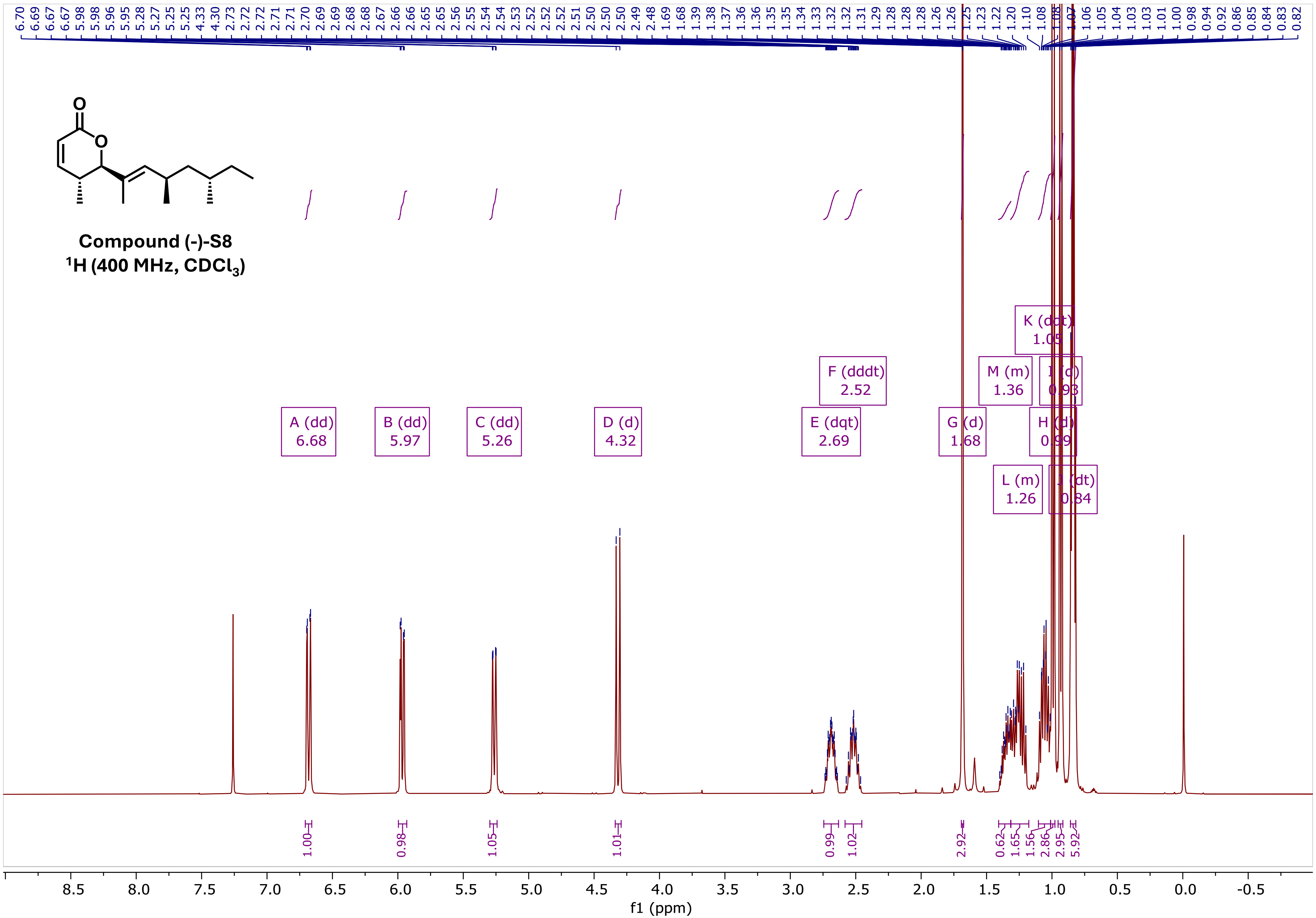

Compound (-)-S8
^{1}H (400 MHz, $CDCl_3$)
6.70 6.69 6.67 6.67 5.98 5.98 5.96 5.95 5.28 5.27 5.25 5.25 4.33 4.30 2.73 2.72 2.72 2.71 2.71 2.70 2.69 2.69 2.68 2.68 2.67 2.66 2.66 2.65 2.65 2.56 2.55 2.54 2.54 2.53 2.52 2.52 2.52 2.51 2.50 2.50 2.50 2.49 2.48 1.69 1.68 1.39 1.38 1.37 1.36 1.36 1.35 1.35 1.34 1.33 1.32 1.32 1.31 1.29 1.28 1.28 1.28 1.26 1.26 1.25 1.23 1.22 1.20 1.10 1.08 1.06 1.05 1.04 1.03 1.03 1.01 1.00 0.98 0.94 0.92 0.86 0.85 0.84 0.83 0.82
A (dd) 6.68
B (dd) 5.97
C (dd) 5.26
D (d) 4.32
E (dqt) 2.69
F (dddt) 2.52
G (d) 1.68
M (m) 1.36
L (m) 1.26
1.00 0.98 1.05 1.01 0.99 1.02 2.92 0.62 1.65 1.56 2.86 2.95 5.92
8.5 8.0 7.5 7.0 6.5 6.0 5.5 5.0 4.5 4.0 3.5 3.0 2.5 2.0 1.5 1.0 0.5 0.0 -0.5
f1 (ppm)

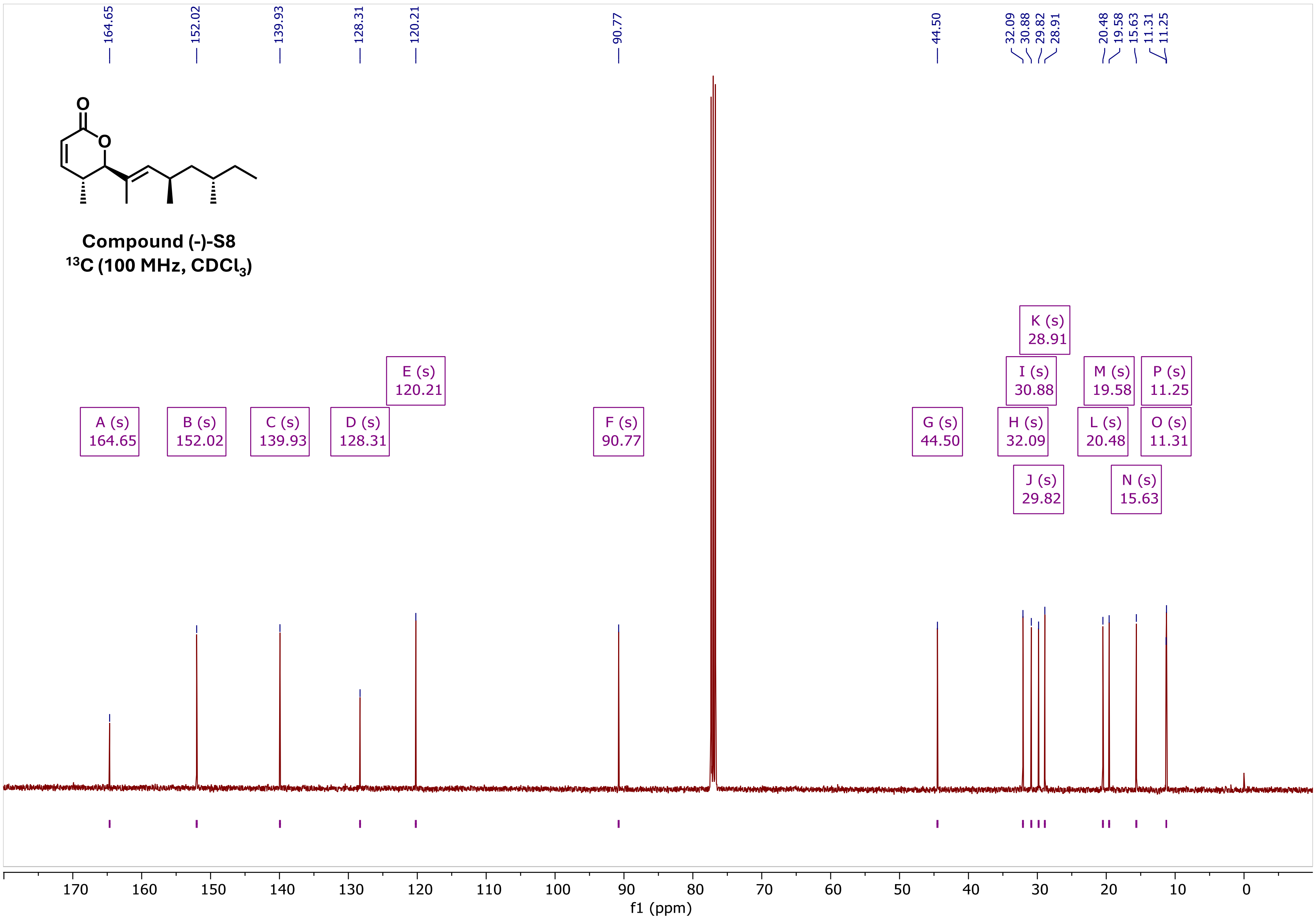
164.65
152.02
139.93
128.31
120.21
90.77
44.50
32.09
30.88
29.82
28.91
20.48
19.58
15.63
11.31
11.25
Compound (-)-S8
13C (100 MHz, CDCl3)
A (s) 164.65
B (s) 152.02
C (s) 139.93
D (s) 128.31
E (s) 120.21
F (s) 90.77
G (s) 44.50
H (s) 32.09
I (s) 30.88
J (s) 29.82
K (s) 28.91
L (s) 20.48
M (s) 19.58
N (s) 15.63
O (s) 11.31
P (s) 11.25
170
160
150
140
130
120
110
100
90
80
70
60
50
40
30
20
10
0
f1 (ppm)

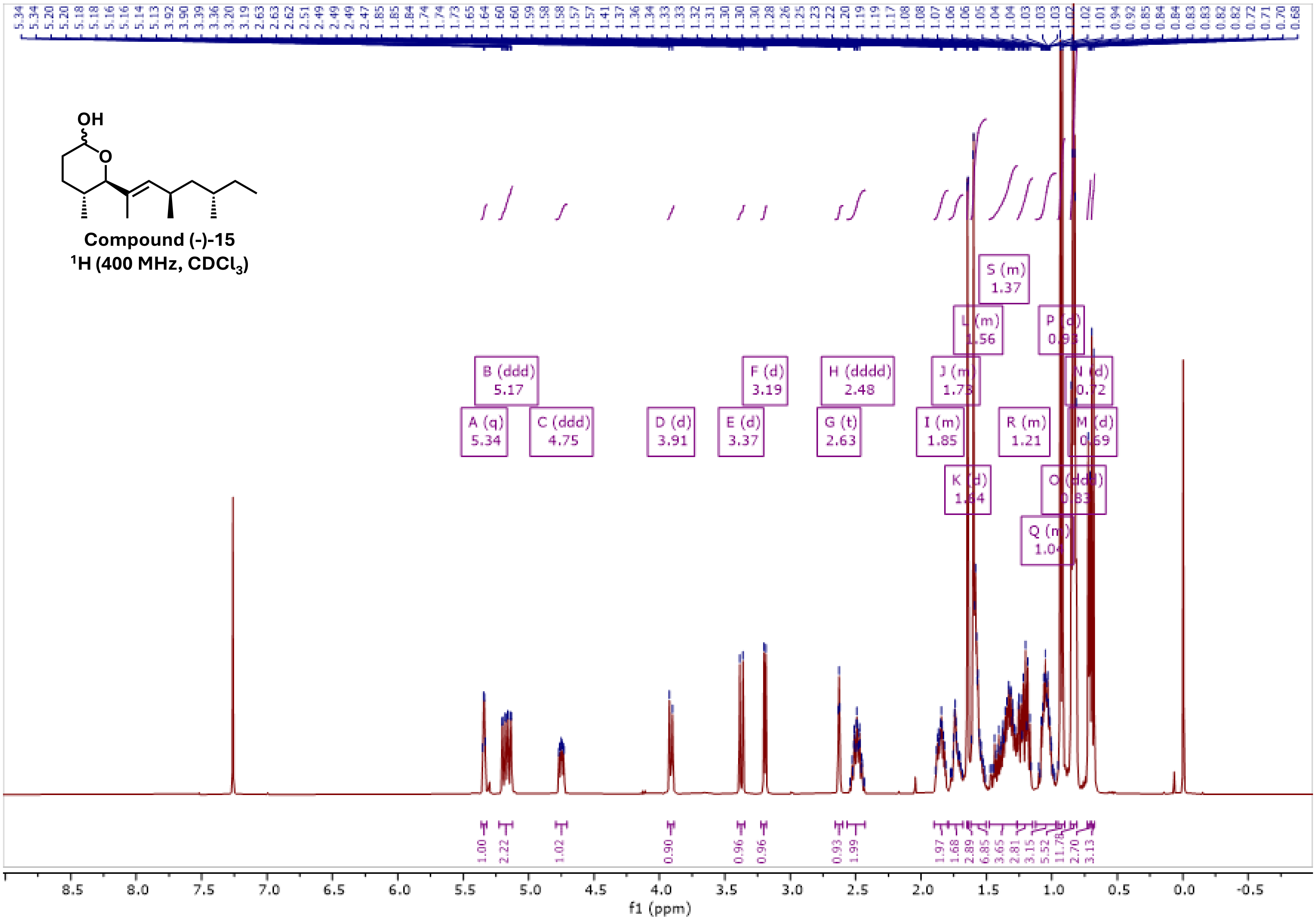
OH
O
Compound (-)-15
1H (400 MHz, CDCl3)
f1 (ppm)

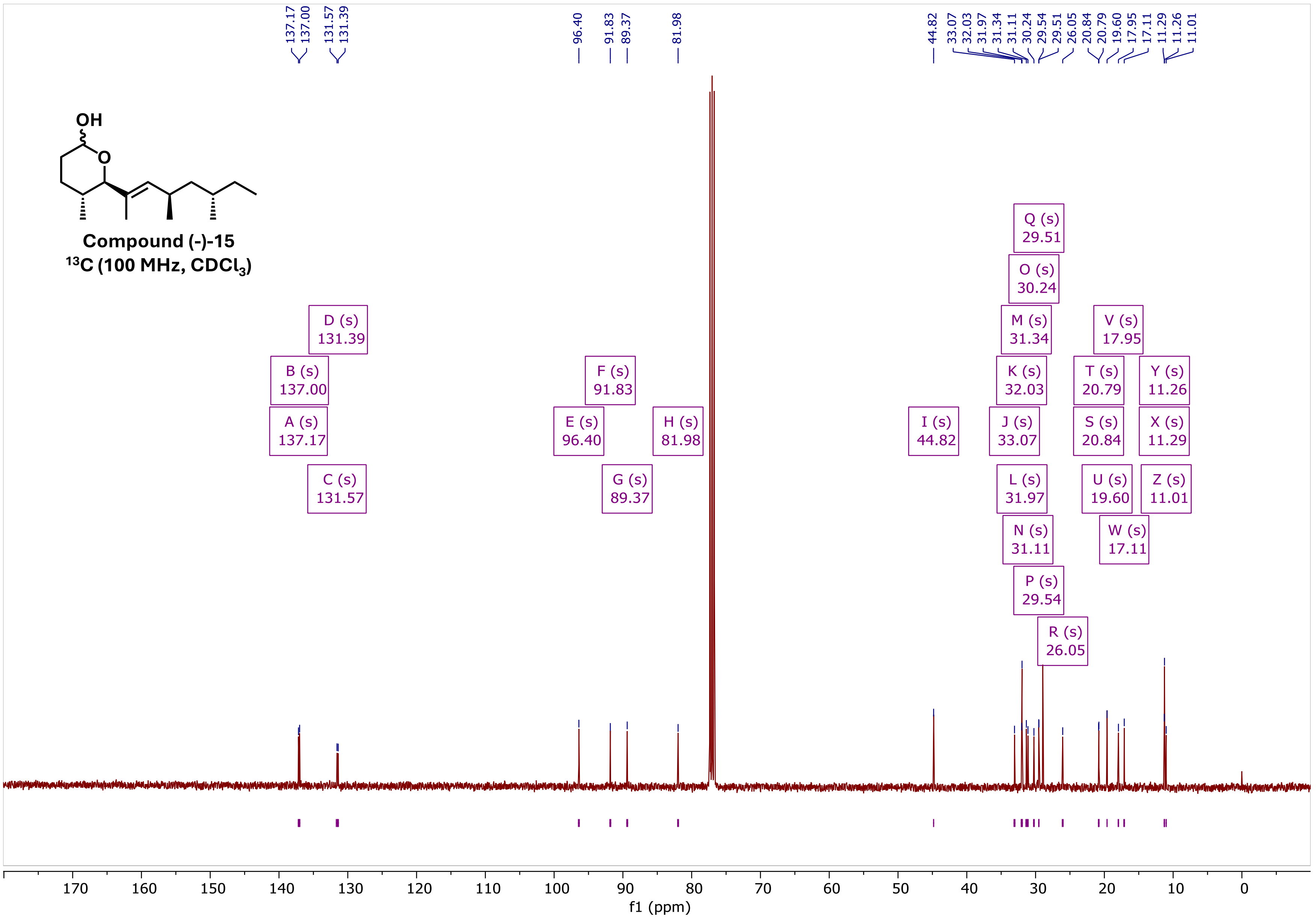

Compound (-)-15

^{13}C (100 MHz, $CDCl_3$)

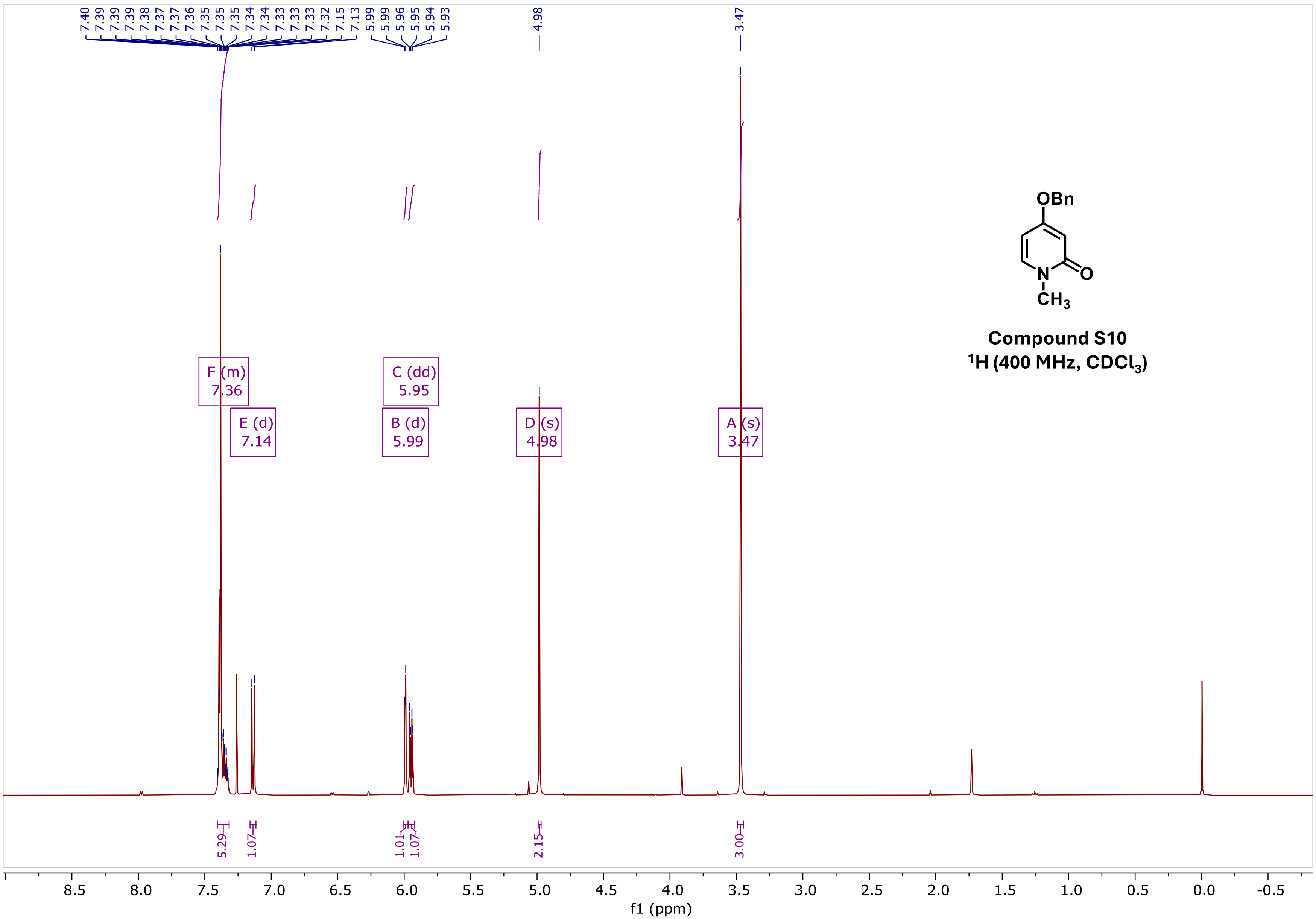

7.40 7.39 7.39 7.39 7.38 7.37 7.37 7.36 7.35 7.35 7.35 7.34 7.34 7.33 7.33 7.33 7.32 7.15 7.13 5.99 5.99 5.96 5.95 5.94 5.93
4.98
3.47
OBn
N
O
CH3
Compound S10
1H (400 MHz, CDCl3)
F (m) 7.36
E (d) 7.14
C (dd) 5.95
B (d) 5.99
D (s) 4.98
A (s) 3.47
5.29
1.07
1.01
1.07
2.15
3.00
8.5 8.0 7.5 7.0 6.5 6.0 5.5 5.0 4.5 4.0 3.5 3.0 2.5 2.0 1.5 1.0 0.5 0.0 -0.5
f1 (ppm)

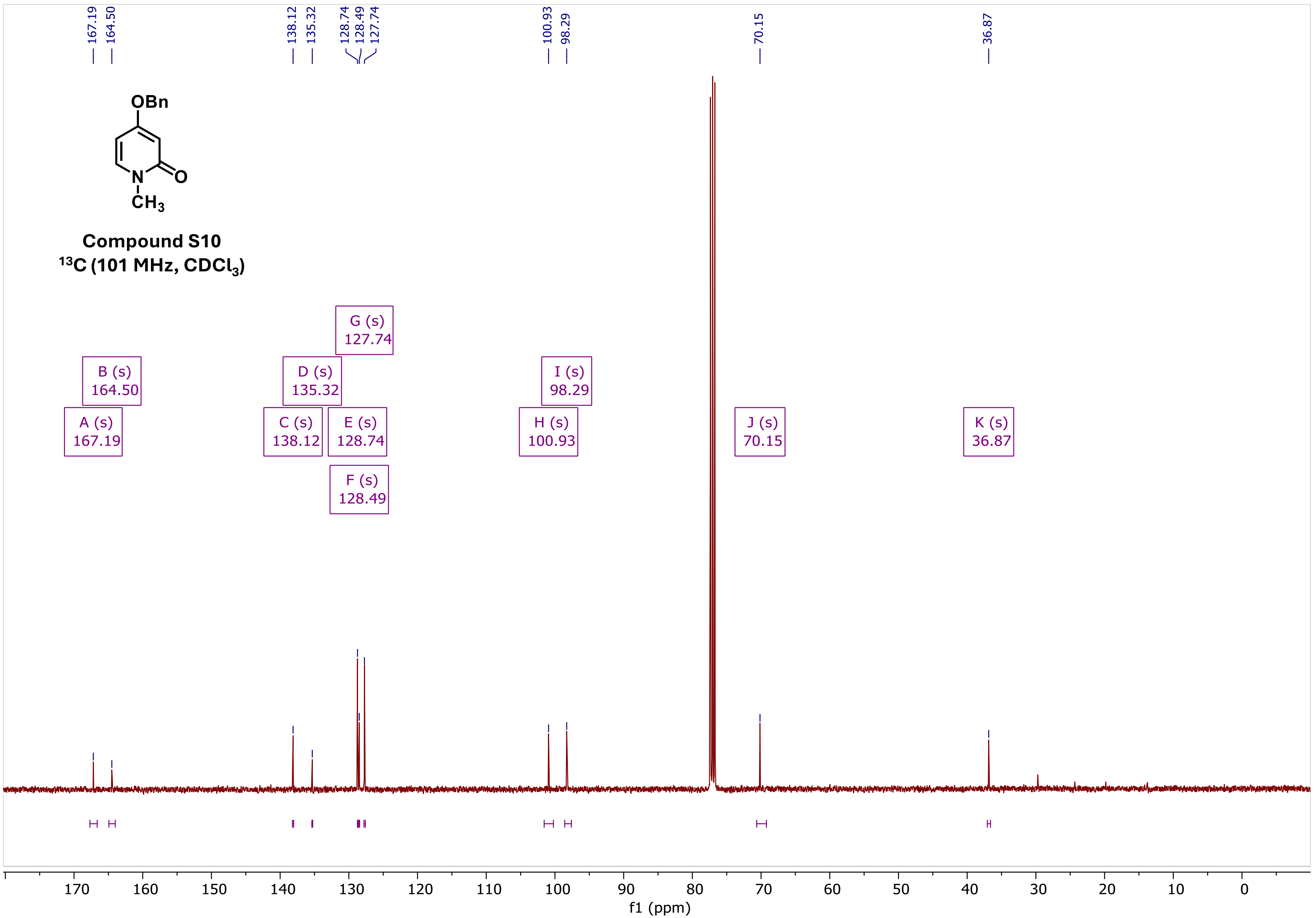
167.19
164.50
138.12
135.32
128.74
128.49
127.74
100.93
98.29
70.15
36.87
OBn
N
O
CH3
Compound S10
13C (101 MHz, CDCl3)
A (s) 167.19
B (s) 164.50
C (s) 138.12
D (s) 135.32
E (s) 128.74
F (s) 128.49
G (s) 127.74
H (s) 100.93
I (s) 98.29
J (s) 70.15
K (s) 36.87
170 160 150 140 130 120 110 100 90 80 70 60 50 40 30 20 10 0
f1 (ppm)

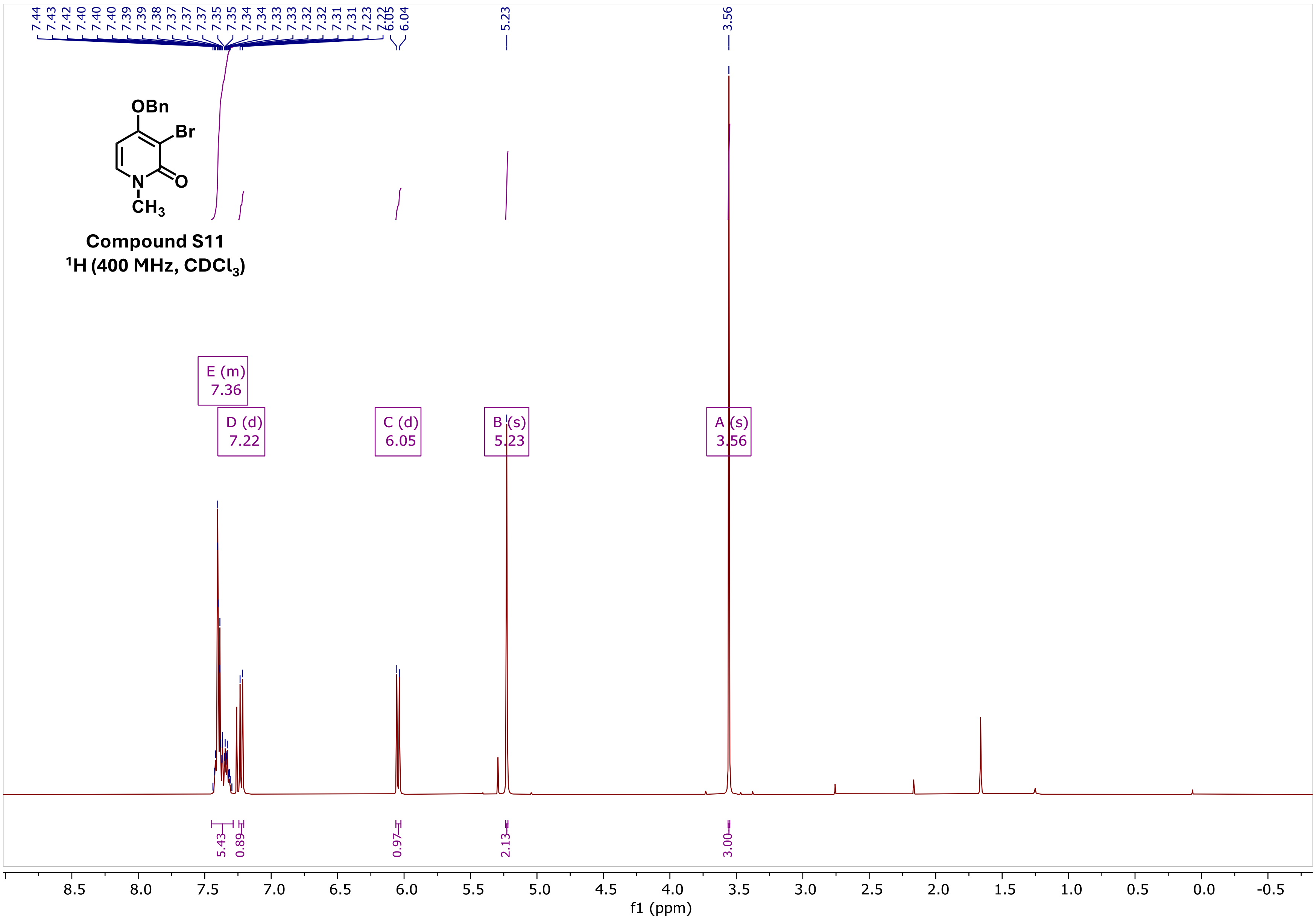
7.44 7.43 7.42 7.40 7.40 7.40 7.39 7.39 7.38 7.37 7.37 7.37 7.35 7.35 7.34 7.34 7.33 7.33 7.32 7.32 7.31 7.31 7.23 7.22 6.05 6.04
5.23
3.56
OBn
Br
N
O
CH3
Compound S11
1H (400 MHz, CDCl3)
E (m) 7.36
D (d) 7.22
C (d) 6.05
B (s) 5.23
A (s) 3.56
5.43
0.89
0.97
2.13
3.00
8.5 8.0 7.5 7.0 6.5 6.0 5.5 5.0 4.5 4.0 3.5 3.0 2.5 2.0 1.5 1.0 0.5 0.0 -0.5
f1 (ppm)

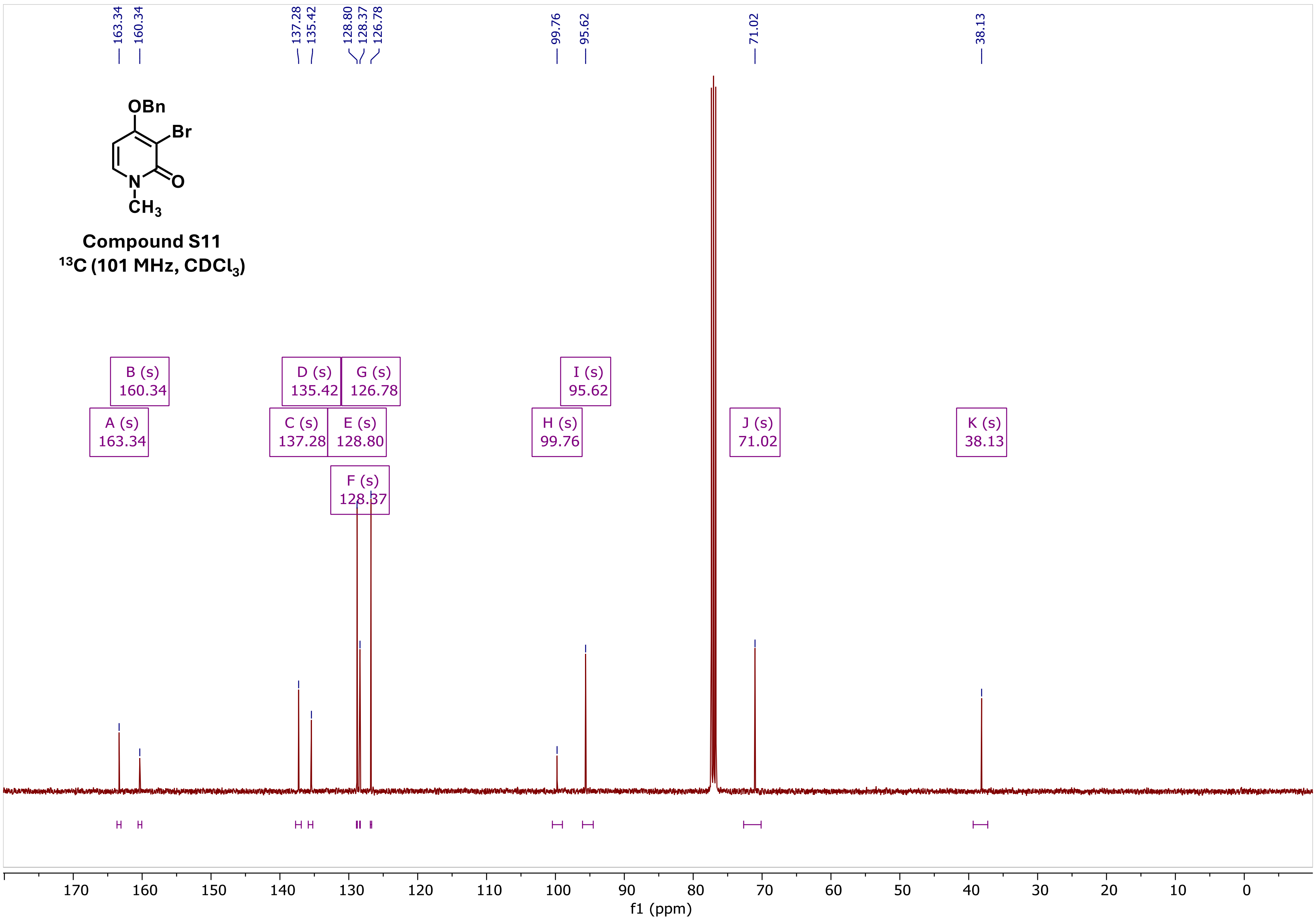

163.34
160.34
137.28
135.42
128.80
128.37
126.78
99.76
95.62
71.02
38.13
OBn
Br
N
O
CH3
Compound S11
13C (101 MHz, CDCl3)
A (s) 163.34
B (s) 160.34
C (s) 137.28
D (s) 135.42
E (s) 128.80
F (s) 128.37
G (s) 126.78
H (s) 99.76
I (s) 95.62
J (s) 71.02
K (s) 38.13
170 160 150 140 130 120 110 100 90 80 70 60 50 40 30 20 10 0
f1 (ppm)

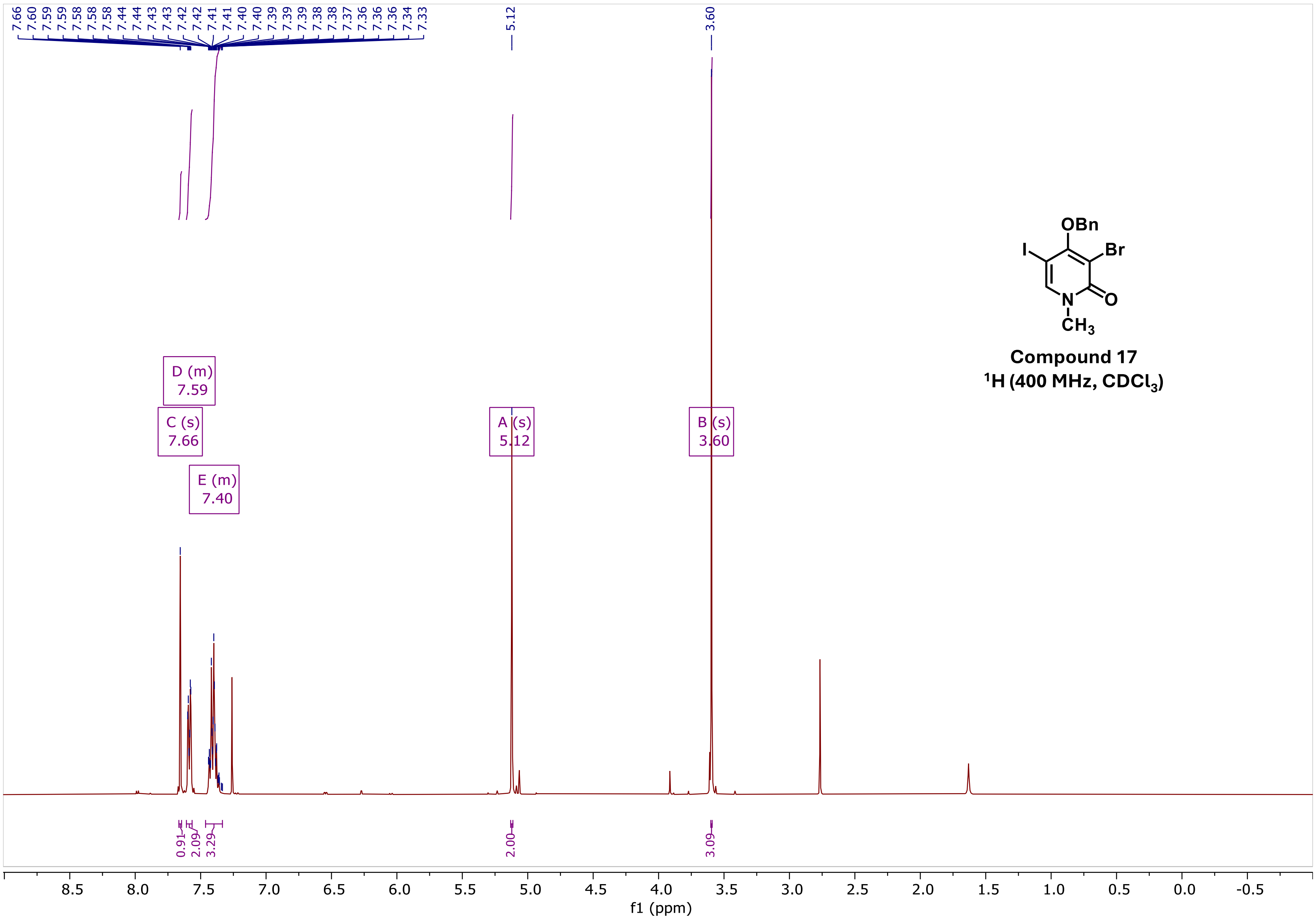

Compound 17

^{1}H (400 MHz, $CDCl_3$)

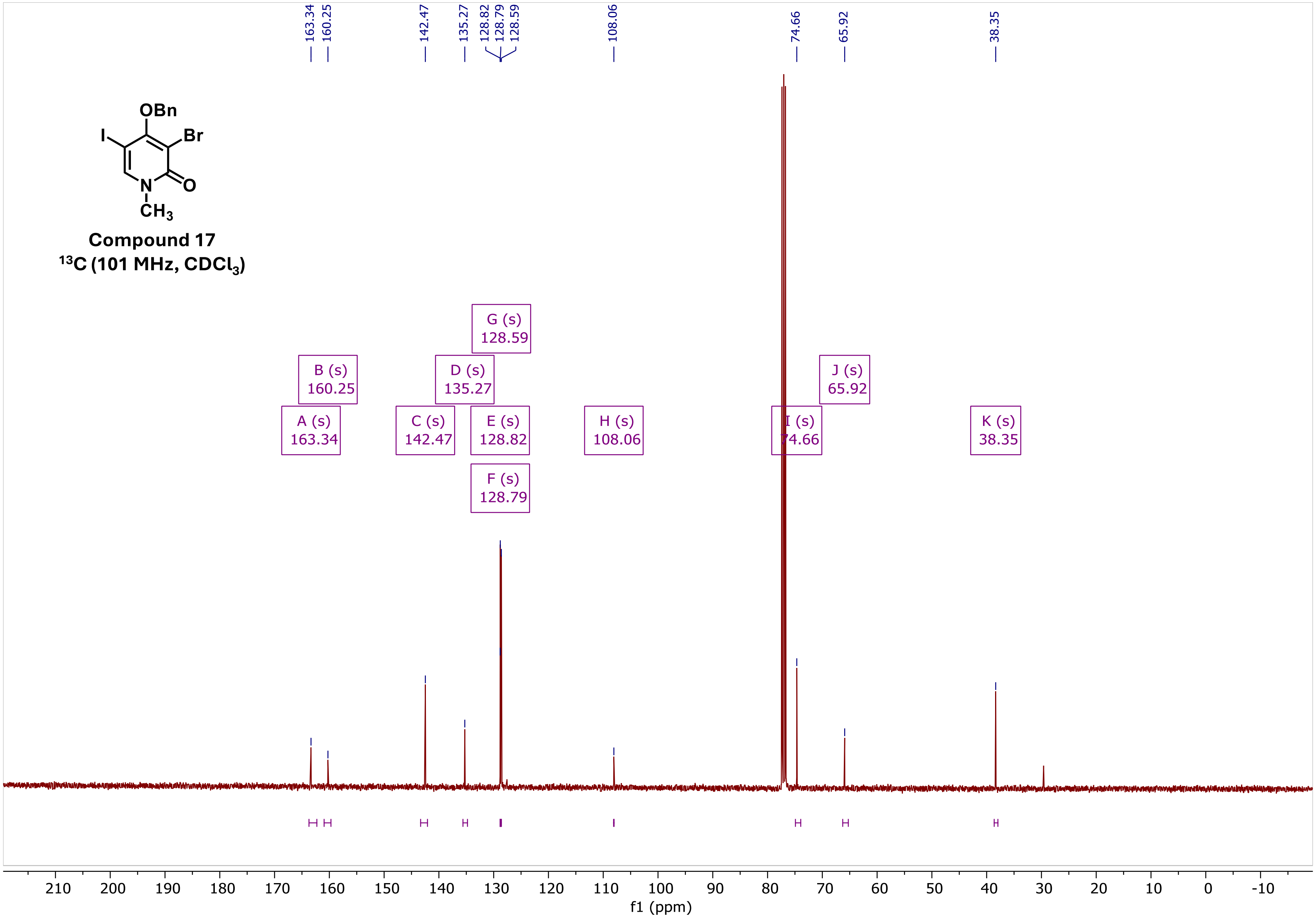
OBn
I
Br
N
O
CH3
Compound 17
13C (101 MHz, CDCl3)
163.34
160.25
142.47
135.27
128.82
128.79
128.59
108.06
74.66
65.92
38.35
A (s) 163.34
B (s) 160.25
C (s) 142.47
D (s) 135.27
E (s) 128.82
F (s) 128.79
G (s) 128.59
H (s) 108.06
I (s) 74.66
J (s) 65.92
K (s) 38.35
210 200 190 180 170 160 150 140 130 120 110 100 90 80 70 60 50 40 30 20 10 0 -10
f1 (ppm)

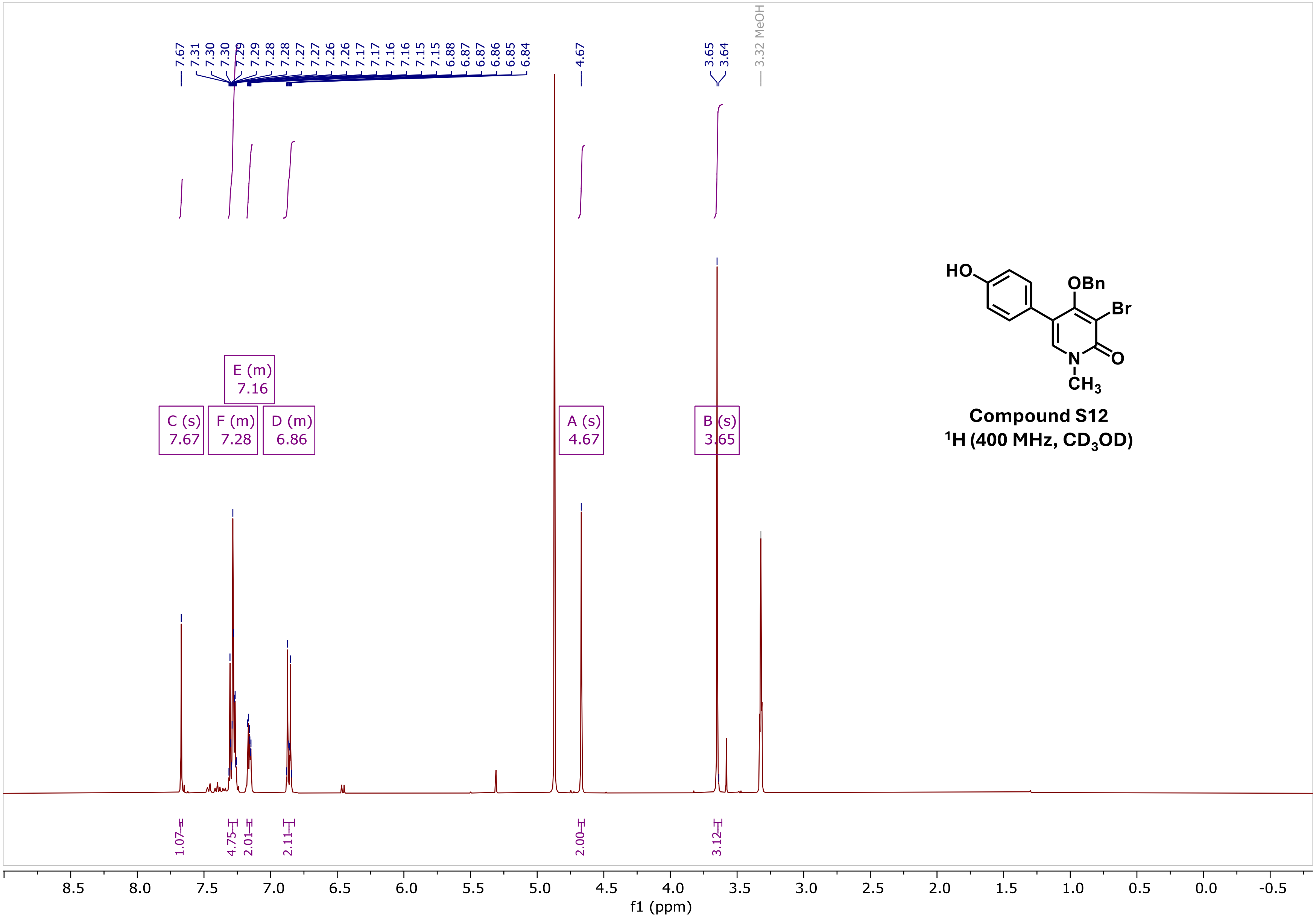
7.67
7.31
7.30
7.30
7.29
7.29
7.28
7.28
7.27
7.27
7.26
7.26
7.17
7.17
7.16
7.16
7.15
7.15
6.88
6.87
6.87
6.86
6.85
6.84
4.67
3.65
3.64
3.32 MeOH
E (m)
7.16
C (s)
7.67
F (m)
7.28
D (m)
6.86
A (s)
4.67
B (s)
3.65
HO
OBn
Br
N
O
CH3
Compound S12
1H (400 MHz, CD3OD)
1.07
4.75
2.01
2.11
2.00
3.12
8.5
8.0
7.5
7.0
6.5
6.0
5.5
5.0
4.5
4.0
3.5
3.0
2.5
2.0
1.5
1.0
0.5
0.0
-0.5
f1 (ppm)

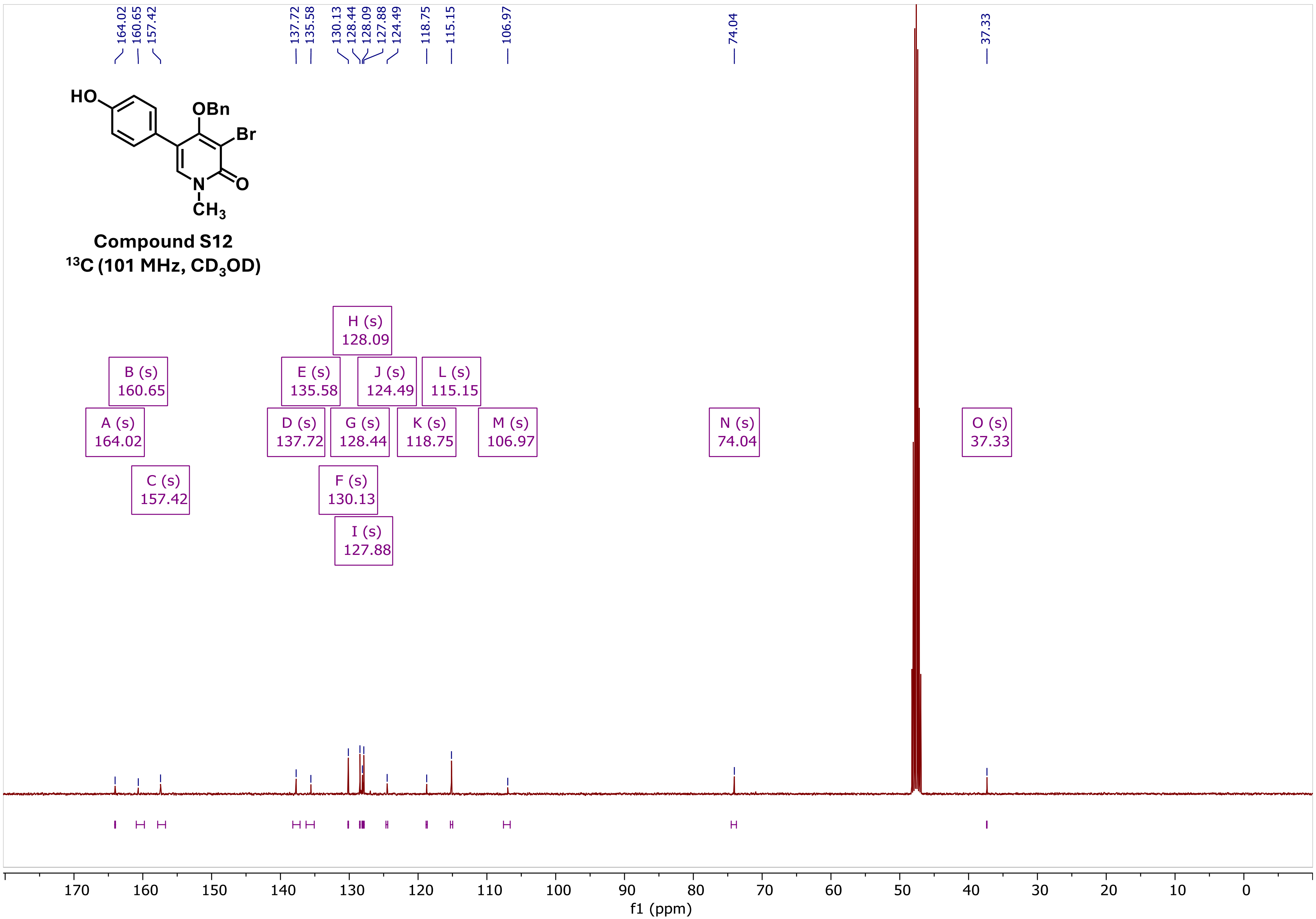
HO
OBn
Br
N
O
CH3
Compound S12
13C (101 MHz, CD3OD)
164.02
160.65
157.42
137.72
135.58
130.13
128.44
128.09
127.88
124.49
118.75
115.15
106.97
74.04
37.33
A (s) 164.02
B (s) 160.65
C (s) 157.42
D (s) 137.72
E (s) 135.58
F (s) 130.13
G (s) 128.44
H (s) 128.09
I (s) 127.88
J (s) 124.49
K (s) 118.75
L (s) 115.15
M (s) 106.97
N (s) 74.04
O (s) 37.33
170
160
150
140
130
120
110
100
90
80
70
60
50
40
30
20
10
0
f1 (ppm)

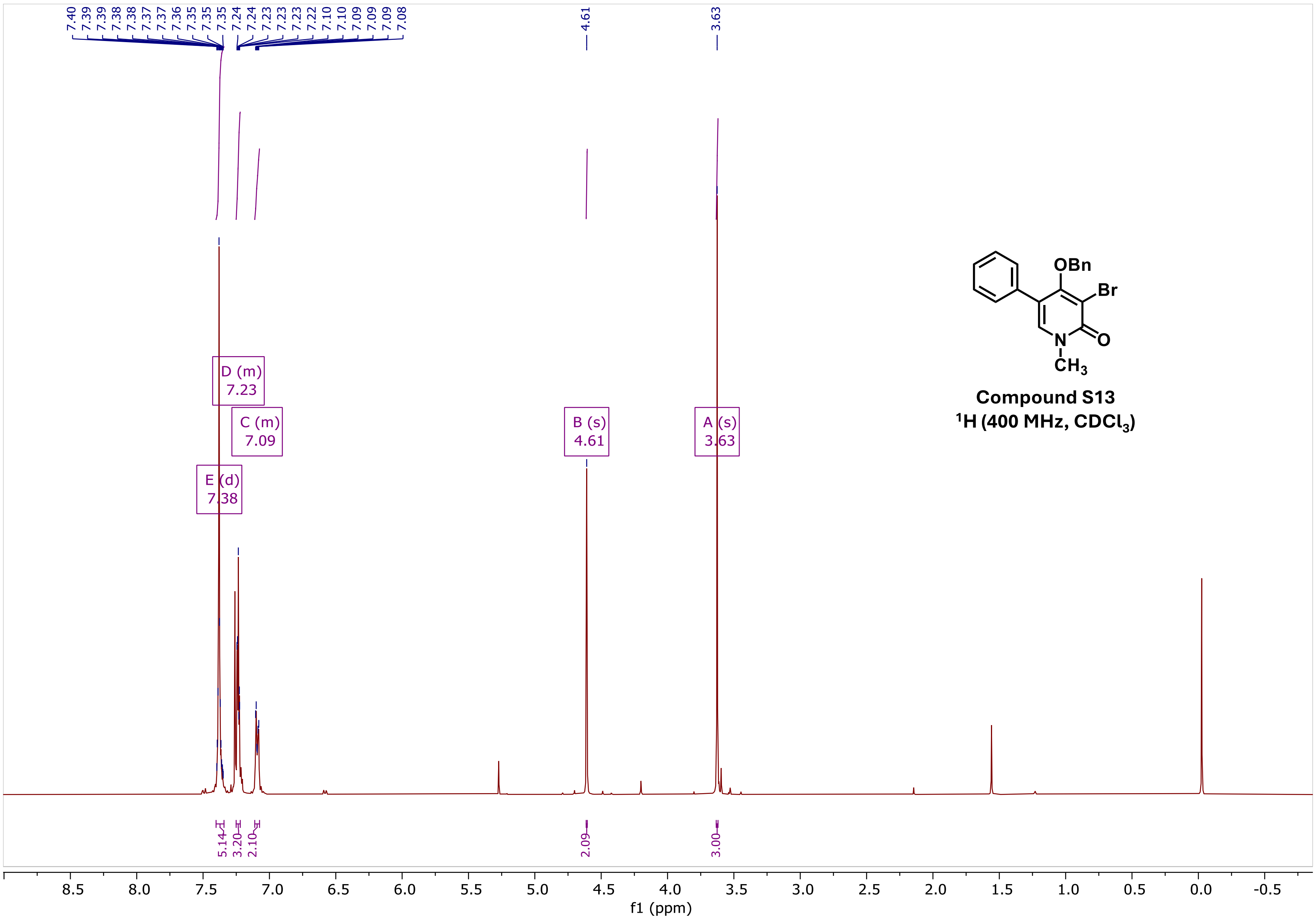

7.40
7.39
7.39
7.38
7.38
7.37
7.37
7.36
7.35
7.35
7.35
7.24
7.24
7.23
7.23
7.23
7.22
7.10
7.10
7.09
7.09
7.09
7.08
4.61
3.63
D (m)
7.23
C (m)
7.09
E (d)
7.38
B (s)
4.61
A (s)
3.63
OBn
Br
N
O
CH3
Compound S13
1H (400 MHz, CDCl3)
5.14
3.20
2.10
2.09
3.00
8.5
8.0
7.5
7.0
6.5
6.0
5.5
5.0
4.5
4.0
3.5
3.0
2.5
2.0
1.5
1.0
0.5
0.0
-0.5
f1 (ppm)

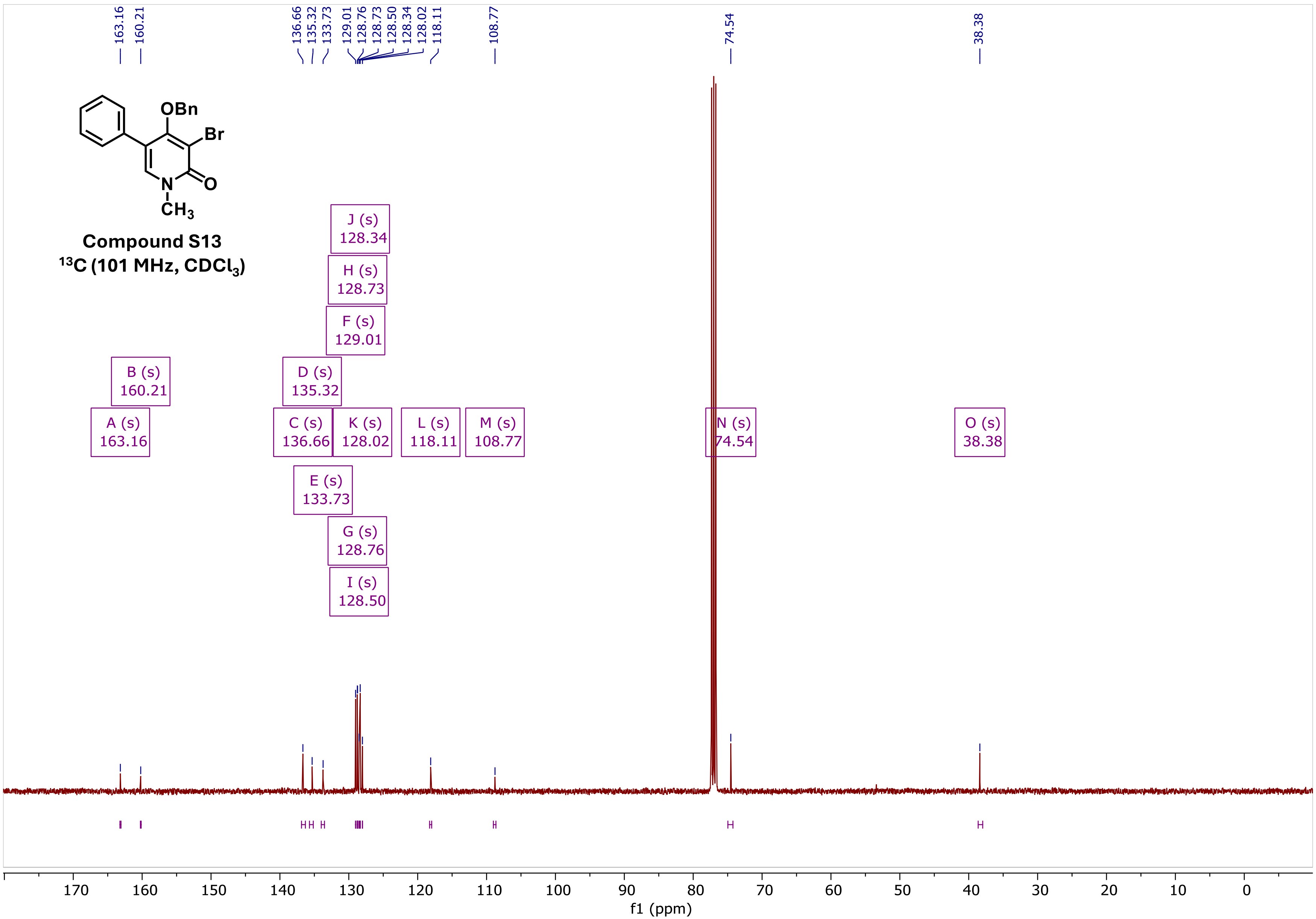
OBn
Br
N
O
CH3
Compound S13
13C (101 MHz, CDCl3)
163.16
160.21
136.66
135.32
133.73
129.01
128.76
128.73
128.50
128.34
128.02
118.11
108.77
74.54
38.38
A (s) 163.16
B (s) 160.21
C (s) 136.66
D (s) 135.32
E (s) 133.73
F (s) 129.01
G (s) 128.76
H (s) 128.73
I (s) 128.50
J (s) 128.34
K (s) 128.02
L (s) 118.11
M (s) 108.77
N (s) 74.54
O (s) 38.38
f1 (ppm)

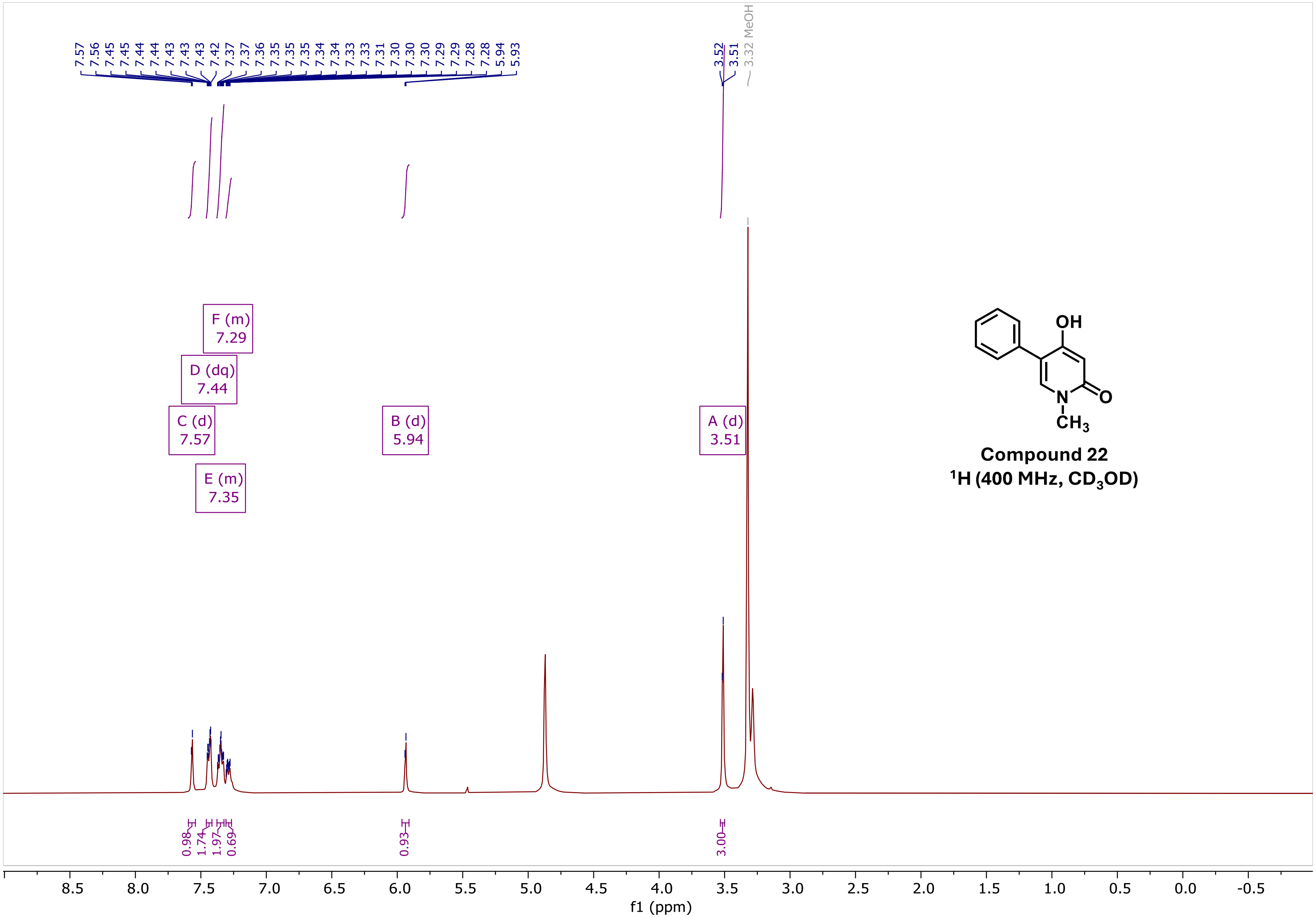
7.57
7.56
7.45
7.45
7.44
7.44
7.43
7.43
7.43
7.42
7.37
7.37
7.36
7.35
7.35
7.35
7.34
7.34
7.33
7.33
7.31
7.30
7.30
7.30
7.29
7.29
7.28
7.28
5.94
5.93
3.52
3.51
3.32 MeOH
F (m)
7.29
D (dq)
7.44
C (d)
7.57
B (d)
5.94
A (d)
3.51
E (m)
7.35
OH
N
O
CH3
Compound 22
1H (400 MHz, CD3OD)
0.98
1.74
1.97
0.69
0.93
3.00
8.5
8.0
7.5
7.0
6.5
6.0
5.5
5.0
4.5
4.0
3.5
3.0
2.5
2.0
1.5
1.0
0.5
0.0
-0.5
f1 (ppm)

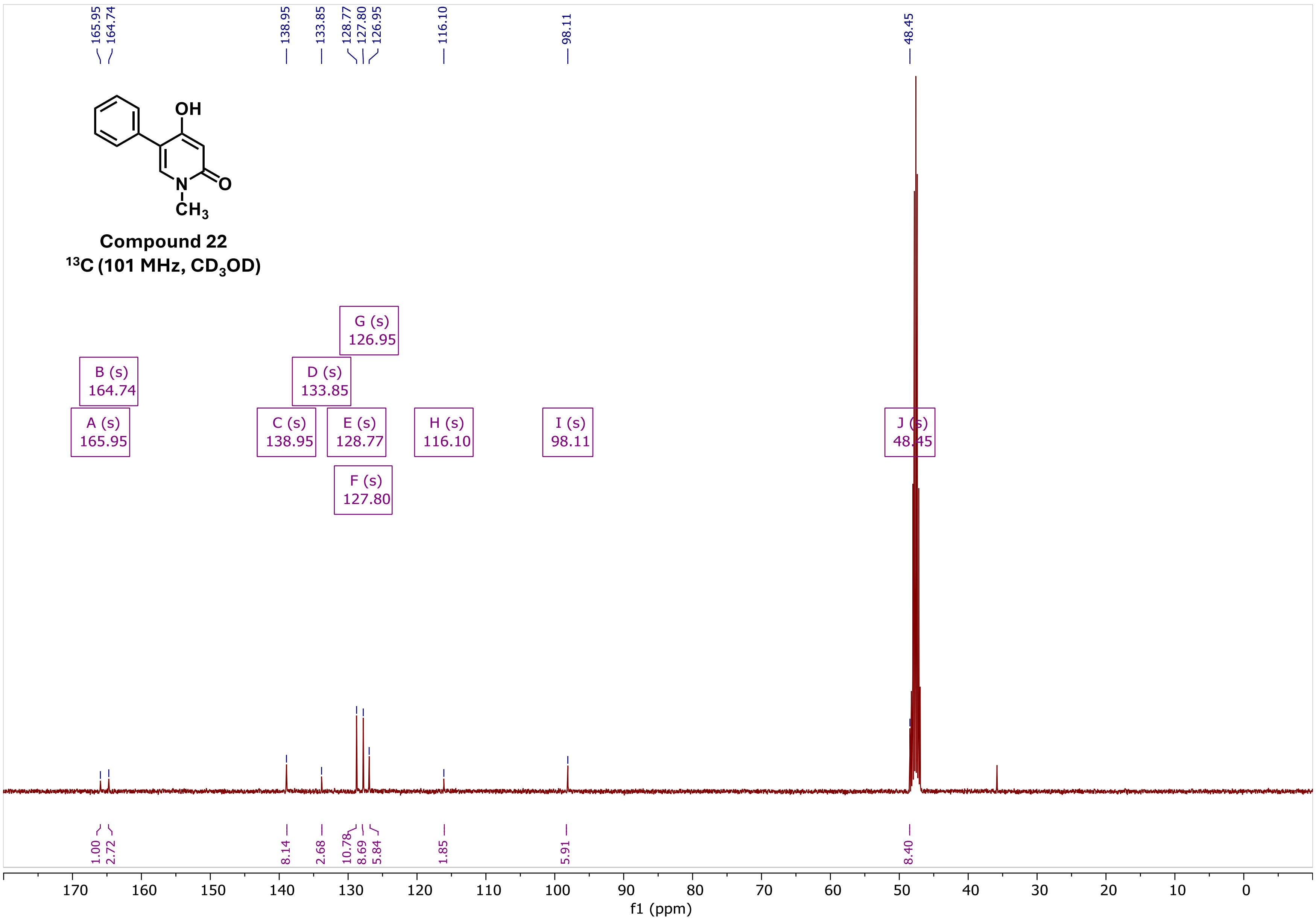

165.95
164.74
138.95
133.85
128.77
127.80
126.95
116.10
98.11
48.45
OH
N
O
CH3
Compound 22
13C (101 MHz, CD3OD)
G (s)
126.95
B (s)
164.74
D (s)
133.85
A (s)
165.95
C (s)
138.95
E (s)
128.77
H (s)
116.10
I (s)
98.11
J (s)
48.45
F (s)
127.80
1.00
2.72
8.14
2.68
10.78
8.69
5.84
1.85
5.91
8.40
170
160
150
140
130
120
110
100
90
80
70
60
50
40
30
20
10
0
f1 (ppm)

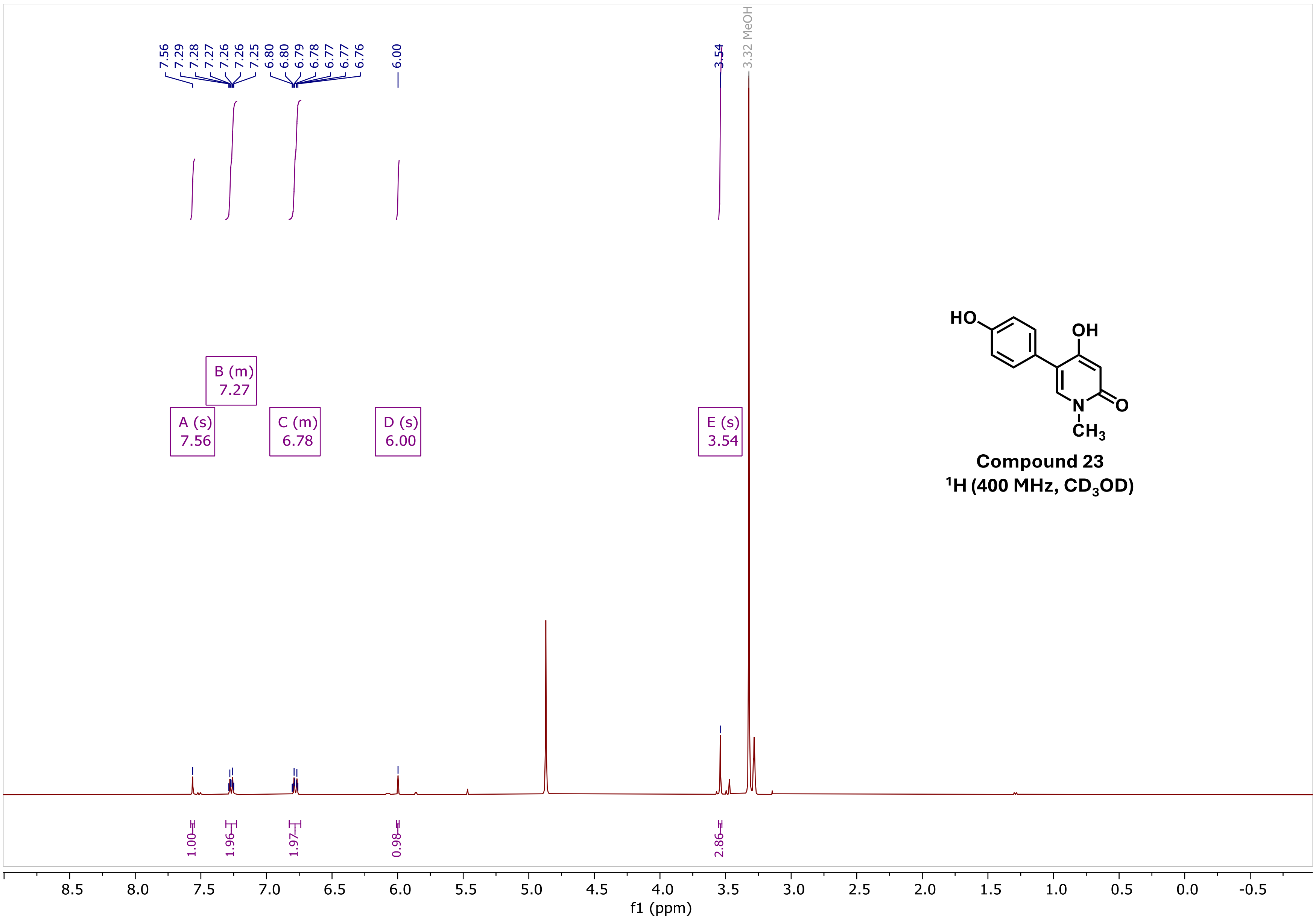
7.56
7.29
7.28
7.27
7.26
7.26
7.25
6.80
6.80
6.79
6.78
6.77
6.77
6.76
6.00
3.54
3.32 MeOH
A (s)
7.56
B (m)
7.27
C (m)
6.78
D (s)
6.00
E (s)
3.54
1.00
1.96
1.97
0.98
2.86
HO
OH
N
O
CH3
Compound 23
1H (400 MHz, CD3OD)
8.5
8.0
7.5
7.0
6.5
6.0
5.5
5.0
4.5
4.0
3.5
3.0
2.5
2.0
1.5
1.0
0.5
0.0
-0.5
f1 (ppm)

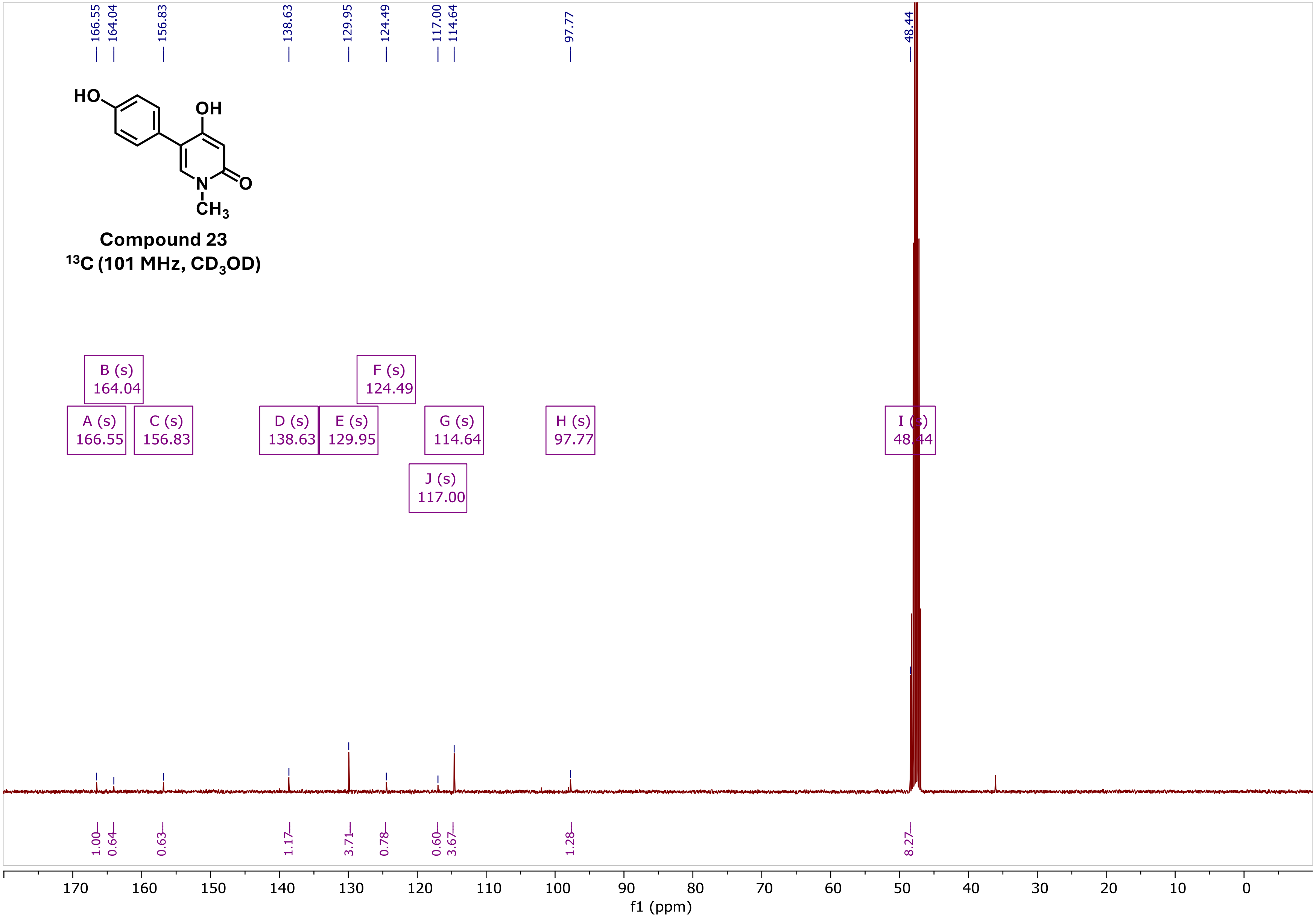

166.55
164.04
156.83
138.63
129.95
124.49
117.00
114.64
97.77
48.44
HO
OH
N
O
CH3
Compound 23
13C (101 MHz, CD3OD)
B (s)
164.04
F (s)
124.49
A (s)
166.55
C (s)
156.83
D (s)
138.63
E (s)
129.95
G (s)
114.64
H (s)
97.77
I (s)
48.44
J (s)
117.00
1.00
0.64
0.63
1.17
3.71
0.78
0.60
3.67
1.28
8.27
170
160
150
140
130
120
110
100
90
80
70
60
50
40
30
20
10
0
f1 (ppm)

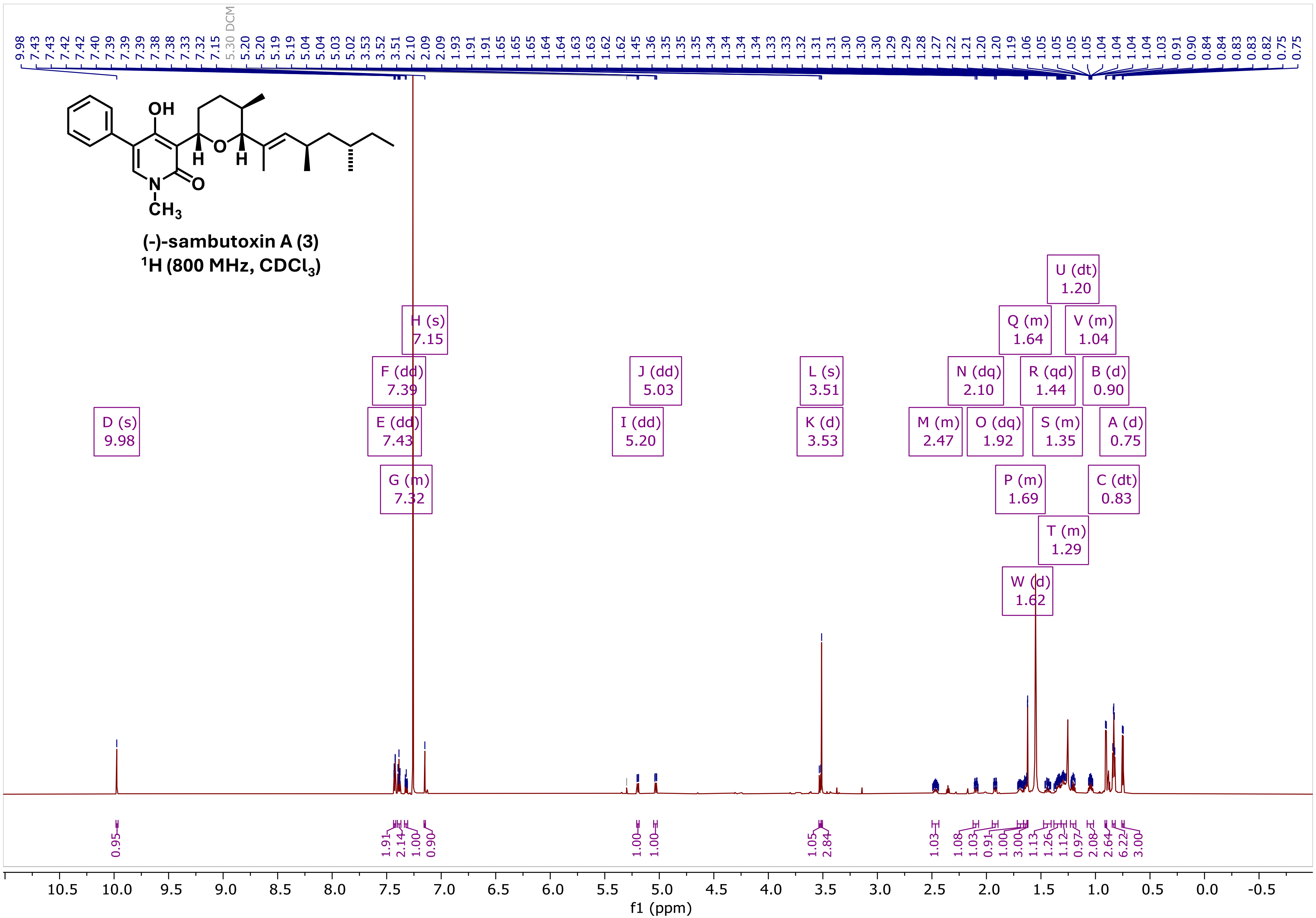
OH
O
N
O
H
H
CH3
(-)-sambutoxin A (3)
1H (800 MHz, CDCl3)
D (s) 9.98
E (dd) 7.43
F (dd) 7.39
G (m) 7.32
H (s) 7.15
I (dd) 5.20
J (dd) 5.03
K (d) 3.53
L (s) 3.51
M (m) 2.47
N (dq) 2.10
O (dq) 1.92
P (m) 1.69
Q (m) 1.64
W (d) 1.62
R (qd) 1.44
S (m) 1.35
T (m) 1.29
U (dt) 1.20
V (m) 1.04
B (d) 0.90
C (dt) 0.83
A (d) 0.75
5.30 DCM
f1 (ppm)

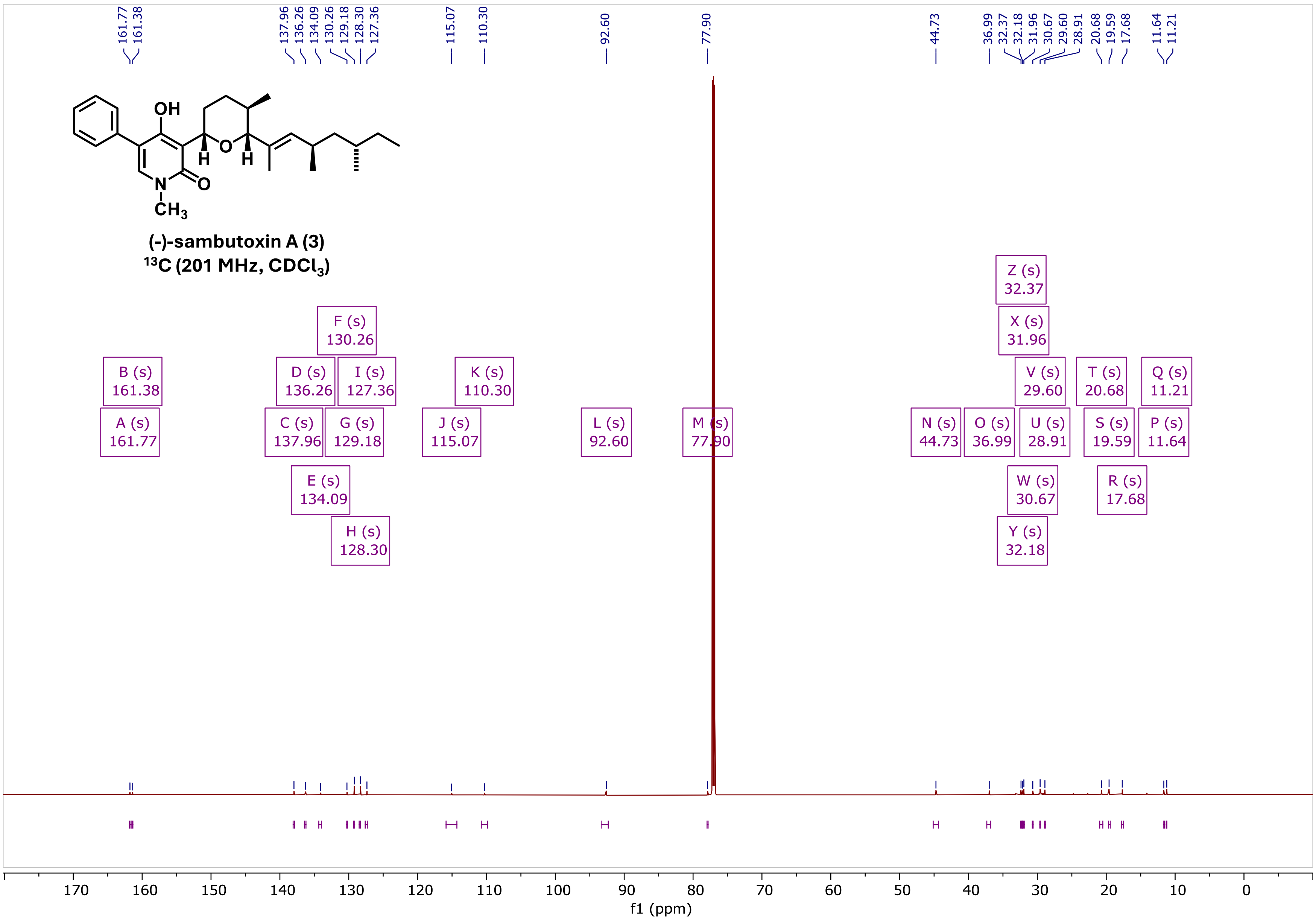

161.77
161.38
137.96
136.26
134.09
130.26
129.18
128.30
127.36
115.07
110.30
92.60
77.90
44.73
36.99
32.37
32.18
31.96
30.67
29.60
28.91
20.68
19.59
17.68
11.64
11.21
OH
O
H
H
N
O
CH3
(-)-sambutoxin A (3)
13C (201 MHz, CDCl3)
A (s) 161.77
B (s) 161.38
C (s) 137.96
D (s) 136.26
E (s) 134.09
F (s) 130.26
G (s) 129.18
H (s) 128.30
I (s) 127.36
J (s) 115.07
K (s) 110.30
L (s) 92.60
M (s) 77.90
N (s) 44.73
O (s) 36.99
P (s) 11.64
Q (s) 11.21
R (s) 17.68
S (s) 19.59
T (s) 20.68
U (s) 28.91
V (s) 29.60
W (s) 30.67
X (s) 31.96
Y (s) 32.18
Z (s) 32.37
170 160 150 140 130 120 110 100 90 80 70 60 50 40 30 20 10 0
f1 (ppm)

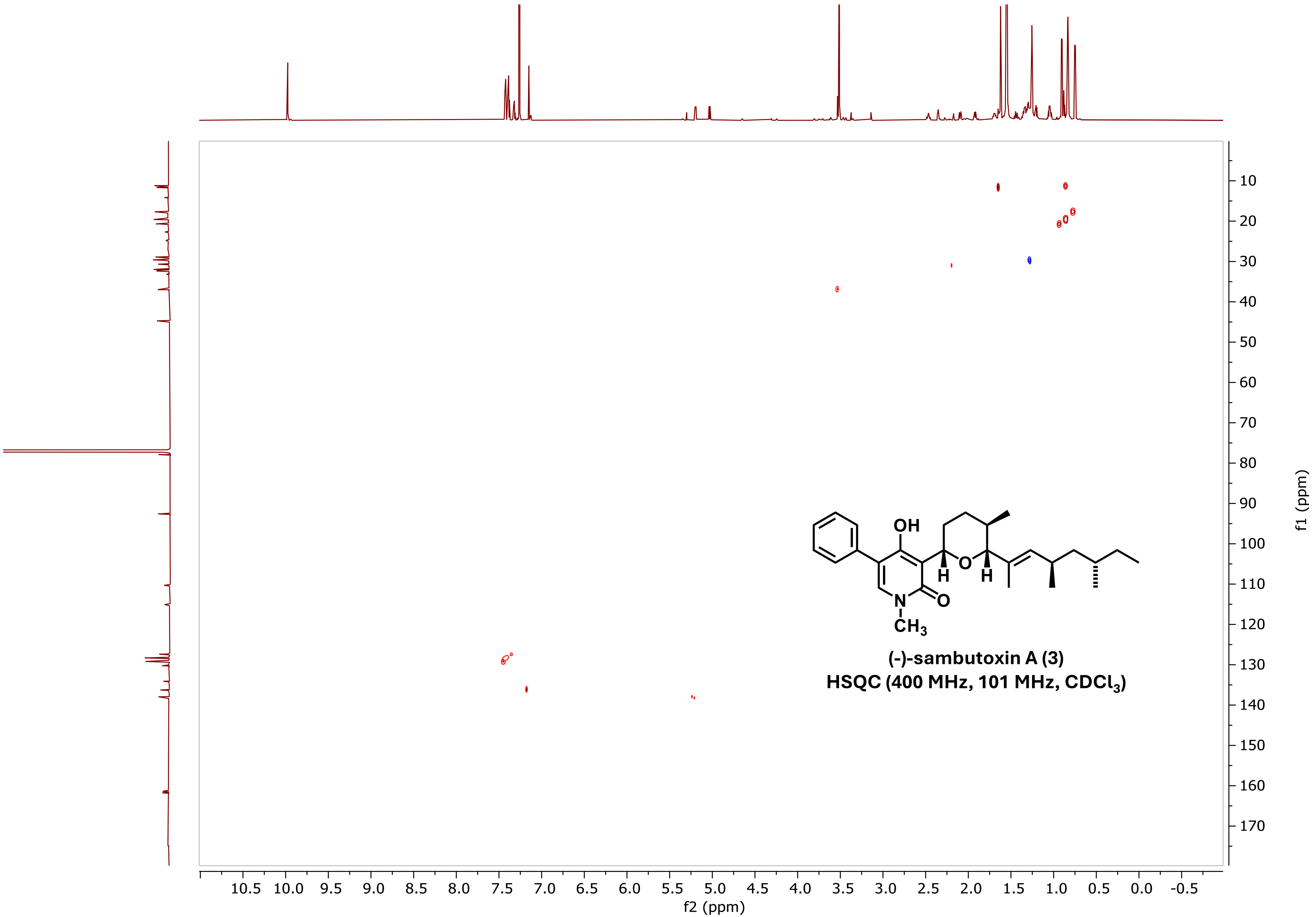

(-)-sambutoxin A (3)
HSQC (400 MHz, 101 MHz, $CDCl_3$)

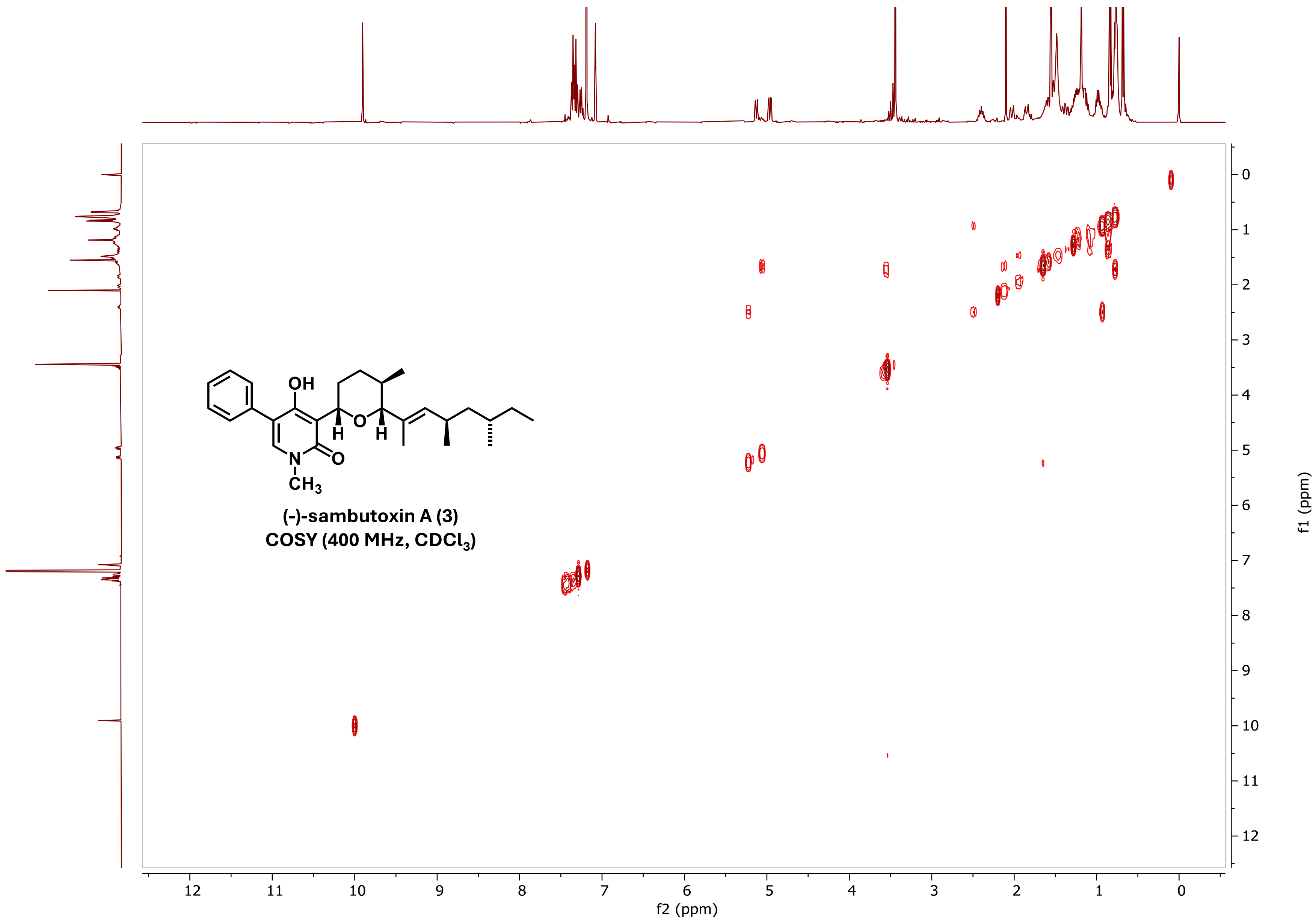
OH
H
O
H
N
O
CH3
(-)-sambutoxin A (3)
COSY (400 MHz, CDCl3)
f2 (ppm)
f1 (ppm)

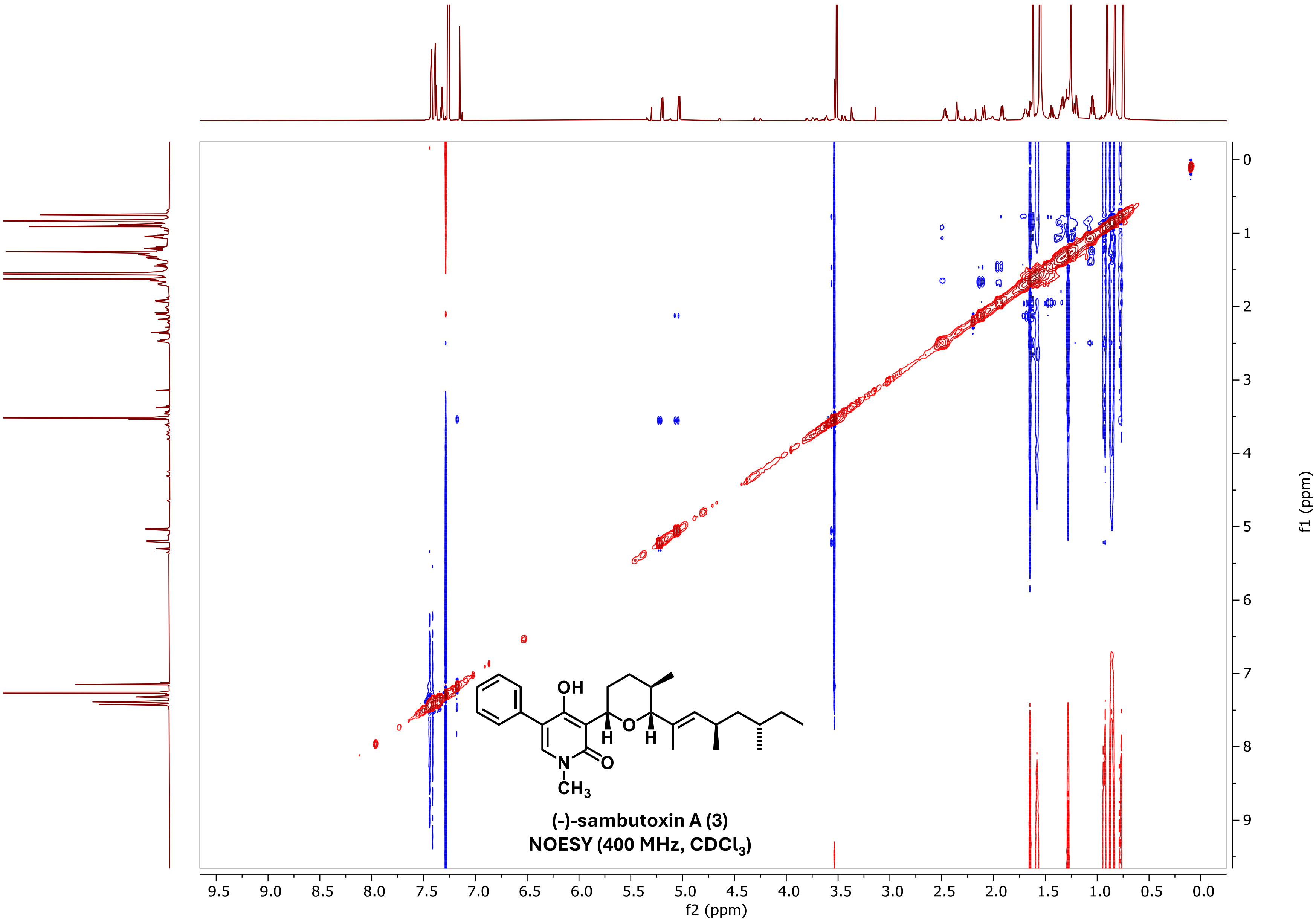

(-)-sambutoxin A (3)
NOESY (400 MHz, $CDCl_3$)

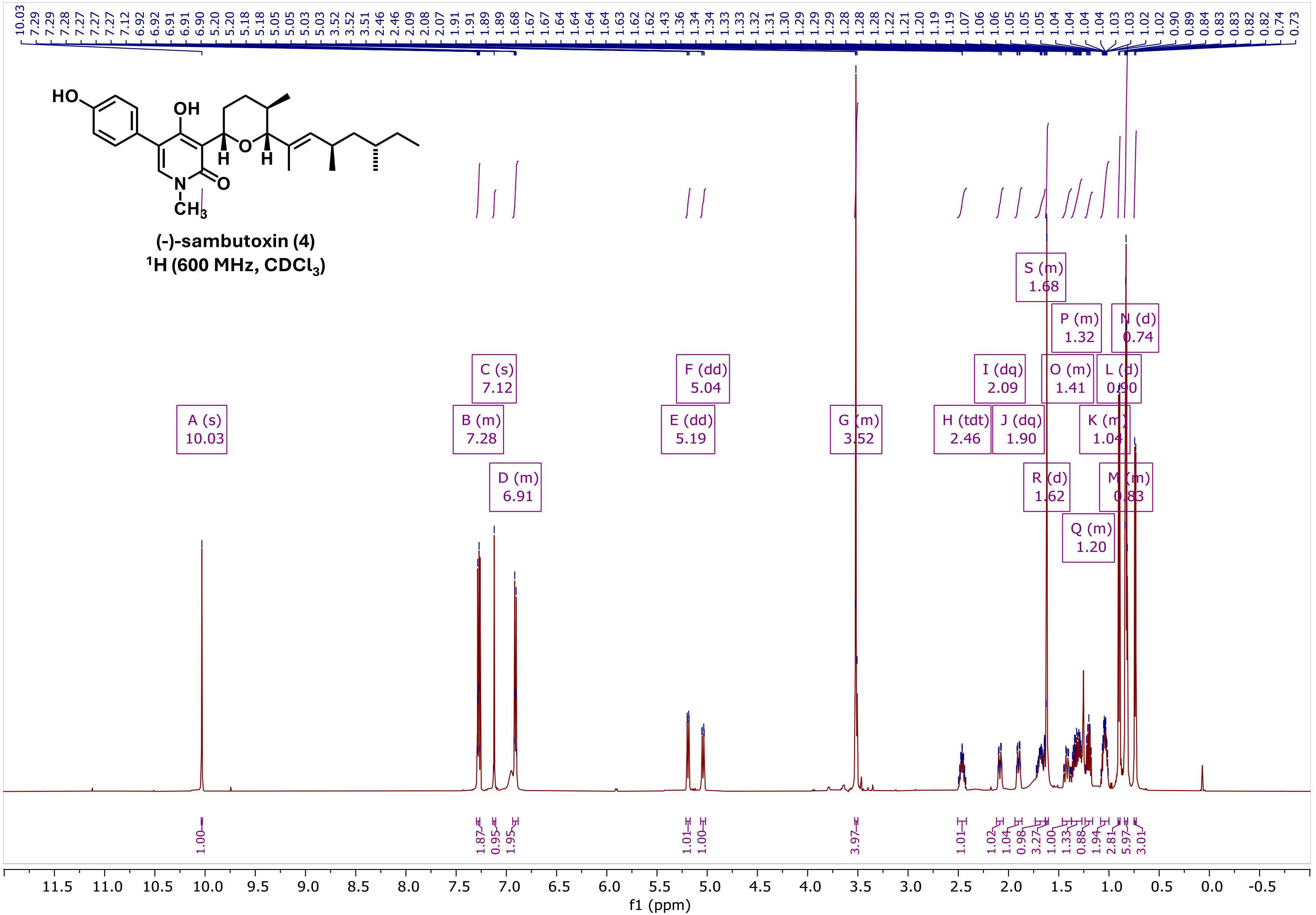

(-)-sambutoxin (4)
^{1}H (600 MHz, CDCl$_3$)
HO
OH
O
H
H
N
CH3
O
10.03 7.29 7.29 7.28 7.27 7.27 7.27 7.12 6.92 6.92 6.91 6.91 6.90 5.20 5.20 5.18 5.18 5.05 5.05 5.03 5.03 3.52 3.52 3.51 2.46 2.46 2.09 2.08 2.07 1.91 1.91 1.89 1.89 1.68 1.67 1.67 1.64 1.64 1.64 1.64 1.63 1.62 1.62 1.43 1.36 1.34 1.34 1.33 1.33 1.32 1.31 1.30 1.29 1.29 1.29 1.28 1.28 1.28 1.28 1.22 1.21 1.20 1.19 1.19 1.07 1.06 1.06 1.05 1.05 1.05 1.04 1.04 1.04 1.04 1.03 1.03 1.02 1.02 0.90 0.89 0.84 0.83 0.83 0.82 0.82 0.74 0.73
A (s) 10.03
B (m) 7.28
C (s) 7.12
D (m) 6.91
E (dd) 5.19
F (dd) 5.04
G (m) 3.52
H (tdt) 2.46
I (dq) 2.09
J (dq) 1.90
K (m) 1.04
L (d) 0.90
M (m) 0.83
N (d) 0.74
O (m) 1.41
P (m) 1.32
Q (m) 1.20
R (d) 1.62
S (m) 1.68
1.00
1.87
0.95
1.95
1.01
1.00
3.97
1.01
1.02
1.04
0.98
3.27
1.00
1.33
0.88
1.94
2.81
5.97
3.01
11.5 11.0 10.5 10.0 9.5 9.0 8.5 8.0 7.5 7.0 6.5 6.0 5.5 5.0 4.5 4.0 3.5 3.0 2.5 2.0 1.5 1.0 0.5 0.0 -0.5
f1 (ppm)

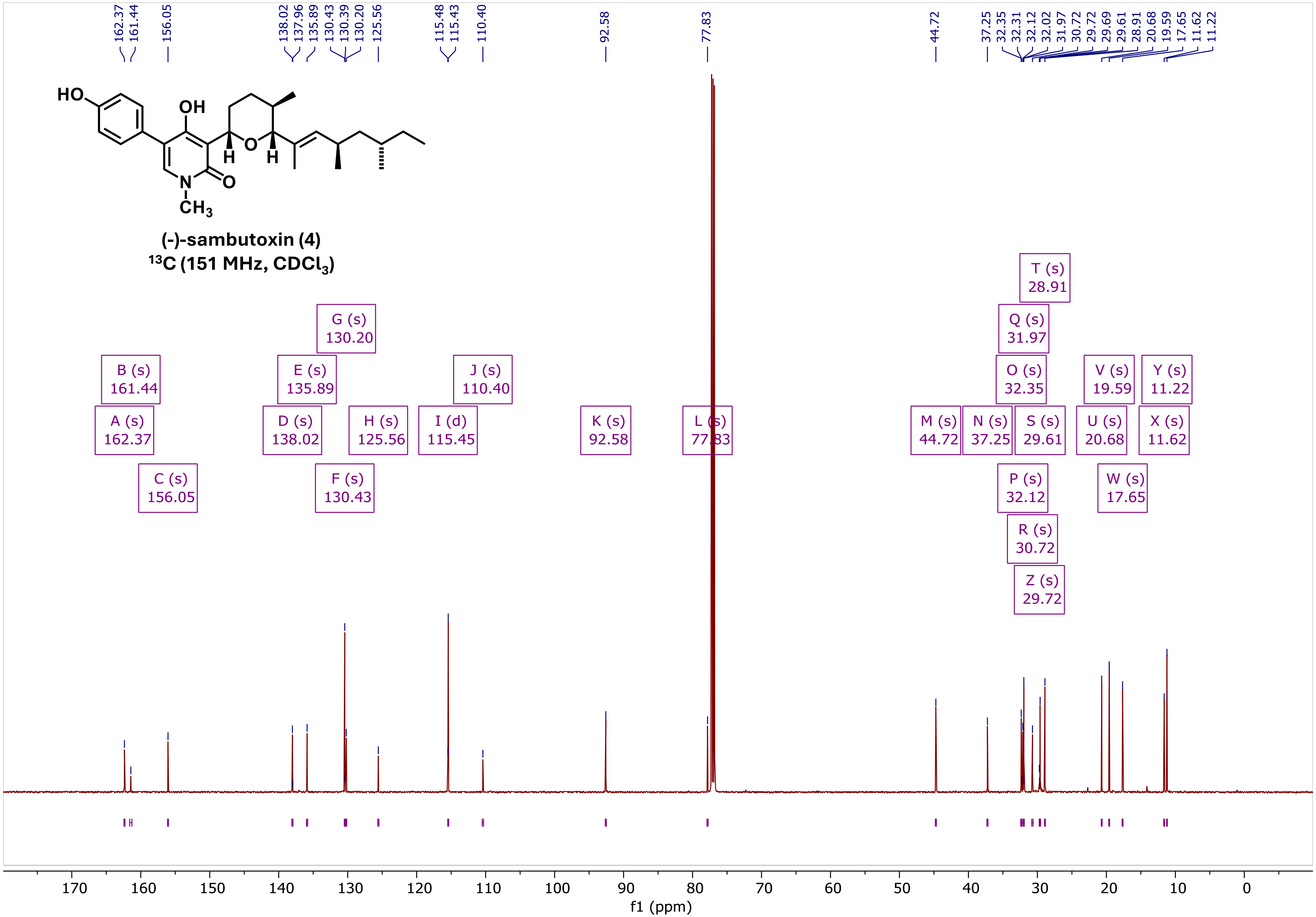
(-)-sambutoxin (4)
^{13}C (151 MHz, $CDCl_3$)
162.37
161.44
156.05
138.02
137.96
135.89
130.43
130.39
130.20
125.56
115.48
115.43
110.40
92.58
77.83
44.72
37.25
32.35
32.31
32.12
32.02
31.97
30.72
29.72
29.69
29.61
28.91
20.68
19.59
17.65
11.62
11.22
A (s) 162.37
B (s) 161.44
C (s) 156.05
D (s) 138.02
E (s) 135.89
F (s) 130.43
G (s) 130.20
H (s) 125.56
I (d) 115.45
J (s) 110.40
K (s) 92.58
L (s) 77.83
M (s) 44.72
N (s) 37.25
O (s) 32.35
P (s) 32.12
Q (s) 31.97
R (s) 30.72
S (s) 29.61
T (s) 28.91
U (s) 20.68
V (s) 19.59
W (s) 17.65
X (s) 11.62
Y (s) 11.22
Z (s) 29.72
170
160
150
140
130
120
110
100
90
80
70
60
50
40
30
20
10
0
f1 (ppm)

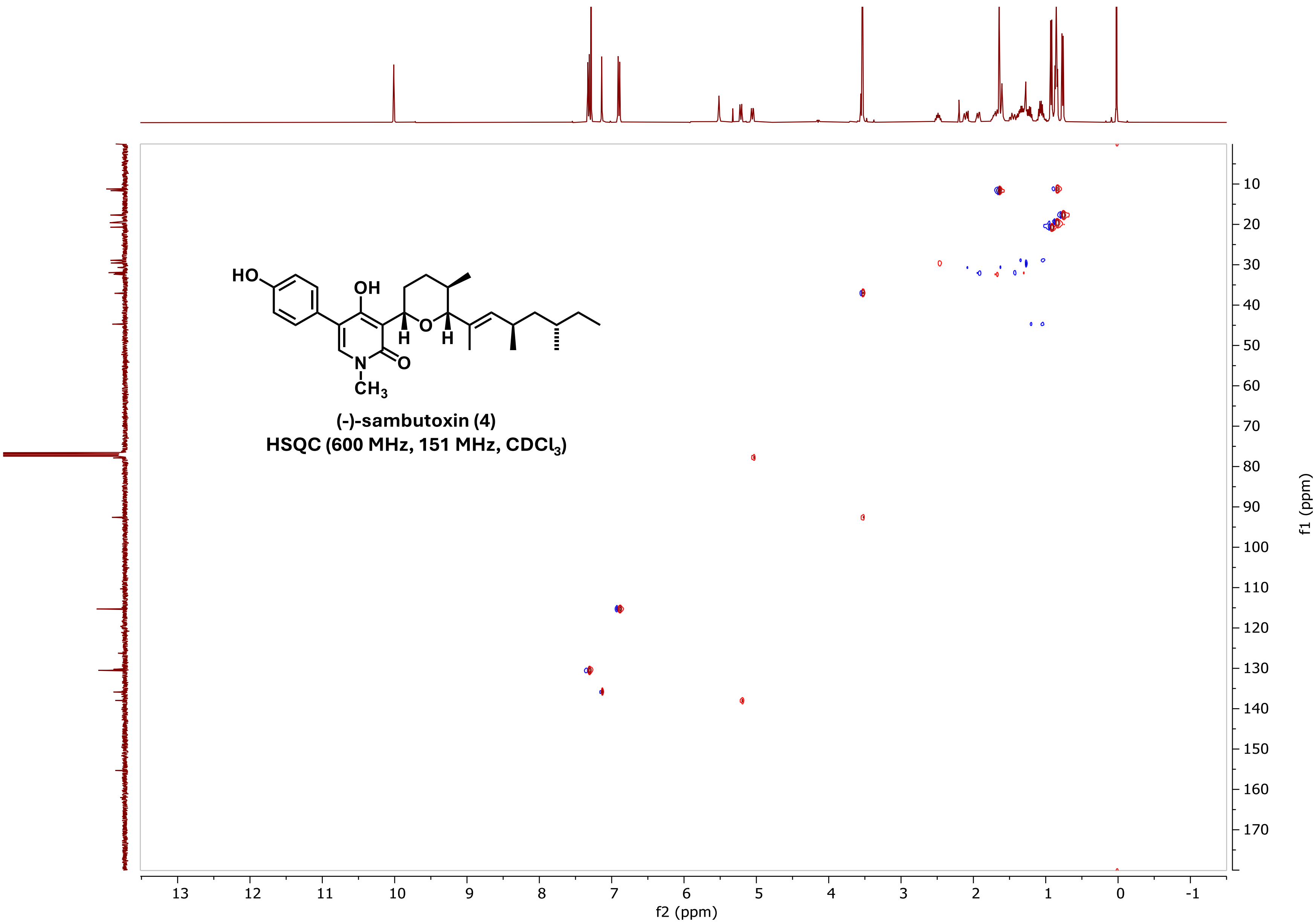
HO
OH
O
H
H
N
O
CH3
(-)-sambutoxin (4)
HSQC (600 MHz, 151 MHz, CDCl3)
f1 (ppm)
f2 (ppm)

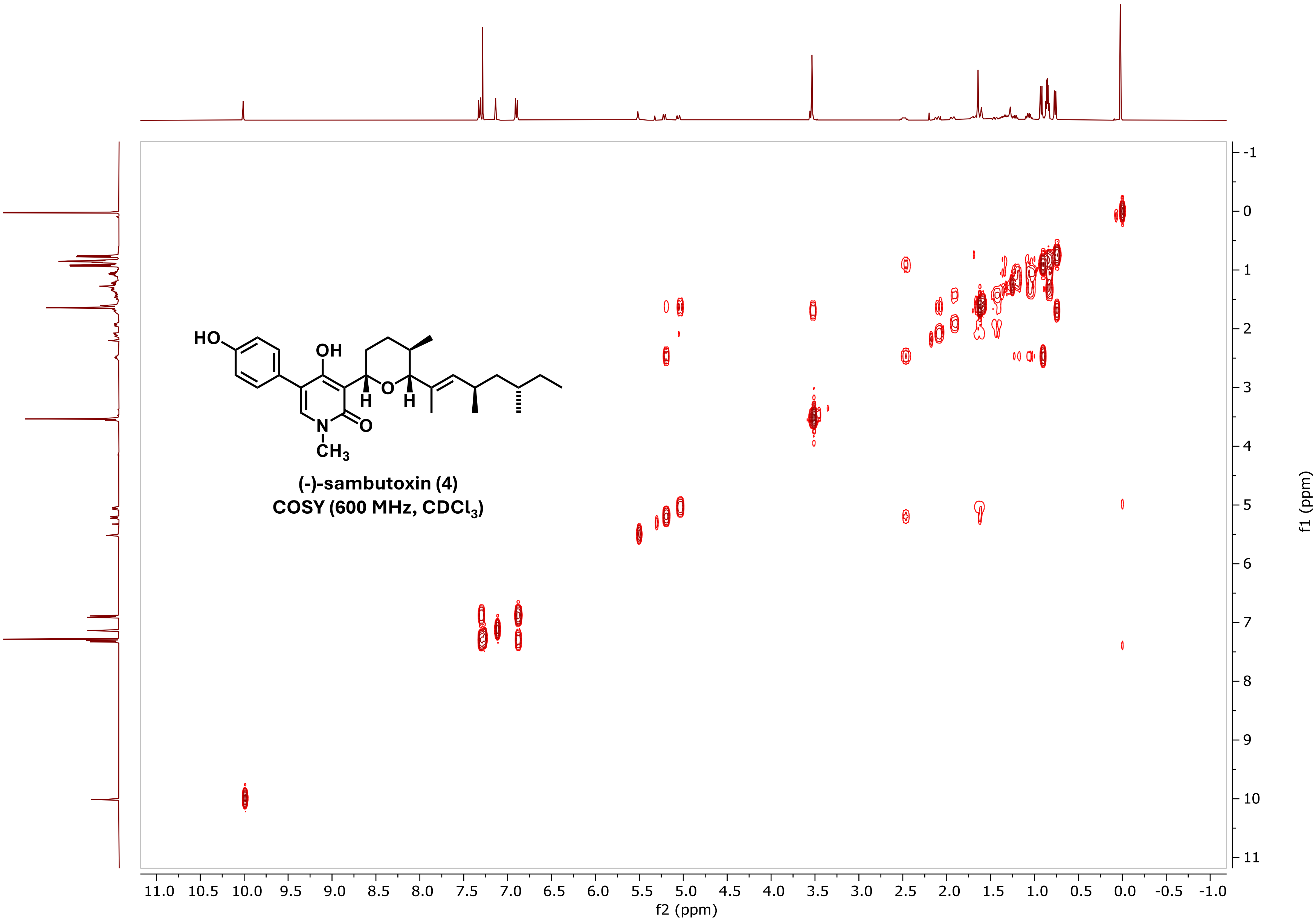

HO
OH
H
O
H
N
O
CH3
(-)-sambutoxin (4)
COSY (600 MHz, CDCl3)
f2 (ppm)
f1 (ppm)

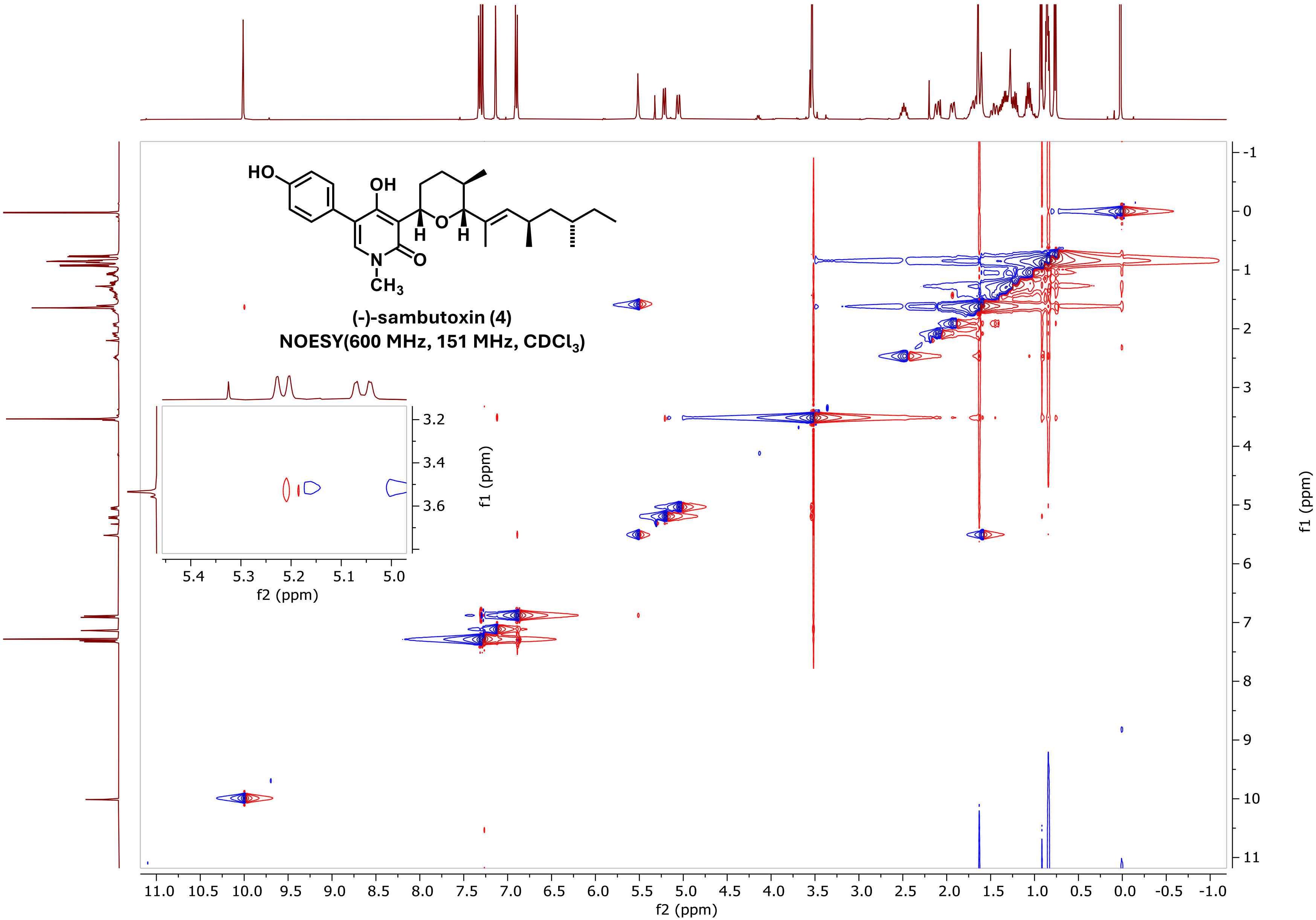
HO
OH
O
H
H
N
CH3
O
(-)-sambutoxin (4)
NOESY(600 MHz, 151 MHz, CDCl3)
f1 (ppm)
f2 (ppm)

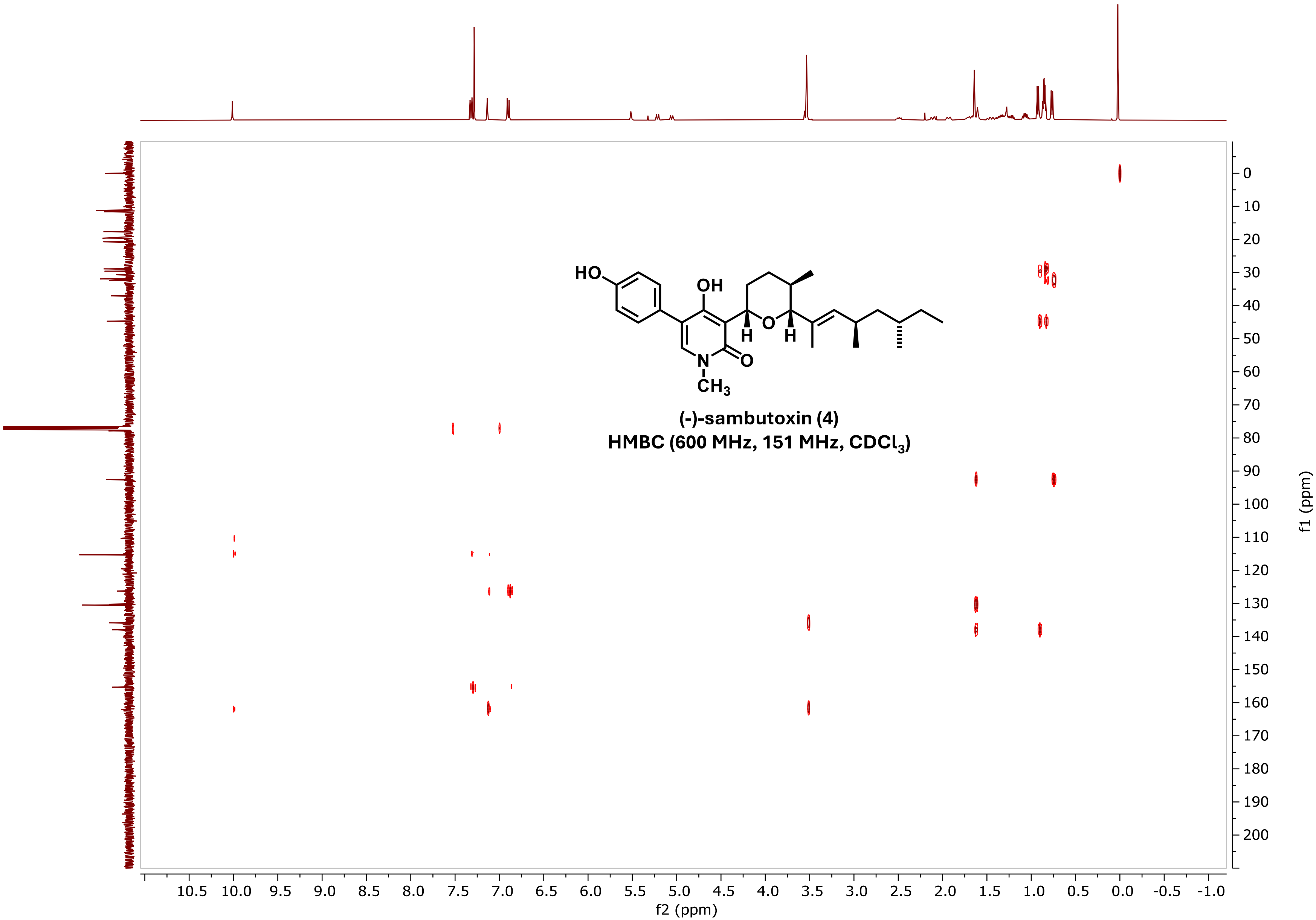

HO
OH
O
H
H
N
O
CH3
(-)-sambutoxin (4)
HMBC (600 MHz, 151 MHz, CDCl3)
f2 (ppm)
f1 (ppm)

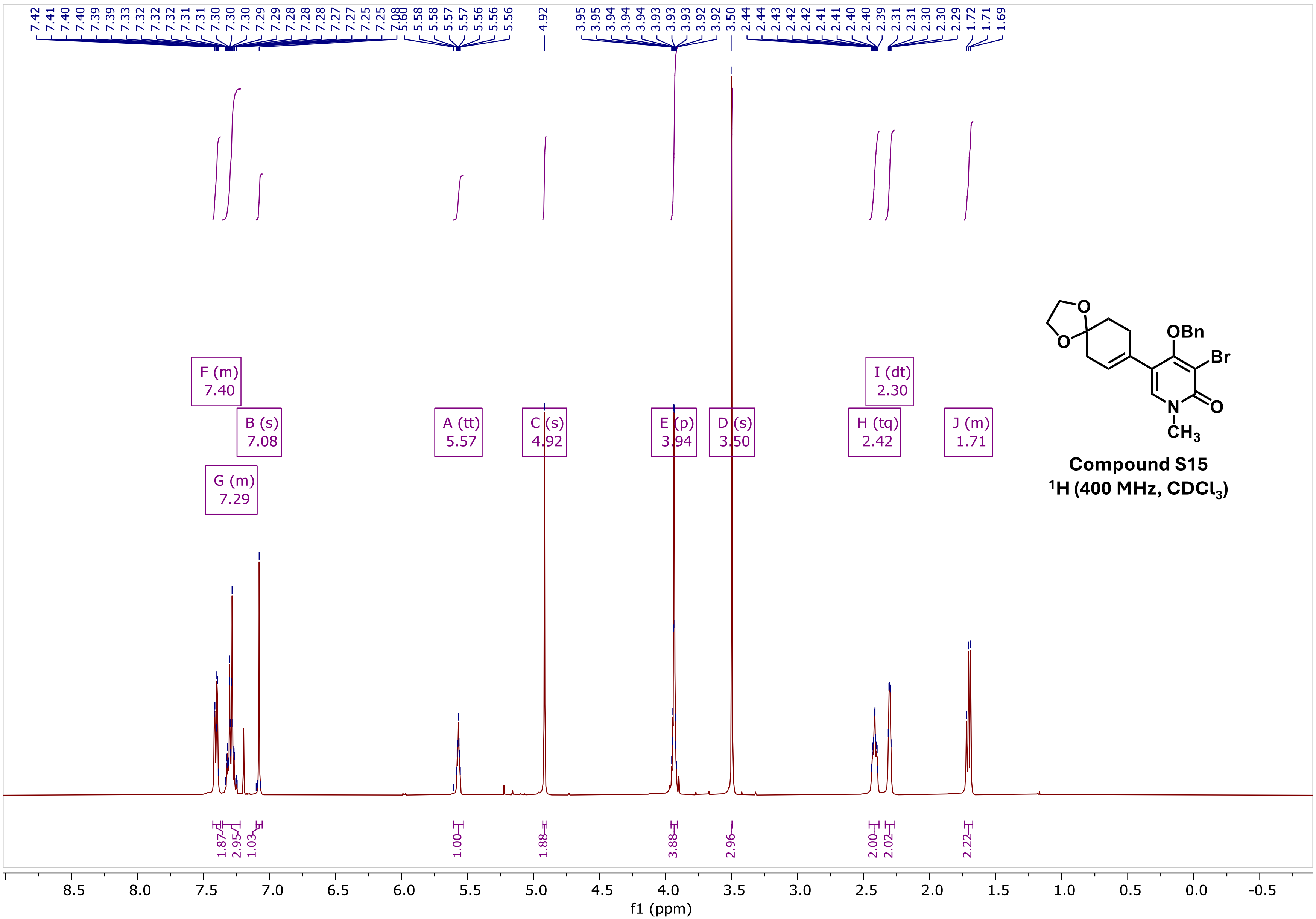

Compound S15
^{1}H (400 MHz, $CDCl_3$)

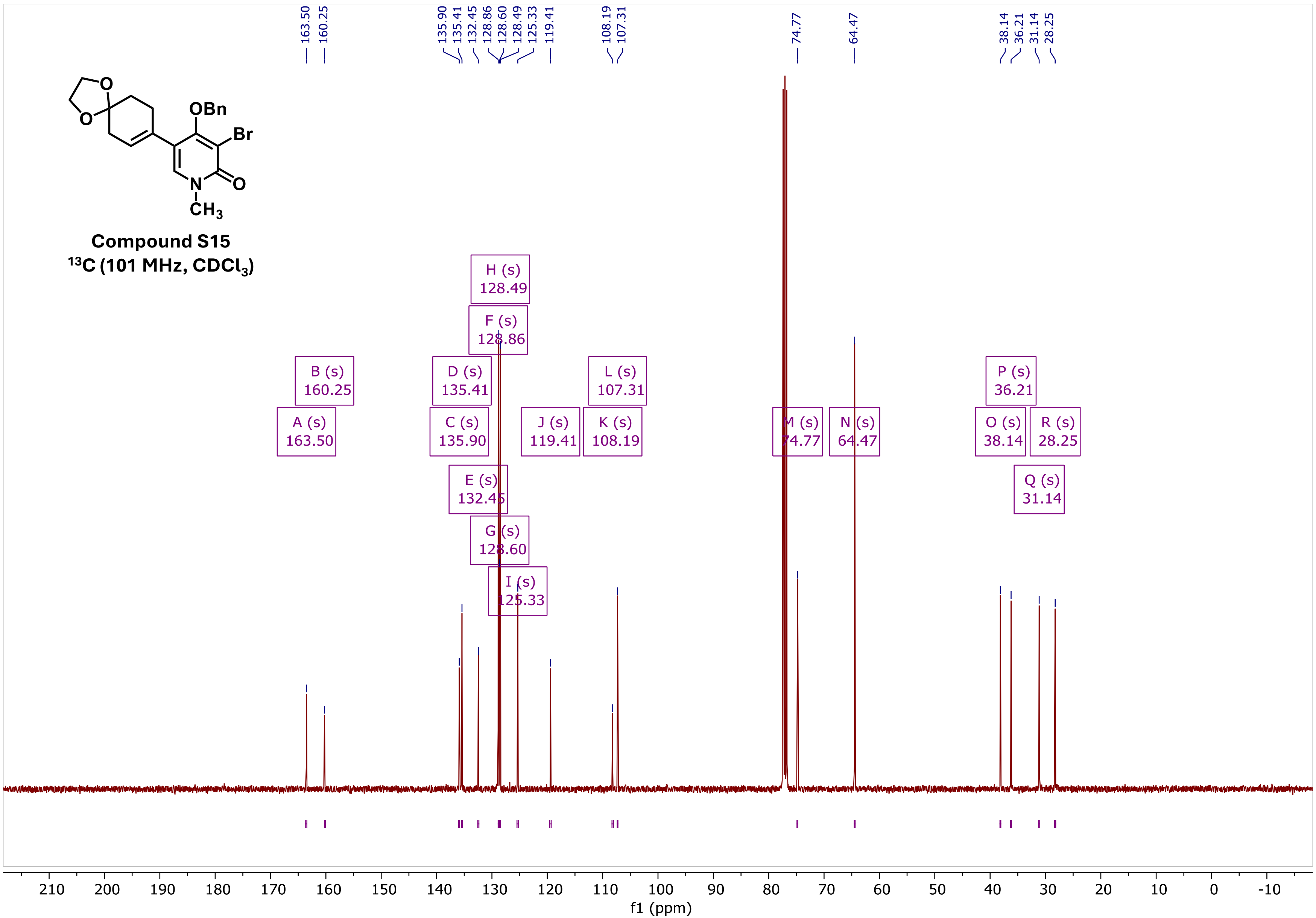

Compound S15

^{13}C (101 MHz, $CDCl_3$)

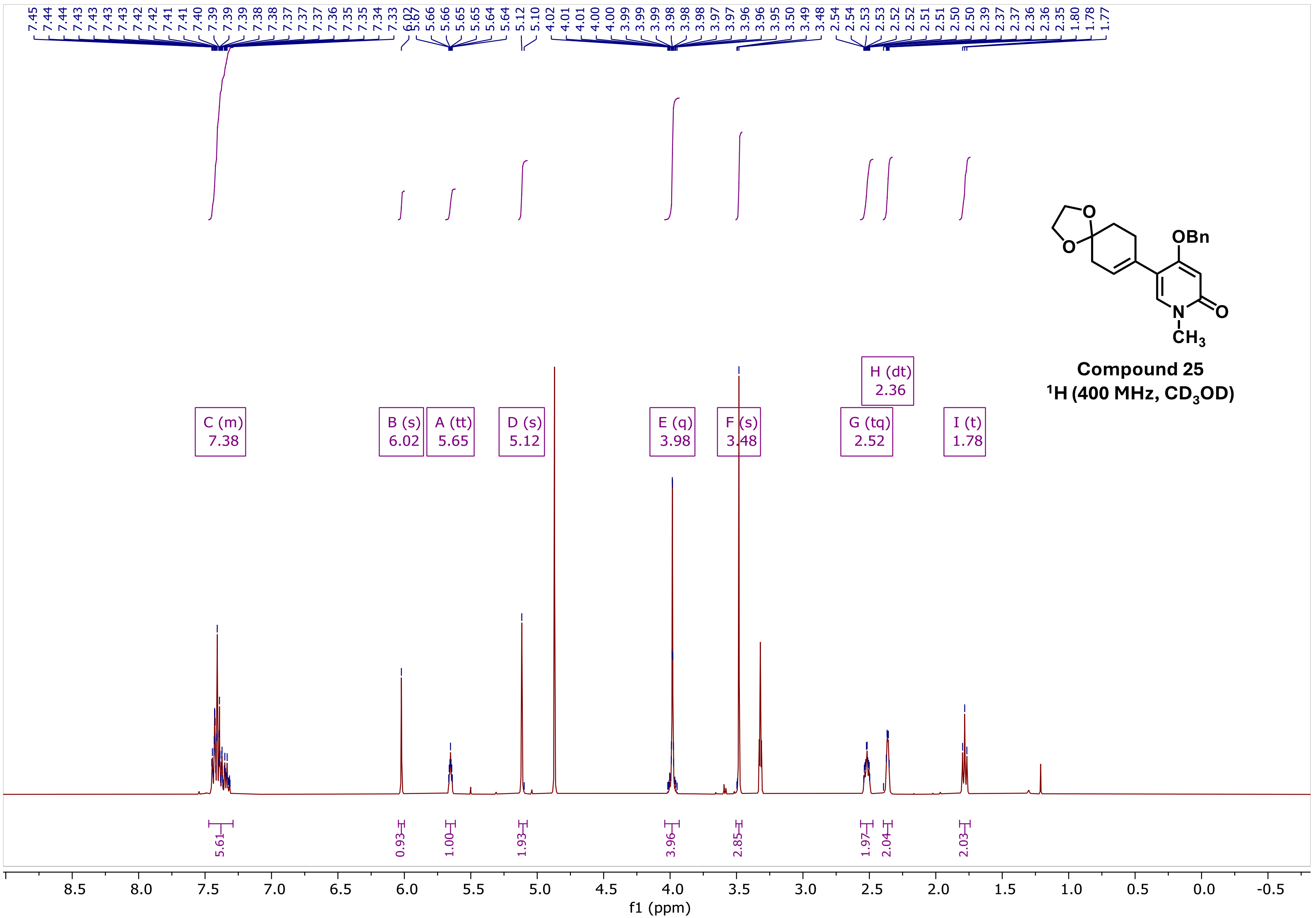
Compound 25
1H (400 MHz, CD3OD)
OBn
O
O
N
O
CH3
C (m) 7.38
B (s) 6.02
A (tt) 5.65
D (s) 5.12
E (q) 3.98
F (s) 3.48
G (tq) 2.52
H (dt) 2.36
I (t) 1.78
5.61
0.93
1.00
1.93
3.96
2.85
1.97
2.04
2.03
f1 (ppm)

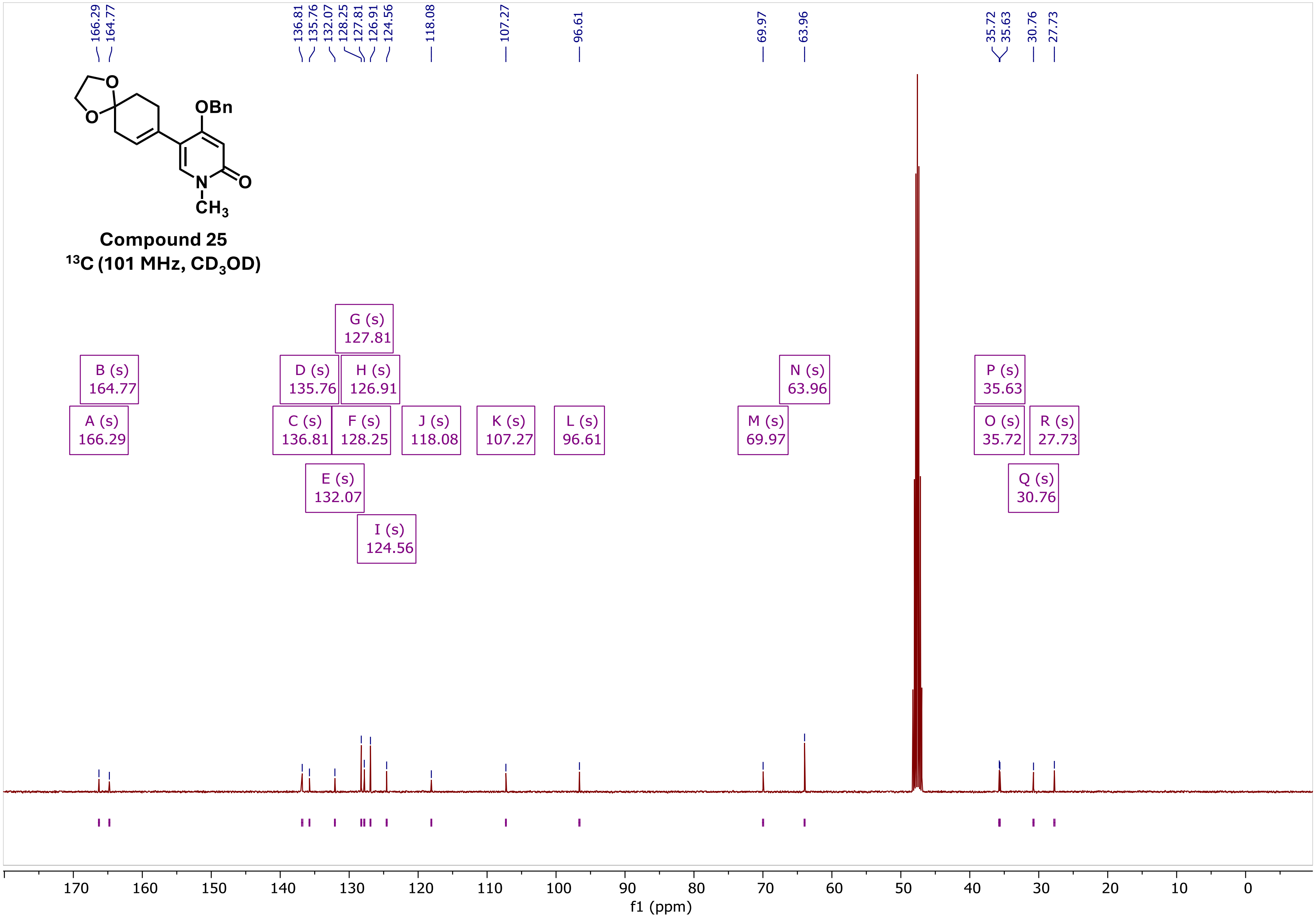
166.29
164.77
136.81
135.76
132.07
128.25
127.81
126.91
124.56
118.08
107.27
96.61
69.97
63.96
35.72
35.63
30.76
27.73
OBn
O
O
O
N
CH_3
Compound 25
^{13}C (101 MHz, CD_3OD)
A (s) 166.29
B (s) 164.77
C (s) 136.81
D (s) 135.76
E (s) 132.07
F (s) 128.25
G (s) 127.81
H (s) 126.91
I (s) 124.56
J (s) 118.08
K (s) 107.27
L (s) 96.61
M (s) 69.97
N (s) 63.96
O (s) 35.72
P (s) 35.63
Q (s) 30.76
R (s) 27.73
170 160 150 140 130 120 110 100 90 80 70 60 50 40 30 20 10 0
f1 (ppm)

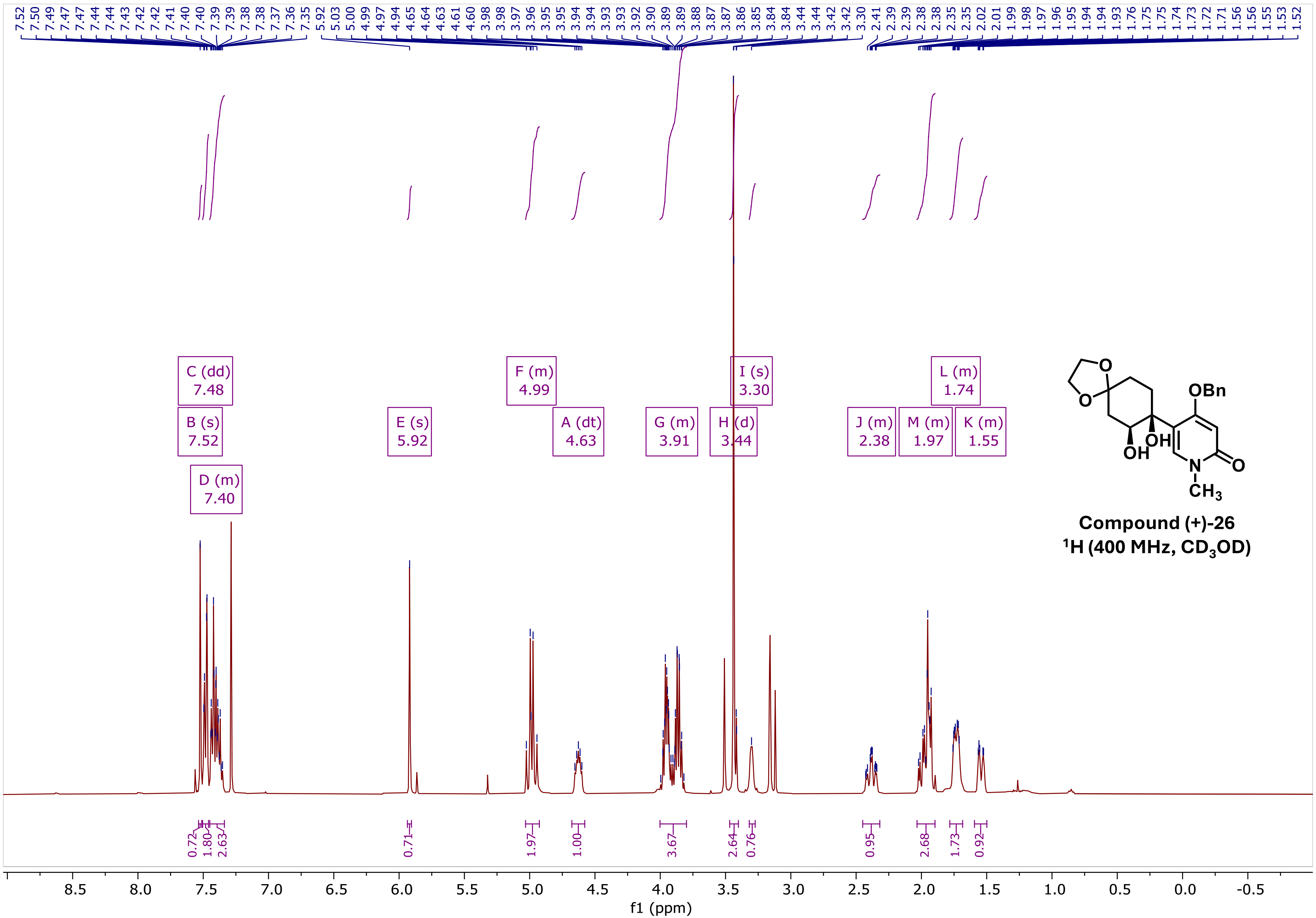

Compound (+)-26
^{1}H (400 MHz, CD_3OD)

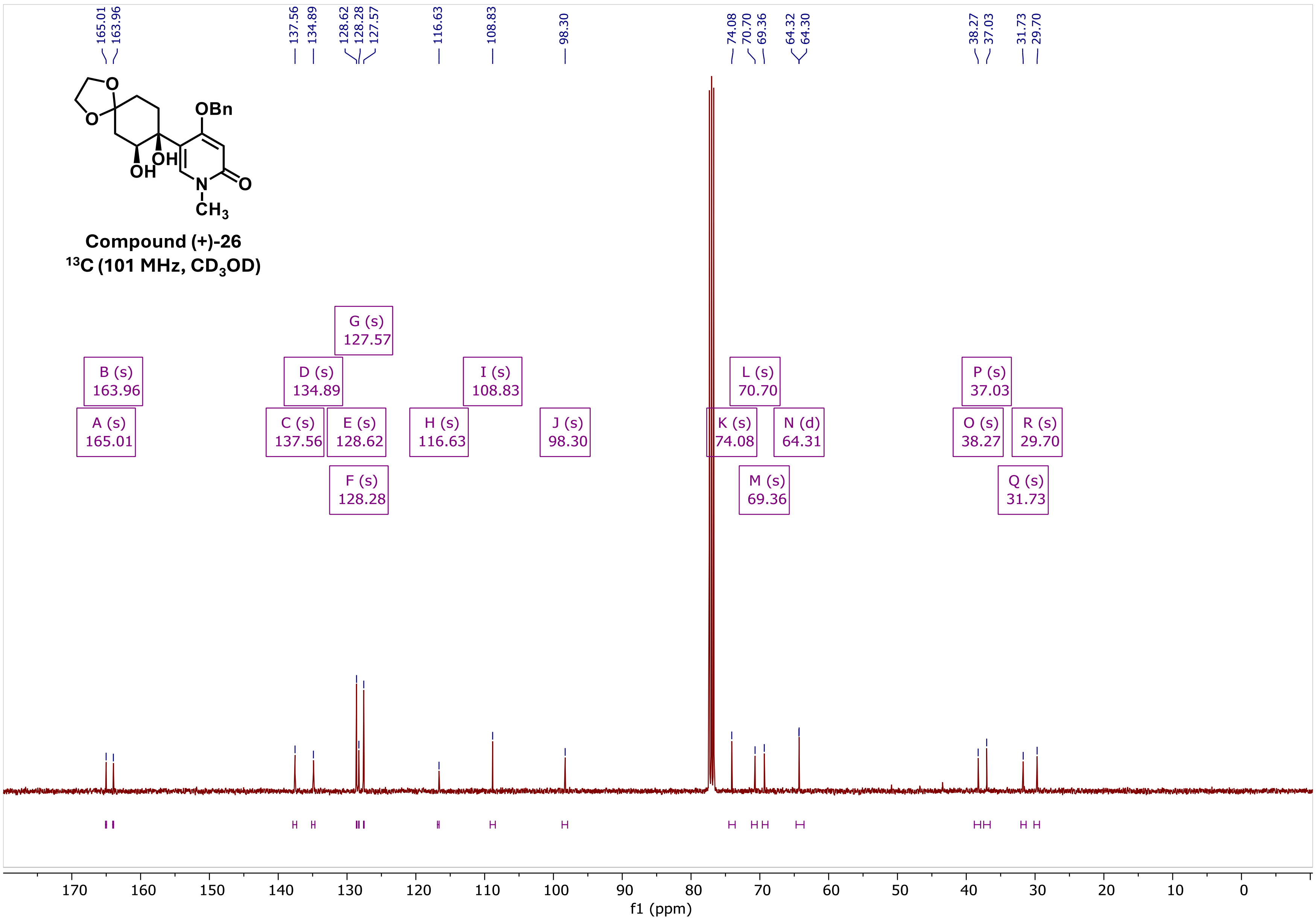

165.01
163.96
137.56
134.89
128.62
128.28
127.57
116.63
108.83
98.30
74.08
70.70
69.36
64.32
64.30
38.27
37.03
31.73
29.70
OBn
O
O
OH
OH
N
O
CH3
Compound (+)-26
13C (101 MHz, CD3OD)
G (s) 127.57
B (s) 163.96
D (s) 134.89
I (s) 108.83
L (s) 70.70
P (s) 37.03
A (s) 165.01
C (s) 137.56
E (s) 128.62
H (s) 116.63
J (s) 98.30
K (s) 74.08
N (d) 64.31
O (s) 38.27
R (s) 29.70
F (s) 128.28
M (s) 69.36
Q (s) 31.73
170
160
150
140
130
120
110
100
90
80
70
60
50
40
30
20
10
0
f1 (ppm)

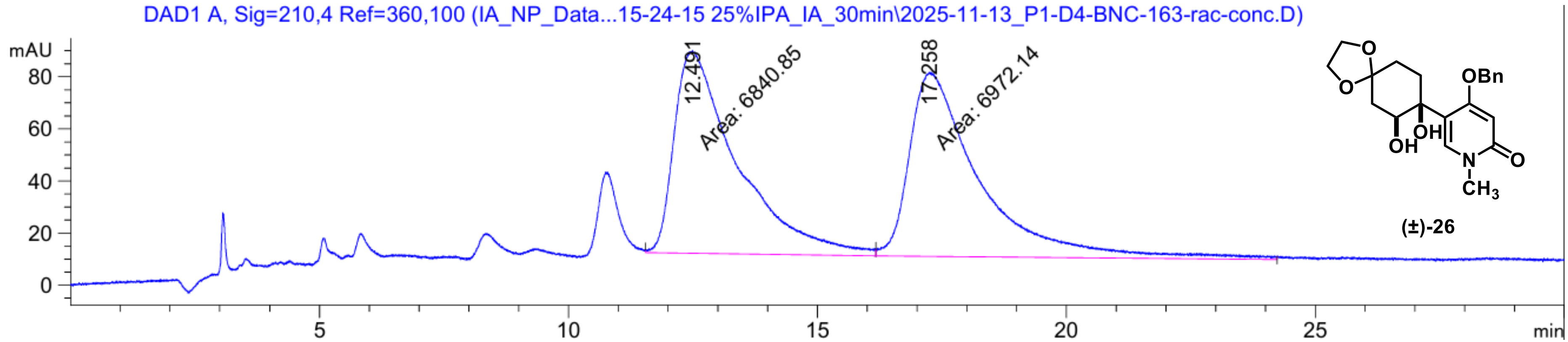

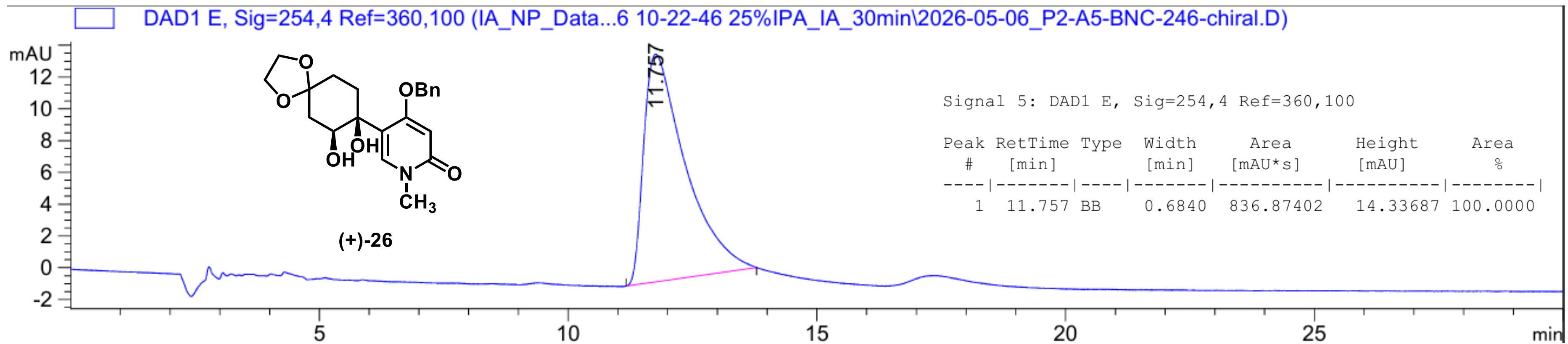

Signal 5: DAD1 E, Sig=254,4 Ref=360,100

Peak #	RetTime [min]	Type	Width [min]	Area [mAU*s]	Height [mAU]	Area %
1	11.757	BB	0.6840	836.87402	14.33687	100.0000

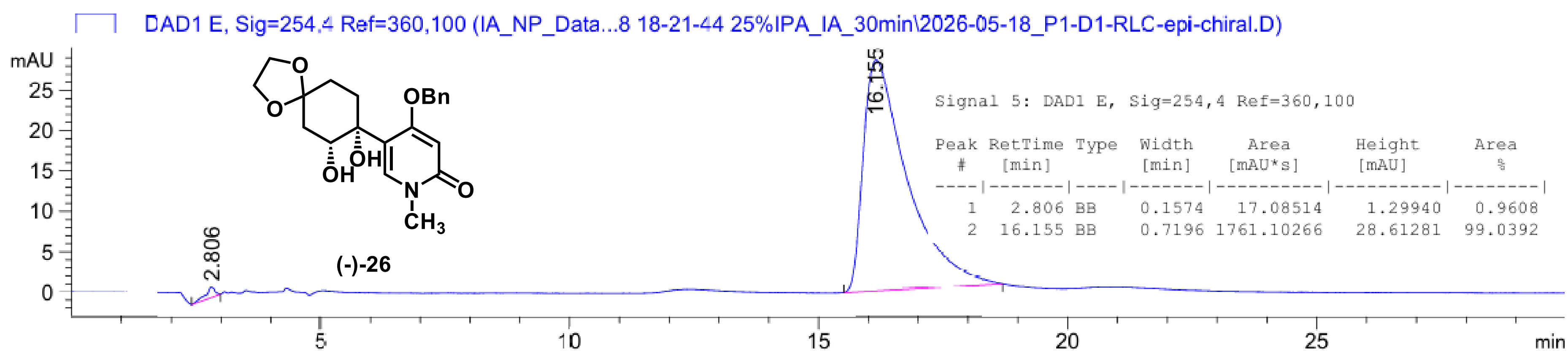

Signal 5: DAD1 E, Sig=254,4 Ref=360,100

Peak #	RetTime [min]	Type	Width [min]	Area [mAU*s]	Height [mAU]	Area %
1	2.806	BB	0.1574	17.08514	1.29940	0.9608
2	16.155	BB	0.7196	1761.10266	28.61281	99.0392

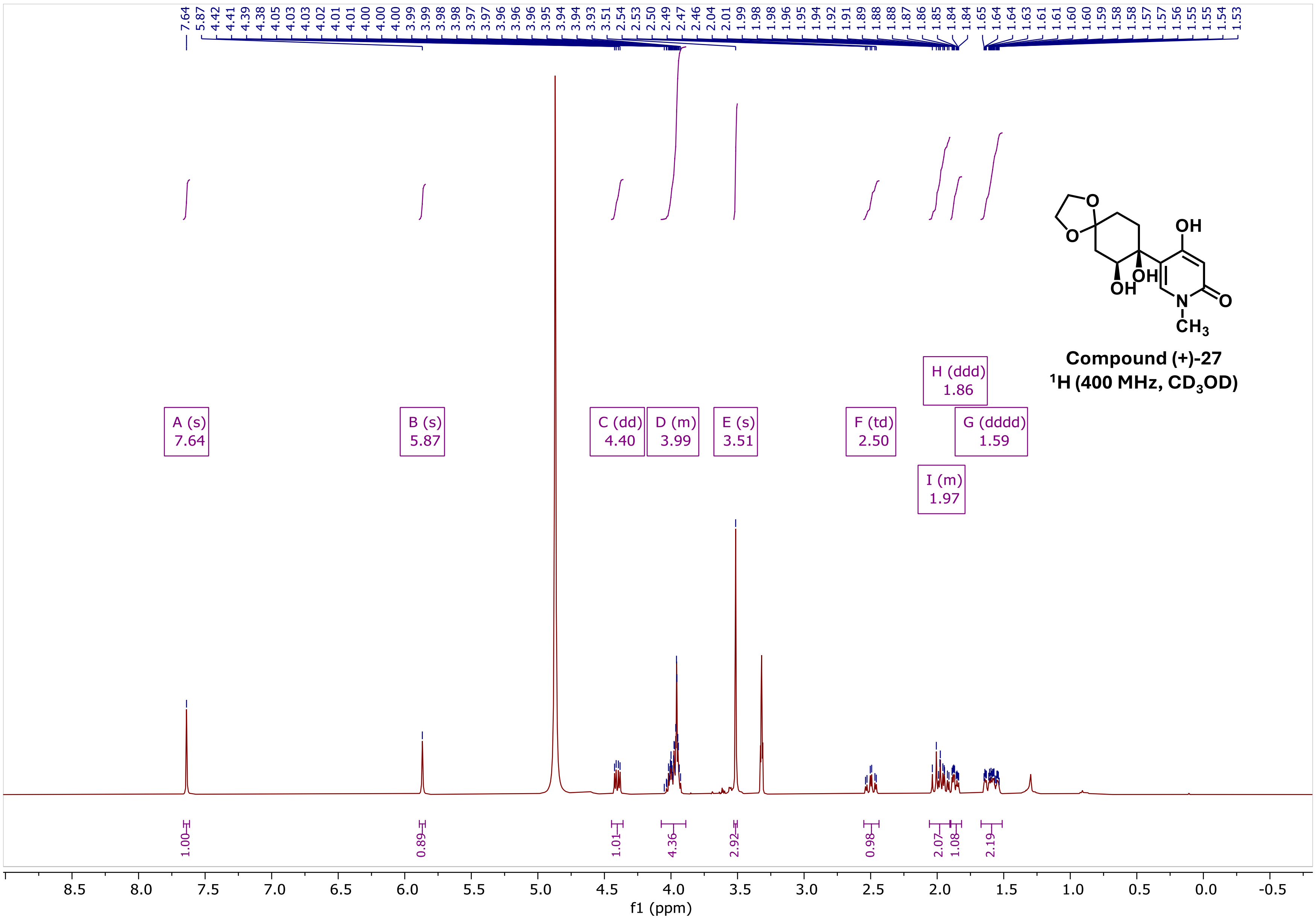
Compound (+)-27
^1H (400 MHz, CD_3OD)
A (s) 7.64
B (s) 5.87
C (dd) 4.40
D (m) 3.99
E (s) 3.51
F (td) 2.50
H (ddd) 1.86
G (dddd) 1.59
I (m) 1.97
1.00
0.89
1.01
4.36
2.92
0.98
2.07
1.08
2.19
f1 (ppm)

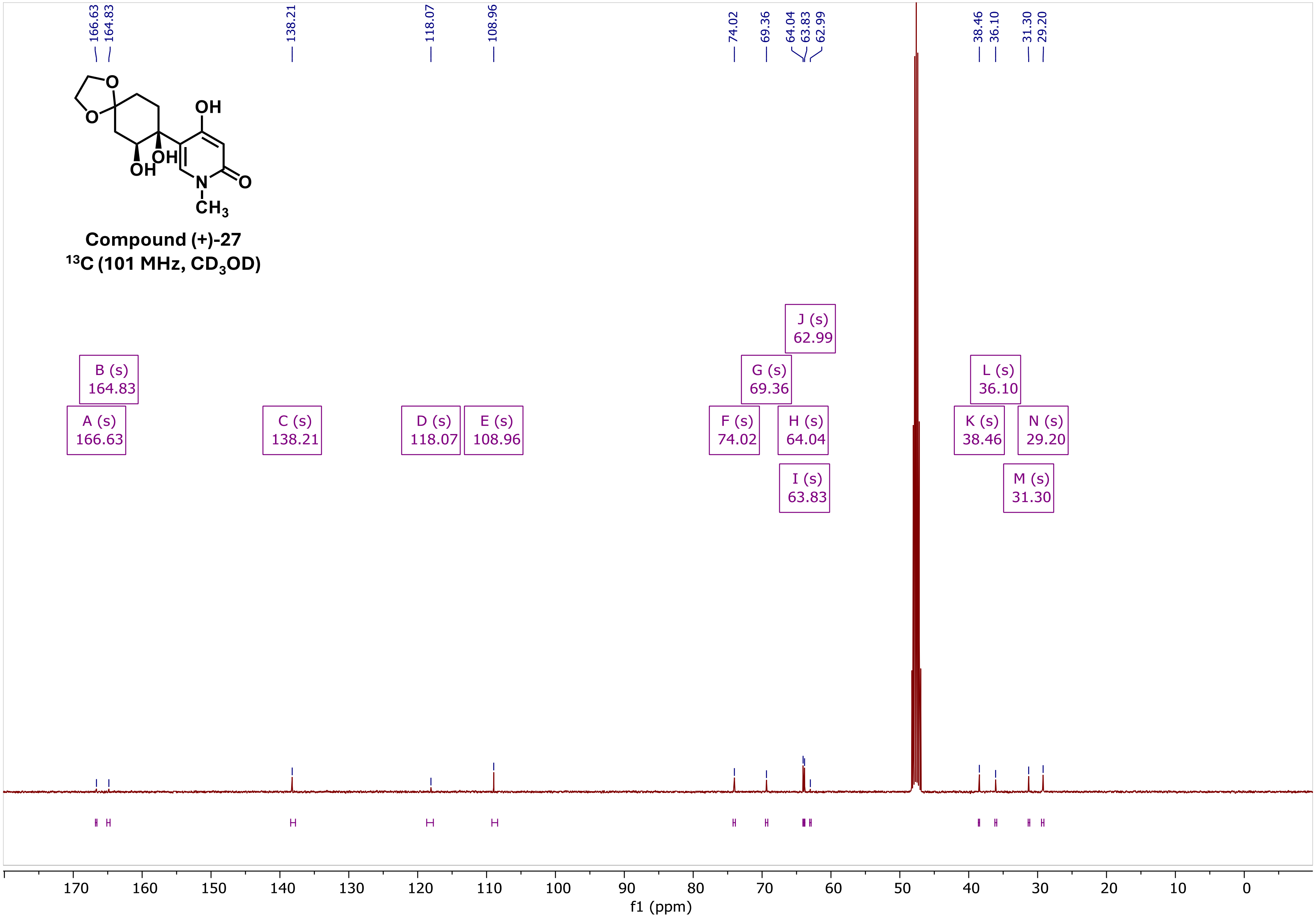

166.63
164.83
138.21
118.07
108.96
74.02
69.36
64.04
63.83
62.99
38.46
36.10
31.30
29.20
OH
O
O
OH
OH
N
O
CH3
Compound (+)-27
13C (101 MHz, CD3OD)
A (s) 166.63
B (s) 164.83
C (s) 138.21
D (s) 118.07
E (s) 108.96
F (s) 74.02
G (s) 69.36
H (s) 64.04
I (s) 63.83
J (s) 62.99
K (s) 38.46
L (s) 36.10
M (s) 31.30
N (s) 29.20
170 160 150 140 130 120 110 100 90 80 70 60 50 40 30 20 10 0
f1 (ppm)

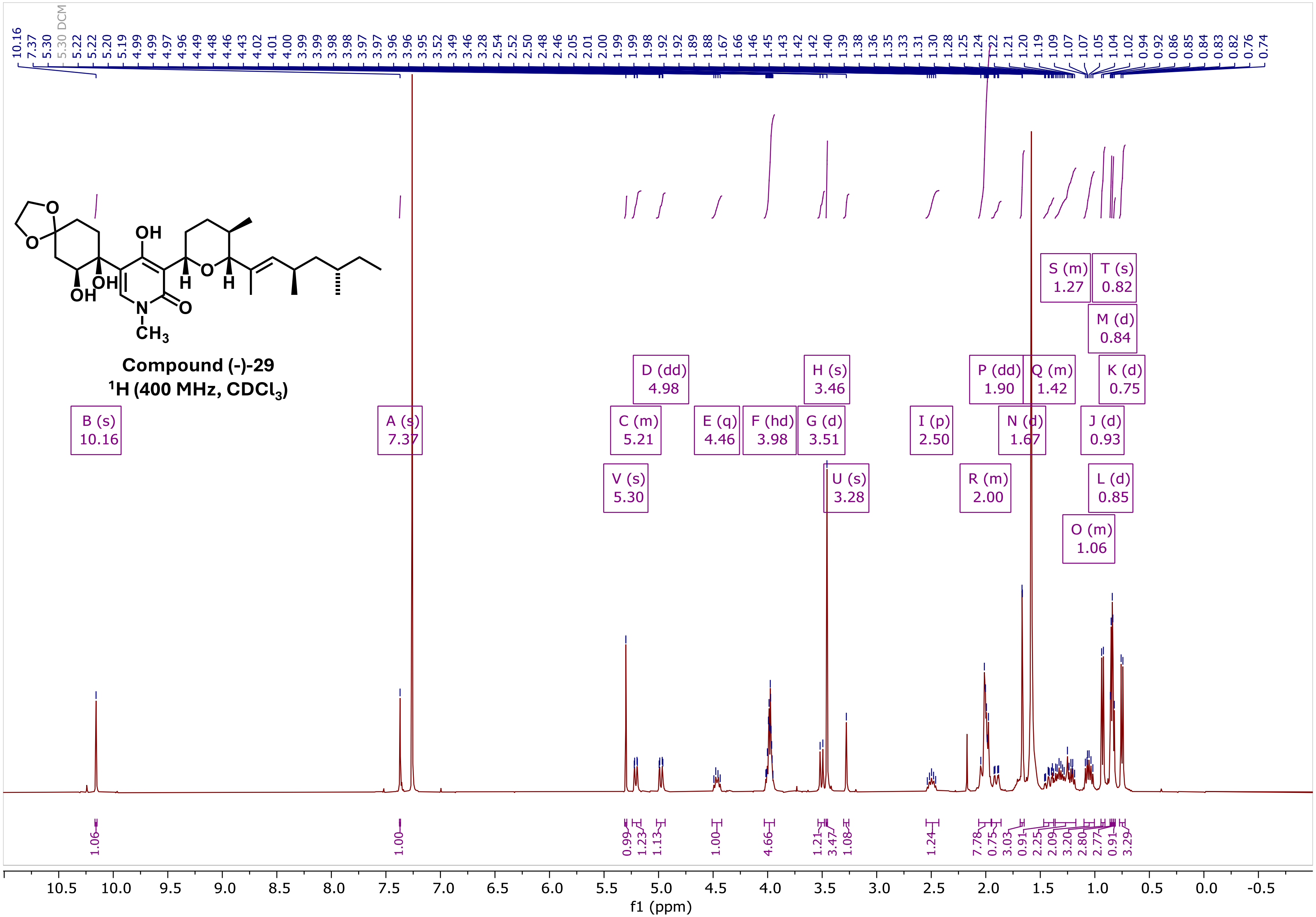
Compound (-)-29
^{1}H (400 MHz, $CDCl_3$)
f1 (ppm)

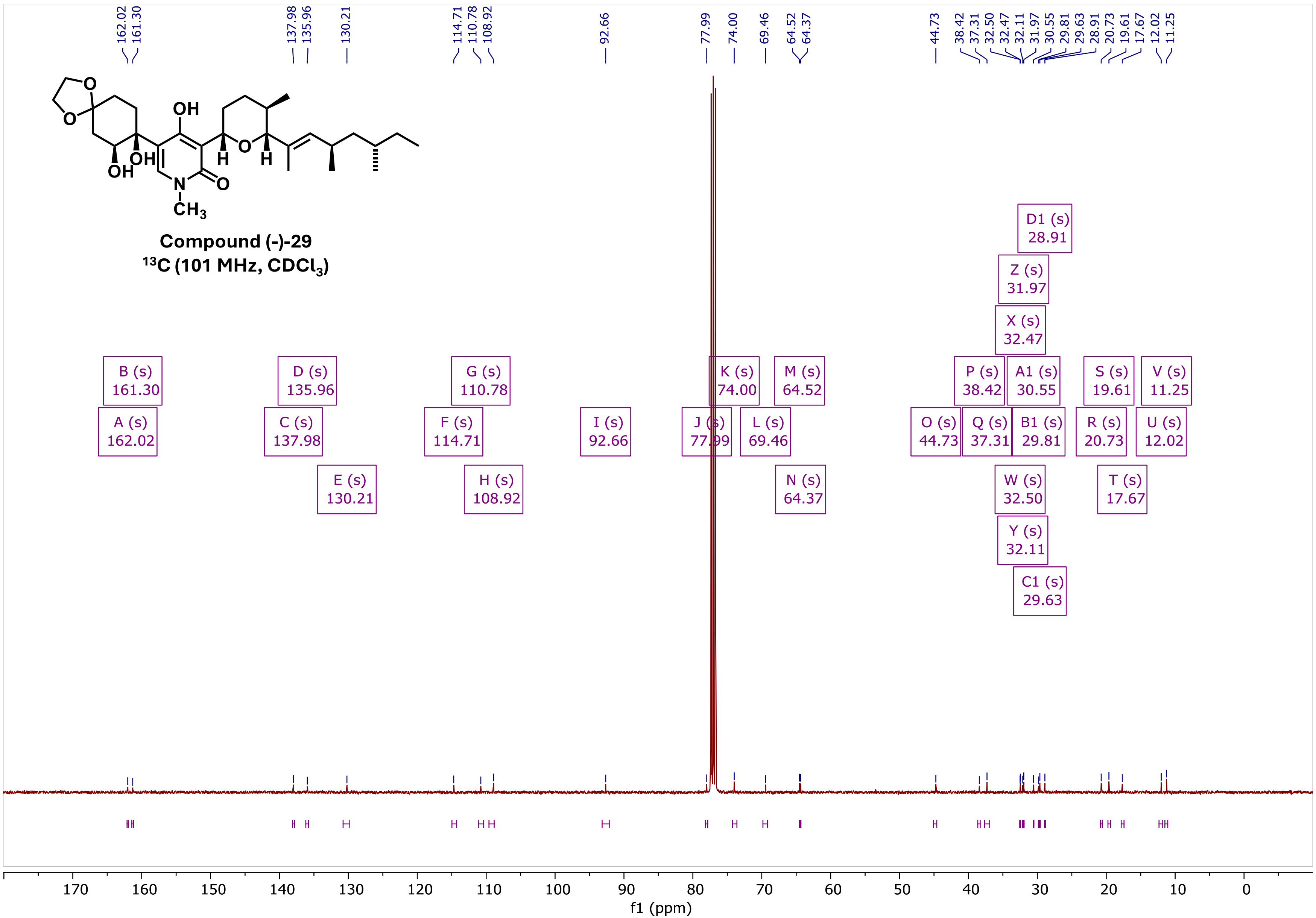
Compound (-)-29
^{13}C (101 MHz, $CDCl_3$)
162.02 161.30 137.98 135.96 130.21 114.71 110.78 108.92 92.66 77.99 74.00 69.46 64.52 64.37 44.73 38.42 37.31 32.50 32.47 32.11 31.97 30.55 29.81 29.63 28.91 20.73 19.61 17.67 12.02 11.25
A (s) 162.02
B (s) 161.30
C (s) 137.98
D (s) 135.96
E (s) 130.21
F (s) 114.71
G (s) 110.78
H (s) 108.92
I (s) 92.66
J (s) 77.99
K (s) 74.00
L (s) 69.46
M (s) 64.52
N (s) 64.37
O (s) 44.73
P (s) 38.42
Q (s) 37.31
R (s) 20.73
S (s) 19.61
T (s) 17.67
U (s) 12.02
V (s) 11.25
W (s) 32.50
X (s) 32.47
Y (s) 32.11
Z (s) 31.97
A1 (s) 30.55
B1 (s) 29.81
C1 (s) 29.63
D1 (s) 28.91
170 160 150 140 130 120 110 100 90 80 70 60 50 40 30 20 10 0
f1 (ppm)

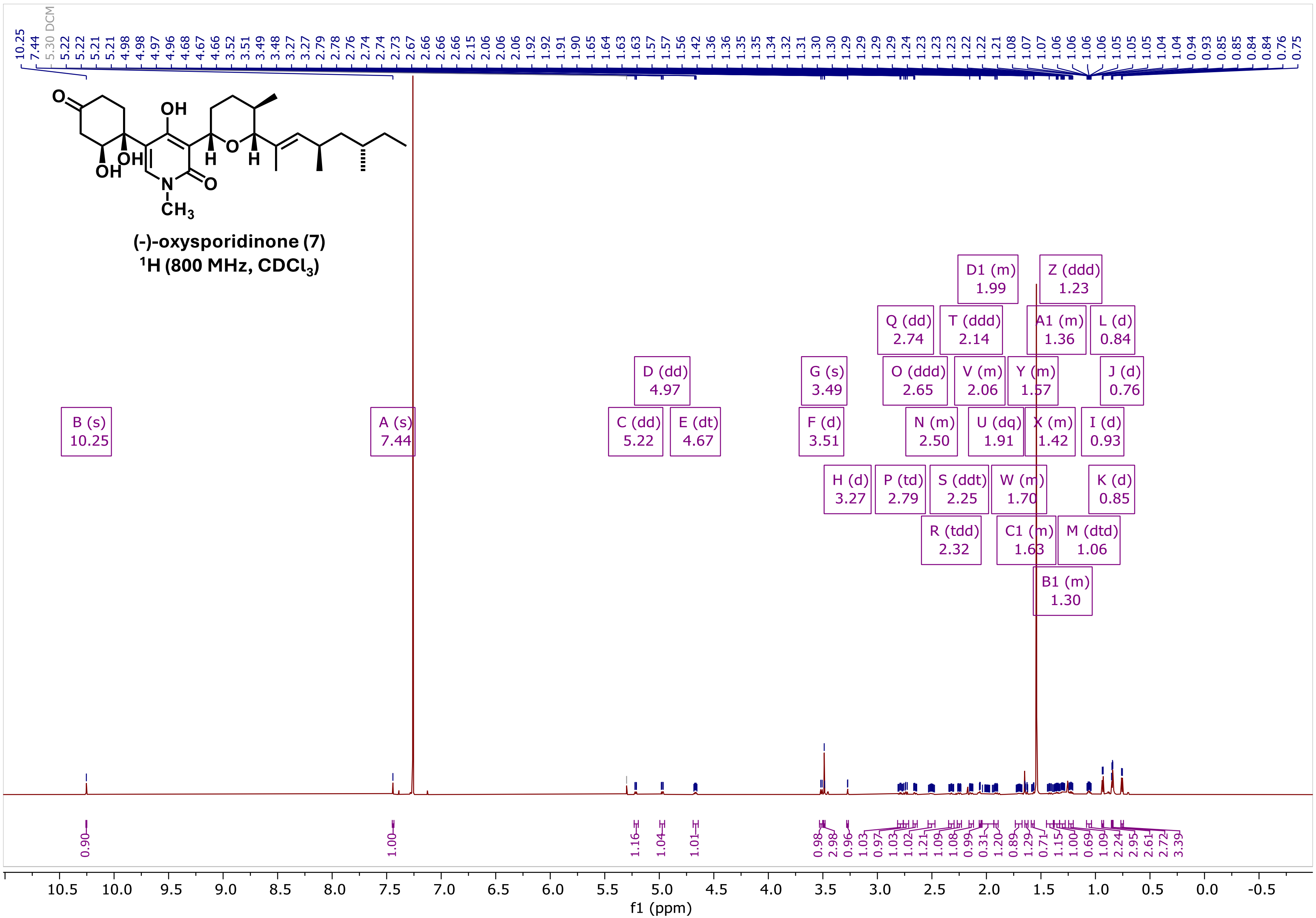

(-)-oxysporidinone (7)
1H (800 MHz, CDCl3)
B (s) 10.25
A (s) 7.44
D (dd) 4.97
C (dd) 5.22
E (dt) 4.67
G (s) 3.49
F (d) 3.51
H (d) 3.27
P (td) 2.79
Q (dd) 2.74
O (ddd) 2.65
N (m) 2.50
S (ddt) 2.25
R (tdd) 2.32
T (ddd) 2.14
V (m) 2.06
D1 (m) 1.99
U (dq) 1.91
W (m) 1.70
C1 (m) 1.63
Y (m) 1.57
X (m) 1.42
A1 (m) 1.36
B1 (m) 1.30
Z (ddd) 1.23
M (dtd) 1.06
I (d) 0.93
K (d) 0.85
L (d) 0.84
J (d) 0.76
f1 (ppm)

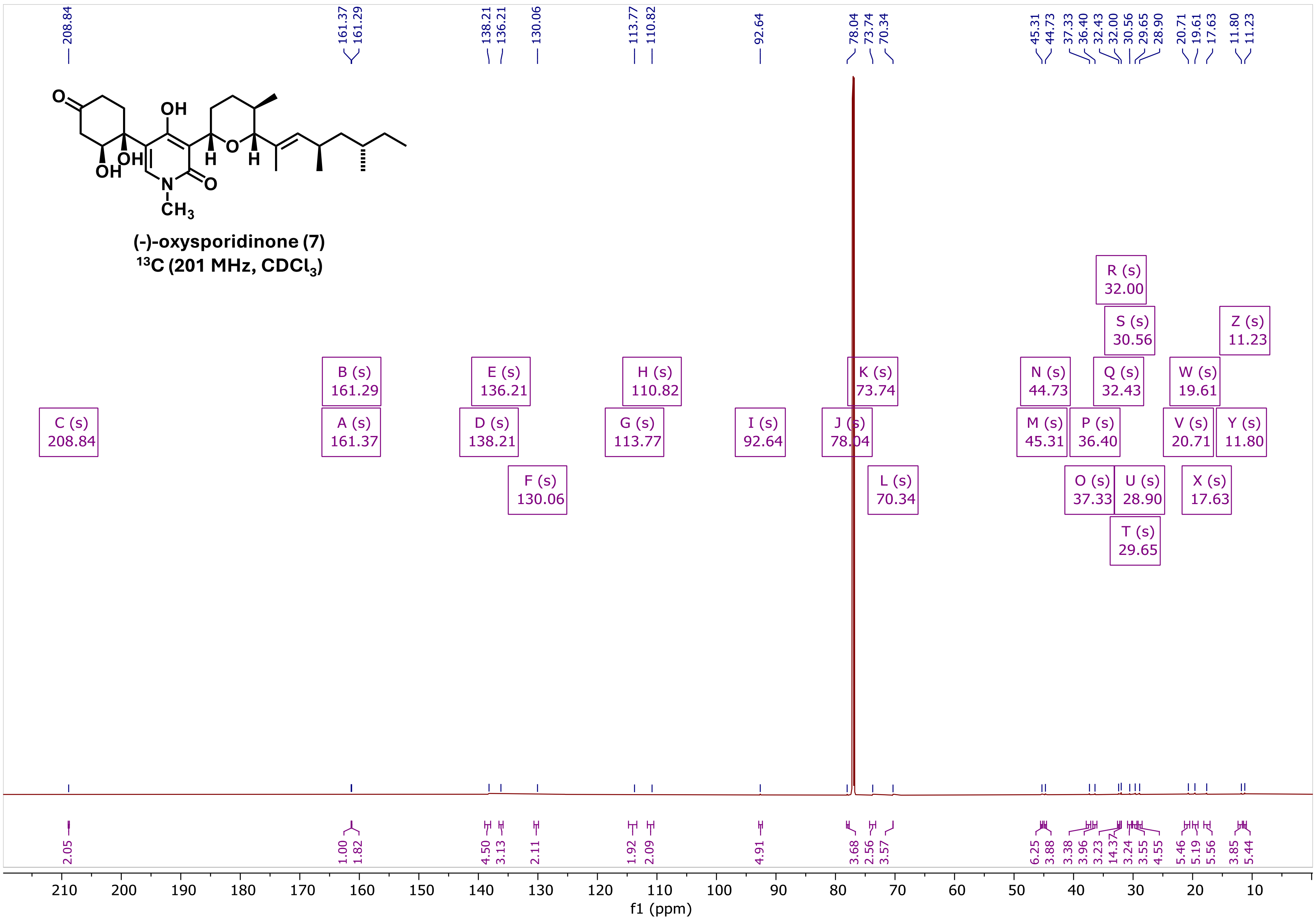

(-)-oxysporidinone (7)

^{13}C (201 MHz, $CDCl_3$)

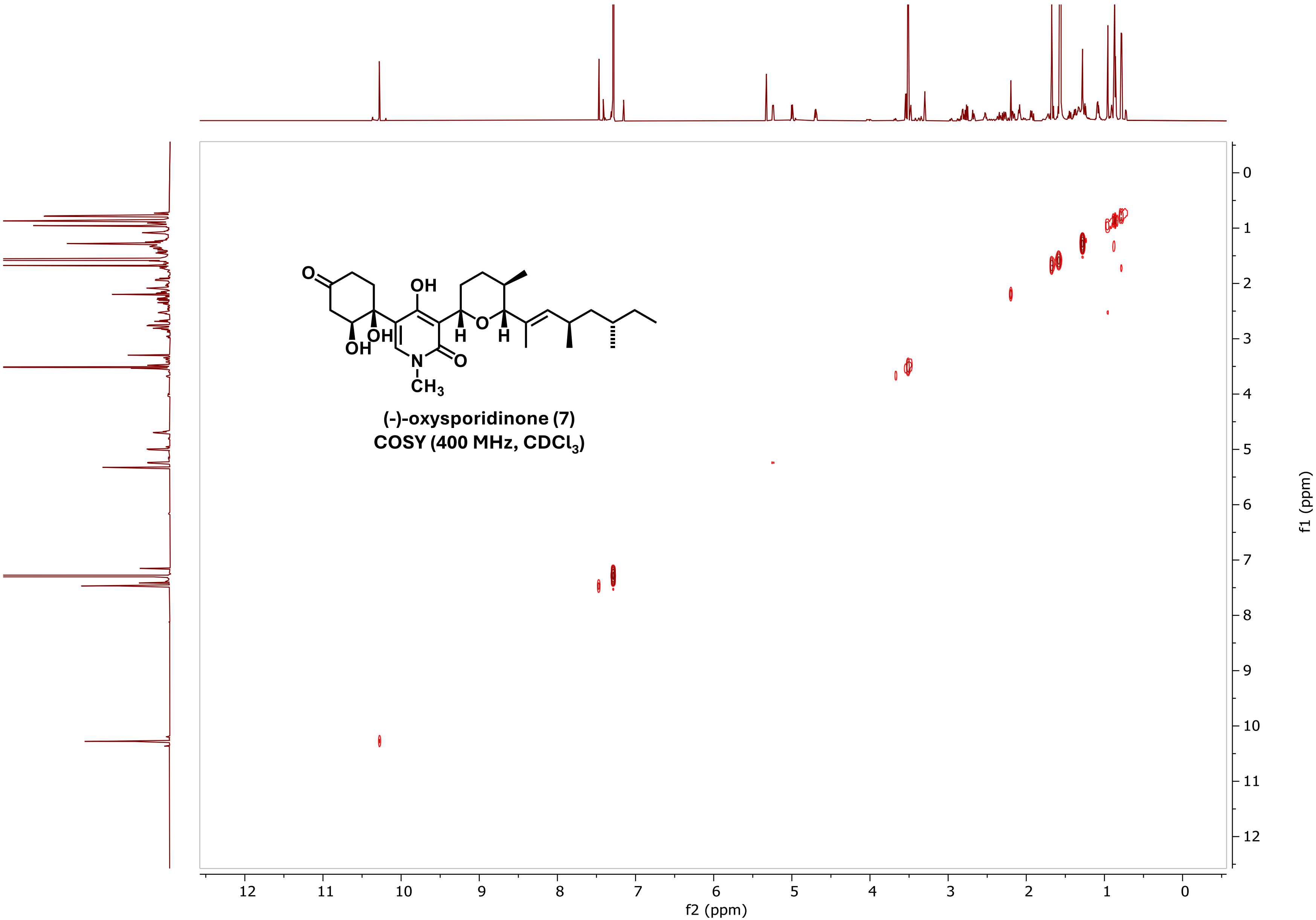
OH
O
OH
OH
H
O
H
N
O
CH3
(-)-oxysporidinone (7)
COSY (400 MHz, CDCl3)
f1 (ppm)
f2 (ppm)

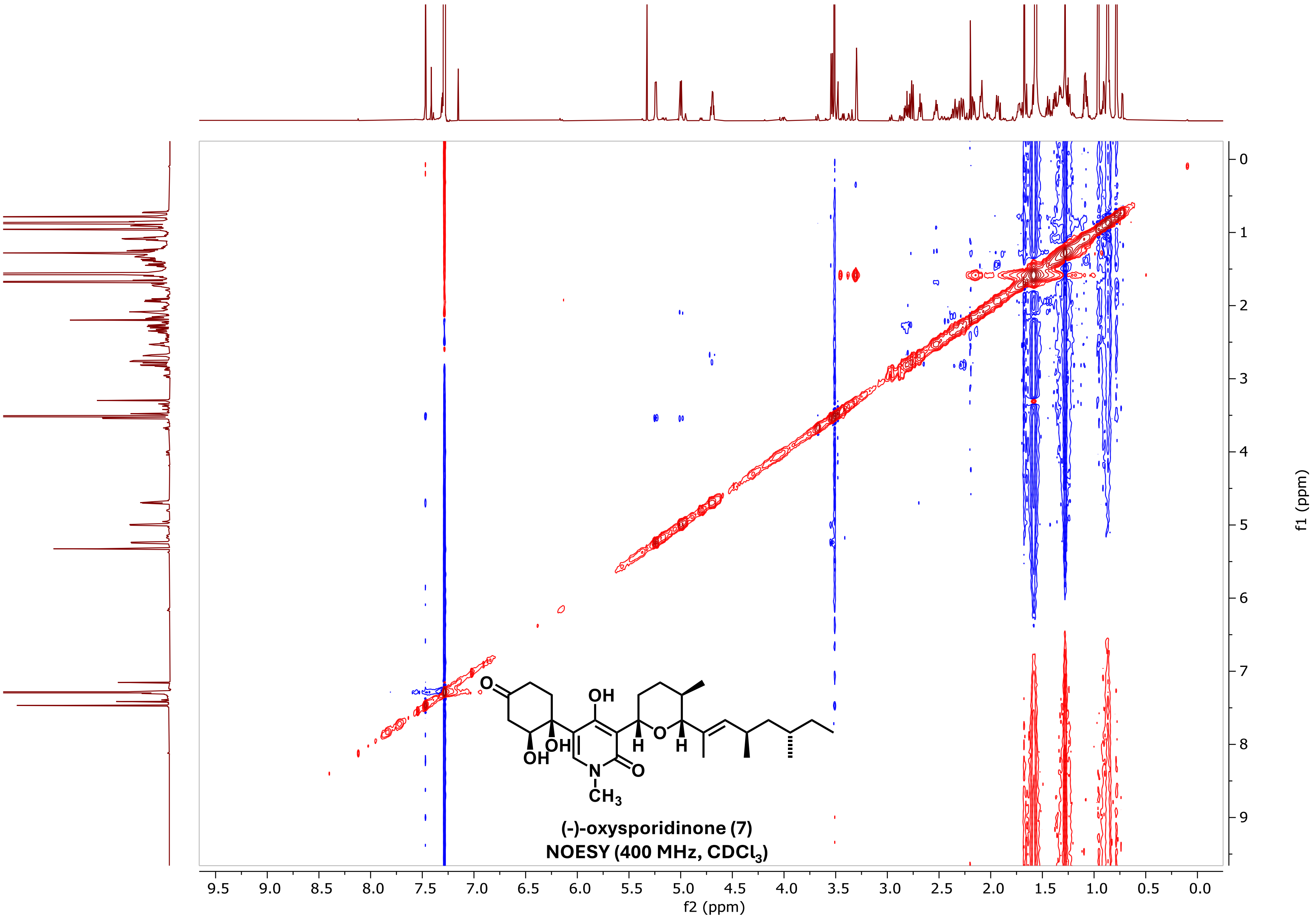
9.5 9.0 8.5 8.0 7.5 7.0 6.5 6.0 5.5 5.0 4.5 4.0 3.5 3.0 2.5 2.0 1.5 1.0 0.5 0.0
f2 (ppm)
0 1 2 3 4 5 6 7 8 9
f1 (ppm)
(-)-oxysporidinone (7)
NOESY (400 MHz, $CDCl_3$)

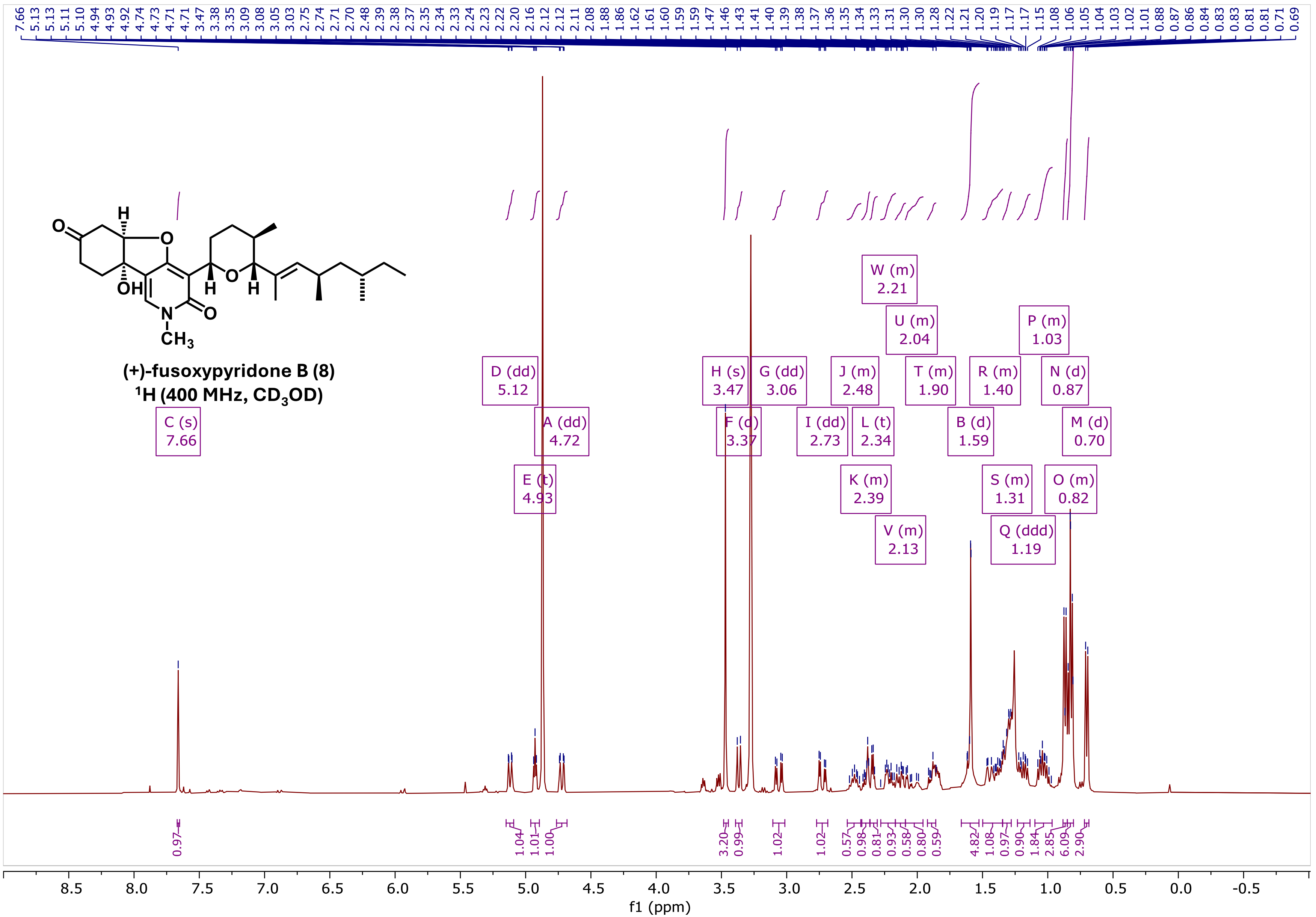

(+)-fusoxypyridone B (8)
^{1}H (400 MHz, CD$_3$OD)

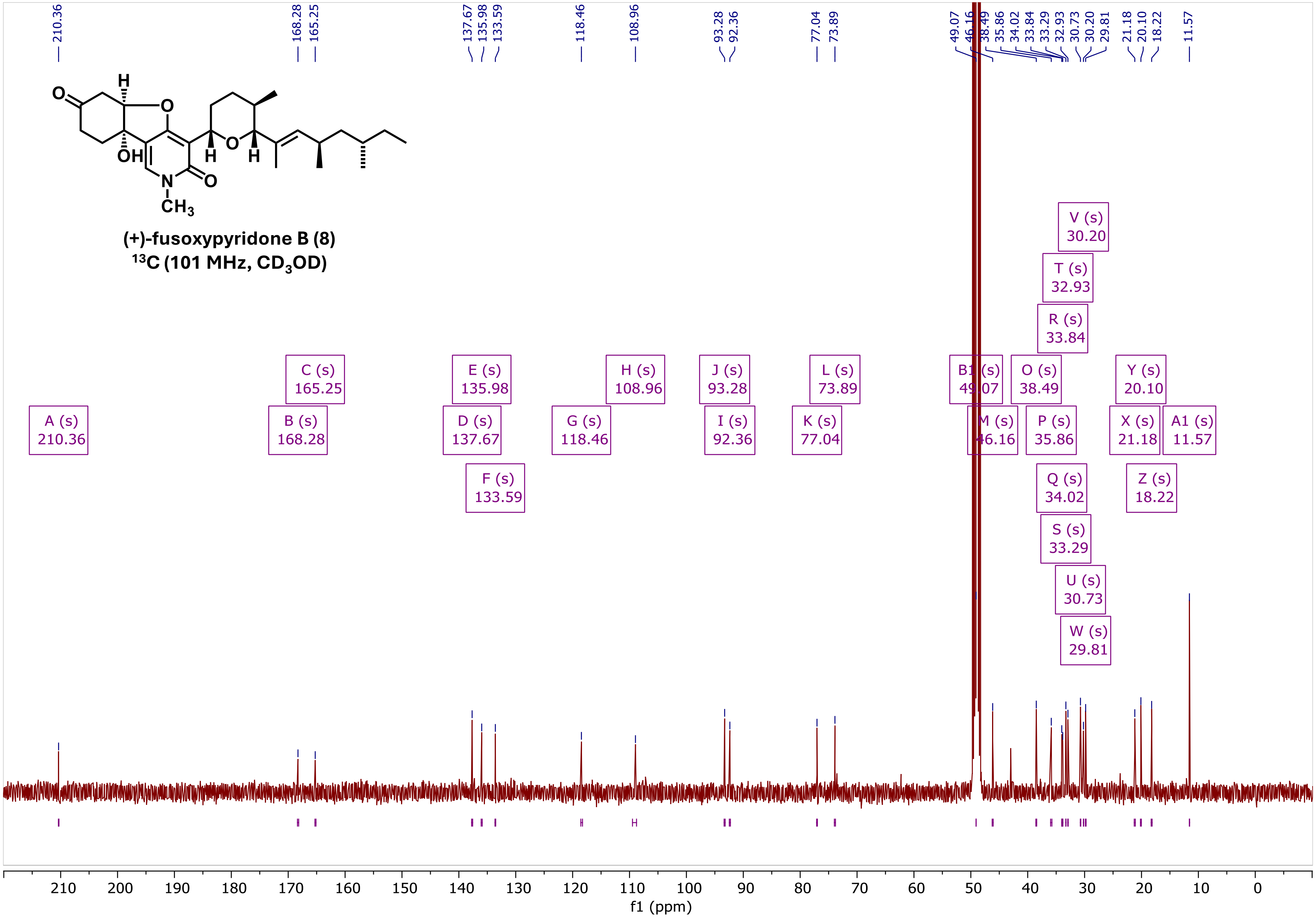
(+)-fusoxypyridone B (8)
^{13}C (101 MHz, CD_3OD)
210.36
168.28
165.25
137.67
135.98
133.59
118.46
108.96
93.28
92.36
77.04
73.89
49.07
46.16
38.49
35.86
34.02
33.84
33.29
32.93
30.73
30.20
29.81
21.18
20.10
18.22
11.57
f1 (ppm)

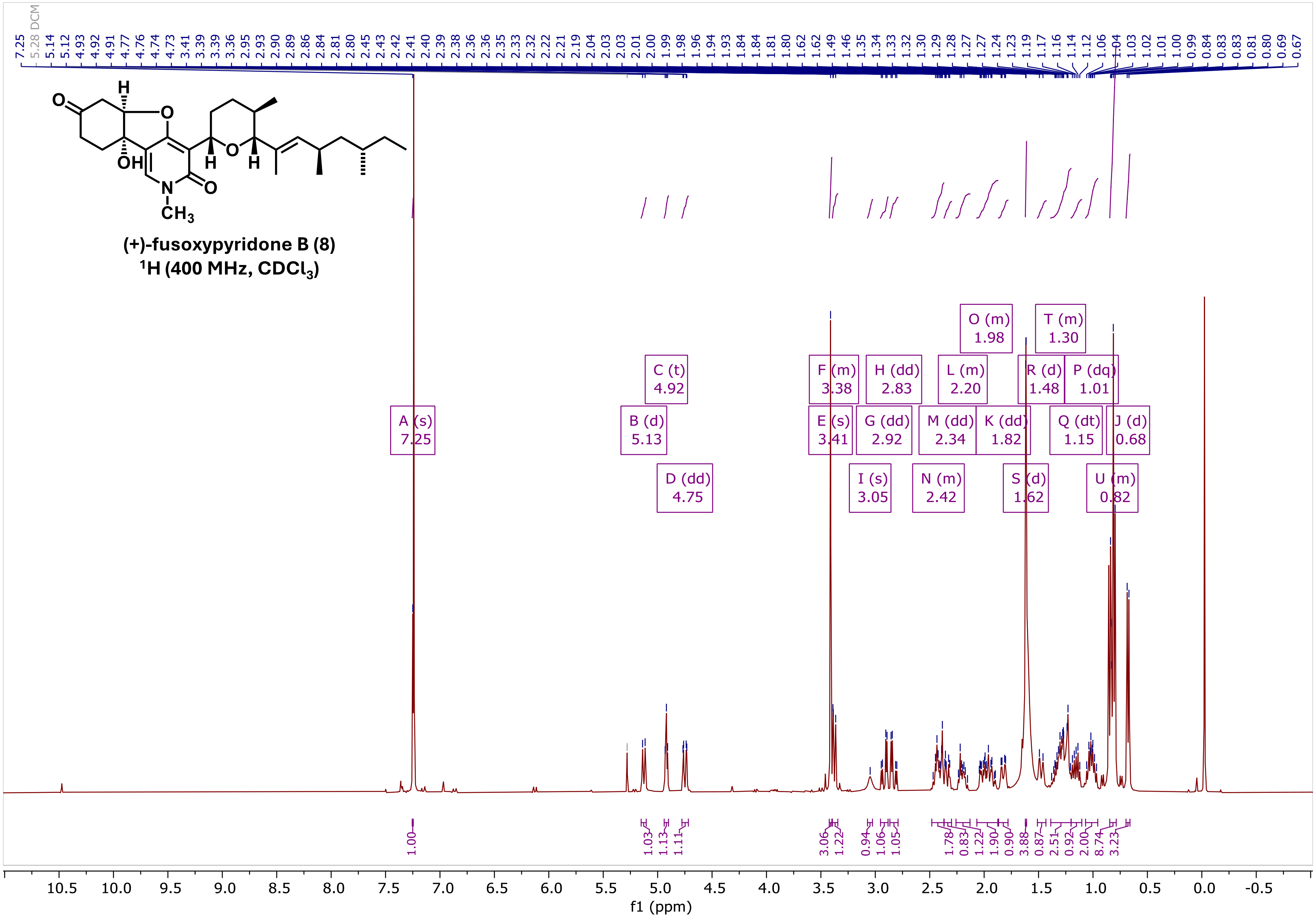

(+)-fusoxypyridone B (8)
^{1}H (400 MHz, $CDCl_3$)

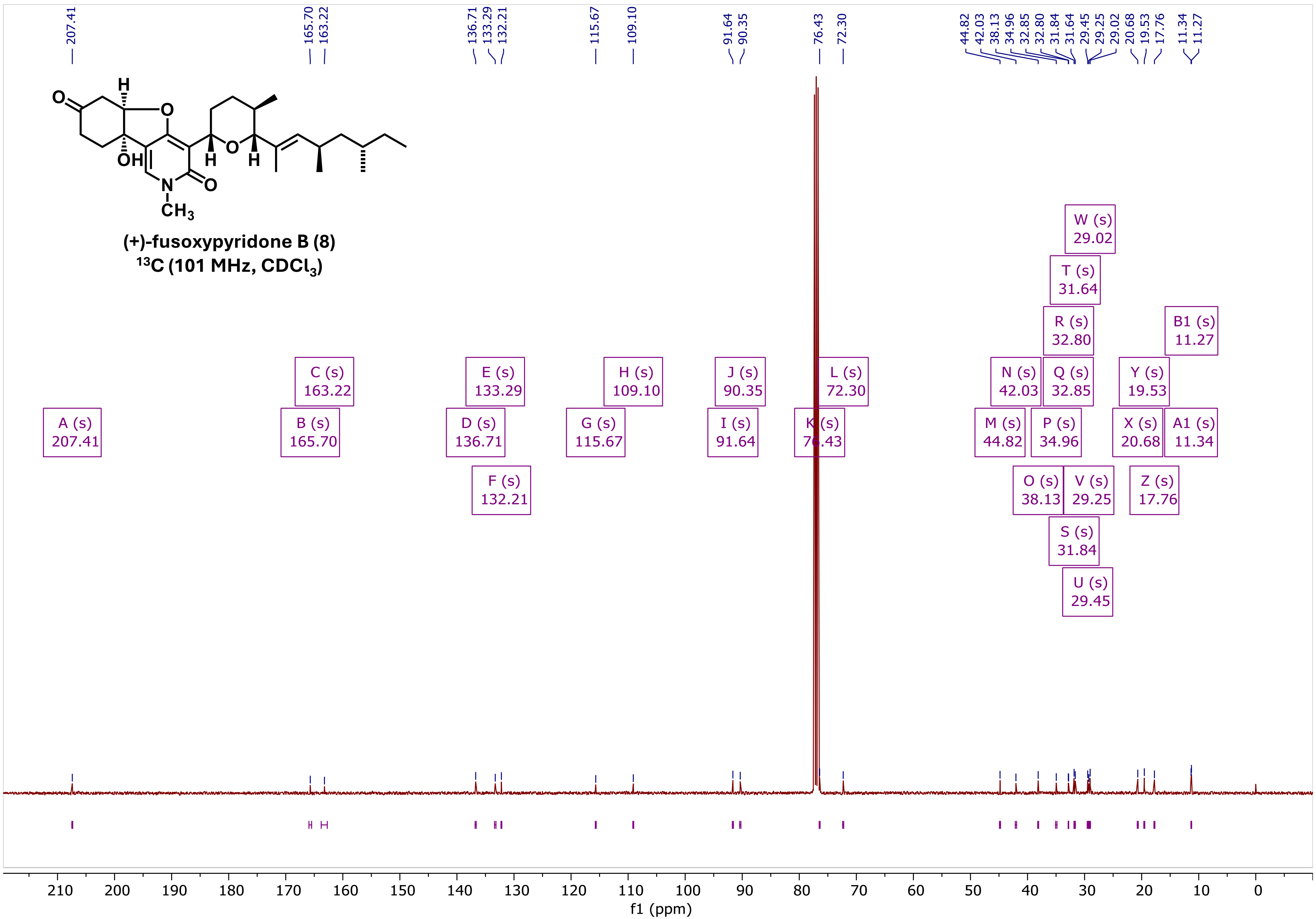

(+)-fusoxypyridone B (8)
^{13}C (101 MHz, $CDCl_3$)

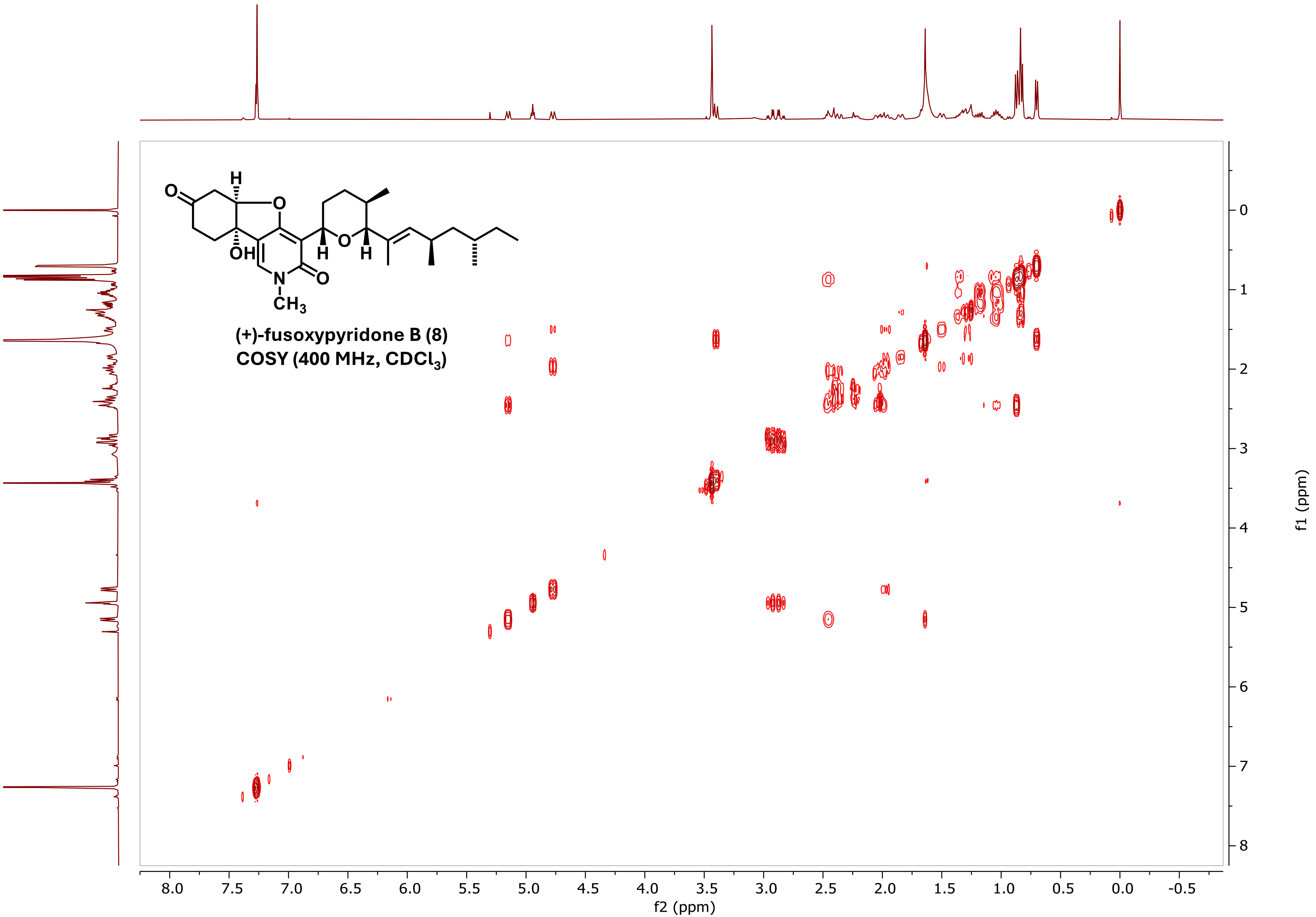

(+)-fusoxypyridone B (8)
COSY (400 MHz, CDCl3)
f2 (ppm)
f1 (ppm)

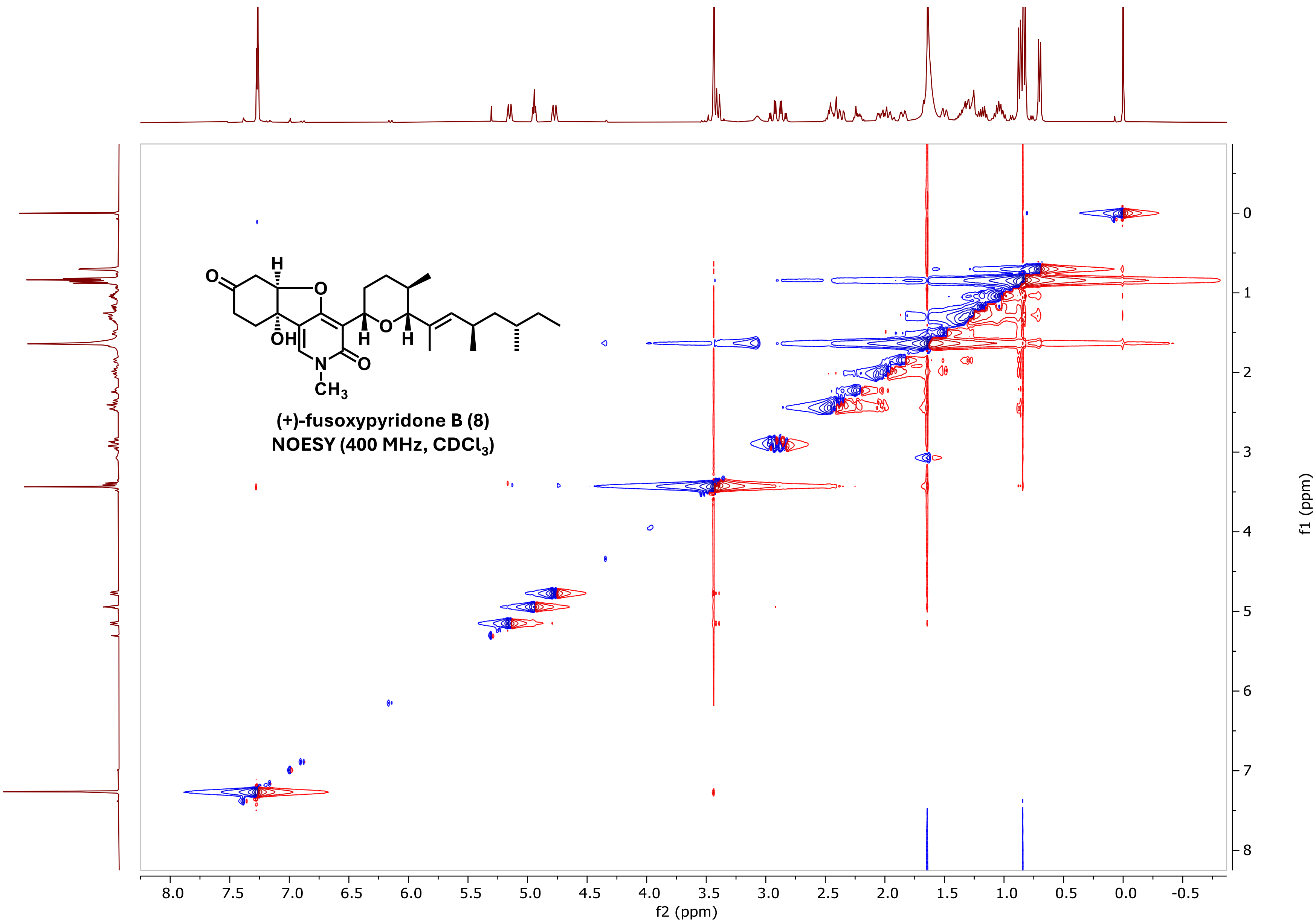
(+)-fusoxypyridone B (8)
NOESY (400 MHz, CDCl$_3$)
f2 (ppm)
f1 (ppm)

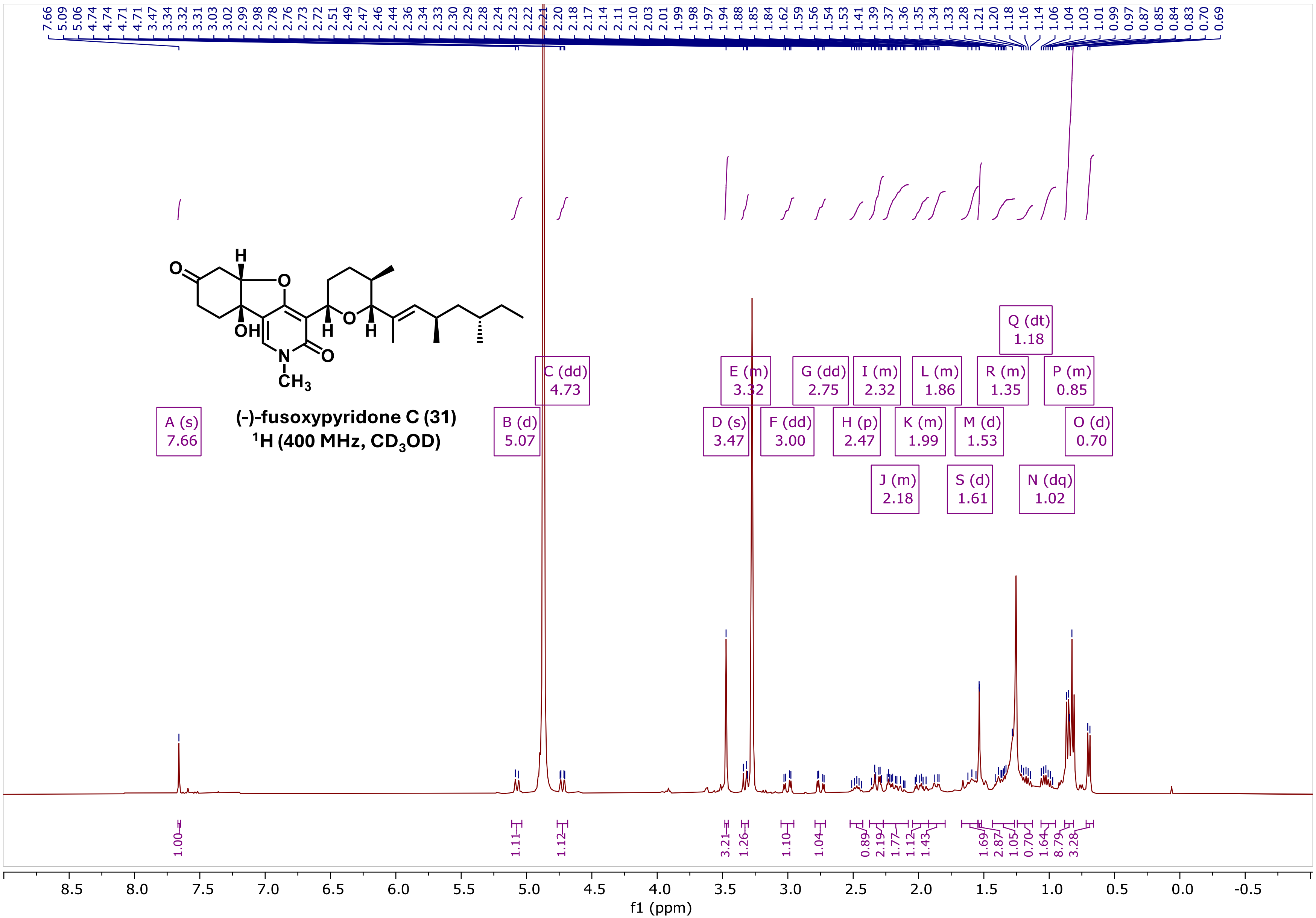

(-)-fusoxypyridone C (31)
¹H (400 MHz, CD₃OD)
7.66 5.09 5.06 4.74 4.74 4.71 4.71 3.47 3.34 3.32 3.31 3.03 3.02 2.99 2.98 2.78 2.76 2.73 2.72 2.51 2.49 2.47 2.46 2.44 2.36 2.34 2.33 2.30 2.29 2.28 2.24 2.23 2.22 2.21 2.20 2.18 2.17 2.14 2.11 2.10 2.03 2.01 1.99 1.98 1.97 1.94 1.88 1.85 1.84 1.62 1.59 1.56 1.54 1.53 1.41 1.39 1.37 1.36 1.35 1.34 1.33 1.28 1.21 1.20 1.18 1.16 1.14 1.06 1.04 1.03 1.01 0.99 0.97 0.87 0.85 0.84 0.83 0.70 0.69
A (s) 7.66
B (d) 5.07
C (dd) 4.73
D (s) 3.47
E (m) 3.32
F (dd) 3.00
G (dd) 2.75
H (p) 2.47
I (m) 2.32
J (m) 2.18
K (m) 1.99
L (m) 1.86
M (d) 1.53
S (d) 1.61
R (m) 1.35
Q (dt) 1.18
N (dq) 1.02
P (m) 0.85
O (d) 0.70
1.00 1.11 1.12 3.21 1.26 1.10 1.04 0.89 2.19 1.77 1.12 1.43 1.69 2.87 1.05 0.70 1.64 8.79 3.28
8.5 8.0 7.5 7.0 6.5 6.0 5.5 5.0 4.5 4.0 3.5 3.0 2.5 2.0 1.5 1.0 0.5 0.0 -0.5
f1 (ppm)

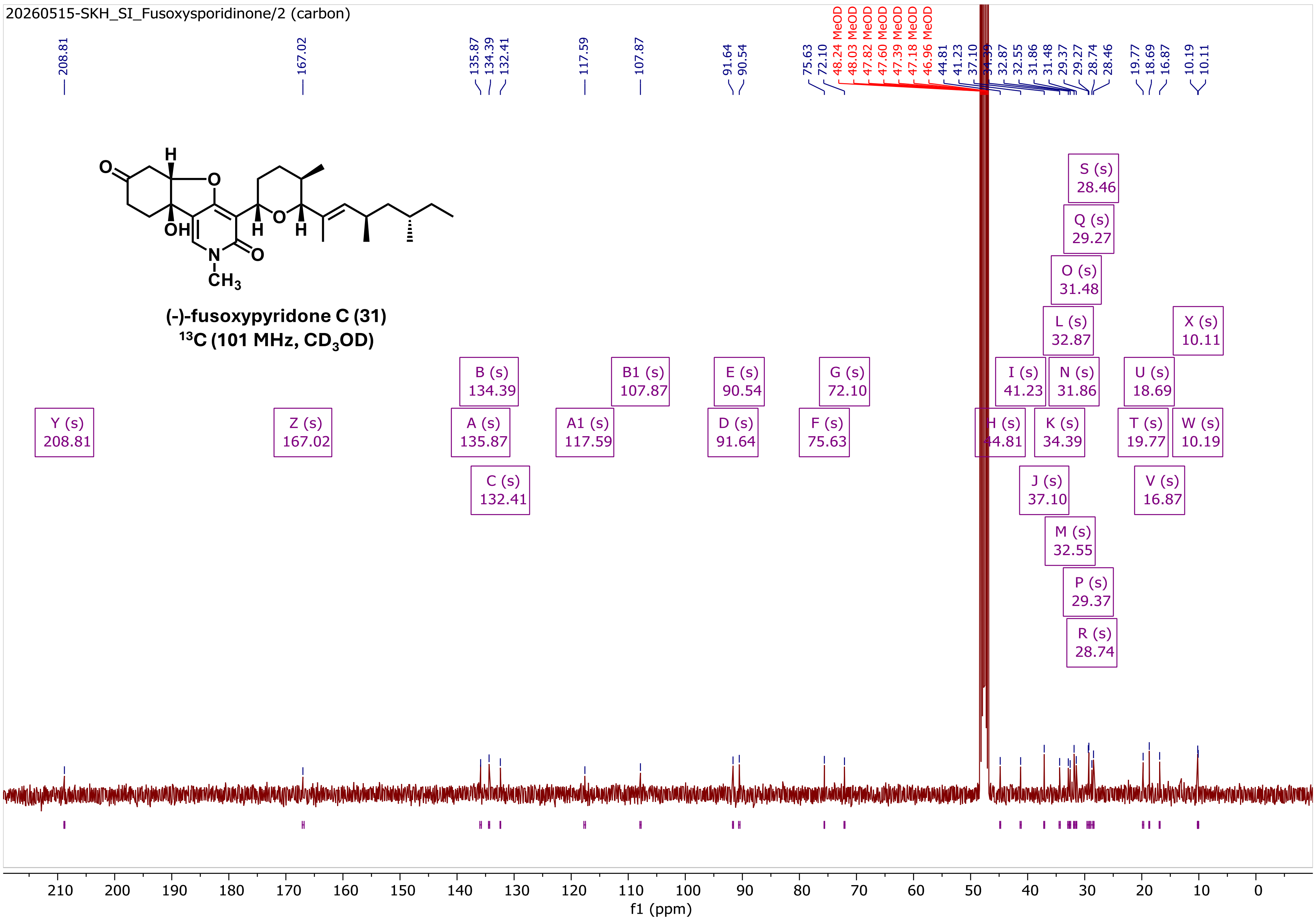
20260515-SKH_SI_Fusoxysporidinone/2 (carbon)
208.81
167.02
135.87
134.39
132.41
117.59
107.87
91.64
90.54
75.63
72.10
48.24 MeOD
48.03 MeOD
47.82 MeOD
47.60 MeOD
47.39 MeOD
47.18 MeOD
46.96 MeOD
44.81
41.23
37.10
34.39
32.87
32.55
31.86
31.48
29.37
29.27
28.74
28.46
19.77
18.69
16.87
10.19
10.11
(-)-fusoxypyridone C (31)
^{13}C (101 MHz, CD_3OD)
Y (s) 208.81
Z (s) 167.02
A (s) 135.87
B (s) 134.39
C (s) 132.41
A1 (s) 117.59
B1 (s) 107.87
D (s) 91.64
E (s) 90.54
F (s) 75.63
G (s) 72.10
H (s) 44.81
I (s) 41.23
J (s) 37.10
K (s) 34.39
L (s) 32.87
M (s) 32.55
N (s) 31.86
O (s) 31.48
P (s) 29.37
Q (s) 29.27
R (s) 28.74
S (s) 28.46
T (s) 19.77
U (s) 18.69
V (s) 16.87
W (s) 10.19
X (s) 10.11
210
200
190
180
170
160
150
140
130
120
110
100
90
80
70
60
50
40
30
20
10
0
f1 (ppm)

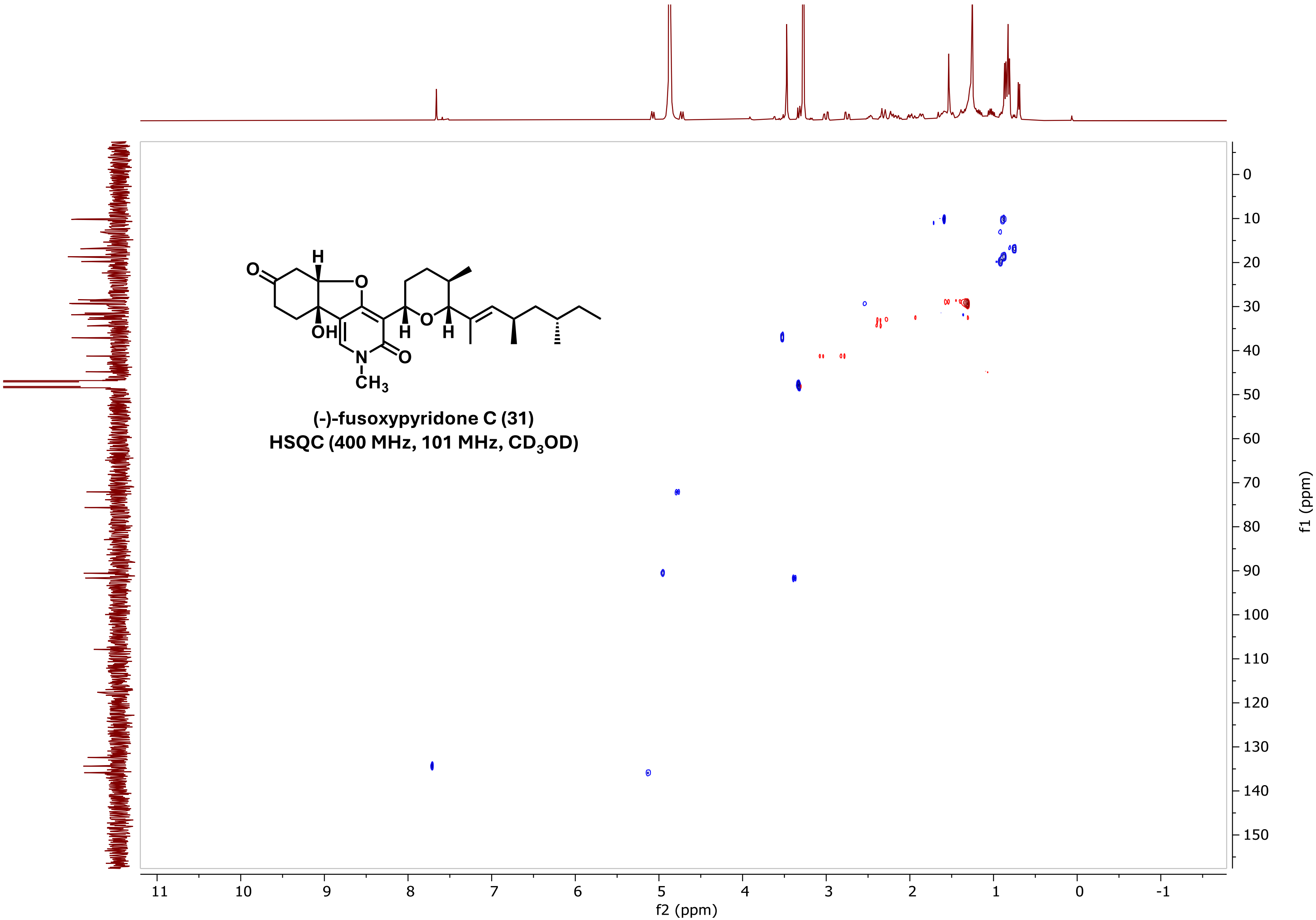
(-)-fusoxypyridone C (31)
HSQC (400 MHz, 101 MHz, CD_3OD)
f2 (ppm)
f1 (ppm)

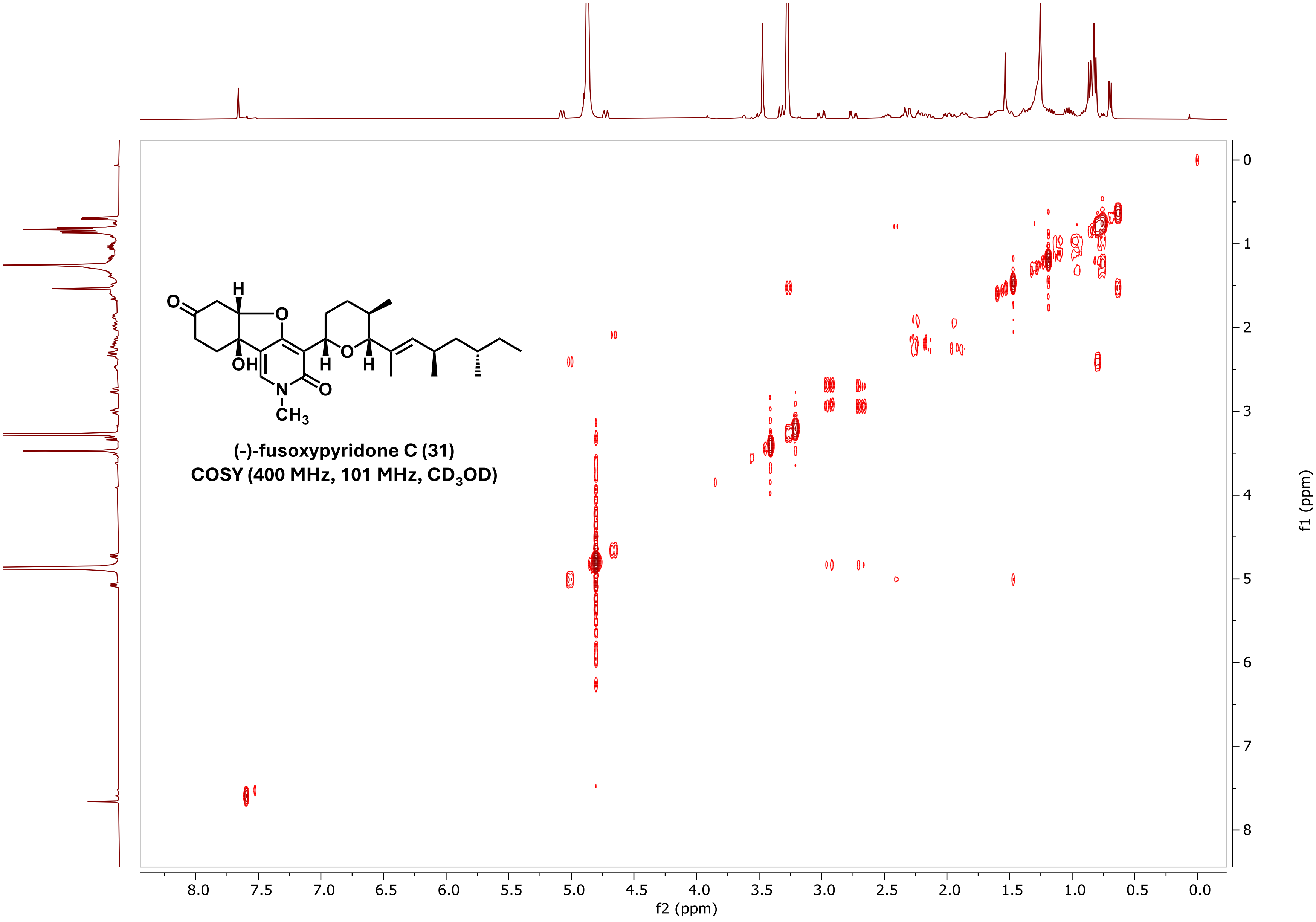

(-)-fusoxypyridone C (31)
COSY (400 MHz, 101 MHz, CD_3OD)
f2 (ppm)
f1 (ppm)

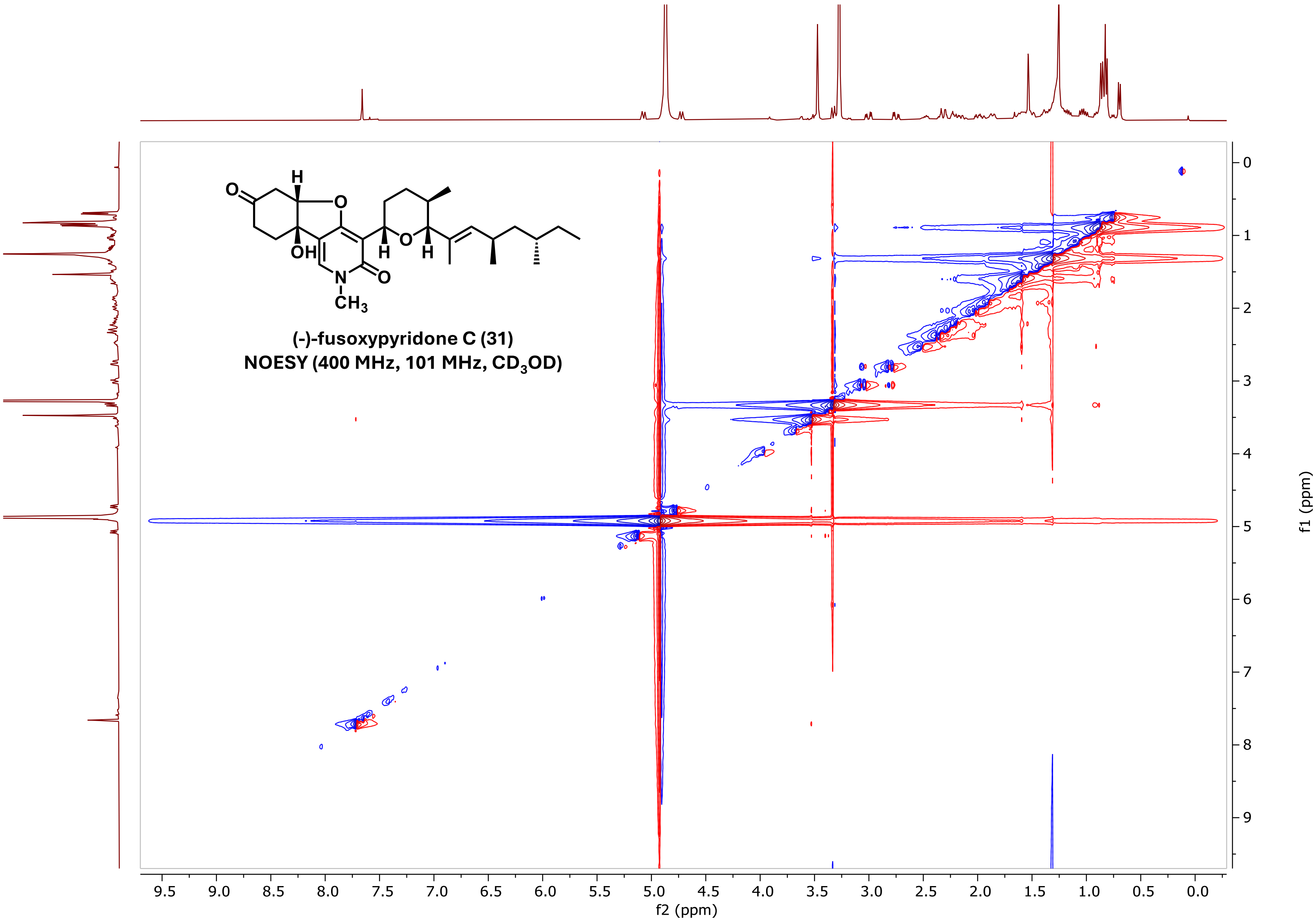

(-)-fusoxypyridone C (31)
NOESY (400 MHz, 101 MHz, CD3OD)
f2 (ppm)
f1 (ppm)

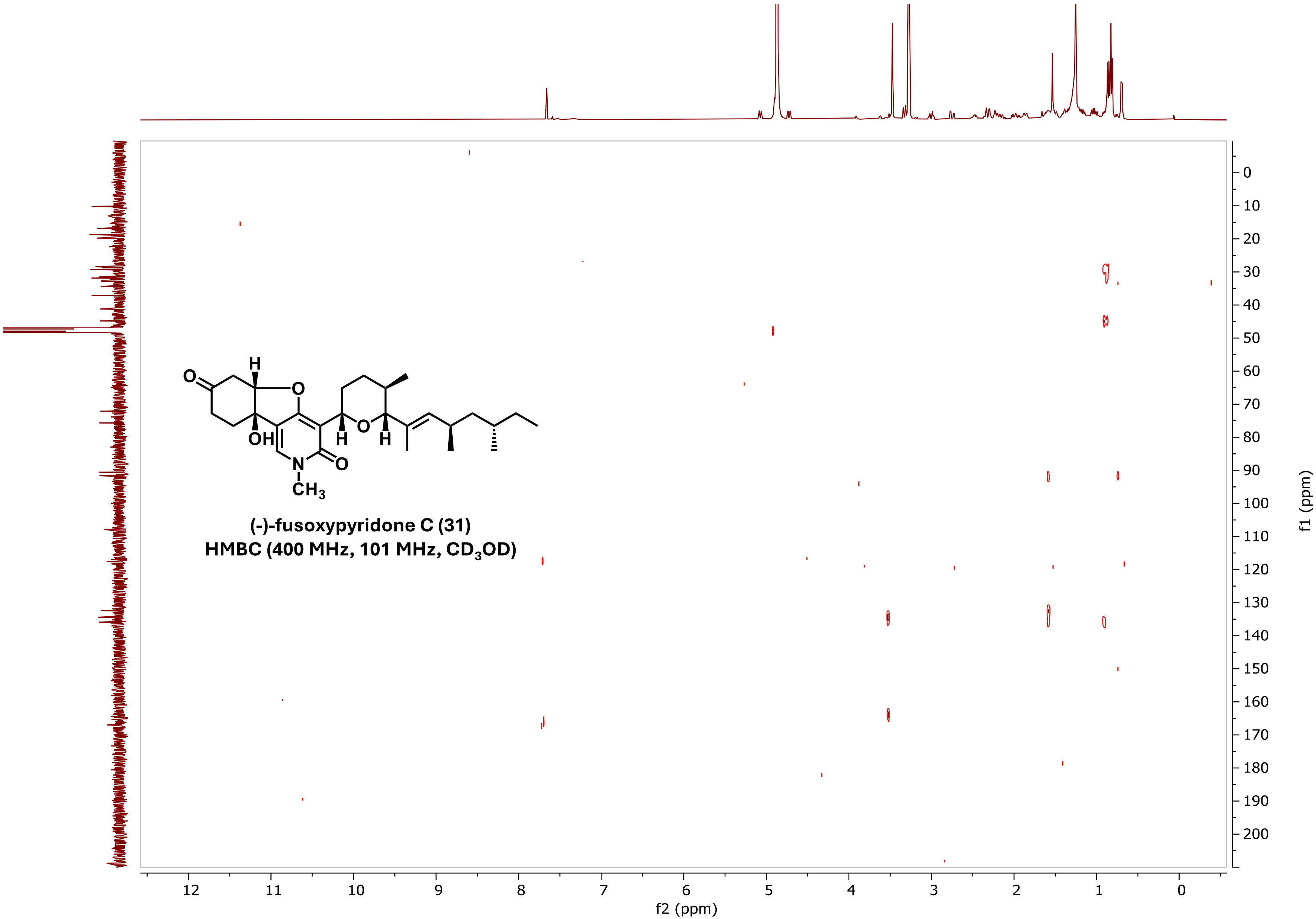
(-)-fusoxypyridone C (31)
HMBC (400 MHz, 101 MHz, CD_3OD)
f2 (ppm)
f1 (ppm)

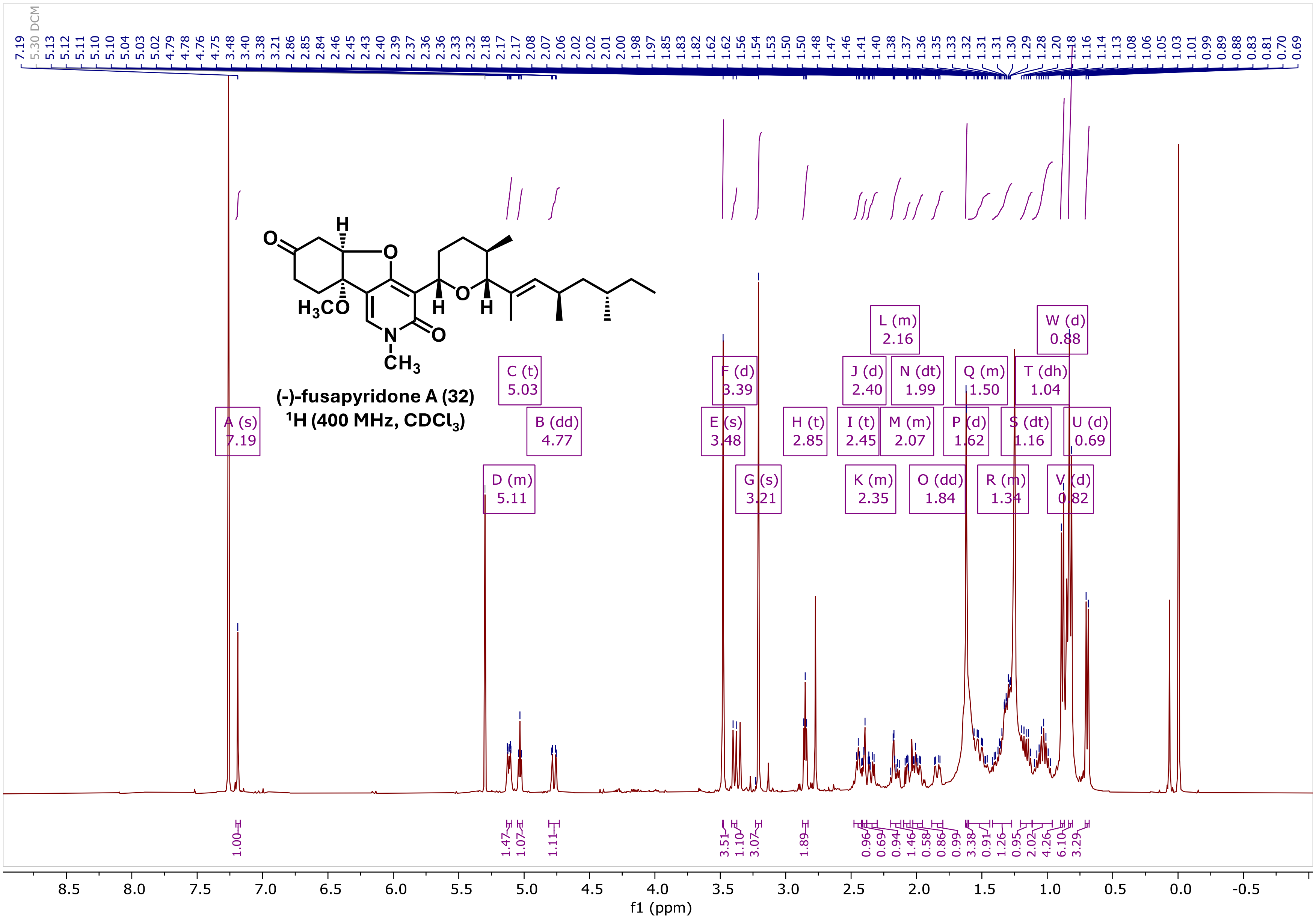
(-)-fusapyridone A (32)
1H (400 MHz, CDCl3)
A (s) 7.19
B (dd) 4.77
C (t) 5.03
D (m) 5.11
E (s) 3.48
F (d) 3.39
G (s) 3.21
H (t) 2.85
I (t) 2.45
J (d) 2.40
K (m) 2.35
L (m) 2.16
M (m) 2.07
N (dt) 1.99
O (dd) 1.84
P (d) 1.62
Q (m) 1.50
R (m) 1.34
S (dt) 1.16
T (dh) 1.04
U (d) 0.69
V (d) 0.82
W (d) 0.88
f1 (ppm)

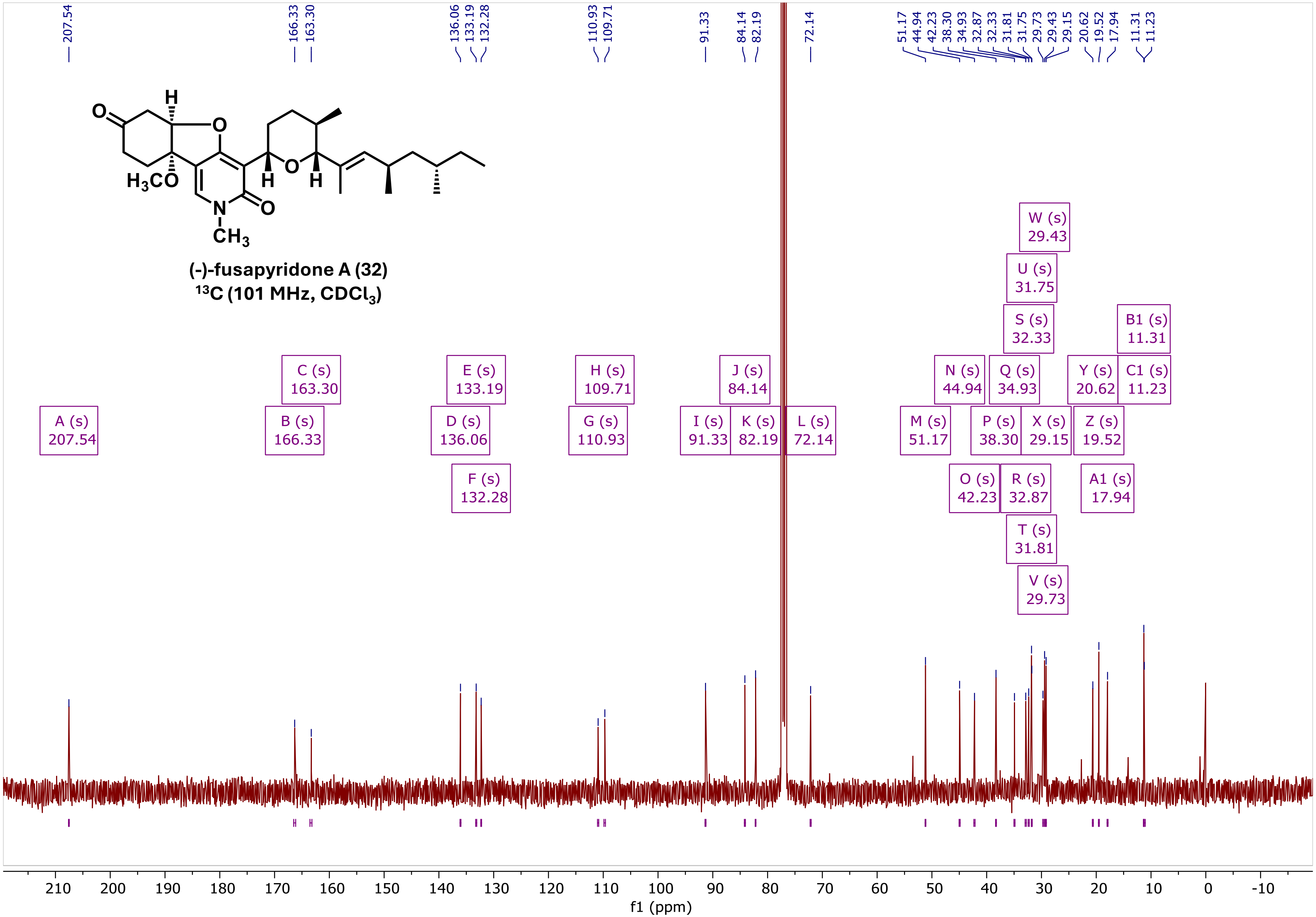
207.54
166.33
163.30
136.06
133.19
132.28
110.93
109.71
91.33
84.14
82.19
72.14
51.17
44.94
42.23
38.30
34.93
32.87
32.33
31.81
31.75
29.73
29.43
29.15
20.62
19.52
17.94
11.31
11.23
(-)-fusapyridone A (32)
13C (101 MHz, CDCl3)
A (s) 207.54
B (s) 166.33
C (s) 163.30
D (s) 136.06
E (s) 133.19
F (s) 132.28
G (s) 110.93
H (s) 109.71
I (s) 91.33
J (s) 84.14
K (s) 82.19
L (s) 72.14
M (s) 51.17
N (s) 44.94
O (s) 42.23
P (s) 38.30
Q (s) 34.93
R (s) 32.87
S (s) 32.33
T (s) 31.81
U (s) 31.75
V (s) 29.73
W (s) 29.43
X (s) 29.15
Y (s) 20.62
Z (s) 19.52
A1 (s) 17.94
B1 (s) 11.31
C1 (s) 11.23
f1 (ppm)

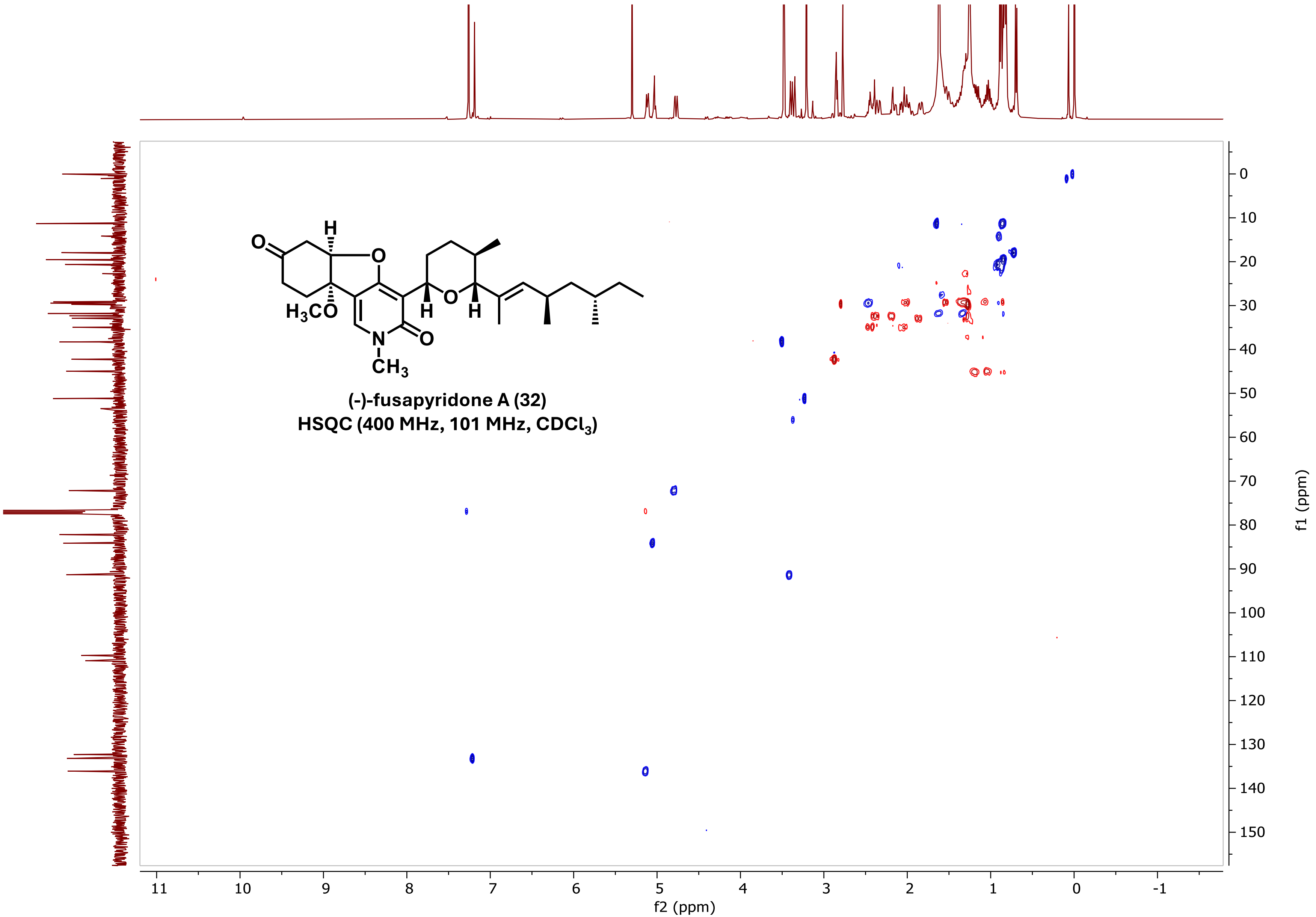

(-)-fusapyridone A (32)
HSQC (400 MHz, 101 MHz, $CDCl_3$)
f2 (ppm)
f1 (ppm)

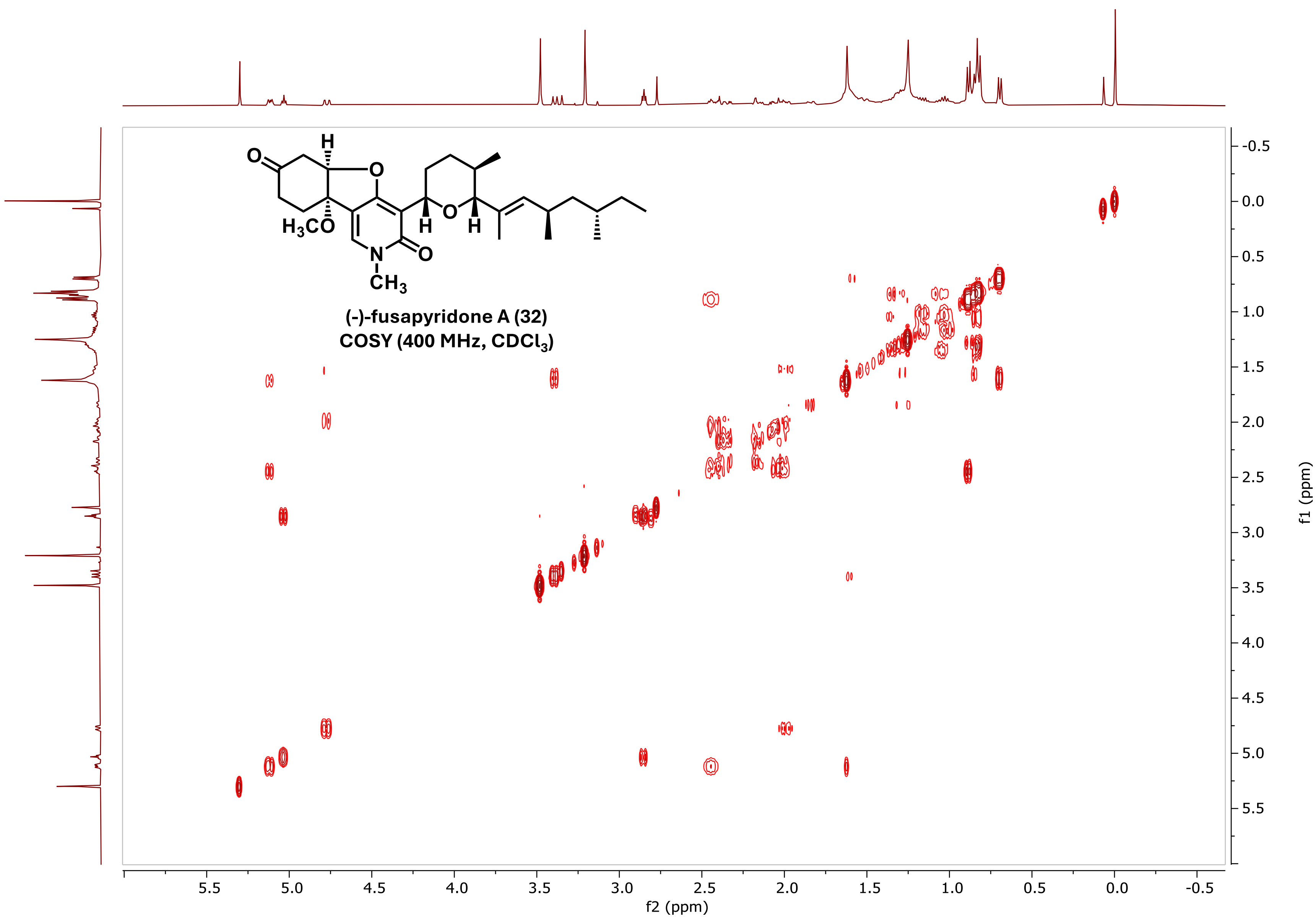

O
H
O
H3CO
N
CH3
O
O
H
H
(-)-fusapyridone A (32)
COSY (400 MHz, CDCl3)
f2 (ppm)
f1 (ppm)

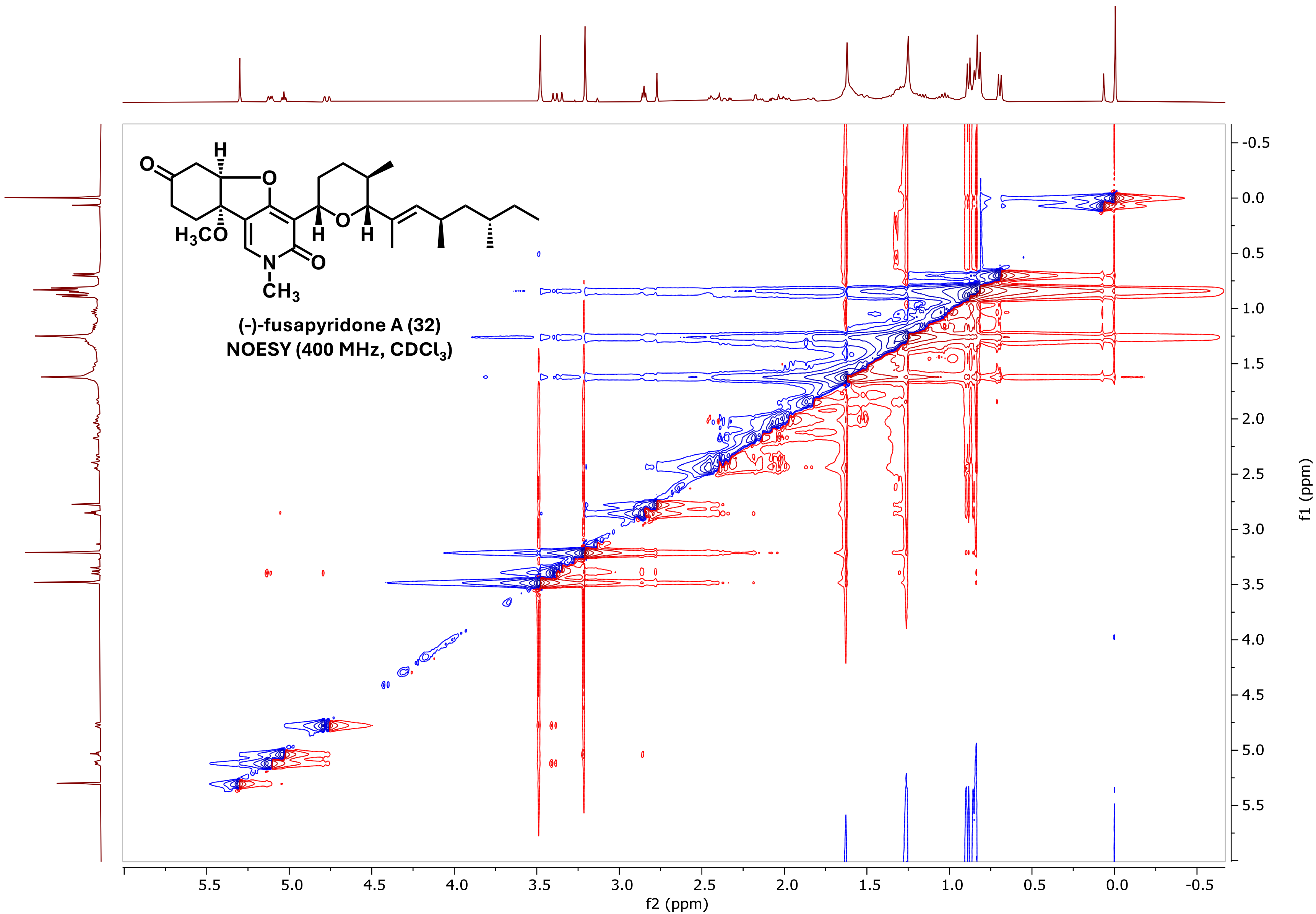

(-)-fusapyridone A (32)
NOESY (400 MHz, CDCl3)
f2 (ppm)
f1 (ppm)

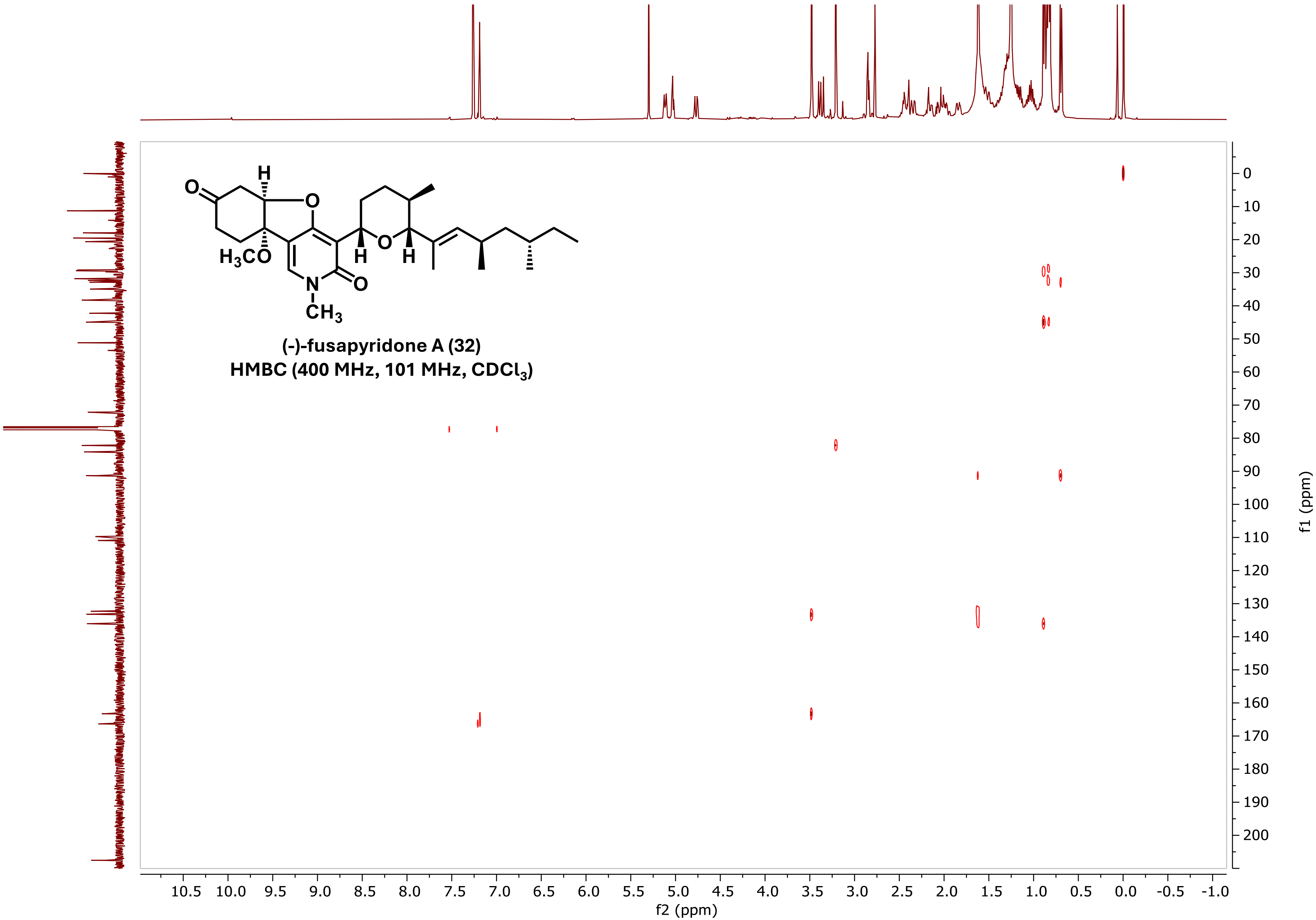

(-)-fusapyridone A (32)
HMBC (400 MHz, 101 MHz, CDCl3)
f2 (ppm)
f1 (ppm)
